# Maternal influences on infant gut microbiome and health

**DOI:** 10.1038/s41586-026-10922-9

**Published:** 2026-08-12

**Authors:** Trishla Sinha, Siobhan Brushett, Asier Fernández-Pato, Sanzhima Garmaeva, Sergio Andreu-Sánchez, Johanne E. Spreckels, Cyrus A. Mallon, Nataliia Kuzub, Milla Brandao Gois, Jiafei Wu, Marloes Kruk, Soesma A. Jankipersadsing, Jackie A. M. Dekens, Ranko Gacesa, Arnau Vich Vila, Corinna Bang, Corine Perenboom, Andre Franke, Hanne L. P. Tytgat, Sara Colombo Mottaz, Lilian Peters, Ank de Jonge, Henkjan J. Verkade, Morris A. Swertz, Cisca Wijmenga, Folkert Kuipers, Sicco Scherjon, Jan Sikkema, Aline B. Sprikkelman, Marlou L. A. de Kroon, Jelmer R. Prins, Sanne J. Gordijn, Gerard H. Koppelman, Sijmen A. Reijneveld, Lifelines NEXT cohort study, Trishla Sinha, Siobhan Brushett, Sanzhima Garmaeva, Johanne E. Spreckels, Soesma A. Jankipersadsing, Jackie A. M. Dekens, Lilian Peters, Ank de Jonge, Henkjan J. Verkade, Morris A. Swertz, Cisca Wijmenga, Folkert Kuipers, Sicco Scherjon, Jan Sikkema, Aline B. Sprikkelman, Marlou L. A. de Kroon, Jelmer R. Prins, Sanne J. Gordijn, Gerard H. Koppelman, Sijmen A. Reijneveld, Milla F. Brandão-Gois, Marcel Bruinenberg, Anouk Marsman, Alexander Kurilshikov, Alexandra Zhernakova, Jingyuan Fu, Moran Yassour, Alexander Kurilshikov, Alexandra Zhernakova

**Affiliations:** 1 Department of Genetics, University of Groningen and University Medical Center Groningen, Groningen, The Netherlands; 2 Department of Health Sciences, University of Groningen and University Medical Center Groningen, Groningen, The Netherlands; 3 Department of Pediatrics, University of Groningen and University Medical Center Groningen, Groningen, The Netherlands; 4 UMCG Innovation Center, University Medical Center Groningen, Groningen, The Netherlands; 5 Department of Gastroenterology and Hepatology, University of Groningen and University Medical Center Groningen, Groningen, The Netherlands; 6 Laboratory of Molecular Bacteriology, Department of Microbiology, Immunology and Transplantation, Rega Institute for Medical Research, KU Leuven, Leuven, Belgium; 7 Laboratory of Bioinformatics and (Eco-)Systems Biology, Center for Microbiology, VIB, Leuven, Belgium; 8 Institute for Clinical Molecular Biology, University Hospital Schleswig-Holstein, Kiel University, Kiel, Germany; 9 Division of Human Nutrition and Health, Wageningen University, Wageningen, The Netherlands; 10 Nestlé Institute of Health Sciences, Nestlé Research, Société des Produits Nestlé, Lausanne, Switzerland; 11 Nestlé Nutrition, Société des Produits Nestlé, Vevey, Switzerland; 12 Department of Primary and Long-term Care, University of Groningen and University Medical Center Groningen, Groningen, The Netherlands; 13 Midwifery Science, Amsterdam University Medical Center, Vrije Universiteit Amsterdam, AVAG, Amsterdam Public Health, Amsterdam, The Netherlands; 14 European Research Institute for the Biology of Ageing (ERIBA), University of Groningen and University Medical Center Groningen, Groningen, The Netherlands; 15 Department of Obstetrics and Gynecology, University Medical Center Groningen, University of Groningen, Groningen, The Netherlands; 16 Groningen Research Institute for Asthma and COPD (GRIAC), University of Groningen and University Medical Center Groningen, Groningen, The Netherlands; 17 Environment and Health, Department of Public Health and Primary Care, KU Leuven, Leuven, Belgium; 18 Microbiology & Molecular Genetics Department, Faculty of Medicine, The Hebrew University of Jerusalem, Jerusalem, Israel; 19 The Rachel and Selim Benin School of Computer Science and Engineering, The Hebrew University of Jerusalem, Jerusalem, Israel; 20 Computational Medicine Center, Faculty of Medicine, The Hebrew University of Jerusalem, Jerusalem, Israel; 21 Lifelines Cohort Study and Biobank, Groningen, The Netherlands

**Keywords:** Metagenomics, Microbiome

## Abstract

The establishment of the infant gut microbiome is critical for later health^1,2^, yet how it is shaped by maternal and early-life factors remains unclear. Here we metagenomically sequenced 4,526 longitudinal faecal samples from 714 mother–infant pairs in the Dutch birth cohort Lifelines NEXT, spanning 12 weeks of pregnancy to 1 year postpartum. We integrated these data with 474 clinical and exposure variables, and with ultra-deep sequencing of breast milk and vaginal microbiomes. We observe that the maternal gut microbiome undergoes only subtle changes during pregnancy and postpartum, influenced by diet, infections and pre-pregnancy smoking. The maternal gut microbiome is a major reservoir for infant gut strains, with only occasional transmission from vaginal and breast milk microbiomes. Mother–infant gut strain sharing is time dependent, and higher maternal gut species abundance increases the likelihood of strain transmission. We find that the maternal gut microbiome is a predictor of infant eczema. Mode of delivery and feeding mode primarily shaped the infant gut microbiome and its functional profiles, with maternal exposures also having a role. Of 585 vaginally delivered infants, 155 were born at home, but home delivery was only moderately associated with infant gut microbiome composition, similar to other birth parameters such as duration of pushing and ruptured membranes. Overall, we highlight the central role of the mother and her microbiome in shaping the infant gut ecosystem and early health outcomes.

## Abstract (web summary)

The maternal gut microbiome is a predictor of infant eczema.

## Main

The establishment and evolution of the infant gut microbiome have a vital role in early immune development and later health outcomes^1,2^. During early life, the gut microbiome transitions from a largely uninhabited ecosystem to a complex microbial community, shaped by interactions between host and environmental factors^3–5^. Previous research has predominantly focused on individual determinants of infant gut microbiome development, including mode of delivery^6–10^, infant feeding practices^3,11,12^, antibiotic exposure^9,10^ maternal diet^13–15^ and pet exposure^16^.

However, an integrated understanding of how multiple pre-, peri- and postnatal exposures jointly shape infant gut microbiome development over time is lacking. In particular, relatively few studies^17^ have investigated the role of maternal exposures and the maternal gut microbiome during pregnancy in shaping the infant gut microbiome composition and health outcomes. Moreover, although mother-to-infant strain sharing is a hallmark of early microbial transmission^6,18–22^, the maternal sources and transmission routes identified through strain-resolved metagenomics remain incompletely understood.

Here, to address these gaps, we analysed 714 mother–infant pairs from the prospective, deeply phenotyped Dutch birth cohort Lifelines NEXT (LLNEXT)^23^. We characterized gut microbiome dynamics during pregnancy and infancy, examined associations with pre-pregnancy, delivery, dietary and health-related factors, and investigated maternal–infant microbial strain transmission across multiple maternal body sites.

## Mother–infant gut microbiome in LLNEXT

**Fig. 1:**
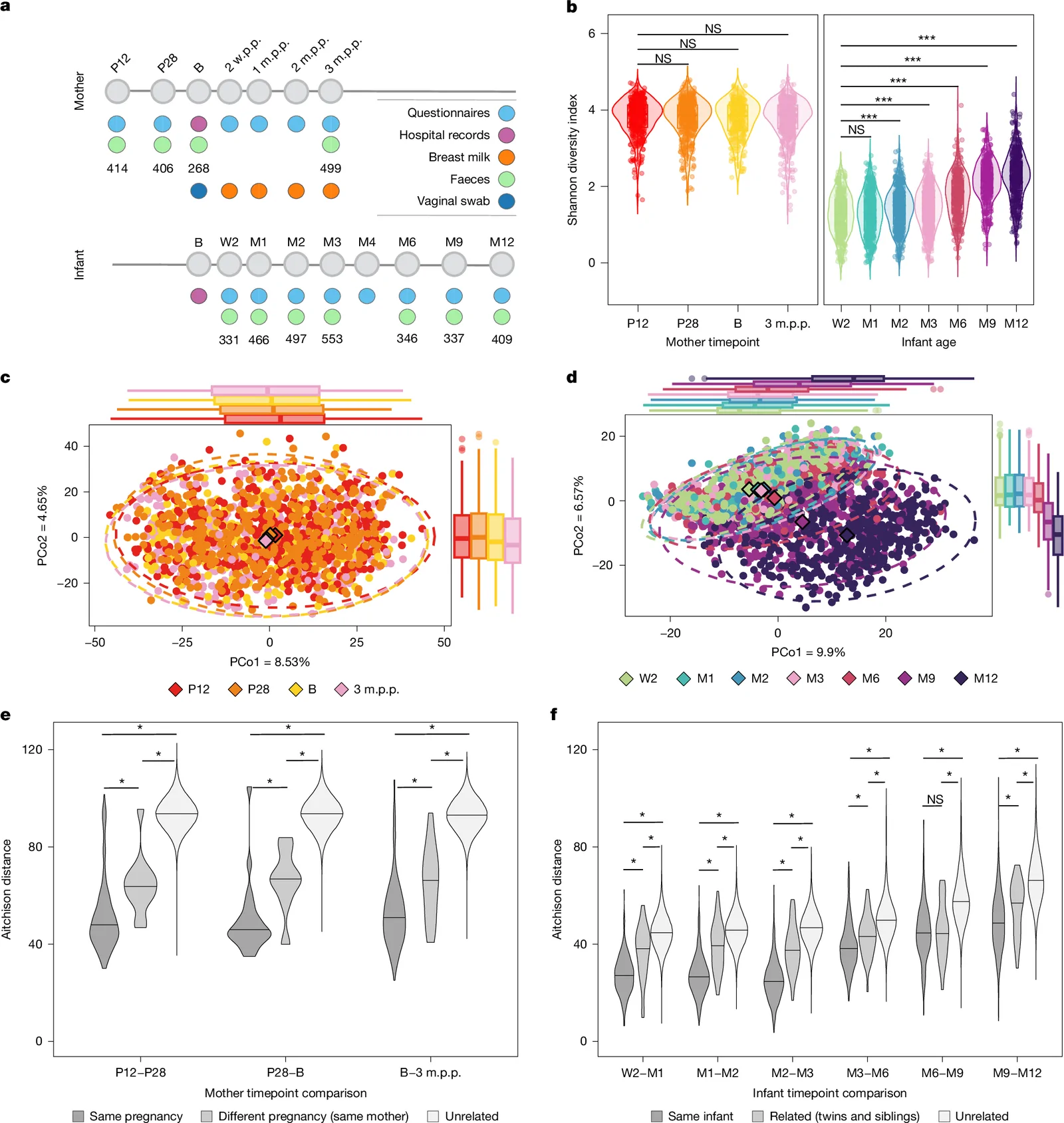
Cohort overview and gut microbiome dynamics in mothers and infants. **a**, Timeline and availability of maternal and infant faecal samples, breast milk, vaginal swabs, questionnaires and hospital records. Maternal faecal samples (green) were collected at gestational weeks 12 (P12) and 28 (P28), at delivery/birth (B) and at 3 months postpartum (m.p.p.); infant faecal samples were collected at week 2 (W2) and months 1 (M1), 2, 3, 6, 9 and 12 of age (green). Breast milk (orange) was collected at week 2 and months 1, 2 and 3 postpartum. Vaginal swabs (dark blue) were collected at birth. Hospital records (purple) were available at birth and questionnaires (light blue) were collected at 10 timepoints from P12 to 12 months postpartum. w.p.p., weeks postpartum. **b**, Maternal and infant Shannon diversity over time. *P* values were derived from two-sided linear mixed-effects models using P12 (mothers) and 2 weeks old (infants) as references. ***FDR < 0.0005; NS, not significant (FDR > 0.05); exact *P* and FDR values are provided in in Supplementary Tables 16 and 18. **c**,**d**, Principal coordinate analysis (PCoA) of species-level Aitchison distances for mothers (**c**) (permutational analysis of variance (PERMANOVA) with 10,000 permutations (restricted to within-patient sample permutations), *R*^2^_time_ = 0.77%, *P* < 1 × 10^−4^) and infants (**d**) (PERMANOVA with 10,000 permutations (restricted to within-patient sample permutations), *R*^2^_time_ = 7.78%, *P *< 1 × 10^−4^). **e**,**f**, The distances between consecutive timepoints calculated per individual, related (12 twins and 18 siblings) and unrelated pairs for maternal (**e**) and infant (**f**) samples. *FDR < 0.05; exact *P* and FDR values are provided in Supplementary Table 20. In **b**–**d**, each point represents one sample, with sample sizes (*n*) at each timepoint indicated in **a**; the colours indicate timepoints. In **c** and **d**, centroids for each timepoint are shown as diamonds in respective colours. Coloured ellipses indicate 95% confidence regions for each timepoint, assuming a multivariate *t*-distribution of the datapoints. Insets show the distributions of PCoA1 and PCoA2 by timepoint; the box plots show the median (centre line), IQR (box limits) and 1.5 × IQR (whiskers). Source data

We longitudinally characterized the gut microbiomes of 714 mother–infant pairs included in the LLNEXT study, profiling 1,587 maternal faecal samples, 2,939 infant faecal samples and 474 clinical, dietary and exposure variables across ten timepoints (Fig. 1a and Supplementary Tables 1 and 2). For a subset of mothers, we also carried out ultra-deep sequencing (40  gigabases per sample) of the vaginal microbiome at delivery/birth (*n* = 82) and the breast milk microbiome at 1 month postpartum (*n* = 90). We also measured 24 human milk oligosaccharides (HMOs) in 586 breast-milk samples collected during the first 3 months postpartum (Fig. 1a). All cohort descriptive statistics are provided in Supplementary Tables 3–15. In brief, the average maternal age at delivery was 32 years (s.d. = 3.94; Supplementary Table 7), with 45.1% (*n* = 183) of mothers delivering their first infant (that is, parity is 0) (Supplementary Table 8). Most infants (92.7%, *n* = 662) were born full-term, with 84.8% (*n* = 585) delivered vaginally and 22.5% (*n* = 155) born at home (Supplementary Table 4). Most infants (83.8%, *n* = 430) were breastfed at birth, 49.7% (*n* = 259) were breastfed at 3 months old and 18.4% (*n* = 42) were still breastfed by 12 months old (Supplementary Tables 4 and 6). In total, 12 sets of twins and 18 mothers who gave birth to 2 infants during the study period were also included (Supplementary Table 4).

**Extended Data Fig. 1:**
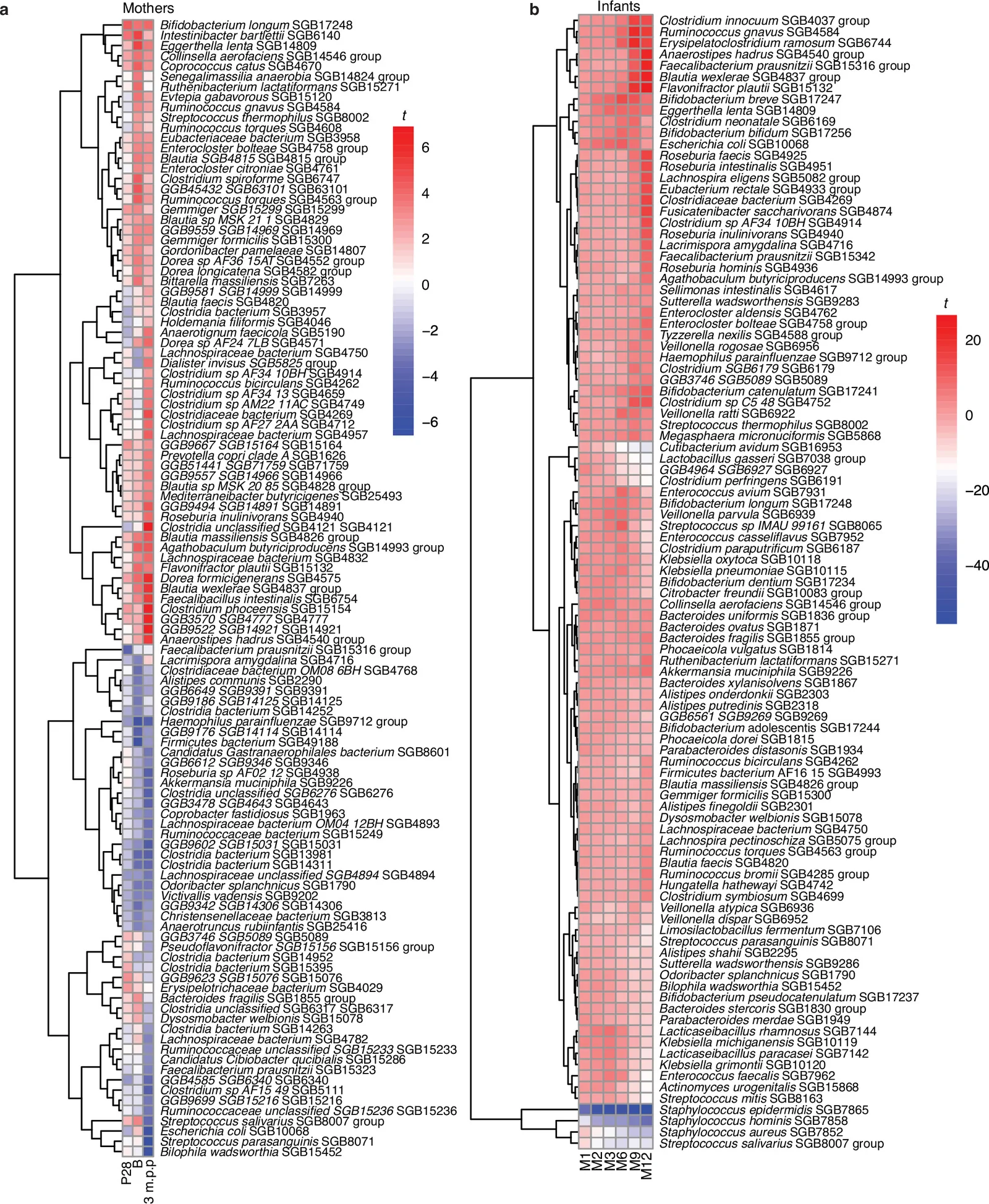
Associations of time point with maternal and infant gut microbiome. Heatmaps of the t-Values of the significant associations of time with **a**, maternal gut microbial species at species-level genomic bin (SGB) level at 28 weeks of pregnancy (P28), delivery/birth (B) and 3 months postpartum (m.p.p.) and **b**, infant gut microbial species at SGB level at months (M) 1, 2, 3, 6, 9 and 12 of age. Only significant results are shown (FDR < 0.05, linear mixed models). Pregnancy week 12 is the reference for mothers and week 2 for infants. Exact *P-* and FDR values can be found in Tables S17 and S19. Source data

We first investigated the overall dynamics of the maternal gut microbiome during pregnancy and post-delivery. Samples were collected at week 12 of pregnancy (P12), P28, at birth and at 3 months postpartum. Maternal alpha diversity remained stable over time, with no significant changes during and after pregnancy (Fig. 1b and Supplementary Table 16). We observed subtle yet significant changes in the overall maternal gut microbiome composition over time (overall *R*^2^_time_ = 0.77%, *P* < 1 × 10^−4^; Fig. 1c), with 111 species changing significantly with time (Extended Data Fig. 1a and Supplementary Table 17). For example, the abundance of *Bifidobacterium longum* (SGB17248) increased during pregnancy and, by 3 months postpartum, slightly decreased, yet remained higher at 3 months postpartum compared with at P12 (false-discovery rate (FDR) = 2.0 × 10^−4^), consistent with previous findings linking higher progesterone levels to increased *Bifidobacterium* abundance^24^.

We next examined the dynamics of the infant gut microbiome at seven timepoints: 2 weeks and 1, 2, 3, 6, 9 and 12 months of age. Infant alpha diversity significantly increased with age (Fig. 1b and Supplementary Table 18), and the overall infant gut microbiome composition was strongly associated with time (overall *R*^2^_time_ = 7.78%, *P* < 1 × 10^−4^; Fig. 1d). At 2 weeks old, the infant gut microbiome was largely dominated by *Bifidobacterium* species, *Escherichia coli* (SGB10068), skin bacteria such as *Staphylococcus epidermidis* (SGB7865) and oral bacteria such as *Streptococcus salivarius* (SGB8007 group) and *Veillonella dispar* (SGB6952). After 2 weeks old, the abundance of these skin and oral bacteria decreased significantly over time (Extended Data Fig. 1b and Supplementary Table 19), and *Bifidobacterium* species increased in the first months with the highest abundances at 6 months old (Supplementary Table 19). By 12 months old, there were marked increases in hallmark adult bacteria capable of fermenting different carbohydrates^25^, such as *Ruminococcus gnavus* (SGB4584) and *Faecalibacterium prausnitzii* (SGB15316 group and SGB15342; Extended Data Fig. 1b and Supplementary Table 19).

**Extended Data Fig. 2:**
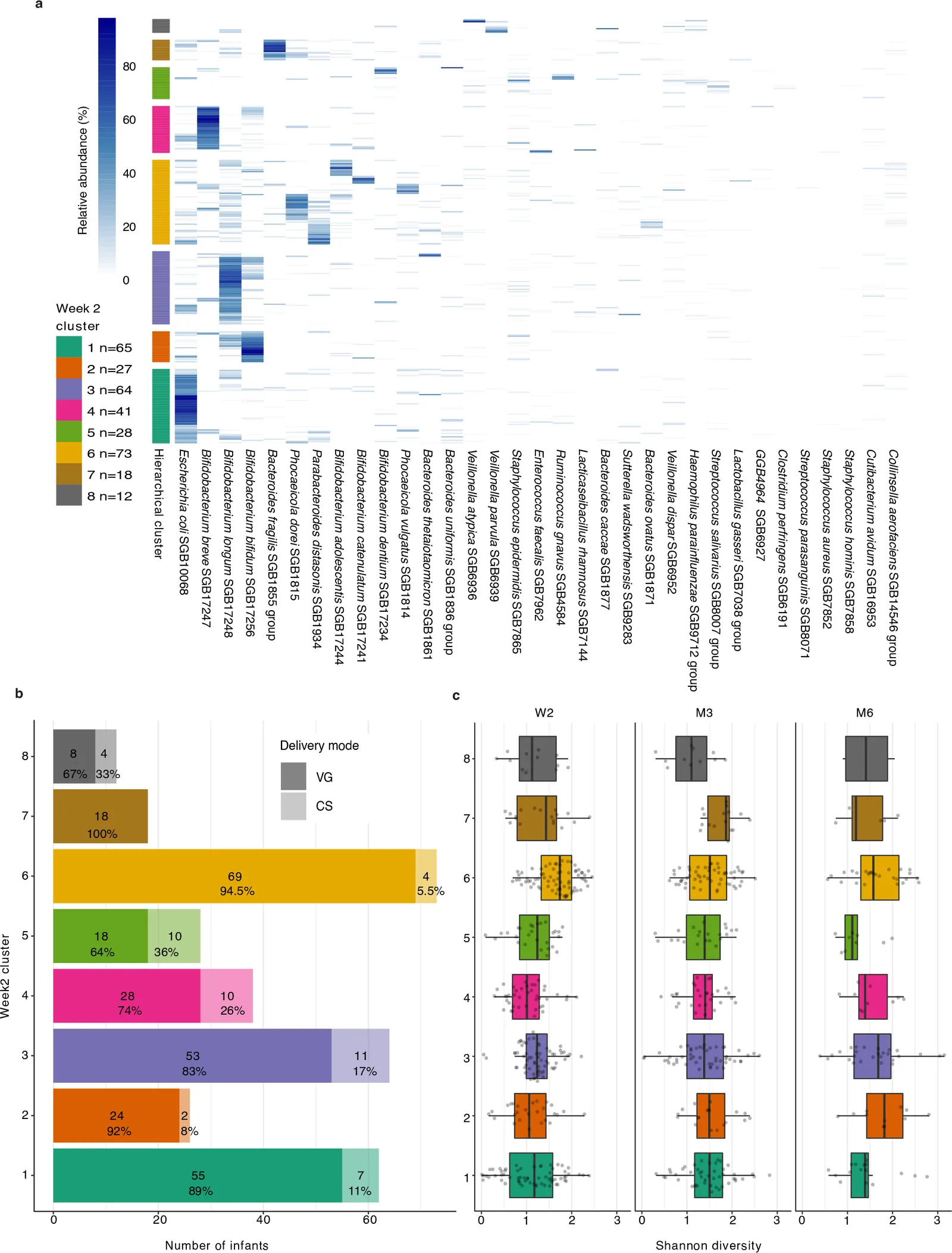
Hierarchical clustering of infant gut microbiome at 2 weeks of age. **a**, Heatmap of the relative abundance of infant gut microbial species (at species-level genomic bin (SGB) level) at week 2 (W2) with each of the eight identified hierarchical clusters defined based on Bray-Curtis distances (Methods). **b**, Proportion of vaginally delivered (VG) or caesarean section delivered (CS) infants in each of the eight identified clusters. **c**, Distribution of Shannon diversity for each cluster for the later time points (months (M) 3 and 6) of the same infants clustered at W2. Source data

**Extended Data Fig. 3:**
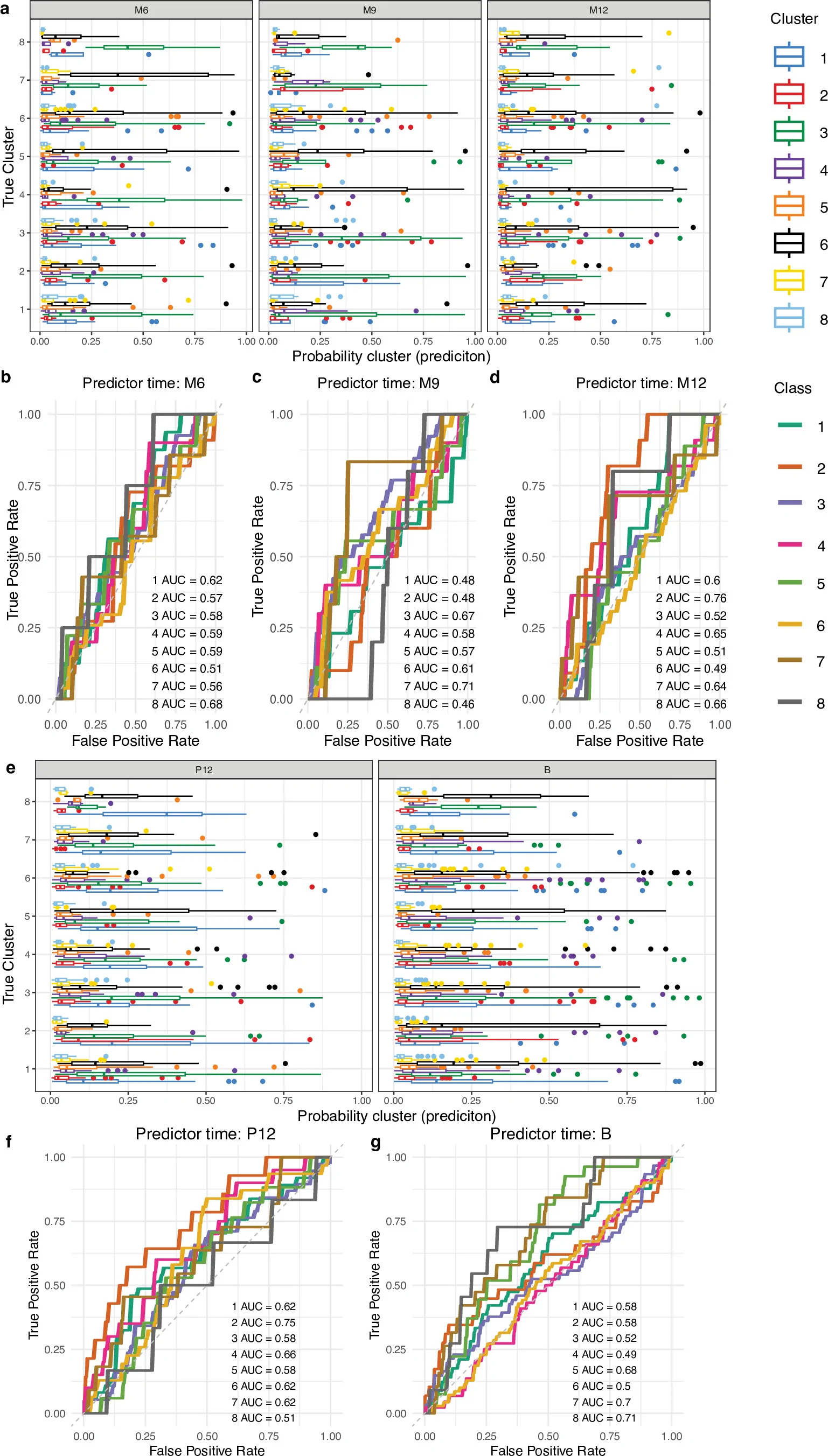
Prediction of early-life clusters using late-time points and maternal community data. **a,e**. Prediction of early time point clusters using **a**, late infant time points at months (M) 6, 9 and 12 and **e**, maternal time points at 12 weeks of pregnancy (P) and at delivery/birth (B). Plots display the prediction probability per sample and per predicted class (colour) per true cluster correspondence. High predictive probabilities do not correspond to cluster-belonging. **b,c,d**. AUC-ROC curves of the prediction of each cluster, using late infant time points and **f,g**. maternal time points.

Subsequently, we assessed whether the infant gut microbiome at 2 weeks old was determined by maternal communities before birth and whether they were deterministic of the later infant gut microbiome composition. Cluster analysis identified 8 distinct community compositions at 2 weeks old, comprising a range of 65 (cluster 1) to 12 (cluster 8) infants (Extended Data Fig. 2). Clusters 1, 2, 3, 4 and 7 were dominated by a single highly abundant taxon: *E. coli* (SGB10068), *Bifidobacterium bifidum* (SGB17256), *B. longum* (SGB17248), *Bifidobacterium breve* (SGB17247) or *Bacteroides fragilis* (SBG1855 group), respectively. Maternal communities before birth did not accurately predict clustering at 2 weeks old, nor did clustering at 2 weeks old predict later infant communities (at 6, 9 or 12 months old) (Extended Data Fig. 3). This suggests that infant gut microbiome maturation follows a non-deterministic trajectory, although this study may have been underpowered to detect such effects.

Lastly, we compared gut microbiome composition within individuals, among related individuals and among unrelated individuals. Compared with the maternal gut microbiome, infants demonstrated lower intra- and interindividual variability (Fig. 1e,f). The infant gut microbiome composition was more similar within the same individuals and among related individuals (siblings and twins) compared with unrelated individuals (Fig. 1f and Supplementary Table 20). Samples from the same mothers during pregnancy and at 3 months postpartum remained more similar to each other than samples from unrelated individuals (Fig. 1e and Supplementary Table 20). In 18 mothers from whom samples were available for both their first and second pregnancies, intraindividual variation was significantly lower than that of unrelated individuals, but higher than the distances observed within individual mothers during the same pregnancy. These patterns align with previous reports demonstrating higher microbiome similarity over time within individuals and among family members^25–27^.

## Delivery mode shapes infant gut microbiome

**Fig. 2:**
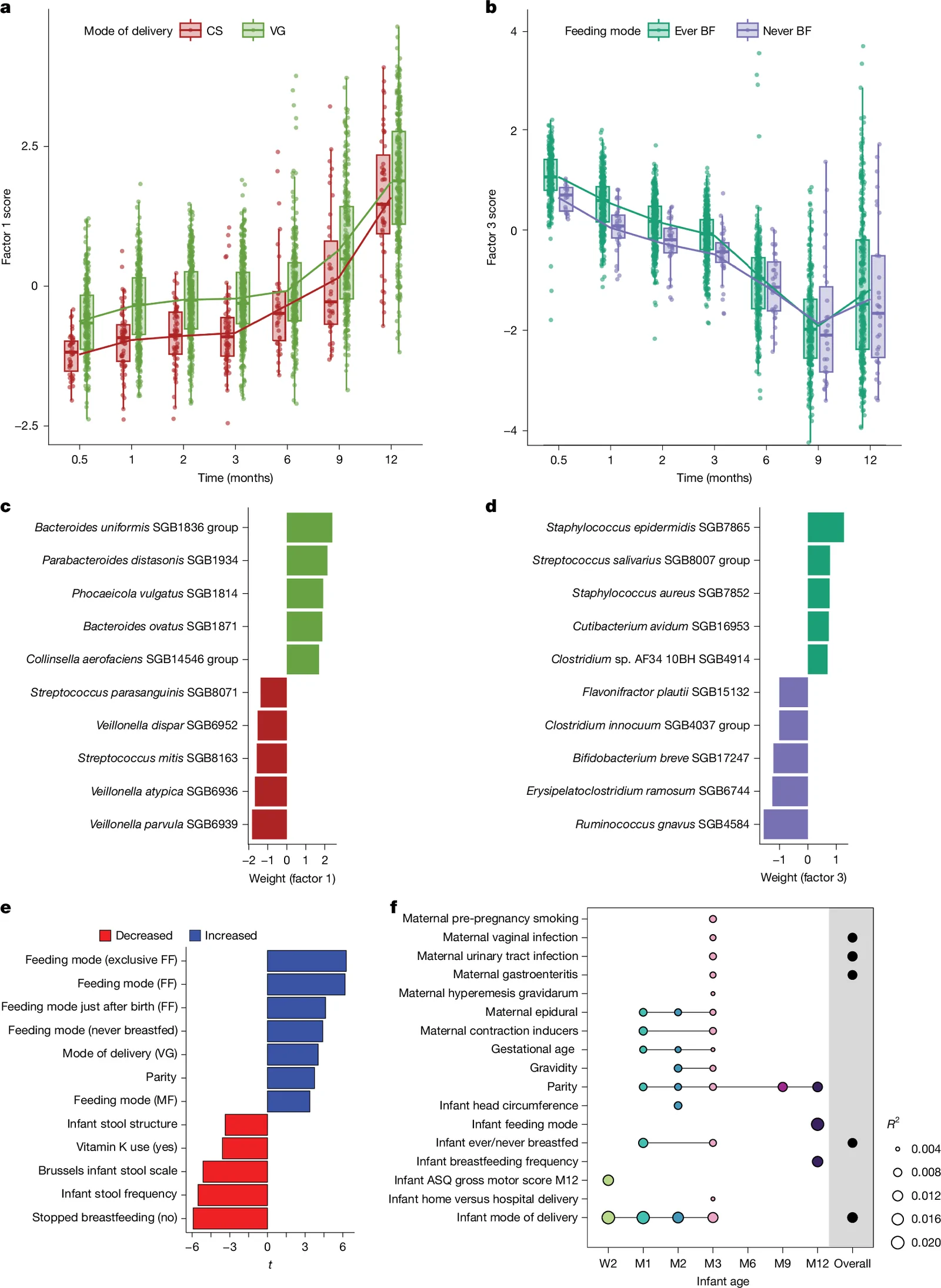
Predictors driving infant gut microbiome composition and diversity over time. **a**,**b**, The trajectories of factor 1 (**a**), associated with delivery mode, and factor 3 (**b**), associated with feeding mode (*n* = 2,428 samples), across all sampling timepoints (Methods). The dots represent inferred factor values per infant at each sampling timepoint. The lines correspond to the mean across all samples in the respective predictor category. The box plots show the median (centre line), IQR (box limits) and 1.5 × IQR (whiskers). BF, breastfeeding. **c**,**d**, The top five species with the largest negative and positive feature weights for factor 1 (**c**) and factor 3 (**d**), coloured by their association with predictor groups. **e**, *t*-Values of significant (FDR < 0.05) associations of infant alpha diversity (represented by the Shannon diversity index) with predictors. Sample sizes and exact *P* and FDR values for each predictor are shown in Supplementary Tables 23 and 24. FF, formula feeding; MF, mixed feeding. **f**, PERMANOVA of infants at seven timepoints and an overall timepoint of combined samples collected at months 1, 3, 6 and 12. The ‘overall’ analysis was based on factors derived from TCAM (Methods). All PERMANOVA analyses were performed with 10,000 permutations. Only results remaining significant (*P* < 0.001) after correction for technical covariates, delivery mode and feeding mode are shown, except when the tested variable was one of these covariates. Sample sizes and exact *P* values for each timepoint are shown in Supplementary Tables 25 and 26. ASQ, Ages and Stages Questionnaire. Source data

**Extended Data Fig. 4:**
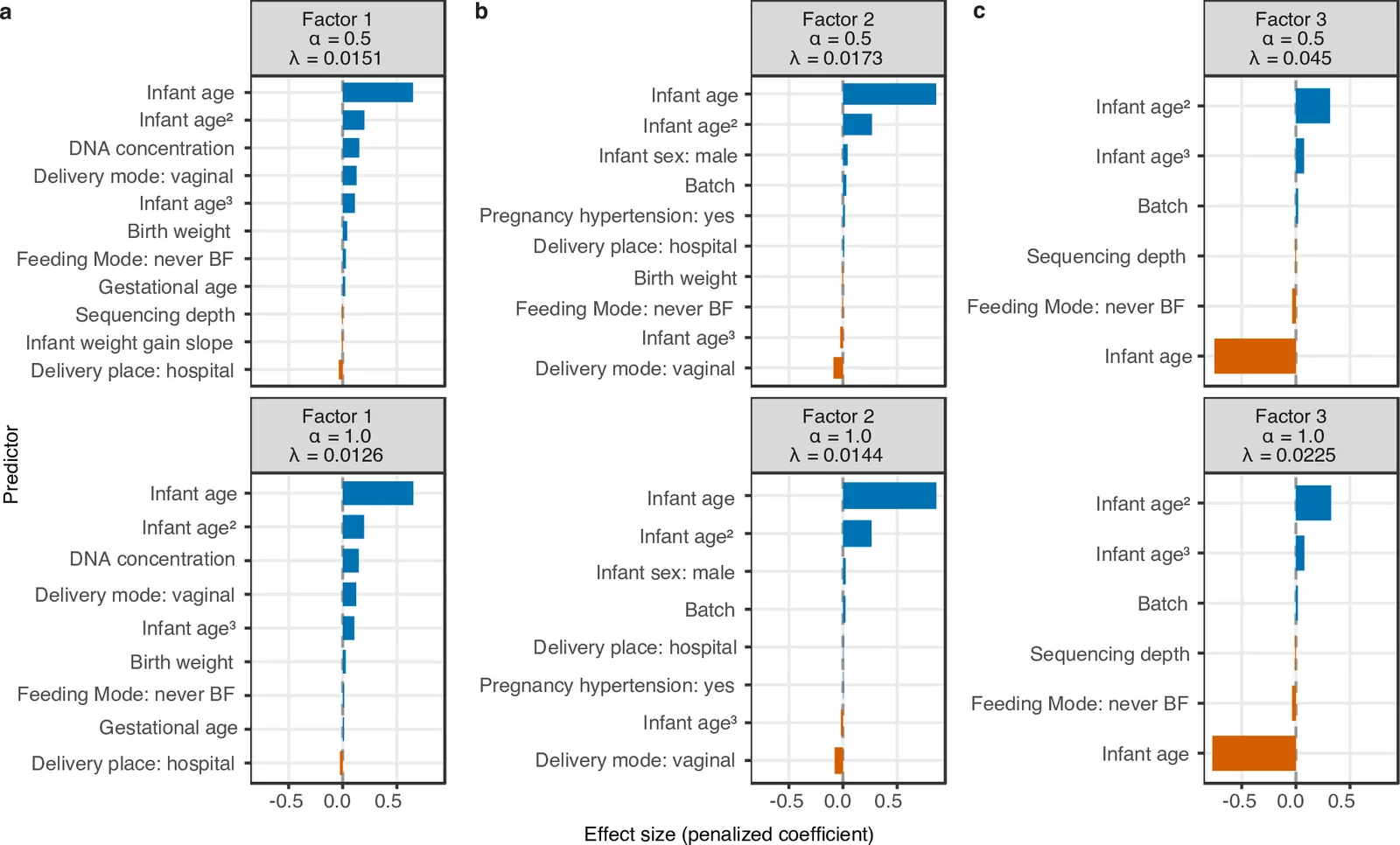
Penalized regression models of predictors with infant microbiome MEFISTO factors in the first year of life. Penalized regression coefficients for predictors (Methods) from separate models of **a**, Factor 1, α = 0.5 (top) α = 1.0 (bottom ), **b**, Factor 2 α = 0.5 (top) α = 1.0 (bottom), and **c**, Factor 3 α = 0.5 (top) and α = 1.0 (bottom). Source data

To examine the drivers of temporal variation in the infant gut microbiome, we performed a factor analysis that assesses the strength of factors (that is, latent variables) through time^28^ (Methods). The gut microbiome of infants could be divided into three time-dependent factors, collectively explaining 19.4% of the variation. We next modelled the inferred factor values using penalized mixed-effects regressions to quantify the contributions of biological predictors, temporal variables and technical covariates to each latent factor (Methods and Supplementary Table 21). Beyond time and technical effects, delivery mode emerged as the strongest biological variable associated with factors 1 and 2 (Extended Data Fig. 4). Vaginal birth was associated with positive values on factor 1 (penalized *β* = 0.13) and negative values on factor 2 (penalized *β* = −0.09), whereas caesarean section (CS) birth showed the opposite pattern (Fig. 2a and Supplementary Table 22). Beyond mode of delivery, place of delivery (home versus hospital) and birth weight were also associated with factor 1, although this effect was much smaller than that of mode of delivery (penalized *β* = −0.04 and 0.04, respectively) (Extended Data Fig. 4 and Supplementary Table 22). Factor 3 was associated with feeding mode (penalized *β* = −0.03 for never breastfed, reference = ever) (Fig. 2b and Supplementary Table 22). Its trajectory exhibited an inflection point at 9 months of age, when separation between feeding-mode groups diminished, suggesting attenuation of feeding-related effects after the introduction of solid foods. For factor 1, the species with the top three positive factor weights were *Bacteroides uniformis* (SGB1836 group), *Phocaeicola vulgatus* (SGB1814) and *Parabacteroides distasonis* (SGB1934), which were previously found to be representative of vaginal births^7^ (Fig. 2c). By contrast, factor 3 was characterized by positive weights for bacteria commonly found on the skin and in the oral cavity (Fig. 2d). Overall, these findings indicate that the development of the infant gut microbiome is primarily shaped by temporal dynamics, delivery mode and feeding mode, followed by minor effects from factors such as place of delivery, with their effects varying in magnitude and persistence over time.

We next associated 221 predictors (Supplementary Tables 3–6) with infant gut microbial diversity, adjusting for the two strongest influencing factors identified above: delivery mode and feeding mode. First, we observed that infant alpha diversity was significantly associated with delivery mode, feeding mode, parity and stool structure (Fig. 2e and Supplementary Tables 23 and 24). Vaginally delivered (VG) and formula-fed infants had a higher alpha diversity compared with CS-delivered and breastfed infants (FDR = 0.002, FDR = 0.001; Supplementary Table 23) and increased parity was associated with higher alpha diversity (FDR = 0.006; Supplementary Table 23). The variation in the overall infant gut microbiome at specific timepoints was explained by delivery-related factors, gestational age, feeding mode, parity, infant birth weight and maternal pre-pregnancy smoking history (*P* < 0.001; Fig. 2f and Supplementary Table 25). Using tensor component analysis for microbiomes (TCAM) (Methods), we examined how predictors explain the overall variation of the infant gut microbiome across months 1, 3, 6 and 12 in 208 infants with samples available at all time points^29^. Here we also observed that mode of delivery, feeding mode and maternal infections during pregnancy were significantly associated with the overall infant gut microbiome (*P* < 0.001; Supplementary Table 26).

When investigating the relationships between individual bacterial species and predictors, we identified 425 significant associations. The majority of these were related to delivery and feeding mode, and 193 associations remained after correction for these variables (Supplementary Tables 27 and 28). Associations were mostly linked to parity, stool consistency, frequency and diet. Notably, we observed very limited associations with some of the unique predictors captured through our detailed questionnaires. For example, only single associations were found for infant sleeping and no FDR-adjusted significant associations were found with crying time (Supplementary Tables 27 and 28).

We identified 280 infant gut microbial pathways associated with 51 variables, primarily feeding mode, delivery-related factors, parity, gravidity and stool frequency, largely mirroring species-level associations (Supplementary Tables 29 and 30). After adjustment for delivery and feeding mode, we found that parity, gravidity, stool frequency, infant complementary feeding (including bread, fish and meat intake) and maternal diet remained associated with numerous infant microbial pathways, with the latter highlighting the potential influence of maternal nutrition on infant microbiome development (Supplementary Table 29).

## Birth environment and infant microbiome

**Fig. 3:**
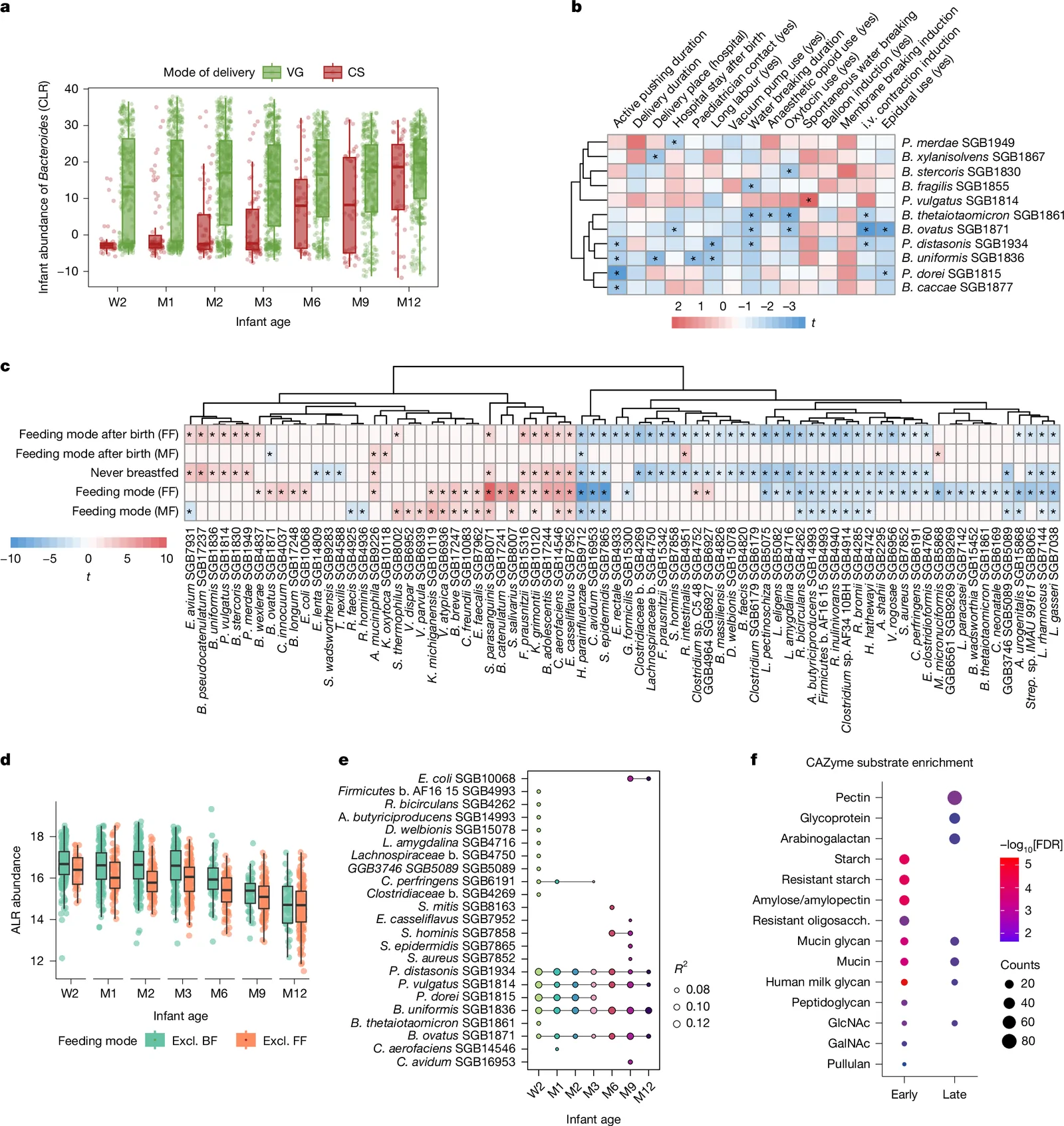
Predictors of the infant gut microbiome and CAZyme profile. **a**, The centred log ratio (CLR)-transformed relative abundance of *Bacteroides* and delivery mode across different timepoints. Sample sizes (CS, VG) at each timepoint were as follows: 51, 273 (2 weeks); 75, 376 (1 month); 74, 407 (2 months); 79, 450 (3 months); 46, 286 (6 months); 43, 281 (9 months); and 53, 334 (12 months). **b**,* t*-Values for the association between birth-related factors and *Bacteroides* species. *P < 0.05; mixed models for repeated measures (Methods); exact* P* and FDR values and full genus and species names are shown in Supplementary Table 31. i.v., intravenous. **c**, *t*-Values for associations between feeding mode at birth, feeding mode from 2 weeks to 12 months of age and ever being breastfed or not with bacterial species at the species-level genomic bin (SGB) level. *FDR < 0.05, exact *P* and FDR values and full genus and species names are shown in Supplementary Tables 27 and 28. **d**, The additive log ratio (ALR)-transformed abundance of acetate synthesis pathway with either exclusive (excl.) breastfeeding or formula feeding over time (generalized additive model FDR = 6.8 × 10^−12^). Sample sizes (exclusive breastfeeding, exclusive formula feeding) at each timepoint were: 187, 32 (2 weeks); 210, 77 (1 month); 220, 100 (2 months); 196, 141 (3 months); 72, 88 (6 months); 50, 123 (9 months); and 34, 140 (12 months). **e**, PERMANOVA with 10,000 permutations of CLR-transformed SGB abundance on the Aitchison distance matrix of the CAZyme profile of infants at seven timepoints. Only significant species (permutation *P* < 0.0001 and *R*^2^ > 0.06) are shown and exact *P* values and full genus and species names are provided in Supplementary Table 41. **f**, Over-representation analysis of CAZyme substrates at early (3 months or younger) versus late (6 months or older) timepoint of infants. Only FDR-corrected significant (<0.05) results are shown; exact *P* and FDR values are shown in Supplementary Table 47. Oligosacch., oligosaccharides. The box plots show the median (centre line), IQR (box limits) and 1.5 × IQR (whiskers). Source data

Given the large number of VG infants in our cohort (*n* = 585, 84.8%), including 155 home births (22.5%; Supplementary Table 4), we further investigated how delivery-related factors influence the infant gut microbiome. Consistent with previous findings^6–9^, infants born through CS showed a depletion of *Bacteroides* until month 3, whereas VG infants exhibited a bimodal distribution of *Bacteroides*, with the difference between CS and vaginal delivery persisting until 12 months old (Fig. 3a). We next investigated the factors influencing *Bacteroides* colonization in VG infants. We observed nominally significant reductions in *Bacteroides* abundance among infants born vaginally in a hospital compared with those born at home, specifically for *B. uniformis* (SGB1836 group; *P* = 0.002) and *Bacteroides xylanisolvens* (SGB1867; *P* = 0.006) (Fig. 3b and Supplementary Table 31). Prolonged labour, maternal use of painkillers and anaesthetics, and a longer duration of ruptured membranes were also linked to reduced abundances of several *Bacteroides* species (Fig. 3b and Supplementary Table 31), suggesting that reduced *Bacteroides* colonization reflects multiple characteristics of prolonged or complicated labour rather than a single delivery factor.

Beyond *Bacteroides*, we found that long labour was associated with increased *Veillonella parvula* (SGB6939) abundance (FDR = 0.001), and that epidural painkillers during delivery were associated with an increased abundance of *Enterococcus casseliflavus* (SGB7952) (FDR = 0.002; Supplementary Table 31). We also found nominally significant associations between a longer duration of ruptured membranes and increased abundances of *Veillonella atypica* (SGB6936) (*P* = 1.33 × 10^−4^) and *Klebsiella pneumoniae* (SGB10115) (*P* = 3.93 × 10^−4^). Lastly, hospital delivery was associated with a decreased abundance of *Sutterella wadsworthensis* (SGB9283) (*P* = 1.35 × 10^−4^).

## Infant feeding and neuroactive potential

Given the numerous associations between feeding mode and the infant gut microbiome, we further examined the individual effects of feeding immediately after birth, feeding patterns over time, and whether infants were ever breastfed on the infant gut microbiome (Supplementary Tables 27 and 28). Overall, the bacterial profiles of infants formula fed at birth closely resembled those of infants who remained formula-fed until 12 months old or were never breastfed (Fig. 3c).

**Extended Data Fig. 5:**
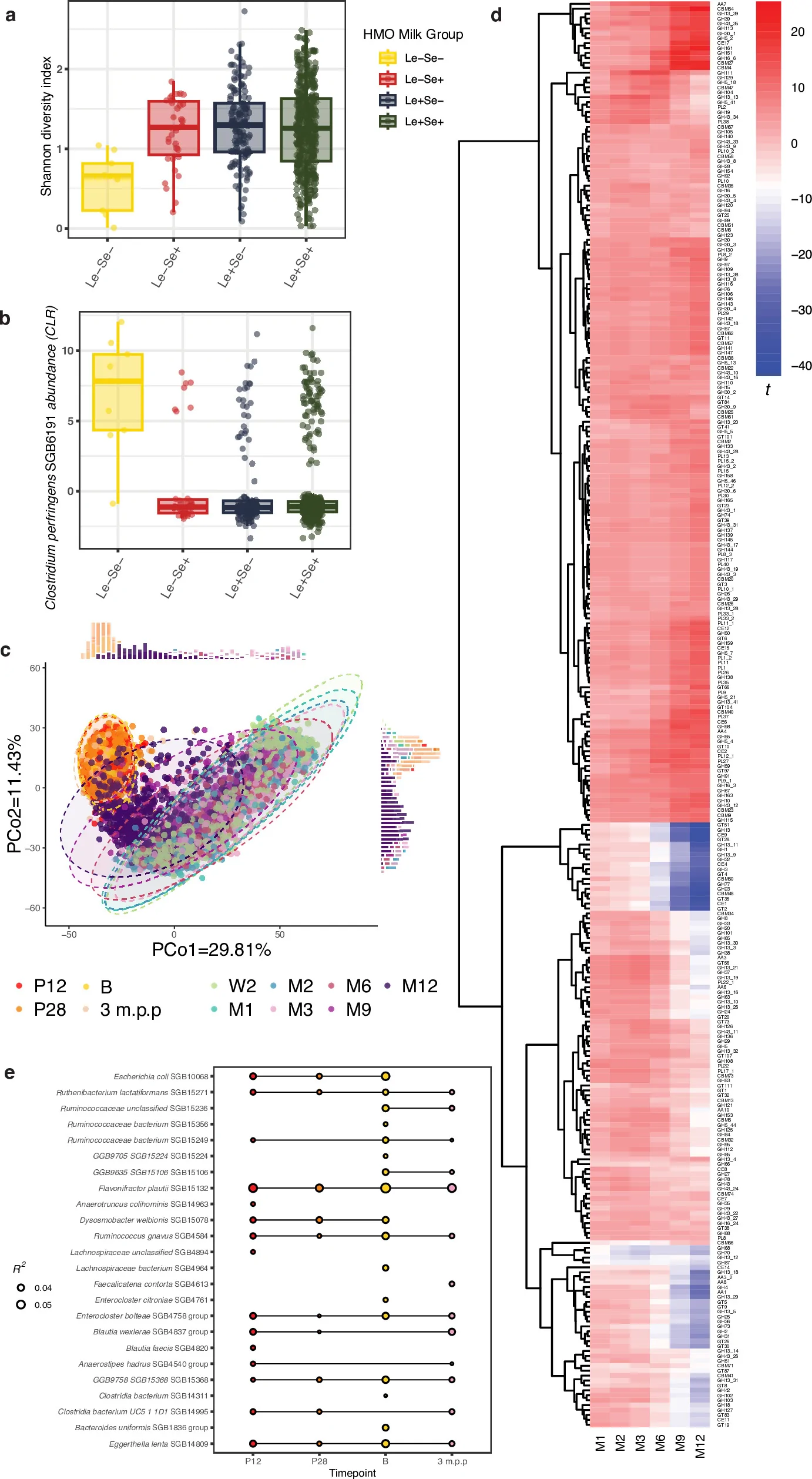
Maternal milk group and infant microbiome associations and maternal and infant CAZyme profile. Maternal secretor status and **a**, infant Shannon diversity index (*P*  values and sample sizes in Table S32), and **b**, infant relative abundance of *Clostridium perfringens* (centred log ratio (CLR)-transformed) (*P* values and sample sizes in Table S33). **c**, Principal coordinate analysis (PCoA) analysis based on Aitchison distance calculated at the sub-family level of maternal and infant CAZyme profiles at 12 weeks of pregnancy (P12), 28 weeks of pregnancy (P28), birth/delivery (B) and 3 months postpartum (m.p.p) for mothers and week 2 (W2) and months (M) 1,2,3,6,9 and 12 for infants. **d**, t-Values of the association of infant time point with CAZyme sub-families, using W2 as the reference (*P* and FDR values in Table S40). **e**, PERMANOVA with 10,000 permutations of CLR-transformed species-level genomic bin (SGB) level abundance on the Aitchison’s distance matrix of the entire CAZyme profile of mothers at various time points. Only significant species (permutation *P-*value < 0.0001 and R^2^ > 0.03) are shown and exact *P*-values can be found in Table S42. HMO: Human milk oligosaccharides. Source data

To determine whether specific components of breast milk contributed to these associations, we next investigated HMOs, which have previously been associated with the infant gut microbiome^30^. In 277 exclusively breastfed infants, we assessed associations between 24 HMOs measured in breast milk and the infant gut microbial features during the first 3 months of life. Infants of maternal non-secretors (Le−Se−) showed lower gut microbial Shannon diversity (*P* = 0.006), although this analysis was limited by its small sample size (*n* = 9 samples from three mothers; Extended Data Fig. 5a and Supplementary Table 32). In the same group (Le−Se−), the abundance of *Clostridium perfringens* (SGB6191) was nominally increased (*P* = 0.0001; Extended Data Fig. 5b and Supplementary Table 33). However, no FDR-adjusted significant associations were observed between measured HMOs and infant gut microbial diversity, species or pathways (Supplementary Tables 32–34). While some smaller studies have linked HMOs to *Bifidobacterium*^31–33^, our findings align with those showing weak or absent associations^34–36^. This may reflect limited statistical power, small HMO effect sizes, high within-feed HMO variability, the lack of data on total breast milk intake and limited variation in early HMO exposure, as 83.8% (*n* = 430) of infants were breastfed immediately after birth (Supplementary Table 4).

We next investigated whether differences in infant gut microbiome composition were also reflected in microbial neuroactive potential by examining associations between the predictors and the estimated abundance of gut–brain modules (GBMs)^37^. We identified 125 FDR-adjusted significant associations, the majority of which were related to infant feeding mode (Supplementary Tables 35 and 36). Among the most significant findings, we observed a decrease in the quinolinic acid degradation pathway in formula-fed infants, while the acetate synthesis I pathway was significantly enriched in breastfed infants (Fig. 3d). These results highlight potential differences in microbial neuroactive metabolism linked to early-life feeding practices.

Finally, we examined whether complementary foods and dietary patterns were associated with the infant gut microbiome after the introduction of solid foods (Supplementary Tables 37–39). Foods were grouped into 18 categories at 6 months old and 21 categories at 9 and 12 months old (Methods and Supplementary Table 37). Most food groups (71.4%) were associated with the microbiome (Supplementary Table 28). However, after adjusting for feeding mode and total caloric intake (kcal per day), only dairy, sweet drinks and legumes remained significantly associated with bacterial species (Supplementary Table 38). Notably, infant yogurt and quark intake were positively associated with *Streptococcus thermophilus* (SGB8002) (FDR = 8.14 × 10^−^^12^), consistent with previous links to yogurt consumption in adults^38^.

## Infant CAZymes

Given the relatively limited capacity of the infant digestive enzyme repertoire and their high reliance on microbial carbohydrate metabolism^39^, we annotated the carbohydrate-active enzyme (CAZyme) profiles of the infant and maternal gut microbiomes to investigate their dynamics and association with predictors. Similar to their gut microbiome profiles, the maternal CAZyme profile remained stable during pregnancy, while the infant CAZyme profile was highly dynamic throughout the first year of life (Extended Data Fig. 5c,d and Supplementary Table 40). In the infant gut, *Bacteroides*, *Parabacteroides* and *Phocaeicola* were the main contributors to CAZyme profile variation (Fig. 3e and Supplementary Table 41), whereas *Bacteroides* had a less-prominent role in the CAZyme profiles of the maternal gut (Extended Data Fig. 5e and Supplementary Table 42).

**Extended Data Fig. 6:**
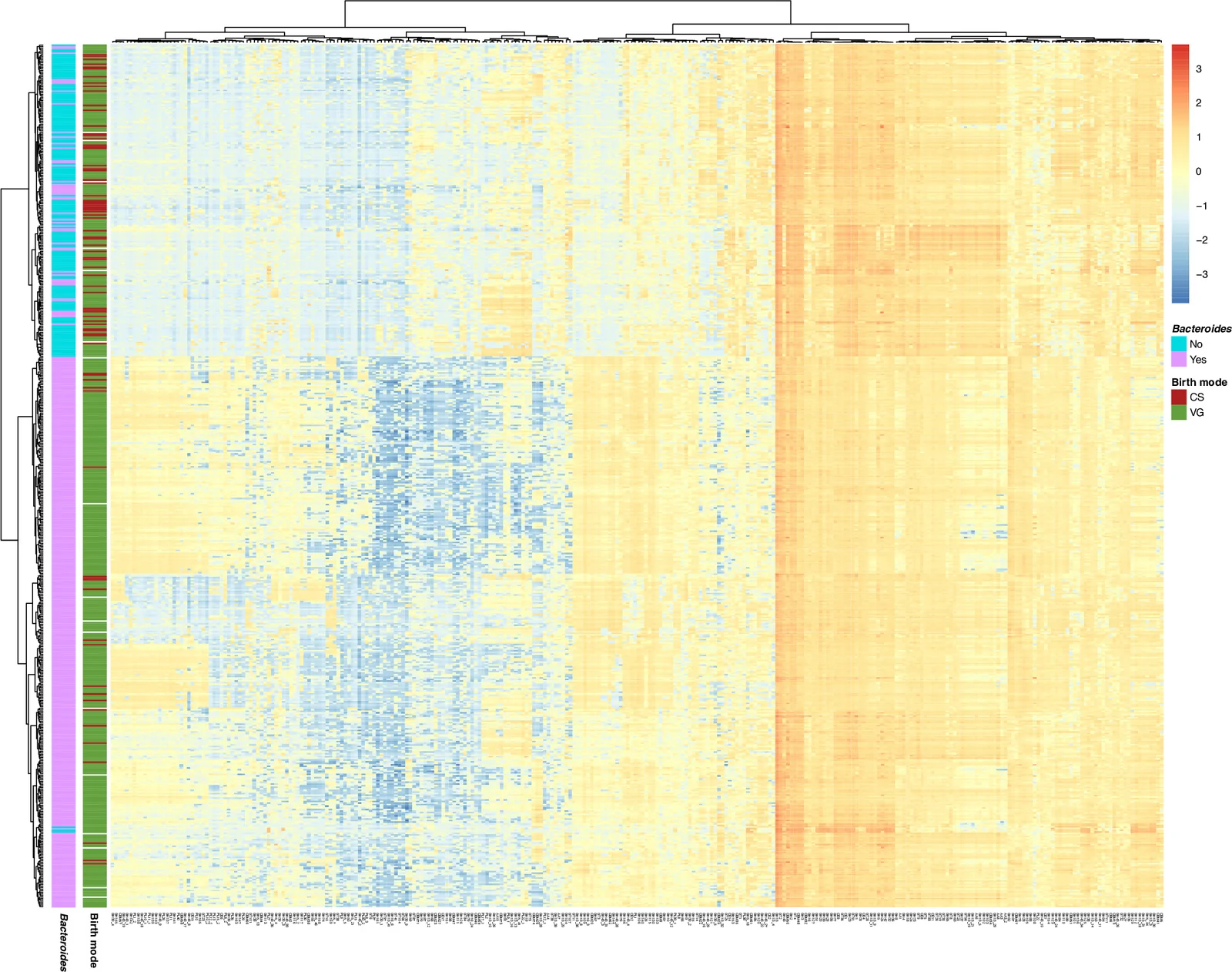
Bacteroides is the major driver of infant CAZyme profile. Heatmap of the infant additive log ratio (ALR)-transformed CAZyme abundances at month 3 (M3), with the X-axis representing CAZyme subfamilies. The Y-axis includes bar plots indicating the presence or absence of *Bacteroides* and the mode of delivery (vaginal (VG) vs. caesarean section (CS)). Source data

Most infant CAZyme associations with predictors were linked to mode of delivery, driven primarily by differences in the presence or absence of *Bacteroides*, *Parabacteroides* and *Phocaeicola* genera between the VG and CS groups (Supplementary Table 43 and Extended Data Fig. 6). We also found significant associations between infant growth indices and CAZyme subfamilies, even after correction for delivery and feeding mode. Specifically, we observed that glycoside hydrolase family 89 (GH89) was associated with infant weight, length and head circumference (FDR = 7.12 × 10^−6^, 4.95 × 10^−4^ and 4.74 × 10^−4^, respectively; Supplementary Table 44). Further annotation of CAZyme substrates revealed that infants delivered vaginally were enriched for CAZymes involved in non-starch polysaccharide metabolism, whereas those delivered by CS showed enrichment for starch-metabolizing CAZymes (over-representation analysis, FDR = 0.002 and 0.008, respectively; Supplementary Table 45). Similarly, breastfeeding was associated with an enrichment of infant CAZymes related to the metabolization of resistant oligosaccharides, mucin and human milk glycans compared with formula feeding (FDR = 0.013, 0.001, 0.000 respectively; Supplementary Table 46). Compared with the later timepoints, earlier timepoints showed a significant enrichment of CAZymes involved in the metabolism of resistant oligosaccharides and starch, peptidoglycan, amylose/amylopectin and pullulan (Fig. 3f and Supplementary Table 47). By contrast, later timepoints were significantly enriched in CAZymes associated with glycoprotein, pectin and arabinogalactan metabolism (Fig. 3f and Supplementary Table 47). This pattern reflects the rapid maturation of the infant gut microbiome’s functional capacity during infancy, highlighting the important roles of delivery and feeding mode in this process.

## The maternal microbiome predicts eczema

**Fig. 4:**
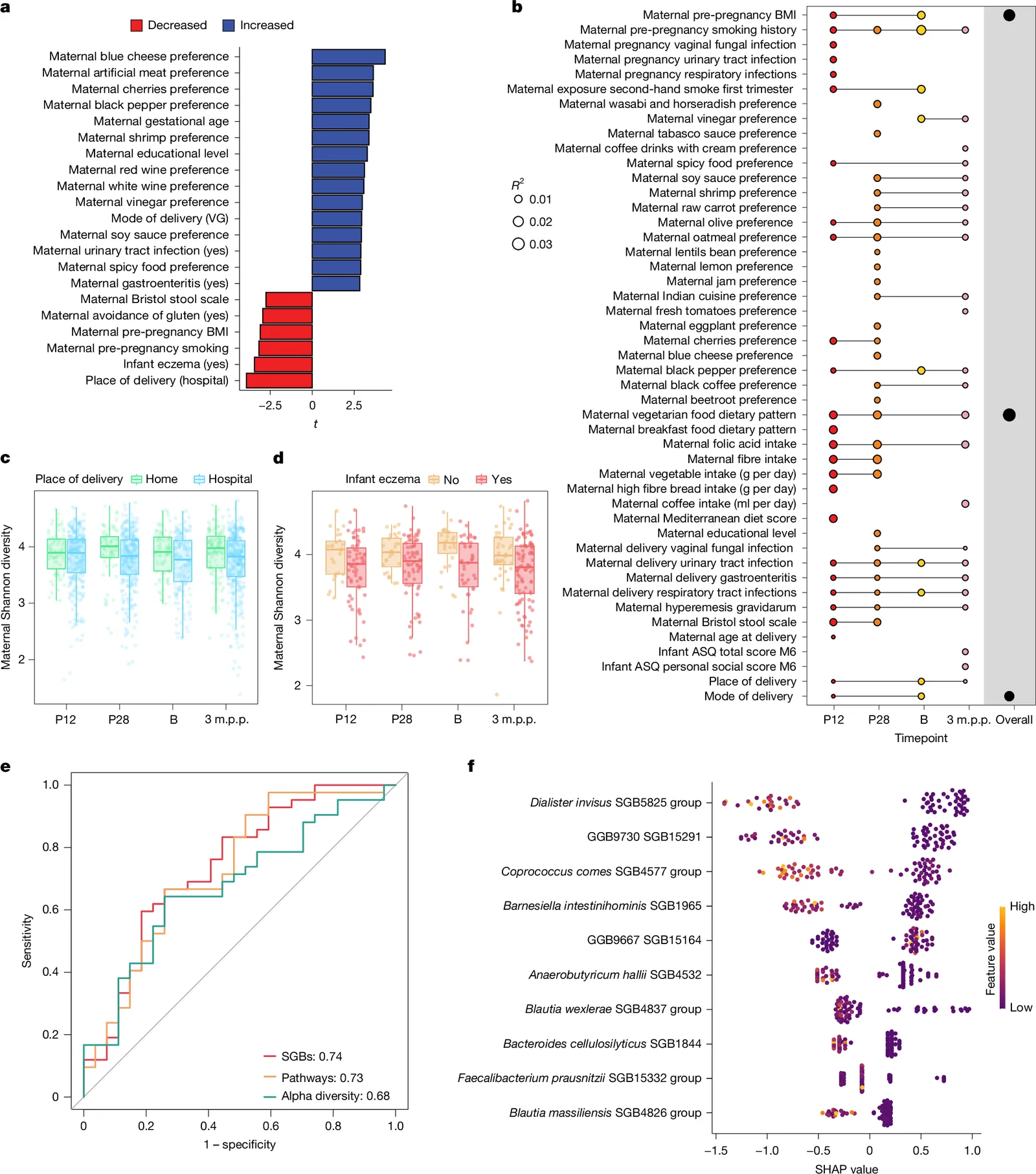
Predictors of the maternal gut microbiome and prediction of infant eczema. **a**, *t*-Values for the significant (FDR < 0.05) associations between maternal alpha diversity (Shannon diversity index) and maternal-specific predictors. Sample sizes and exact *P* values for each predictor are shown in Supplementary Tables 48 and 49. **b**, Maternal microbiome variation explained (*R*^2^) by predictors (PERMANOVA) at four timepoints (sample sizes in Fig. 1a) and an overall timepoint of combined samples (*n* = 101) collected at P12, birth/delivery and 3 months postpartum. The overall analysis was based on factors derived from TCAM (Methods). All PERMANOVA analyses were performed with 10,000 permutations and only results with *P* < 0.005 are shown. Exact *P* values for each predictor at each timepoint and overall are shown in Supplementary Tables 50 and 51. **c**,**d**, The distribution of maternal alpha diversity (Shannon diversity index) between place of delivery (**c**; FDR = 0.006) and infant eczema (**d**; FDR = 0.02) diagnosis at P12, P28, birth and 3 months postpartum. *P* values were obtained using a mixed model for repeated measures (Methods). Sample sizes (eczema status: no, yes) at each timepoint were as follows: 26, 63 (P12); 28, 70 (P28); 27, 42 (birth); and 40, 95 (3 months postpartum). Sample sizes (home, hospital birth) at each timepoint were as follows: 81, 311 (P12); 70, 318 (P28); 79, 172 (birth); and 123, 360 (3 months postpartum). The box plots show the median (centre line), IQR (box limits) and 1.5 × IQR (whiskers). **e**, Receiver operating characteristic (ROC) curves derived from an XGBoost prediction model of infant eczema using maternal bacterial SGBs, pathways or alpha diversity. Curves represent statistics from each model obtained from a leave-one-out cross-validation. The overall AUC, calculated from predictions across all models, is shown. **f**, SHAP values of the top 10 species contributing most to model predictions. Source data

We next associated 292 maternal-specific factors (Supplementary Tables 7–10) with maternal gut microbiome features. Maternal alpha diversity was significantly associated with several food preferences, delivery mode and place of delivery, gestational age, educational level, pre-pregnancy body mass index (BMI) and smoking, infant eczema, urinary tract infections, gastroenteritis and stool characteristics (Fig. 4a and Supplementary Tables 48 and 49). Notably, maternal alpha diversity was lower in women who delivered in hospitals than in those who delivered at home (FDR = 0.006), with differences already evident at P28 (Fig. 4c and Supplementary Table 48). In the Netherlands, home birth is typically limited to women with low-risk pregnancies and no previous obstetric complications^40^, who generally have better overall health. Better overall health has previously also consistently been linked to higher gut microbiome diversity^41,42^. In our study, lower alpha diversity in women was also significantly associated with several indicators of poorer health, including higher pre-pregnancy BMI and smoking exposure (Fig. 4a and Supplementary Table 48). Together, these findings suggest that differences by delivery setting probably reflect underlying maternal health and lifestyle factors.

The variation in the overall maternal gut microbiome at specific timepoints was explained by factors including maternal pre-pregnancy BMI, smoking history, urinary and respiratory tract infections, and food preferences (*P* < 0.005; Fig. 4b and Supplementary Table 50). The variation in the overall maternal gut microbiome across all timepoints (*n* = 101; Methods) was explained by maternal pre-pregnancy BMI, maternal vegetarian diet and delivery mode (*P* < 0.005; Fig. 4b and Supplementary Table 51).

When investigating bacterial species, we found 71 associations with maternal dietary features, 38 with stool characteristics, 5 with maternal age at delivery and 8 with other factors (Supplementary Tables 52 and 53). For example, maternal Mediterranean diet score (Methods) was negatively associated with *Ruthenibacterium lactatiformans* (SGB15271; FDR = 0.04; Supplementary Table 52), a species previously associated with poor cardiometabolic health^43^. When exploring associations between bacterial pathways and maternal-specific predictors, most associations were observed with dietary preferences, delivery-related factors, smoking, infections during pregnancy, maternal age and stool characteristics (Supplementary Tables 54 and 55).

Lastly, given that maternal alpha diversity was significantly lower in women whose infants developed eczema during the first year of life (FDR = 0.02; Fig. 4d and Supplementary Table 48), we further investigated whether the maternal gut microbiome could predict this infant health outcome. To assess whether this association was independent of other risk factors, we first performed a multivariable logistic regression including maternal Shannon diversity, smoking history (as it was previously linked to reduced diversity; Fig. 4a and Supplementary Table 48) and family history of allergic disease. Notably, only maternal alpha diversity remained significantly associated with infant eczema (*P* = 0.004), while the other variables did not.

We then developed predictive models to evaluate three sets of maternal gut microbiome features at birth: SGB taxonomic composition (area under the curve (AUC) = 0.74), alpha diversity (AUC = 0.68) and functional pathway profiles (AUC = 0.73) (Fig. 4e). We next identified the major microbial contributors to the taxonomic model (using Shapley additive explanations (SHAP) values; Methods). The top-ranked species was *Dialister invisus*, which was associated with a lower risk of eczema in infants (Fig. 4f). This species has previously been implicated in modulating systemic inflammation in response to a prudent maternal diet^44^.

## Mother–infant strain sharing

We next investigated maternal–infant microbial strain transmission across multiple maternal body sites. As some of the maternal body-niches have low bacterial biomass (for example, milk) or very high amounts of human DNA (for example, vaginal), we generated ultra-deep metagenomes (around 40 gigabases per sample) for 90 breast milk samples collected during the first month of life and 82 vaginal samples collected near birth, in addition to faecal samples. For each individual species, we defined strain-sharing among two metagenomes using a previously developed approach^22^. To differentiate between the same and different strains, we established genetic distance cut-offs using longitudinal samples from individuals (specific cut-offs per SGB are shown in Supplementary Table 56).

**Extended Data Fig. 7:**
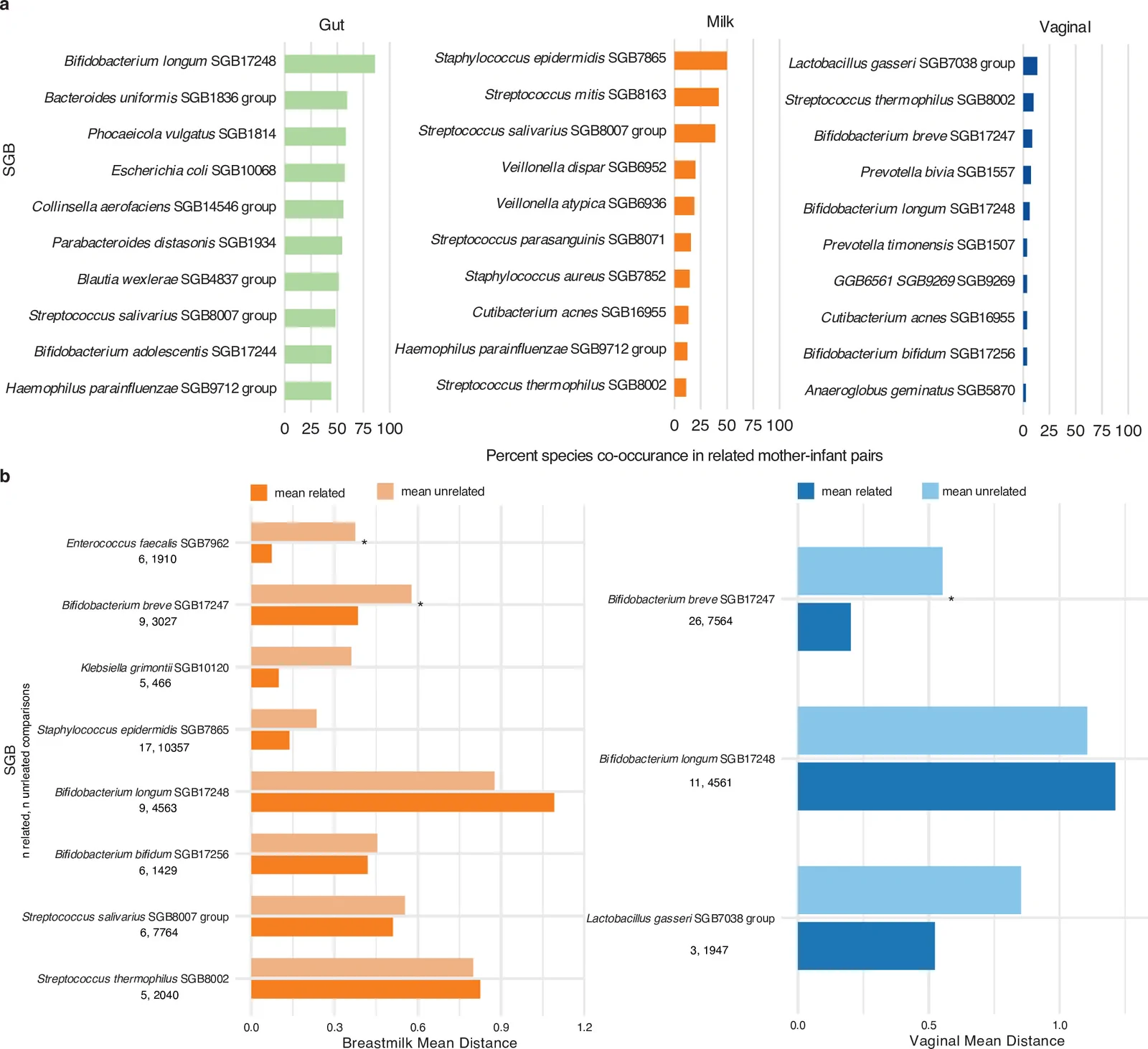
Species co-occurrence between mothers and infants and phylogenetic distances. **a**, Percentage of species co-occurrence between maternal samples (gut, breast milk or vaginal) and the infant gut in paired mother-infants. Presence was defined as a relative abundance of a species-level genomic bin (SGB) level >0.01% in both mother and infant. Sample sizes for gut, milk and vaginal pairs were 666, 90 and 82, respectively. The top 10 SGBs for each group are shown. **b**, Mean phylogenetic distances between related and unrelated mother-infant pairs between breast milk and infant gut (left in dark and light orange) and vagina and infant gut (right in dark and light blue). The number of related versus unrelated comparisons are shown in the numbers below the SGB name. *P-*values were calculated using a permutation-based test with 10,000 permutations. * *P-*value < 0.05. Source data

When analysing maternal breast milk samples, we found that *S. epidermidis* (SGB7865), *Streptococcus mitis* (SGB8163) and the *S. salivarius* group (SGB8007) were abundant and prevalent members of the breast milk microbiome (Supplementary Table 57). These species co-occurred in the infant gut (relative abundance > 0.01%) in 50.0%, 42.2% and 38.9% of the 90 mother–infant pairs, respectively (Supplementary Table 58 and Extended Data Fig. 7a). Among those co-occurring cases, strain-level sharing between breast milk and the infant gut was observed only sporadically (Supplementary Table 59), with strain sharing of maximally co-occurrent taxa *S. epidermidis* (SGB7865) detected in just two families (Supplementary Table 59). Furthermore, while phylogenetic distances between dominant strains of related mother–infant pairs were lower than those between unrelated mother–infant pairs in the majority of the cases, this was only significant for *B. breve* (SGB17247) and *Enterococcus faecalis* (SGB7962) (Extended Data Fig. 7b), with these two species showing strain transmission from the breast milk to the infant gut in two families each (Supplementary Table 59). Thus, although there is some species-level overlap, strain transmission from maternal breast milk to the infant gut appears infrequent, suggesting that breast milk is not a major reservoir for gut-colonizing strains that become dominant in infants.

**Extended Data Fig. 8:**
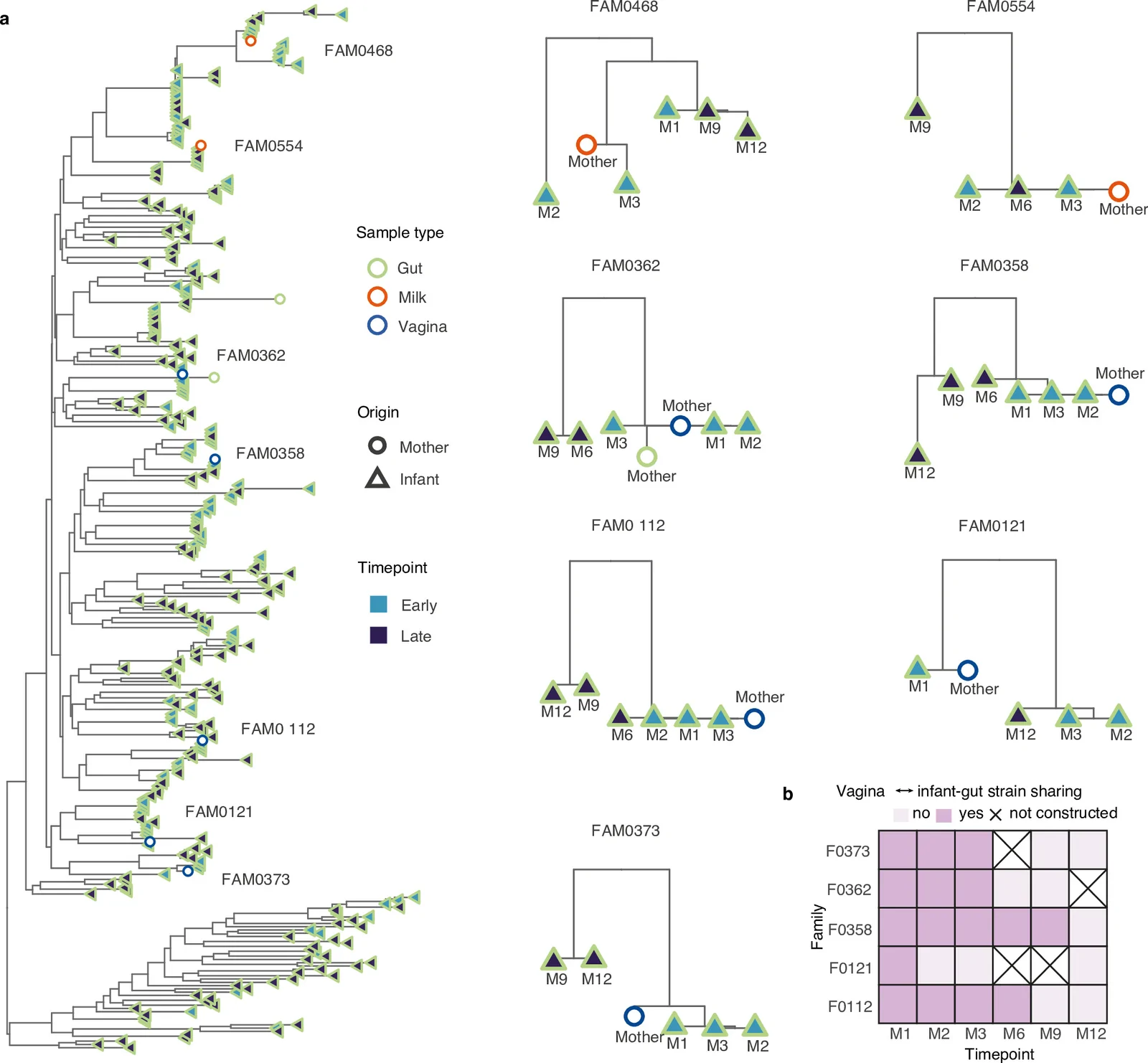
Strain-sharing between maternal breast milk, vaginal and infant gut samples. **a**, Maximum likelihood phylogenetic tree of *Bifidobacterium breve* dominant strains reconstructed from families from which a vaginal sample or breast milk sample was sequenced. Maternal samples are depicted by circles, infant samples by triangles. Outlined colours indicate sample type: green for gut, blue for vagina and orange for breast milk. Solid colours indicate time point: early (depicting months (M) 1, 2 and 3) in light blue and late (depicting M6, M9 and M12) in dark blue. Expanded insets for all five families are also shown. **b**, Heatmap depicting families in which *Bifidobacterium breve* strains were reconstructed from vaginal samples at infant birth and infant gut over time (M1 to M12). Light colour indicates no strain-sharing. Cross indicates that the strain could not be reconstructed from the infant gut at the corresponding time point. Source data

We observed even fewer species shared between the vaginal microbiome and the infant gut. The vaginal microbiome was dominated by *Lactobacillus crispatus* (SGB7045), *Lactobacillus iners* (SGB7025) and *Gardnerella vaginalis* (SGBs 17302 and 17307), all of which were rarely detected in infant gut samples, corresponding to previous literature^7^ (Supplementary Tables 58 and 60). The species *Lactobacillus gasseri* (SGB7038 group) co-occurred in 13.4% of mother–infant pairs, with strain transmission observed in a single family (Supplementary Tables 58 and 61 and Extended Data Fig. 7b). Notably, for all five mothers in whom we could construct the dominant vaginal microbiome strains of *B. breve* (SGB17247), the exact same strain was constructed in their infant’s gut (Extended Data Fig. 8a,b). We could construct the same strain in the maternal gut from only one of these five families. Four of these five infants were born by vaginal delivery, while the fifth was born by post-labour CS. Although strains of *B. breve* were also detected at later timepoints (Extended Data Fig. 8b) in all infants, these strains differed from those found earlier, strongly suggesting vertical transmission of the vaginal *B. breve* strain during birth.

**Fig. 5:**
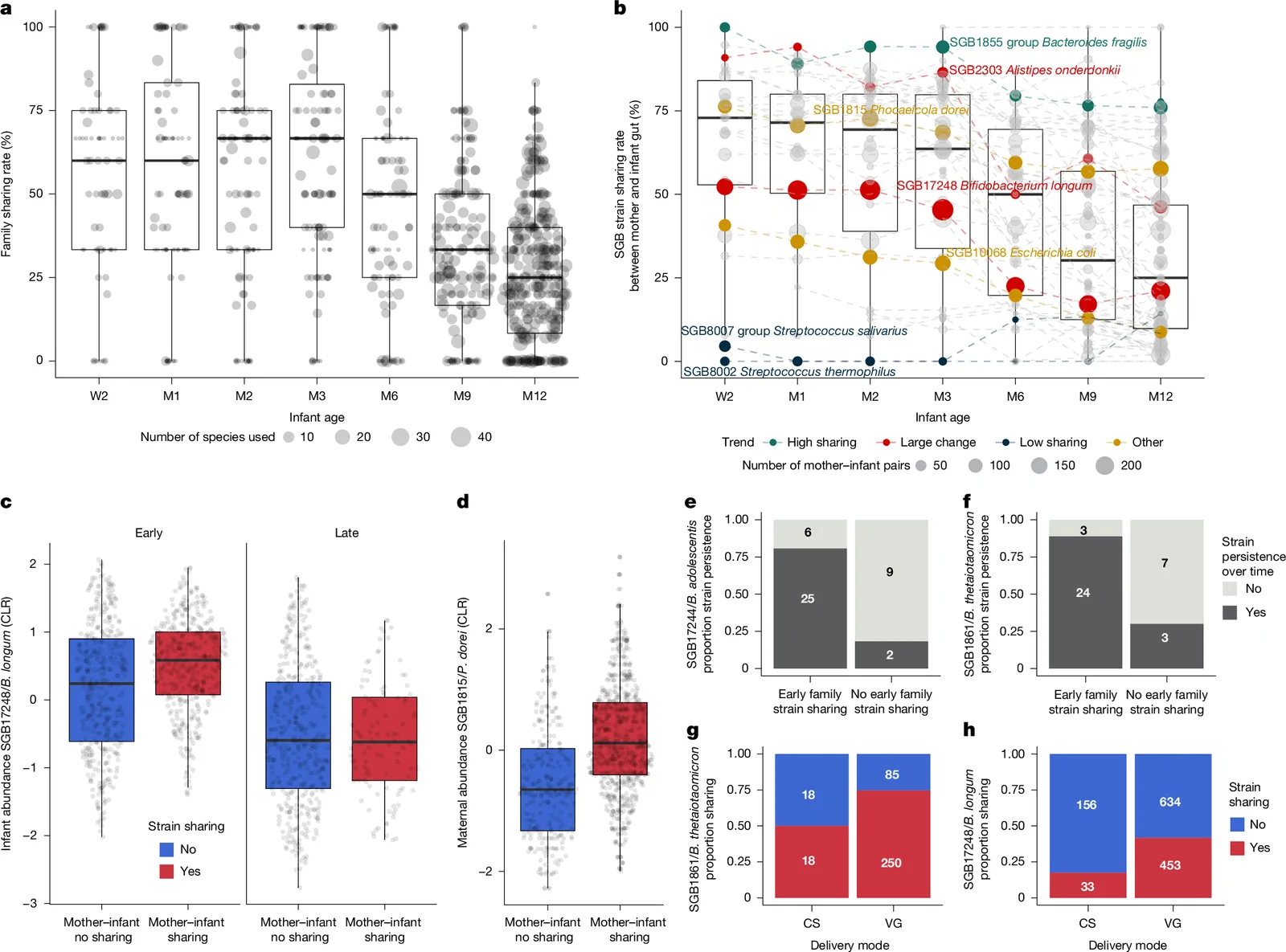
Mother-to-infant gut microbial transmission. **a**, The distribution of overall strain sharing (the percentage of same strains when at least 20 common species are present) between mothers (during pregnancy/birth) and infants over time shows a decrease in strain sharing with time (linear mixed-effect model, *P* = 6.27 × 10^−175^; Supplementary Table 62). **b**, The individual bacterial species, at the SGB level, sharing rate between mothers (during pregnancy/birth) and infants over time (Supplementary Table 63). **c**, Infant abundance of *B. longum* in relation to mother–infant strain sharing. Infants in whom *B. longum* was shared with the maternal strain tended to have a higher (CLR-transformed) *B. longum* abundance at early timepoints (up to 3 months old) than infants in whom the strain was not shared (*n* = 1,310, linear mixed-effect model, effect = 0.39, FDR = 3.26 × 10^−7^; Supplementary Table 64), but this was less so at late timepoints (*n* = 1,310, linear mixed-effect model, effect_late-timepoint × strain-sharing_ = −0.42, FDR = 0.001; Supplementary Table 64). **d**, The maternal CLR-transformed abundance of *P. dorei* is higher in mother (during pregnancy/birth) infant (up to 3 months old) pairs with the same strain (linear mixed-effect model, *n* = 1,056, effect = 1.81, FDR = 9.69 × 10^−13^; Supplementary Table 65). **e**,**f**, The proportion of infant strain persistence (same strain present at 2 weeks or 1 month and 9 months and 12 months old) of *B. adolescentis* (**e**; *n* = 42, Fisher test, FDR = 0.006; Supplementary Table 66) and *B. thetaiotaomicron* (**f**; *n* = 37, Fisher test, FDR = 0.006; Supplementary Table 66) with strain sharing. **g**,**h**, The proportion of mother (during pregnancy/birth) infant strain sharing in relation to delivery mode in *B. thetaiotaomicron* (**g**; *n* = 371, linear mixed-effect model, effect = 12.5, FDR = 0.001; Supplementary Table 68) and *B. longum* (**h**; *n* = 1,276, linear mixed-effect model, effect = 3.17, FDR = 0.01; Supplementary Table 68). The box plots show the median (centre line), IQR (box limits) and 1.5 × IQR (whiskers). In **c**, **d**, **g** and **h**, red indicates mother–infant strain sharing and blue indicates a mother–infant pair with a different strain. All *P* values and FDR-adjusted *P* values are two-sided and are provided in the respective Supplementary Tables. Source data

Given the high species overlap between maternal and infant gut microbiomes (Extended Data Fig. 7a), we next focused on gut-to-gut strain transmission over time and its association with maternal and infant predictors (Supplementary Tables 62–68). We analysed strain sharing between infant gut samples collected from 2 weeks to 12 months old and maternal gut samples collected at birth or, when unavailable, at P28. In total, for the 81 species for which we could estimate strain-sharing in more than 20 mother–infant pairs, we detected 7,345 strain-sharing events in 15,462 comparisons. All of the examined species showed at least one strain-sharing event between a mother–infant pair. We next investigated the overall sharing rate between these mother–infant pairs, which was defined as the proportion of all common mother–infant species for which both strain-sharing occurs and the strain-sharing rate changes over time (Fig. 5a and Supplementary Table 62). We found that the overall strain-sharing rate between mother–infant pairs varied from 0% to 100%, with a median of 33% of species being shared per mother–infant pair (median of unrelated mother–infant pairs was 0%). This overall sharing rate was time dependent, with higher sharing rates at early timepoints compared with at the later ones, decreasing from a median of 65% of infant species shared with mothers at 2 weeks and 1 month old, to 25% by 12 months old (*P* = 6.27 × 10^−175^, Fig. 5a and Supplementary Table 62). Such a trend was observed previously and can be attributed to a gain of new environmental strains and to horizontal transmission from new social contacts^22^.

We next explored the strain-sharing rate of individual species (Fig. 5b and Supplementary Table 63). Among the 81 species, we observed a median strain-sharing rate of 30% (combining all timepoints), which varied from 2.21% in *S. salivarius* (SGB8007 group) (136 pairs) to 100% in *Phocaeicola coprophilus* (SGB1888) (20 pairs). Six species showed a significant decrease in strain-sharing over time, including *B. longum* (SGB17248) (*β* = −0.31, FDR = 1.82 × 10^−20^) and *E. coli* (SGB10068) (*β* = −0.34, FDR = 2.33 × 10^−5^) among others (Fig. 5b and Supplementary Table 68). Conversely, some species, such as *Bacteroides clarus* (SGB1832) and *B. fragilis* (SGB1855 group), remained highly shared independent of timepoint, with overall sharing rates of 90.6% and 86.3%, respectively (Supplementary Table 63). Finally, we observed that certain species, such as *S. thermophilus* (SGB8002) and *S.* *salivarius* (SGB8007 group), were rarely shared between mother and infants, with overall sharing rates of 4.3% and 2.2%, respectively (Supplementary Table 63). We have already shown that these species are significantly associated with yogurt or quark intake (*S. thermophilus*) and formula feeding (*S.* *salivarius*), respectively, and are therefore probably gained from the diet or environment rather than vertically transmitted.

We next tested whether vertically transmitted species were more abundant in infants, consistent with priority effects, and identified 10 taxa positively associated with infant abundance levels (FDR < 0.05; Supplementary Table 64). The strongest associations were with *P. vulgatus* (SGB1814) (*β* = 0.578, FDR = 8.28 × 10^−15^) and *B. longum* (SGB17248) (*β* = 0.386, FDR = 3.26 × 10^−7^; Fig. 5c). However, this effect decreased significantly at later timepoints (ages 6–12 months compared with 2 weeks to 3 months) for *B longum* (SGB17248) (*β* = −0.419, FDR = 0.001; Fig. 5c), which may be related to strain replacement events. Notably, we also found that higher bacterial abundance in the maternal gut microbiome before birth was associated with a greater likelihood of strain-sharing in infants for 6 out of 64 species (Supplementary Table 65), including *Phocaeicola dorei* (SGB1815) (log-odds = 1.81, FDR = 9.69 × 10^−13^; Fig. 5d) and *B. uniformis* (SGB1836 group) (log-odds = 0.76, FDR = 2.36 × 10^−5^).

Given previous evidence that vertically transmitted bacteria may persist longer over time^45^, we next investigated whether early mother-to-infant strain transmission was associated with later persistence. Here we tested 16 species selected on the basis of whether we could estimate if a strain was shared between mother and infant at early timepoints (2 weeks and 1 month old) and whether it was also present at later timepoints (9 months or 12 months old; Supplementary Table 66). Six species were associated with significantly higher odds of persistence due to early transmission (Supplementary Table 66), with the top associations in over 30 samples being *Bifidobacterium adolescentis* (SGB17244) (odds ratio (OR) = 17, FDR = 0.006; Fig. 5e) and *Bacteroides thetaiotaomicron* (SGB1861) (OR = 16.5, FDR = 0.006; Fig. 5f).

We then assessed which infant-specific factors were associated with vertically transmitted species. Regarding the overall sharing rates between mother–infant pairs, we observed a positive association between breastfeeding and strain-sharing when compared to formula feeding (*β*_FF_ = −0.18, FDR = 1.74 × 10^−18^), and a trend suggesting that VG infants had more strain-sharing than CS infants (*β*_VD_ = 0.11, FDR = 0.049; Supplementary Table 67). At the species level, 5 out of 45 tested species showed associations between strain-sharing and infant factors (FDR < 0.05; Supplementary Table 68). Notably, vaginal delivery was associated with increased mother–infant strain sharing of *B. thetaiotaomicron* (SGB1861) (*β* = 12.5, FDR = 0.001; Fig. 5g) and *B.* *longum* (SGB17248) (*β* = 3.29, FDR = 0.001; Fig. 5h) compared with CS.

**Extended Data Fig. 9:**
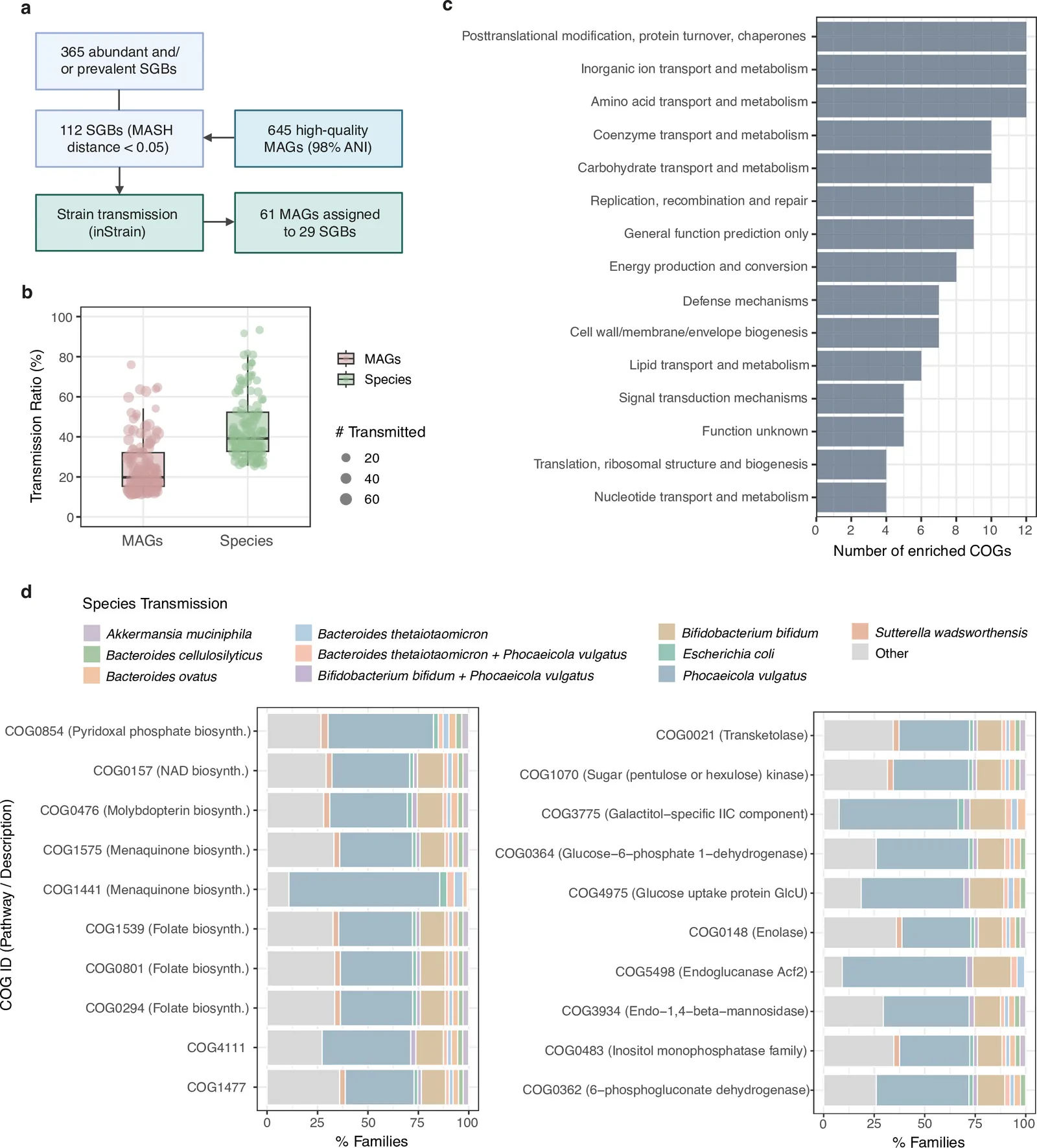
Functional enrichment of transmitted bacterial strains. **a**, Schematic overview of genome-resolved strain-sharing analysis. Starting from 365 prevalent and/or abundant bacterial species at species-level genomic bin (SGBs) level detected in mothers and infants, 645 high-quality metagenome-assembled genomes (MAGs) were identified (dereplicated at 98% average nucleotide indentity (ANI)), corresponding to 112 SGBs (MASH distance <0.05). Strain transmission was detected for 61 MAGs assigned to 29 SGBs based on high nucleotide identity (population ANI > 99.999) between maternal and infant metagenomes. **b**, Boxplots comparing transmission ratios of individual Clusters of Orthologous Genes (COGs) across MAGs (red) and species (green). Each dot represents a single COG. The Y-axis shows the transmission ratio, calculated as the number of MAGs (or species) that encode the COG and were transmitted, divided by the total number of MAGs (or species) that encode the COG. The size of each dot reflects the number of transmitted MAGs or species in which the COG was detected. **c**, Bar plot of the number of significantly enriched COGs grouped by COG functional categories (top 15 categories are shown). **d**, Species-level contributions to the transmission of enriched COGs within the Coenzyme Transport and Metabolism (left) and Carbohydrate Transport and Metabolism (right) categories. Stacked bars show the percentage of mother-infant pairs in which each species or species combination contributed to COG transmission. Top contributors are highlighted; others are grouped as “Other”. Exact sample sizes, one-sided *P-* and FDR values are found in Table S69. Source data

Lastly, we investigated whether bacterial strains transmitted from mothers to infants are enriched for specific metabolic capacities compared with the strains that were not transmitted. We identified 61 metagenome-assembled genomes (MAGs) belonging to 29 different SGBs with high nucleotide identity between maternal and infant metagenomes (population average nucleotide identity (ANI) ≥ 99.99) (Extended Data Fig. 9a). These SGBs were also shown to be transmitted in families in our previous approach using dominant strain marker genes (99.52% concordance). Functional enrichment analysis revealed 121 clusters of orthologous genes (COGs) that were significantly over-represented among the transmitted MAGs, based on their presence across a higher number of unique mother–infant pairs than expected by chance (FDR < 0.05; Supplementary Table S69). On average, enriched COGs were transmitted across 25.4% of MAGs and 44.6% of species in which they were detected (interquartile range (IQR): 15.3–32.1% for MAGs and 32.8–52.3% for species per COG), suggesting that these functions may be broadly relevant to vertical transmission across diverse bacterial taxa (Extended Data Fig. 9b). Grouping the enriched COGs into broader functional categories revealed several functions potentially relevant to the early-life gut environment, including carbohydrate transport and metabolism, coenzyme transport and metabolism (Extended Data Fig. 9c) and potentially immunomodulatory functions such as LPS acetylation^46,47^ (Supplementary Table 69). To identify the microbial contributors to the transmission of these functions, we focused on the two main functional categories (carbohydrate transport and metabolism and coenzyme transport and metabolism) and estimated the proportion of mother–infant pairs in which each species, or species combination, contributed to COG transmission. We found that *P. vulgatus* was the predominant contributor, showing functional transmission in the largest proportion of mother–infant pairs, followed by *B. bifidum* and several *Bacteroides* species (Extended Data Fig. 9d). Overall, this analysis highlights that a large proportion of strains are transmitted from the maternal gut to the infant gut, where the frequency of transmission events is positively associated with the abundance of those bacteria in the maternal gut. Strain sharing was highest at early timepoints and in VG and/or breastfed infants, with the transmitted strains enriched in functions potentially relevant to infant health.

## Infant intraspecies phylogenies

**Extended Data Fig. 10:**
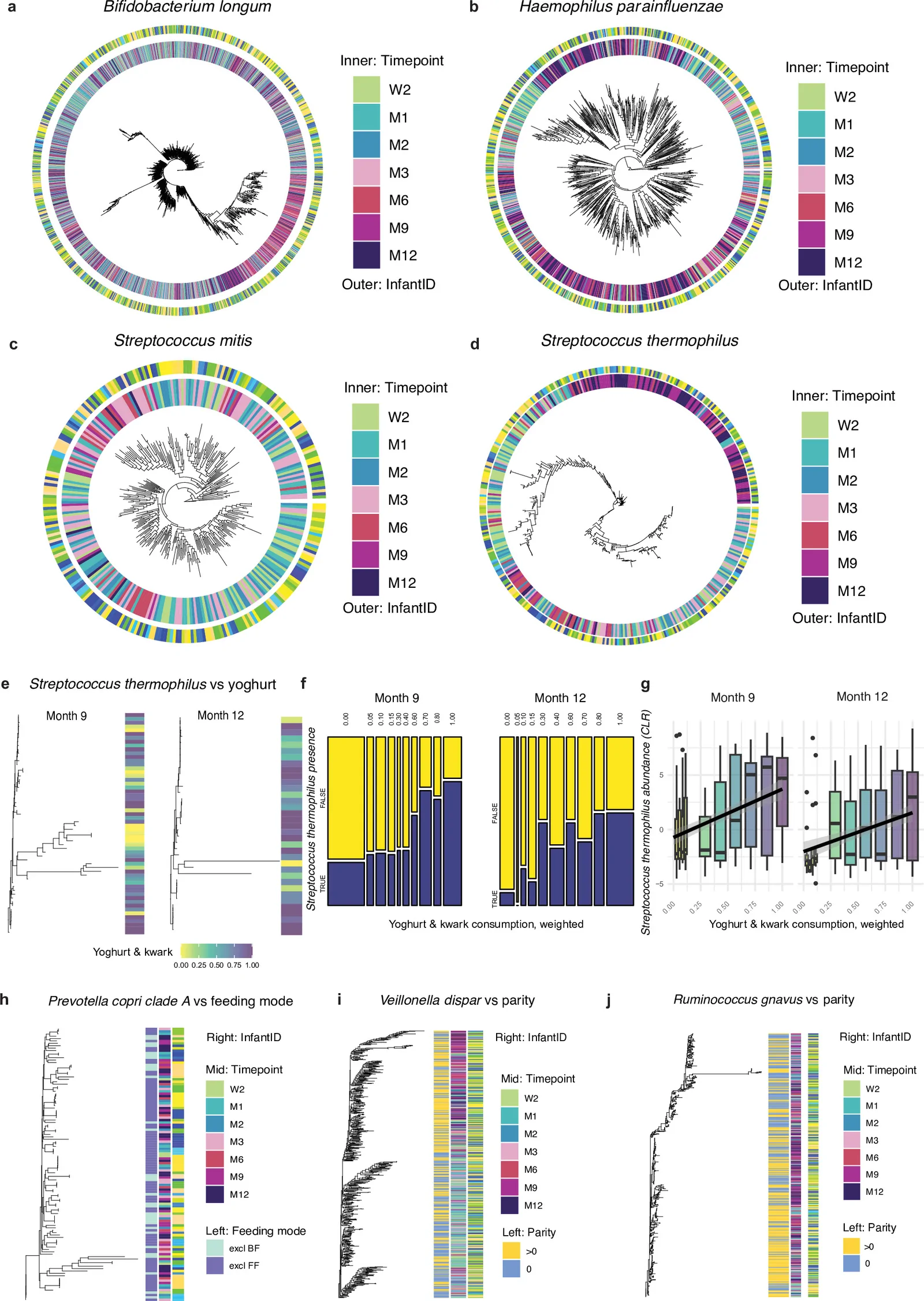
Species phylogenetic trees and their associations with time and traits. **a**, *Bifidobacterium longum* phylogenetic tree **b**, *Haemophilus parainfluenzae* phylogenetic tree **c**, *Streptococcus mitis* phylogenetic tree and **d**, *Streptococcus thermophilus* phylogenetic tree across multiple time points, including 2 weeks (W2) and months (M) 1, 2, 3, 6, 9 and 12. In a, b, c, d the inner circle represents sampling time point and the outer circle represents infant ID. **e**, *Streptococcus thermophilus* phylogenetic trees at M9 and M12. Colour bar represents weighted frequency of yogurt and quark intake. **f**, Mosaic plot of the frequency of yogurt and quark intake at M9 and M12 in relation to presence of *Streptococcus thermophilus*. **g**, Frequency of consumption of yogurt and quark at M9 and M12 in relation to centered log ratio (CLR)-transformed abundance of *Streptococcus thermophilus*. **h**, Phylogenetic tree of *Prevotella copri clade A* in relation to feeding mode (exclusive breastfeeding (excl. BF) and exclusive formula feeding (excl. FF). Left bar represents feeding mode, middle bar represents sampling time point, and right bar represent infant ID. **i**, Phylogenetic tree of *Veillonella dispar* in relation to parity. **j**, Phylogenetic tree of *Ruminococcus gnavus* in relation to parity. In i, j left bar represents parity, middle bar represents sampling time point and right bar represent infant ID. Exact sample sizes, *P-* and FDR values are provided in Tables S70, S71. Source data

Subspecies genetic variation has previously been linked to health outcomes in adults^48–50^ but little is known about its role in infants. We therefore assessed infant intraspecies phylogenetic variation in relation to infant age and other predictors (Methods). The phylogenetic variation in 19 species (FDR < 0.05), including multiple *Bifidobacterium*, *Veillonella* and *Streptococcus* species, was significantly associated with infant age (Supplementary Table 70). Several species phylogenies, including *S. thermophilus*, showed clear separation between early-colonizing (age 2 weeks to 3 months) and late-colonizing (age 6–12 months) strains (Extended Data Fig. 10a–d). Notably, the phylogeny of *S. thermophilus* was largely driven by yogurt introduction at later timepoints (9 months and 12 months old), with yogurt-consuming infants acquiring strains from a narrow clade (Extended Data Fig. 10e). This phylogenetic specificity aligns with the previously observed associations between *S. thermophilus* abundance and prevalence, and the frequency of yogurt and quark intake at 9 months and 12 months old (Extended Data Fig. 10f–g).

We next examined the relationships between other predictors and species phylogenies, adjusting for sampling time. Among 6,831 associations tested, 18 were statistically significant (FDR < 0.05; Methods and Supplementary Table 71). Multiple species’ phylogenies were associated with infant diet, including one species associated to feeding mode: *Prevotella copri* clade A (SGB1626) (Extended Data Fig. 10h). Phylogenetic variation of two highly prevalent species, *V. dispar* (SGB6952) and *R. gnavus* (SGB4584), were associated with parity (Extended Data Fig. 10i–j). Together, these findings suggest that feeding mode, diet and parity shape the infant gut microbiome not only at the species level, but also at strain level.

## Conclusions

In this high-resolution longitudinal metagenomic study, we analysed the gut microbiomes of 714 mother–infant pairs from pregnancy through the infant’s first year of life. By leveraging extensive data on pre-, peri- and postnatal exposures, including maternal, environmental, dietary and health-related factors, we validated previously reported associations and identified predictors of both maternal and infant gut microbiome composition and function, and related health outcomes.

Most infant gut microbiome studies begin at birth, often overlooking important maternal predictors, especially the maternal gut microbiome during pregnancy. In our study, the maternal gut microbiome remained largely stable during pregnancy, with moderate variations in specific bacteria, including *Bifidobacteria* species. However, the changes in the gut microbiome during pregnancy were less pronounced than previously reported^51^. This may reflect the characteristics of our relatively healthy cohort, which may not capture the microbiome alterations seen in more diverse or high-risk pregnancies, such as those involving excessive weight gain or high rates of pre-eclampsia. Yet, despite this, one finding of our study is that the maternal gut microbiome predicts infant eczema. While the underlying mechanism remains unclear, previous research suggests that the maternal gut microbiome may influence the developing fetal immune system through microbial metabolites during pregnancy or direct transfer of antibodies or cytokines^52–54^. As this is the first report of such an association, replication in diverse populations and further exploration of causal pathways are needed. If confirmed, this could open avenues for preventive interventions targeting the maternal gut microbiome.

Our analysis further highlighted the critical role of the maternal gut microbiome in seeding and shaping the infant gut microbiome. We found that a substantial proportion of infant gut strains were identical to maternal gut strains, emphasizing the maternal gut, rather than the vaginal or breast milk microbiomes, as a major reservoir for infant colonization. However, when a species typically associated with the gut microbiome was detected in either breast milk or the vaginal microbiome, it was highly likely to be transmitted to the infant. A notable example is *B. breve*, for which the specific strains were transmitted from the mother’s vagina to the infant gut in all families in which *B. breve* was present in the vaginal microbiome. We also observed that delivery mode and feeding practices were significantly associated with the rate of mother–infant gut strain-sharing, whereas place of delivery was not. Moreover, a higher maternal microbial abundance increased the likelihood of transmission, and maternally shared strains were more likely to persist throughout infancy, consistent with previous findings of smaller studies^45^. Notably, transmitted strains were enriched in genes related to carbohydrate metabolism, coenzyme transport and metabolism and immunomodulatory potential, suggesting functional advantages that may support successful colonization and long-term persistence in the infant gut.

Another gap in infant gut microbiome studies is whether the infant gut microbiome follows a deterministic maturation path. We show that, at 2 weeks of age, the infant gut microbiome was largely dominated by a single bacterial species. However, the microbiome composition at this early age did not predict the composition at a later age (up to 12 months), suggesting that the very early-life colonizers are not deterministic of later microbial communities. Throughout the first year, the primary factors shaping the infant gut microbiome and its carbohydrate-metabolizing and neuroactive potential were delivery mode and feeding mode. Consistent with previous research^7,18^, we observed that VG infants exhibited a bimodal colonization of *Bacteroides* in their gut. In our cohort, features of complicated delivery, such as longer duration of pushing and membrane rupture, and hospital birth, were moderately associated with reduced *Bacteroides* abundance in VG infants. Given the large numbers of VG infants in our cohort (*n* = 585), and the unique availability of information on 155 home deliveries, which is a common practice in the Netherlands compared with other European countries^55^, we showed that place of delivery (home versus hospital) has only small effects on the development of the infant gut microbiome. Associations were also observed with infant stool characteristics, parity (as a proxy for siblings) and diet, affecting not only species composition but also strain-level phylogenetic diversity. Notably, we also identified numerous associations with maternal pre-pregnancy smoking, pregnancy infections and diet, further supporting the importance of maternal exposures in shaping the infant gut microbiome.

Overall, we provide a comprehensive overview of microbiome dynamics during pregnancy and infancy, highlighting the central role of the mother and her microbiome in shaping the infant gut ecosystem and influencing early health outcomes.

## Methods

### Study design

The samples for this study were obtained from the Lifelines NEXT (LLNEXT) cohort, a birth cohort designed to study the effects of intrinsic and extrinsic predictors of health and disease in a four-generation design^23^. LLNEXT is embedded within the Lifelines (LL) cohort study, a prospective three-generation population-based cohort study recording the health and health-related aspects of 167,729 individuals living in the northern Netherlands^56,57^. From 2016 to 2023, 1,452 pregnant women were recruited for LLNEXT, along with their partners and infants, and monitored up to at least 1 year after birth. Several biomaterials, including faeces, breast milk and vaginal swabs, were collected from participants. Data on medical, social, lifestyle and environmental factors were collected through questionnaires at 14 different timepoints and through connected devices. The LLNEXT study was approved by the Ethics Committee of the University Medical Center Groningen (UMCG), document number METC UMCG METc2015/600. Written informed consent forms were signed by the participants or their parents/legal guardians. The present study included the first 714 mother–infant pairs recruited between 2016 and 2019.

### Predictors

Extensive predictor data were collected for all participants through questionnaires and medical records from the LLNEXT and LL cohort studies (if the parents were part of LL). Information from several sources were combined to ensure completeness. Data regarding predictors included information from mothers from before pregnancy, during pregnancy, at delivery and after pregnancy. Information from infants were obtained during birth and over the first year after birth. Predictors were investigated both cross-sectionally (for example, static predictors such as delivery mode; *n* = 316 predictors; Supplementary Table 1) and longitudinally (for example, dynamic predictors such as feeding mode; *n* = 158 predictors; Supplementary Table 2).

#### Pre-pregnancy predictors

Maternal pre-pregnancy BMI (kg m^−2^) data were obtained from the LL cohort study and complemented with medical records of LLNEXT to obtain BMI measurements for mothers up to 8 years before conception.

Maternal pre-pregnancy smoking history was classified as ‘yes’ if the mother reported smoking consistently for at least one full year at any point in her life. This period of smoking in the woman in LLNEXT occurred between the ages of 10 and 29, with a median age of 15 years.

#### Pregnancy and delivery predictors

Maternal education and income were reported at week 18 of pregnancy (P18). Mother education was categorized as follows: elementary to lower-secondary education (including incomplete and completed special primary education, primary or pre-vocational education and general secondary education), upper-secondary education (including vocational or secondary vocational education and higher general and pre-university education); or tertiary education (including higher professional education and university)^58^. Maternal income per month was derived using data from the Dutch Social and Cultural Plan^59^ and categorized as low (<€800–1,800), middle (€1,800–2,400) and high (>€2,400) income. Questionnaires related to maternal reproductive and dental health, as well as substance use were also completed at P18.

Maternal food preference and avoidance questionnaires, comprising 94 items, were completed at P12 and P32 and were developed by Wageningen University & Research (WUR). Food preference and avoidance items were evaluated using a Likert scale ranging from 0 (strong aversion) to 10 (strong preference). Given that these items were highly correlated (*r* > 0.8) at both timepoints, the questionnaires were combined, and missing data were imputed using a linear regression approach of one timepoint to another.

Delivery predictors were recorded through comprehensive medical records (birth cards completed by midwives or obstetricians at home or in the hospital) and questionnaires, which included details on delivery mode, place of delivery, duration of delivery, medication administered during pregnancy and pregnancy complications.

#### Postpartum predictors

At M2, maternal dietary intake was assessed using an online self-administered, semi-quantitative Dutch food frequency questionnaire (FFQ) comprising 177 food items (Supplementary Table 72). The FFQ was quality-checked by trained research dieticians at WUR. FFQs referred to the previous month, and mothers reported consumption frequencies from never to 7 days per week. Portion sizes were estimated in g per day using natural portions and household measures according to the ‘Measures, Weights and Codes’ booklet^60^. Daily energy intake (kcal per day) was calculated by multiplying consumption frequency by portion size and energy content from the Dutch food composition table (NEVO) of 2006 (Dutch Food Composition Database (NEVO), RIVM^61^). Mothers reporting total caloric intakes <500 kcal per day or >4,000 kcal per day were excluded (*n* = 6). Food items were grouped into 28 broader food categories (Supplementary Table 72), each named according to the most abundant food item(s) within that category. Moreover, an alternative Mediterranean diet quality score was derived^62^. To derive dietary patterns, we performed unsupervised hierarchical clustering on the food groups, measured in g per day using Euclidean distances, followed by complete-linkage clustering. Various clustering heights were tested and a dendrogram was used to identify the optimal cut height for the branches. A cut height of 26 was selected, and centroids were extracted to represent the mean consumption of all variables within a cluster (*n* = 10) for each mother. This was performed using the dendextend package (v.1.17.1)^63^ in R.

#### Infant predictors

Infant stool consistency was measured using the Brussels Infants and Toddlers Stool Scale (BITSS^64^) at 2 weeks and 1, 2, 3, 6, 9 and 12 months old. Infant stool characteristics and gastrointestinal (GI)-related symptoms were derived from the validated ROME IV questionnaire^65^ at 1, 2, 3, 6, 9 and 12 months old. The 13-item Infant Gastrointestinal Symptom Questionnaire (IGSQ) was used to assess parent-perceived GI functioning and distress, along with feeding tolerance in infants at 3 and 12 months old. Items were scored according to the user guidelines^66^, and a composite IGSQ score was derived as instructed. Scores were dichotomized at each timepoint according to the population median (as scores were right-skewed): infants with scores at or below the population median were considered to experience no-to-little GI distress and those with scores above the population median were considered to experience medium-high GI distress.

Infant feeding mode and dietary intake were obtained from medical records (that is, birth cards) and FFQs. FFQs were completed by parents or guardians and were specifically designed for Dutch infants, covering items over the previous month at 6 (49 items), 9 (66 items) and 12 (66 items) months old (Supplementary Table 37). These FFQs underwent quality checks by trained research dieticians at WUR. Feeding mode at birth (initial feeding mode infants had directly after birth), which included breastfeeding, mixed feeding (defined in this study as any ratio of breastfeeding and formula feeding (FF)) and FF, was obtained from birth cards. Feeding mode after birth was investigated longitudinally, and missing data were imputed, where possible, based on feeding mode combinations at 2 weeks to 3 months old. For example, infants with the following feeding mode combinations (2 weeks–1 month–2 months–3 months): FF–NA–FF–FF, FF–FF–NA–FF, were imputed to be formula-fed infants for all available timepoints from 2 weeks to 3 months old. Complementary feeding was derived from food items of the 6, 9 and 12 month FFQs according to the CDC definition: “foods or drinks other than breast milk or infant formula (e.g. infant cereals, fruits, vegetables, water)”. Like the mother FFQ at month 2, intake was recorded based on consumption frequencies ranging from ‘never’ to ‘7 days per week’. Portion sizes were estimated in g per day using the ‘Measures, Weights and Codes’ booklet^67^, and daily energy intake (kcal per day) was calculated using information from the Dutch food composition table (NEVO) of 2006^61^. Owing to challenges in accurately quantifying breast milk intake (in grams and energy), total daily grams and energy intake were calculated only for complementary foods, excluding breast and formula milk. Macronutrients were converted to energy percentages, where carbohydrates and protein contain 4 calories per gram, fat 9 calories per gram and fibre 2 calories per gram. Food items were grouped into 19 groups at the 6 months old and into 22 groups at 9 and 12 months old (Supplementary Table 37). Considering the dietary patterns prevalent in the Netherlands, food items related to dairy and fruit were investigated as both individual and grouped items. These items were assessed based on their consumption (yes/no) and weighted frequencies (that is, with ‘1 day per week’ being equivalent to 0.04, given that 1 month was considered to comprise 28 days). Infant dietary patterns at 6, 9 and 12 months old were derived by factor analysis (FA), using the extraction method of principal components on all standardized FFQ items (in g per day). Suitability for conducting FA was confirmed by Bartlett’s test of sphericity and the Kaiser–Mayer–Olkin test, where Kaiser–Mayer–Olkin values for all items were >0.5. To determine the number of dietary patterns to retain, eigenvalues ≥ 1.5 were considered relevant and the ‘elbow’ of the scree plot was investigated. This was followed by orthogonally rotating (using the varimax rotation method) the components. For each component, food items with loadings |>0.35| were considered to contribute significantly to the pattern. Component scores were calculated with the regression method^68^. Bartlett’s test of sphericity and the Kaiser–Mayer–Olkin test were conducted using the psych package (v.2.2.9)^69^ in R, and FA was implemented using the factanal function of the stats package (v.4.2.1)^70^ in R.

Infant anthropometric measurements, which included weight (kg), length (cm) and head circumference (cm), were obtained from medical records, growth charts and questionnaires from birth to 12 months old. Missing data were estimated, from birth to 12 months old, according to third-degree polynomial regressions of the separate weight, length and head circumference measurements. s.d. scores for all measurements were generated using the Growth Analyzer Research Calculation Tool (v4.1), using data from the Fifth Dutch Growth Study of 2008–2009 as a reference^71^.

Infant development was assessed based on the 30-item ASQ at 6 months old (ASQ-6) and 12 months old (ASQ-12). The total ASQ score (range: 0–300) and its five composite domains (range: 0–60 each) of communication, gross motor, fine motor, problem solving and personal–social skills were derived according to the ASQ-3 User’s Guide^72^. Missing data were handled according to the ASQ-3 user guide. Infant development was classified as either development_typical or development_delay based on relaxed and strict thresholds for each domain, as specified in the guide. ASQ-6 scores were derived for infants aged 5–7 months. ASQ-12 scores were derived for infants aged 11–13 months.

Infant health was derived and complemented from general infant health questionnaires (including skin irritation, eczema and diseases) at 4 months and 12 months old; food allergy questionnaires at 4, 6, 9 and 12 months old; and the International Study of Asthma and Allergies in Childhood (ISAAC) questionnaire^73^ at 12 months old. Infant atopic dermatitis (in this paper, referred to as eczema) was assessed by research nurses at 3 and 12 months old using the SCORring Atopic Dermatitis (SCORAD) approach^74^. SCORAD evaluates the area of affected skin, itching, sleeplessness and other symptoms, generating a score with a maximum of 83 that can be used to aid in diagnosing infants with eczema. In our study, infant eczema was defined in three ways: ‘infant_health_eczema_questionnaire’ (based on parent-completed questionnaires), ‘infant_health_eczema_diagnosis_strict’ and ‘infant_health_eczema_diagnosis_relaxed’. For the latter two definitions, infants were considered to have eczema if they used eczema medication between 2 weeks and 12 months old, parents reported that their infant had eczema, or the objective SCORAD score was >0. For the strict definition, infants without eczema (controls) were defined as infants who did not use eczema medication, whose parents reported that their infant did not have eczema and whose objective SCORAD scores were 0. For the relaxed definition, controls identified by the strict definition were complemented with predictors derived from the first definition (infant_health_eczema_questionnaire).

Infant crying behaviour was assessed at 2 weeks and 1, 2, 3, 6, 9 and 12 months old by an 11-item crying questionnaire that was previously validated in Dutch infants^75^.

Infant sleeping behaviour was assessed at 3, 6 and 12 months old with the 13-item brief infant sleep questionnaire (BISQ)^76^.

Infant medication use was reported by parents at 2 weeks and 1, 2, 3, 6, 9 and 12 months old and complemented with medication use reported in other LLNEXT questionnaires. The in-house-developed tool SORTA (System for Ontology-based Re-coding and Technical Annotation)^77^ was used to semi-automatically match textual medication predictors with Anatomical Therapeutic Chemical classification codes, followed by a manual curation of any unmatched medications. Infant medications at all timepoints were longitudinally investigated and updated to refer to the infant having ever received the specified medication up until the timepoint assessed.

#### Other predictors across pregnancy, delivery and/or postpartum

Data on family pets were combined from several maternal (P18, P32 and 1 month and 4 months postpartum) and infant (4 and 12 months old) questionnaires. Family living situation predictors were combined from maternal questionnaires at P32 and 4 months postpartum and included ‘house’, ‘farm’, ‘flat’ or ‘other’ types of living situations.

Maternal stool consistency was measured using the Bristol Stool Scale at P12, P28, birth and 3 months postpartum. The validated ROME III questionnaire^78^ was completed at P28 and 3 months postpartum and was used to characterize functional gastrointestinal disorders (FGIDs). Mothers were classified as having either no FGIDs or irritable bowel syndrome, functional constipation or functional bloating. Stool frequency characteristics were also derived from the ROME III questionnaire. Moreover, stool diaries were completed by mothers at P28 and 3 months postpartum to assess maternal gastrointestinal health.

Maternal medication use was self-reported at P28, birth and 3 months postpartum. Similar to infants, the in-house SORTA tool was used to semi-automatically match textual medication-related predictors with Anatomical Therapeutic Chemical codes, followed by the manual curation of unmatched medications.

The 12-item Long-term Difficulties Inventory (LDI)^79^, which assesses maternal stress in the past year, was completed by mothers at 1 and 10 months postpartum. Mothers were asked to rate stressful situations related to work, home, family and other as not stressful (score: 0), somewhat stressful (score: 1) or very stressful (score: 2). Items were summed and averaged to generate a pregnancy and post-pregnancy LDI stress score.

### Correlations between predictors

Spearman correlations were computed for all predictors (Supplementary Tables 73–76). To avoid collinearity in subsequent analyses, we excluded predictors with correlation coefficients >0.7 or <−0.7 with FDR < 0.05, including certain mother and infant diet variables, infant growth measurements and ASQ predictors.

### Sample collection

#### Faecal samples

Overall, 1,587 maternal and 2,939 infant faecal samples were collected and successfully sequenced from 714 mother–infant pairs across 10 timepoints. Mothers collected their samples during pregnancy at P12 (*n* = 414) and P28 (*n* = 406), during delivery (*n* = 268) and 3 months postpartum (*n* = 499; Supplementary Tables 12 and 13). Parents/guardians collected infant faecal samples (*n* = 2,939) at 2 weeks old (*n* = 331), and 1 (*n* = 466), 2 (*n* = 497), 3 (*n *= 553), 6 (*n* = 346), 9 (*n* = 337) and 12 (*n* = 409) months old, with an average of four samples collected per infant. The exact timeframe within which the infant samples collected in each of these categories are as follows: 2 weeks old, day 3 to day 22; 1 month old, day 23 to day 45; 2 months old, day 46 to day 77; 3 months old, day 78 to day 143 (4.69 months); 6 months old, day 148 (4.86 months) to day 232 (7.6 months); 9 months old, day 244 (8.01 months) to day 329 (10.81 months); and 12 months old, day 335 (11.01 months) to day 436 (14.32 months). Meconium was also collected from infants, but the very low microbial biomass in these samples meant our attempts to isolate viable microbial reads were unsuccessful, as previously reported^20^. These samples were therefore not included in the current study. As previously described, parents used stool collection kits provided by the UMCG and froze the faecal samples at home at −20 °C within 10 min of stool production^20^. Frozen samples were collected by UMCG personnel, transported to the UMCG in portable freezers and stored in −20 °C (short-term) or −80 °C (long-term) freezer until DNA extraction.

#### Human breast milk samples

Mothers were asked to pump breast milk with their usual breast pump equipment and without specific cleaning of the breast tissue. They were instructed to pump milk from one breast, preferably the right, from the second feeding after midnight with a time interval of at least 2 h since the last feed from that breast. Mothers homogenized milk samples by gentle shaking and, using plastic dropper pipettes (H10041, MLS), prepared 2 ml aliquots in cryotubes (122279, Greiner Bio-One). Milk samples were stored at −20 °C in home freezers, transported to the laboratory in transportable freezers, then stored at −20 °C (short-term) or −80 °C (long-term) until analysis.

#### Vaginal samples

Women were asked to collect a vaginal swab close to birth. This was placed by mothers in a Power Bead Solution (Qiagen) and stored at −20 °C (short-term) or −80 °C (long-term) until analysis.

### Microbiome processing and profiling

#### Faecal DNA extraction

Microbial DNA was isolated from 0.2–0.5 g faecal material using the QIAamp Fast DNA Stool Mini Kit (Qiagen) and the QIAcube (Qiagen), according to the manufacturer’s instructions, at the Institute for Clinical Molecular Biology, Kiel, Germany, with a final elution volume of 100 μl. DNA eluates were stored at –20 °C.

#### Human breast milk and vaginal DNA extraction

DNA was isolated from 3.5 ml breast milk and from vaginal swabs using the DNeasy PowerSoil Pro Kit (47016, Qiagen), as described previously^80,81^. In brief, breast milk samples were centrifuged at 13,000*g* for 15 min at 4 °C. Fat and whey were removed. Cell pellets were resuspended in 800 µl solution CD1, transferred to PowerBead Pro tubes and vortexed briefly. Vaginal swab samples were thawed at 4 °C for 1 h. Each sample was then vortexed for 3 min in 500 µl of PowerBead Solution. The liquid was transferred to PowerBead Pro Tubes, the total volume adjusted to 800 µl using CD1 solution and then vortexed briefly. Prepared milk and vaginal samples were incubated at 65 °C for 10 min and subsequently bead-beat at 5,000 rpm at 4 °C for 45 s on a Precellys Evolution tissue homogenizer (Bertin Instruments). The samples were then centrifuged at 15,000*g* for 1 min at 4 °C, and a 600 µl sample was used for automatic DNA extraction on Qiacubes (Qiagen) using the ‘DNeasy PowerSoil Pro Kit with Inhibitor Removal Technology Protocol’. DNA was eluted in 50 µl and stored at −20 °C.

#### Genomic library preparation and sequencing

Faecal, vaginal and breast milk microbial DNA samples were sent to Novogene, Cambridge, UK for genomic library preparation and shotgun metagenomics sequencing. Sequencing libraries were prepared using the NEBNext Ultra DNA Library Prep Kit or the NEBNext Ultra II DNA Library Prep Kit (depending on the sample DNA concentration), and sequenced using HiSeq 2000 or NovaSeq 6000 sequencing with 2 × 150 bp paired-end chemistry (Illumina), as previously described^20^.

#### Profiling of the gut, vaginal and breast milk microbiome

Bioinformatic analysis was performed in-house using our bioinformatic pipeline (https://github.com/GRONINGEN-MICROBIOME-CENTRE/gmc-mgs-pipeline). In brief, adapters were first trimmed from the gut microbiome sequencing reads with BBDuk (v.39.01)^82^ and then quality trimmed using KneadData (v.0.10.0)^83^. Thereafter, the KneadData-integrated Bowtie2 tool (v.2.4.2)^84^ was used to remove reads that aligned to the human genome (GRCh38/hg38), and the quality of the processed data was examined using the FastQC toolkit (v.0.11.9)^85^. Taxonomic composition of metagenomes was profiled using the MetaPhlAn4 tool with the MetaPhlAn database of marker genes mpa_vJan21 and the ChocoPhlAn Species level Genomic Bin (SGB) database (202103)^86^. Bacterial strain haplotypes were generated using StrainPhlAn4^86^. This method is based on reconstructing consensus sequence variants within species-specific marker genes and using them to estimate strain-level phylogenies. It considers only the dominant strain of species and therefore misses overlap in secondary strains. We profiled the abundance of microbial metabolic pathways in gut microbiomes using HUMAnN (v.3.6)^83^.

#### SGB filtration and data transformation for gut microbiome analysis

For the downstream analysis of SGBs, maternal and infant gut microbiome data were first separated. For mothers, we set a relative abundance cut-off of 0.001% and a prevalence cut-off of 30%, resulting in 322 SGBs for further analysis. For infants, we set a relative abundance cut-off of 0.1% and a prevalence cut-off of 10%, resulting in 105 SGBs for further analysis. CLR transformation was then applied at the SGB level and at higher taxonomic levels. All microbial taxa, regardless of taxonomic level, were CLR-transformed using the geometric mean of the relative abundance of microbial species as the CLR denominator. As CLR transformation cannot be applied to zero values, zeros were adjusted by adding half of the lowest non-zero value.

#### MetaCyc pathway filtration and data transformation

We filtered metabolic pathways based on a prevalence of >30% and a minimum relative abundance of 0.005% for both mothers and infant gut microbiome profiles separately, resulting in 289 pathways for infants and 171 pathways for mothers. Transformation for MetaCyc pathways was performed using ALR transformation with the geometric mean of species abundances as the denominator. As the external denominator was used, we applied additional treatment for jitter introduced to zero abundances by subtracting the geometric mean. For each pathway, all values that were zero before the transformation are made equal and placed below the smallest non-zero value.

#### GBM filtration and data transformation

GBMs comprise curated modules of microbial pathways involved in the metabolism of molecules with the potential to interact with the human nervous system^37^. We performed this analysis selectively in infants including 34 GBMs present in over 30% of infants in the analysis. Transformation for GBMs was performed using ALR transformation with the geometric mean of species abundances as denominator.

#### CAZyme filtration and data transformation

CAZymes were annotated using Cayman^87^, and a prevalence filter of 30% was applied separately to maternal and infant samples. Hand-annotated substrate specificity per CAZyme was retrieved^87^. Transformation for CAZymes was performed using ALR transformation with the geometric mean of species abundances as denominator.

### HMO profiling

The concentrations of 24 HMOs were measured in maternal breast milk samples and quantified using ultra-high-performance liquid chromatography as described previously^88^. To determine maternal Le and Se status, the milk concentrations of HMOs LNFP-II and of 2′FL, respectively were used as proxies.

### Associations between timepoint and alpha and beta diversity and species-level composition

To investigate microbial diversity within samples, the microbial alpha diversity of gut microbiome samples, as represented by the Shannon diversity index, was calculated on SGB relative abundances using the diversity function of the vegan package (v.2.7-1)^89^ in R. To test the effect of timepoint on alpha diversity and each sample, we tested this as a fixed effect in a mixed model using the lmerTest package (v.3.1-3)^90^ in R (Supplementary Tables 16 and 18). Read depth, sample DNA concentration and batch number were included as covariates and sample ID as a random effect. P12 was used as the reference for mothers and 2 weeks old as the reference for infants. To calculate the beta diversity within and between samples, we used Aitchison distances performed on filtered CLR-transformed data. The association between microbial beta diversity and time was performed using PERMANOVA (adonis2 analysis of the vegan package) with constraint to within-participant sample permutations (10,000) to derive *P* and *R*^2^. The effect of time on CLR-transformed SGB relative abundances in mothers and infants was calculated using timepoint as a fixed effect in a mixed model using again the lmerTest package with the same covariates and references (Supplementary Tables 17 and 19).

### Early-life bacterial composition clustering analysis

To define early-life clusters, 2-week-old samples were used. A prevalence cut-off of 10% on a minimum relative abundance of 0.1% was applied to focus on bacterial species at SGB level, which were prevalent in the population with medium to large relative abundances (33 out of 512 species). Using the filtered taxonomy table, we used the vegan package in R to calculate Bray–Curtis dissimilarities, which were governed by the few dominant species at 2 weeks old. Hierarchical clustering (complete-linkage) was then performed on the dissimilarity matrices. We identified the optimal number of clusters by applying a cut-off that optimized the Calinski–Harabasz index^91^. This was done using the as.clustrange function from the WeightedCluster package (v.1.6-4)^92^ in R, with a maximum number of 20 clusters.

We next assessed the community composition of the clustered samples at late timepoints (6, 9 and 12 months old) and attempted to predict the early 2-week-old clustering based on the microbial composition at these later timepoints. We built a multi-class XGBoost prediction model using R package XGBoost (v.1.7.9.1) (objective: multi:softprob, default parameters)^93^. We applied this prediction using a compositional data analysis approach^94^ by generating all possible log-ratios between bacterial species abundances with >20% prevalence in a leave-one-out cross-validation (LOO-cv) setting. We performed the same analysis using the microbial community from maternal samples at P12, at either P28 or birth and at 3 months postpartum.

Associations between clusters and predictors were performed using logistic regression, using each of the binarized cluster-belonging/-not belonging vectors as an outcome variable and each predictor as the independent variable. We ran a model per predictor and per cluster and controlled for false discovery using the Benjamini–Hochberg FDR procedure. Alpha diversity at 2 weeks, 3 months and 6 months old was associated with clusters using ANOVA, followed by a Tukey post hoc test for pairwise comparisons.

### Within and between individual distances in mothers and infants

To calculate the significance of pairwise Aitchison distances between (1) the same individuals; (2) related individuals; and (3) unrelated individuals, we used a permutation-based approach. This involved calculating the *t*-statistics between the distances of different groups and deriving *P* values from an empirical null distribution of *t*-statistics derived from 10,000 permutations of the group labels.

### Latent variable analysis assessing the temporal variation in the infant gut microbiome (MEFISTO)

To understand the drivers of temporal variation in the infant microbiome, we performed a FA that assesses the strength of factors (that is, latent variables) through time^28^ (package MOFA2 (v.1.14.0)), trained using CLR transformed data at SGB abundance level. It therefore permits a longitudinal analysis that estimates latent factors structuring the data across all timepoints and allows inclusion of samples with missing observations.

To establish a stable and appropriate number of factors for MEFISTO, we performed a resampling-based factor stability analysis. MEFISTO models were fitted across a range of factor dimensionalities, from 2 to 12 factors. For each dimensionality, 15 random subsampling splits were generated by selecting 80% of individuals from the full infant microbiome dataset, without replacement. Each subsampled dataset was used to refit the MEFISTO model, yielding estimates of factor scores (*Z*) and factor weights (*W*). For each factor dimensionality, factor stability was assessed by computing pairwise Pearson correlations across all combinations of subsampling splits. For the factor weights (*W*), correlations were calculated after matching corresponding factors across splits based on similarity of their weight vectors, as factor ordering is not identifiable. Factor directions were additionally aligned by sign-flipping to ensure consistent orientation across runs. For factor scores (*Z*), stability was assessed by first computing the mean factor score across individuals within each subsampling split. After matching and sign-aligning factors across splits, pairwise Pearson correlations were calculated for the mean factor scores across all combinations of subsampling splits at each factor number.

Together, these analyses quantify the stability of inferred factors and their associated weights as a function of the number of estimated factors. Higher correlations indicate greater robustness of the inferred latent structure, thereby informing the selection of an appropriate number of factors for downstream analyses. The three factor solution exhibited near perfect reproducibility of both *Z* and *W* across splits, indicating a highly stable latent structure. When adding four or more factors, values of *Z* and *W* exhibited lower mean correlations and substantially greater variability across subsampling splits, suggesting overparameterization and reduced reproducibility of the inferred latent structure. We therefore chose a three-factor model. These factors explained 8.27% (factor 1), 4.73% (factor 2) and 3.88% (factor 3) of total variance, respectively, with 19.4% explained by the full model. These factors were moderately correlated (*r* = 0.40–0.58), consistent with partially overlapping but non-orthogonal dimensions of microbial variation.

We next modelled the inferred factor values as outcomes in penalized mixed-effects regressions to quantify the relative contributions of biological predictors (for example, mode of delivery), temporal variables (for example, time) and technical covariates (for example, sequencing depth) to variation in each latent factor. In selecting predictors from all cross-sectional variables, we included those with a maximum of 15% missingness and excluded those for which the most frequent category accounted for more than 95% of observations. We then retained predictors with a Spearman’s correlation not exceeding 0.5. This resulted in 10 predictors, 3 time variables (the first-, second- and third-degree polynomials of time) and technical variables, such as DNA concentration, sequencing depth and batch number, resulting in 2,428 infant samples with no missing data for those variables. To ensure comparability of penalized coefficients, all predictors were standardized to mean zero and unit variance before model fitting, including continuous and dummy-coded categorical variables. To include the assessment of non-linear time in addition to linear effects of time, we used the base R function poly() to calculate the first three polynomials of time, standardize their unit sum of squares and orthogonalize their values. This results in variables of time that have a mean of zero. Thereafter, all these components of time were scaled to have a s.d. of one, thereby matching the s.d. of all other biological or technical variables.

This set of standardized predictor variables were then used in a penalized regression model that takes into account random effects at the level of the individual. For each factor, a model was fitted using the R package glmmPen (v.1.5.4.8)^95^, which uses a Monte Carlo expectation–maximization algorithm, with adaptively increasing Monte Carlo sample sizes during optimization, until convergence. The regularization parameter lambda (*λ*) was selected via the Bayesian information criterion using a penalized likelihood optimization built into glmmPen. Models were fitted using two elastic-net mixing parameters corresponding to pure LASSO (*α* = 1.0) and a partially ridge-regularized model (*α* = 0.5) to confirm that our conclusions based on variable selection were not sensitive to the regularization scheme chosen. Results mentioned in the text are for *α* = 0.5. Extended Data Fig. 4 shows results for both *α* = 0.5 and *α* = 1.0.

### Associations of infant and maternal predictors with overall gut microbiome composition

In this analysis, we investigated the effects of both cross-sectional predictors, which were static over time (for example, place of delivery), and longitudinal predictors, which varied over time (for example, stool frequency). We first filtered predictors to those present in at least 50 samples at each infant timepoint and at least 100 samples at each maternal timepoint, excluding predictors for which the most frequent category accounted for more than 95% of observations. To test the effect of predictors on the overall gut microbiome composition at each timepoint, we used the adonis2 function from the vegan package in R with 10,000 permutations, using Aitchison distances as described above and correcting for read depth, DNA concentration and batch number for the base model. For infants, this was followed by an extended model including these covariates plus mode of delivery, and a final model incorporating mode of delivery and feeding mode. In all cases, predictors were added to the model with covariates and the setting by=“margin” was used. To test the effect of predictors on the overall composition (termed overall in the main text and figures), considering the temporal patterns of the gut microbiomes of mothers and infants, we used TCAM, a dimensionality reduction method for longitudinal ’omics data analysis^29^. TCAM is an unsupervised tensor factorization method that enables the decomposition of time-series data, thereby reducing the dimensionality for microbiome studies. For maternal data, three timepoints were selected from pregnancy to three months postpartum (P12, birth and 3 months postpartum). For infants, four timepoints across the first year were selected (1, 3, 6 and 12 months old). Only samples from individuals with all specified timepoints were included in the analysis, as TCAM does not allow for missing data. Gut microbiome profiles were filtered at the SGB level to retain taxa with a relative abundance >0.01% in at least five individuals and were subsequently CLR-transformed. TCAM analysis was conducted according to the protocol described in the original publication^29^, using the mprod package (v.0.0.5a1)^96^ in Python. We calculated the Euclidean distance between TCAM components, and associations between the distance matrix and predictors were determined with 10,000 permutations, as described above. These results were then combined with the per-timepoint analysis.

### Associations of infant- and maternal-specific predictors and HMOs with the gut microbial species and pathways in mothers and infants

These relationships were investigated both cross-sectionally, where predictors (such as place of delivery) were static over time, and longitudinally, where predictors (such as stool frequency) changed over time. The effects of static predictors on alpha diversity, SGBs relative abundance and pathway abundance were tested with mixed models for repeated measures, using the mmrm package (v.0.3.12)^97^ in R. For dynamic predictors, we used generalized additive models, using the mgcv package (v.1.9-1)^98^ in R. In both cases, read depth, sample DNA concentration and batch number were included as covariates in the base model. For infants, this was followed by an extended model including these covariates plus mode of delivery, and a final model incorporating mode of delivery and feeding mode (when these were not the primary predictors of interest). Sample ID was included as a random effect in all models. Numeric variables were inverse-rank transformed before analysis. We included only predictors of interest that were present in more than 200 samples and excluded those for which the most frequent category accounted for more than 95% of observations. Associations of delivery-related predictors with the gut microbiome was performed as above, only in VG infants. To assess associations between breast-milk HMOs and the infant gut microbiome, we used generalized additive models and linked the inverse-rank-transformed HMO concentrations in breast milk at a specific timepoint with the infant gut microbiome at the same timepoint. Associations were corrected for technical variables (DNA concentration, sequencing depth and sample batch), and the analysis was restricted to breastfed individuals. Benjamini–Hochberg correction was used to control for multiple testing in all these analysis, and the number of tests equal to the number of feature–predictor pairs was tested. Results were considered significant at FDR < 0.05.

### Association of GBMs with species and predictors

Associations between variables and GBMs were tested on ALR-transformed data using the same modelling framework used for microbial taxa and adjusting for technical covariates, delivery mode and feeding mode when these were not the primary variables of interest.

### Association of CAZymes with species and predictors

To assess the influence of bacterial composition on the variation in CAZyme profiles, we tested the CLR-transformed abundances of taxa at the SGB level in mothers and infants using PERMANOVA (adonis) with 10,000 permutations against the Aitchison distance matrix calculated from CLR-transformed CAZyme data. Associations between variables and CAZyme ALR-transformed data were tested using the same modelling framework applied to microbial taxa, adjusting for technical covariates, delivery mode and feeding mode when these were not the primary variables of interest. Hand-annotated substrate specificity per CAZyme was retrieved^87^. Enrichment analysis was performed using the clusterProfiler::enricher function in R^99^, using FDR-significant associations between CAZymes and timepoint (early versus late), mode of delivery (VG versus CS) and feeding mode (exclusive breastfeeding versus exclusive formula feeding), tested against a background of all detected CAZymes fulfilling the above-mentioned filter.

### Prediction model of maternal gut microbiome and infant eczema

To assess the independent association between maternal gut microbiome alpha diversity and the risk of infant eczema, we first performed a multivariable logistic regression including maternal alpha diversity, smoking history (previously linked to reduced diversity), and family history of allergic disease as covariates. We then evaluated the predictive performance of microbiome-derived features using a leave-one-out cross-validation (LOO-CV) framework with XGBoost classifiers^93^. Three sets of predictors derived from the maternal gut microbiome at birth were tested: (1) taxonomic features, represented by CLR–transformed SGB abundances of mother at birth (filtered at a 30% prevalence); (2) functional pathways, represented by ALR–transformed MetaCyc pathway abundances at birth (filtered at a 50% prevalence); and (3) alpha diversity, represented by the Shannon diversity index of mother at birth. Predictive performance was assessed using ROC curves and AUC metrics. To identify the most influential microbial features contributing to model predictions, SHAP values were computed for the species-level taxonomic mode^100^.

### Bacterial strain transmission between mothers and infants

#### Defining strain-sharing

Phylogenetic distance matrices were extracted from maximum likelihood trees. To define phylogenetic distance cut-offs to identify strain-sharing, we followed a previously published approach^22^. Samples collected within 6 months of each other were assumed to contain the same strain. A genetic distance cut-off is then optimized to separate the distributions of longitudinal samples (assuming they contain the same strain) and unrelated samples (assuming they have different strains). To differentiate between the same and different strains, we established cut-offs for a total of 1,205 species (specific cut-offs per species are shown in Supplementary Table 56). We then investigated strain-sharing between infants at any age (2 weeks and 1, 2, 3, 6, 9 and 12 months) and maternal strains at birth or P28 if birth strains were not available. Per species, interindividual phylogenetic distances were normalized to their maximum distance. We prepared a training distance dataset to define cut-offs for strain-sharing. For this, we defined training samples with the same strain as those from the same subject and taken within 180 days of each other (that is, 6 months). If a single individual had multiple samples taken within 180 days, the shortest time difference was selected. For the training dataset of samples with different strains, we took unrelated samples (not the same individual or family). If multiple timepoints were present between two samples, we randomly selected one.

Using this training dataset, we optimized a Youden cut-off for the distance that better optimized the function ‘sensitivity + specificity – 1’, given that we defined ‘same strain’ as samples collected from the same individual within 180 days, and ‘different strain’ as those collected from unrelated individuals. If the number of training individuals with the same strain was below 50, we used the third quantile of the distribution instead of the Youden cut-off since we believe the Youden cut-offs to be overfitted. If the Youden cut-off was larger than the distance value identified in the fifth quantile, the fifth quantile was used instead as a more conservative metric. Using this approach, we identified cut-offs for 1,205 species. Using the complete distance matrix, we considered two samples to have the same strain of a species if their normalized phylogenetic distance was equal to or lower than the identified cut-off.

#### Strain-sharing associations with predictors

Per species, we removed all pairs of samples that did not belong to mother–infant pairs (that is, each mother with her own offspring). If we did not identify at least two mother–infant pairs, the species was removed from analysis. We then considered only one mother timepoint, either at birth or P28 (as the closest timepoint to birth), to focus on possible vertical transmission events.

Mother–infant strain sharing was then associated with infant age (in days). For this analysis, we focused only on species with over 20 mother–infant pairs (multiple timepoints of the same infant with the same timepoint from mother were used). We further removed species where there were fewer than 10 mother–infant unique pairs (without counting multiple timepoints), or if there were less than 3 samples with at least 5 timepoints available. This allowed us to test for 49 species. We then ran a generalized mixed-effects model with a logit link (logistic regression), where mother–infant strain sharing was used as the dependent variable, while age in days, mother timepoint (P28 or birth) and infant ID (as a random effect) were used as covariates.

To associate mother–infant strain sharing with predictors, we used species with over 20 mother–infant pairs (multiple timepoints of the same infant and the same timepoint from mother were used). Different predictors had different missing rates, and only those complete in at least ten individuals were used for analysis. However, for categorical predictors, we required at least five strain-sharing events per predictor level. Finally, we performed another logistic generalized linear mixed model using strain sharing as the dependent variable and the predictors, mother timepoint, infant timepoint and infant ID as covariates. All associations were merged, and a Benjamini–Hochberg FDR was estimated.

To associate overall sharing rates (that is, not per species, but rather the percentage of strains shared between a mother and an infant per timepoint), we merged all mother–infant distances (using a single mother timepoint as described above) from all species. Then, per mother–infant pair, we assessed the percentage of species for which the same strain was shared. We included only pairs for which we could assess this rate using at least five species. We then ran linear mixed models using the rate as the dependent variable, which was associated with an independent model per predictor, while also accounting for infant and mother timepoints and infant ID as a random effect. Finally, we estimated an FDR from all the predictor associations.

All associations with the predictors duration of ruptured membranes, duration of delivery and place of delivery were only run in VG infants.

#### Strain sharing and bacterial abundance

For each species, we examined whether its CLR-transformed abundance in the infant was associated with whether the strain was shared with the mother (at birth or P28 if no birth sample available). As this effect might be different in early timepoints compared to later timepoints, we labelled the data as early if the infant data were from infants aged 2 weeks or 1 , 2 or 3 months and as late if the data were from infants aged 6, 9 or 12 months. To run the model, we required there to be at least five strain-sharing events and not-strain-sharing events in both early and late timepoints. We then ran a linear mixed-effect model in which the (CLR-transformed) bacterial abundance acted as dependent variable, and the independent variables were whether the strain was shared with the mother, whether the timepoint was early or late and an interaction term between strain-sharing and early/late timepoint, in addition to a random effect with sample ID. We ran this model for all species and extracted the effects of strain-sharing and the interaction between strain-sharing and timepoint. FDR was estimated for these two variables among all tests.

We also associated the mother’s bacterial abundance with the likelihood of strain sharing. For that, we took all available mother timepoints and retained only the early infant timepoints. We required at least five strain-sharing events for association. We associated mother–infant strain sharing, as a dependent variable, with the (CLR-transformed) bacterial abundance of mothers, while controlling for mother timepoint, infant timepoint and mother ID as a random effect. FDR were estimated for the effect of abundance in all tested species.

#### Strain sharing and strain persistence

We defined strain persistence as those strains that were the same within the same infant at an early (2 weeks or 1 month) and late (9 or 12 months) timepoint, choosing the longest distance among the available timepoints. We then matched information about strain persistence with mother–infant strain sharing at either 2 weeks or 1 month. We required at least 20 mother–infant pairs with information about persistence for analysis. We performed a Fisher exact test to estimate whether the odds of being a persistent strain were higher for strains shared between mother and infants (null hypothesis = higher), than those that were not shared. FDRs were estimated.

#### De novo assembly and binning

Quality-filtered and trimmed reads from each sample (including unmatched reads) were assembled into contigs using MetaSPAdes (v.3.15.5) using the default parameters^101^. Bacterial binning was performed separately for each metagenome using metaWRAP (v.1.3.2)^102^. The resulting bins were dereplicated using skDER (v.1.2.7)^103^ with the dynamic approach and the following parameters: --percent-identity-cutoff 98.0 and --aligned-fraction-cutoff 90.0.

#### Functional enrichment of transmitted bacterial strains

High-quality bacterial MAGs (completeness ≥ 90%, contamination < 5%), dereplicated at the subspecies level (98% ANI), were assigned SGB taxonomy using PhyloPhlAn’s phylophlan_assign_sgbs with the mpa_vJan21 MetaPhlAn database, based on MASH distance (<0.05). In total, we identified 645 MAGs that could be assigned to 112 of these SGBs. Metagenomic reads were mapped to selected MAGs using Bowtie2 (v.2.5.1), and mapping files were processed with inStrain (v.1.9.0)^104^ using the profile module (minimum mapQ score = 0; insert size = 160). inStrain ‘compare’ was used to estimate genome similarity across samples by comparing profiles with a minimum genome breadth ≥ 0.5. Only regions with ≥5× coverage were included in the comparison. Sample pairs with <50% comparable regions of the genome were excluded. Strain sharing was assessed based on population-level ANI (popANI), with bacterial strains considered shared between mother and infant if they exhibited ≥99.999% popANI across comparable regions. Protein sequences in MAGs were predicted using prodigal (v.2.6.3)^105^ and were functionally annotated using eggNOG-mapper (v2.1.12)^106^ with the ‘--m hmmer -d 2 --evalue 1e-05’ parameters to retrieve COG annotations. Functional enrichment was assessed by testing whether COGs (encoded in ≥20 MAGs) were transmitted across more unique mother–infant pairs than expected by chance, using a permutation-based test with 10,000 iterations. An FDR < 0.05 was considered significant.

### Association of infant age and maternal and infant traits to SGB phylogeny

To associate time and maternal and infant traits with SGB phylogeny, we used multivariate mixed model distance matrix regression. RAxML phylogenetic trees were transformed to distance matrices by their branch lengths using the cophenetic.phylo function from R package ape (v.5.8-1)^107^. Next, using the MDMR package (v.0.5.2)^108^, we performed mixed model distance matrix regression treating SGB distance as the outcome. To associate phylogenies to time, we included timepoint (factor) and technical covariates (DNA concentration and sequencing depth) as fixed effects and individual ID as random effects. Associations that passed a FDR (Benjamini–Hochberg) cut-off of 5% were considered significant.

$${\rm{Phylogenetic\; distance\;  \sim \; covariates\; +\; Timepoint\; +\; (1|Individual\; ID)}}$$

To associate species phylogenies to maternal and infant predictors, we executed the models with timepoint adjusted as fixed effect:

$$\begin{array}{c}{\rm{Phylogenetic\; distance}} \sim {\rm{covariates}}+{\rm{Timepoint}}+{\rm{Predictor}}\\ \,+(1|{\rm{Individual\; ID}})\end{array}$$

Quantitative predictors were normalized using inverse rank transformation. As the sample size varied across the trees tested (due to differences in species prevalence), we included only predictors of interest that were present in more than 100 samples and for which the most frequent category comprised no more than 75% of observations.

The phylogeny–predictor associations with analytical MDMR *P* values that passed FDR (Benjamini–Hochberg) control of 5% were additionally validated by performing 20,000 permutations and calculating empirical *P* values. The associations were considered significant if both the analytical and empirical *P* value reached the 5% FDR threshold.

### Reporting summary

Further information on research design is available in the Nature Portfolio Reporting Summary linked to this article.

## Online content

Any methods, additional references, Nature Portfolio reporting summaries, source data, extended data, supplementary information, acknowledgements, peer review information; details of author contributions and competing interests; and statements of data and code availability are available at https://doi.org/10.1038/s41586-026-10922-9.

## Supplementary information

Supplementary Information Description of the MEFISTO work process.

Reporting Summary

Supplementary Results Association of predictors to infant gut microbiome week 2 clusters.

Supplementary Tables Supplementary Tables 1–15 and 16–76.

Peer Review File

## Source data

Source Data Figs. 1–5

Source Data Extended Data Figs. 1, 2, 4–10

## Data Availability

Quality-trimmed, human-decontaminated shotgun metagenomic sequencing data are publicly available at the NCBI SRA under accession number PRJNA1468137. Metadata (including questionnaire and clinical information) and quality-trimmed, human-decontaminated sequencing reads are available at the EGA under accession numbers EGAD50000001187 (faecal samples), EGAD50000002469 (breast milk and vaginal samples) and EGAD50000002677 (metadata). Access can be requested through the application form available online (https://forms.gle/A4Jem2rMnjcygWRD6). Access to Lifelines NEXT data is available to all qualified researchers and is governed by the Lifelines NEXT Data Access Agreement (https://groningenmicrobiome.org/?page_id=2598). This controlled-access procedure ensures that metadata are used exclusively for scientific research purposes and in accordance with the informed consent provided by Lifelines NEXT participants. To facilitate data access and reuse, we have prepared a detailed instruction guide describing the EGA access procedure, available at GitHub (https://github.com/GRONINGEN-MICROBIOME-CENTRE/LLNEXT_MOM_MATTERS/blob/main/Data_access_EGA.md). No fees are associated with access to the metadata or sequencing data. Researchers with further questions regarding data access are encouraged to contact the corresponding authors. MAGs recovered from faecal samples are publicly available at Zenodo^109^ (https://zenodo.org/records/18965233). Links to other datasets or databases used in the present study can be found online: Human reference genome GRCh38.p13 (https://www.ncbi.nlm.nih.gov/datasets/genome/GCF_000001405.39/); and MetaPhlAn database of marker genes (mpa_vJan21) (https://doi.org/10.1038/s41587-023-01688-w). Source data are provided with this paper.

## Code availability

All codes used in this study are available at GitHub (https://github.com/GRONINGEN-MICROBIOME-CENTRE/LLNEXT_MOM_MATTERS) and Zenodo^110^ (https://doi.org/10.5281/zenodo.21355872).
