# Supplementary material for: Maternal influences on infant gut microbiome and health
Source: Nature. 2026 Aug 12;658(8134):218–29. doi: 10.1038/s41586-026-10922-9 (PMC13626959; doi:10.1038/s41586-026-10922-9)

nature portfolio

https://doi.org/10.1038/s41586-026-10922-9

Supplementary information

Maternal influences on infant gut microbiome and health

In the format provided by the authors and unedited

MEFISTO analysis workflow for LLNEXT infant microbiome data

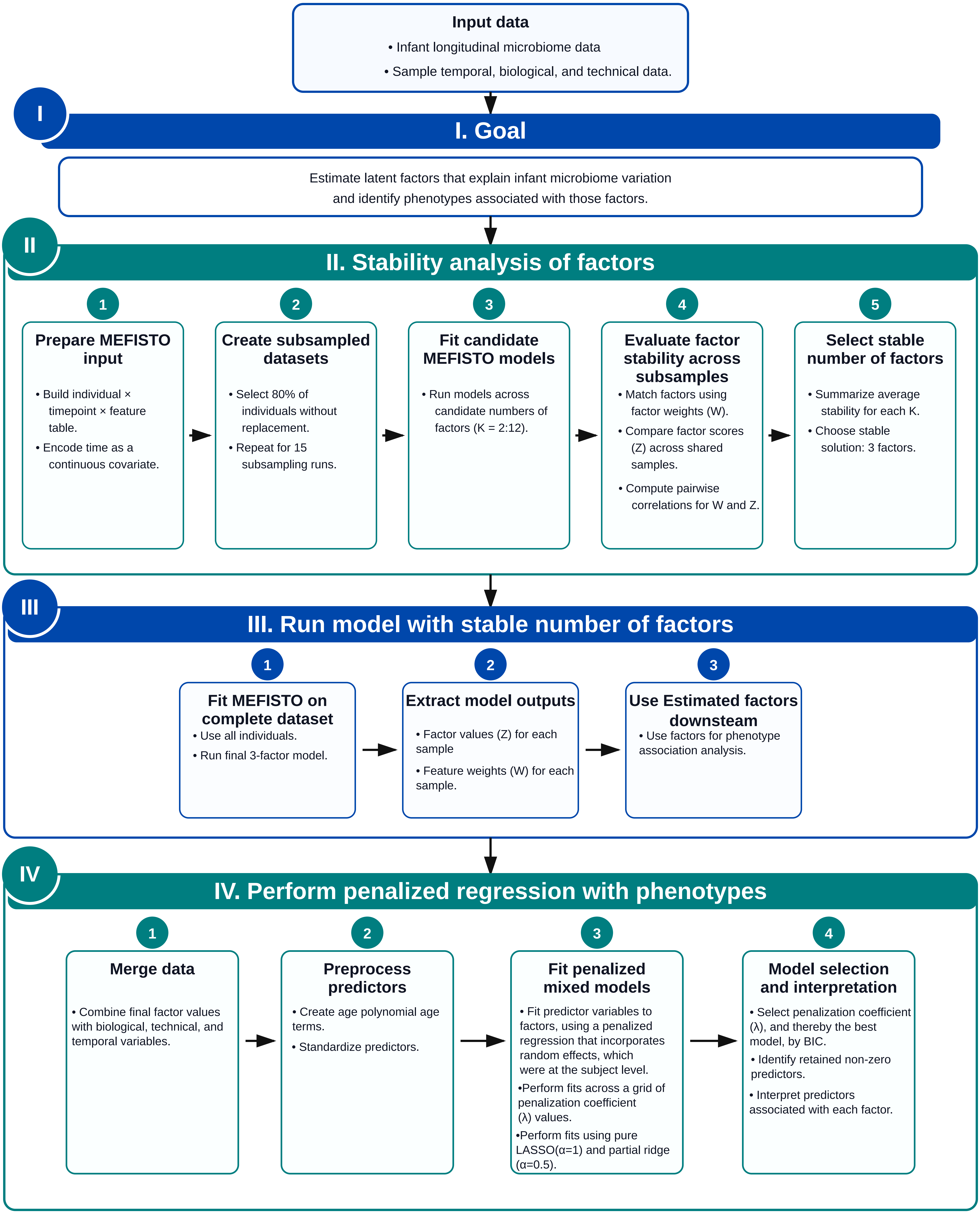

Supplement: Supplementary file 1 [file 41586_2026_10922_MOESM1_ESM.pdf]
