# Supplementary material for: Maternal influences on infant gut microbiome and health
Source: Nature. 2026 Aug 12;658(8134):218–29. doi: 10.1038/s41586-026-10922-9 (PMC13626959; doi:10.1038/s41586-026-10922-9)
Supplement: Supplementary file 2 [file 41586_2026_10922_MOESM2_ESM.pdf]

nature portfolio

Corresponding author(s): T.Sinha, A. Zhernakova

Last updated by author(s): Jun 11, 2026

Reporting Summary

Nature Portfolio wishes to improve the reproducibility of the work that we publish. This form provides structure for consistency and transparency in reporting. For further information on Nature Portfolio policies, see our Editorial Policies and the Editorial Policy Checklist.

Statistics

For all statistical analyses, confirm that the following items are present in the figure legend, table legend, main text, or Methods section.

n/a	Confirmed	
☐	☒	The exact sample size (*n*) for each experimental group/condition, given as a discrete number and unit of measurement
☐	☒	A statement on whether measurements were taken from distinct samples or whether the same sample was measured repeatedly
☐	☒	The statistical test(s) used AND whether they are one- or two-sided<br>*Only common tests should be described solely by name; describe more complex techniques in the Methods section.*
☐	☒	A description of all covariates tested
☐	☒	A description of any assumptions or corrections, such as tests of normality and adjustment for multiple comparisons
☐	☒	A full description of the statistical parameters including central tendency (e.g. means) or other basic estimates (e.g. regression coefficient) AND variation (e.g. standard deviation) or associated estimates of uncertainty (e.g. confidence intervals)
☐	☒	For null hypothesis testing, the test statistic (e.g. *F*, *t*, *r*) with confidence intervals, effect sizes, degrees of freedom and *P* value noted<br>*Give P values as exact values whenever suitable.*
☐	☒	For Bayesian analysis, information on the choice of priors and Markov chain Monte Carlo settings
☒	☐	For hierarchical and complex designs, identification of the appropriate level for tests and full reporting of outcomes
☐	☒	Estimates of effect sizes (e.g. Cohen's *d*, Pearson's *r*), indicating how they were calculated

Our web collection on statistics for biologists contains articles on many of the points above.

Software and code

Policy information about availability of computer code

Data collection	Data from questionnaires, growth charts and birth cards were entered, checked and stored in Utopia and Molgenis Data System and was managed by trained data managers. This data was then exported into a .txt file when was further imported for downstream analysis.
Data analysis	Tools used for this study were: dendextend (v.1.17.1) psych (v 2.2.9) stats (v 4.2.1) BBDuk (v39.01) KneadData tools (v0.10.0) Bowtie2 (v2.4.2) & Bowtie2 (v2.5.1) FastQC toolkit (v0.11.9) MetaPhlAn4 (mpa_vJan21) StrainPhlAn4 (mpa_vJan21) HUMAnN (v3.6) vegan (2.7-1) lmerTest (v3.1-3) WeightedCluster package (v1.6-4) XGBoost (v1.7.9.1) MOFA2 (v1.14.0) glmmPen (v1.5.4.8) mprod package (v0.0.5a1) mmrm package (v 0.3.12) mgcv package (v1.9-1) MetaSPAdes (v3.15.5) metaWRAP (v1.3.2) skDER (v1.2.7) inStrain (v1.9.0) prodigal (v2.6.3) eggNOG-mapper (v2.1.12) ape (v.5.8-1) MDMR package (v.0.5.2)

For manuscripts utilizing custom algorithms or software that are central to the research but not yet described in published literature, software must be made available to editors and reviewers. We strongly encourage code deposition in a community repository (e.g. GitHub). See the Nature Portfolio guidelines for submitting code & software for further information.

Data

Policy information about availability of data

All manuscripts must include a data availability statement. This statement should provide the following information, where applicable:

- Accession codes, unique identifiers, or web links for publicly available datasets
- A description of any restrictions on data availability
- For clinical datasets or third party data, please ensure that the statement adheres to our policy

Quality-trimmed, human-decontaminated shotgun metagenomic sequencing data are publicly available at the NCBI SRA under accession number PRJNA1468137. Metadata (including questionnaire and clinical information) and quality-trimmed, human-decontaminated sequencing reads are available under accession numbers EGAD50000001187 (fecal samples), EGAD50000002469 (breast milk and vaginal samples), and EGAD50000002677 (metadata). Access can be requested through the application form available at https://forms.gle/A4Jem2rMnjcygWRD6. Access to Lifelines NEXT data is available to all qualified researchers and is governed by the Lifelines NEXT Data Access Agreement (https://groningenmicrobiome.org/?page_id=2598). This controlled-access procedure ensures that metadata are used exclusively for scientific research purposes and in accordance with the informed consent provided by Lifelines NEXT participants. To facilitate data access and reuse, we have prepared a detailed instruction guide describing the EGA access procedure, available at: https://github.com/GRONINGEN-MICROBIOME-CENTRE/LLNEXT_MOM_MATTERS/blob/main/Data_access_EGA.md. No fees are associated with access to the metadata or sequencing data. Researchers with further questions regarding data access are encouraged to contact the corresponding authors.

MAGs recovered from fecal samples are publicly available through Zenodo at: https://zenodo.org/records/18965233.

Links to other datasets or databases used in the present study can be found here: Human reference genome GRCh38.p13 https://www.ncbi.nlm.nih.gov/datasets/genome/GCF_000001405.39/; MetaPhlAn database of marker genes (mpa_vJan21); https://doi.org/10.1038/s41587-023-01688-w;

Research involving human participants, their data, or biological material

Policy information about studies with human participants or human data. See also policy information about sex, gender (identity/presentation), and sexual orientation and race, ethnicity and racism.

Reporting on sex and gender	Our study deals with pregnant women and their infants. For their infants we use the term "sex" referring to the biological sex assigned to them by the healthcare professionals at the hospital they were born in. These included 348 female infants and 366 male infants (SI 4)
Reporting on race, ethnicity, or other socially relevant groupings	No socially relevant categorization variables such as race or ethnicity were used in the current manuscript.
Population characteristics	The mother-infant pairs that collected their samples for this study were a part of Lifelines NEXT cohort, a birth cohort designed to study the effects of intrinsic and extrinsic determinants on health and disease in a four-generation design (Warmink-Perdijk 2020, 10.1007/s10654-020-00614-7). Lifelines NEXT is embedded within the Lifelines cohort study, a prospective three-generation population-based cohort study recording the health and health-related aspects of 167,729 individuals living in the Northern Netherlands. From 2016 to 2023, 1,452 pregnant women were recruited for LLNEXT, along with their partners and infants, and monitored up to at least 1year after birth.<br><br>For this study we included the first 714 mother-infant pairs from the Lifelines NEXT cohort. Average maternal age at delivery was 32 years (SD:3.94; Table S7), with 45.1% (n=183) of mothers delivering their first infant (i.e. parity is 0) (Table S8). Most infants (92.7%, n=662) were born full-term, with 84.8% (n=585) delivered vaginally and 22.5% (n=155) born at home (Table S4). Most infants (83.8%, n=430) were breastfed at birth, 49.7% (n=259) were exclusively breastfed at 3 months of age (M3) and 18.4% (n=42) were still exclusively breastfed by the age of 12 months (M12) (Tables S4 and S6). Twelve sets of twins and 18 mothers who gave birth to two infants during the study period were also included (Table S4).
Recruitment	This study is part of a larger population cohort study (Lifelines NEXT). Recruitment took place by the healthcare professional of the pregnant woman or through self enrollments of interested participants. All pregnant candidates were invited to participate in Lifelines NEXT without any exclusion criteria. Informed consent forms were available for all participants and all participants were 18 years or older at the time of sample collection. Participants of the present study were enrolled from 2016 to 2021, without any bias in selection, besides the availability of longitudinal fecal samples throughout pregnancy and the 1st year of life. We acknowledge that our study is susceptible to self selection bias. Voluntary participation in this cohort skews the participation to more health aware women and women with higher educational background.
Ethics oversight	The ethical board of the University Medical Centre Groningen approved this study (METc 2015/600). Study procedures were only carried out after a written informed consent was obtained.

Note that full information on the approval of the study protocol must also be provided in the manuscript.

Field-specific reporting

Please select the one below that is the best fit for your research. If you are not sure, read the appropriate sections before making your selection.

☒ Life sciences ☐ Behavioural & social sciences ☐ Ecological, evolutionary & environmental sciences

For a reference copy of the document with all sections, see nature.com/documents/nr-reporting-summary-flat.pdf

Life sciences study design

All studies must disclose on these points even when the disclosure is negative.

Sample size	No sample size was calculated for our study (714 mother-infants pairs) due to the exploratory nature of the study.
Data exclusions	Briefly, DNA was successfully isolated from 4,724 faecal samples, of which 4,673 were successfully sequenced. Thereafter, 26 samples with a low read depth (<5 million reads) were excluded, leaving 4,647 samples. After removing 28 isolation and sequencing controls, 4,619 samples remained. Subsequently, 19 samples were excluded because of major outliers (between mothers and infants), duplicate samples, or known laboratory mix-ups identified solely from the sequencing data, without consideration of any predictor variables, leaving 4,600 samples. We then removed 23 samples with >75% unclassified reads as estimated by MetaPhlAn4, combined with a high abundance of known contaminants identified from negative controls, leaving 4,577 samples. Finally, 51 maternal samples collected at one and two months postpartum were removed because the sample sizes at these time points were too small for meaningful analysis and comparison with other timepoints. This resulted in a final dataset of 4,526 gut microbiome samples from mothers and infants. For breast milk, 90 of 91 isolated samples were successfully sequenced and included in the analyses. For vaginal samples, 82 of 91 isolated samples were successfully sequenced and included in the analyses.
Replication	Not applicable for this observational metagenomic study
Randomization	Not applicable, not an intervention study
Blinding	Not applicable, not an intervention study

Reporting for specific materials, systems and methods

We require information from authors about some types of materials, experimental systems and methods used in many studies. Here, indicate whether each material, system or method listed is relevant to your study. If you are not sure if a list item applies to your research, read the appropriate section before selecting a response.

Materials & experimental systems

n/a	Involved in the study
☒	☐ Antibodies
☒	☐ Eukaryotic cell lines
☒	☐ Palaeontology and archaeology
☒	☐ Animals and other organisms
☒	☐ Clinical data
☒	☐ Dual use research of concern
☒	☐ Plants

Methods

n/a	Involved in the study
☒	☐ ChIP-seq
☒	☐ Flow cytometry
☒	☐ MRI-based neuroimaging

Plants

Seed stocks	*Report on the source of all seed stocks or other plant material used. If applicable, state the seed stock centre and catalogue number. If plant specimens were collected from the field, describe the collection location, date and sampling procedures.*
Novel plant genotypes	*Describe the methods by which all novel plant genotypes were produced. This includes those generated by transgenic approaches, gene editing, chemical/radiation-based mutagenesis and hybridization. For transgenic lines, describe the transformation method, the number of independent lines analyzed and the generation upon which experiments were performed. For gene-edited lines, describe the editor used, the endogenous sequence targeted for editing, the targeting guide RNA sequence (if applicable) and how the editor was applied.*
Authentication	*Describe any authentication procedures for each seed stock used or novel genotype generated. Describe any experiments used to assess the effect of a mutation and, where applicable, how potential secondary effects (e.g. second site T-DNA insertions, mosiacism, off-target gene editing) were examined.*
