# Supplementary material for: Maternal influences on infant gut microbiome and health
Source: Nature. 2026 Aug 12;658(8134):218–29. doi: 10.1038/s41586-026-10922-9 (PMC13626959; doi:10.1038/s41586-026-10922-9)
Supplement: Supplementary file 3 [file 41586_2026_10922_MOESM5_ESM.pdf]

nature portfolio

Peer Review File

Maternal influences on infant gut microbiome and health

Corresponding Author: Dr Trishla Sinha

Any redactions in this file are there to maintain patient confidentiality, the confidentiality of unpublished data, or to remove third-party material.

This file contains all reviewer reports in order by version, followed by all author rebuttals in order by version.

Version 0:

Reviewer comments:

Referee #1

(Remarks to the Author)
This study by Sinha et al. is a commendable investigation into maternal and early-life microbiomes and their influencing factors. The authors leverage a unique dataset of 4,577 longitudinally collected metagenomes from a cohort of mother-infant pairs, which is a significant contribution to the field. This effort undoubtedly advances our understanding of early-life microbiome development. The authors also confirm several well-established findings, including maternal-to-infant microbial strain transmission and the roles of infant feeding and delivery mode in shaping the microbiome.
Despite these strengths, I have concerns about the statistical methods employed, insufficient functional investigations (which limit the translational potential of this study), and overinterpretations based on underpowered analyses. Furthermore, the limited exploration of connections between the developing microbiome and host health outcomes, despite intriguing results related to infant eczema, represents another missed opportunity to strengthen the study's impact. My detailed comments are as follows:

1. Lines 140-142:
"We observed subtle yet significant changes in the overall maternal gut microbiome composition over time (Figure 1c, Table S16), with 111 species changing significantly with time (Figure S1a, Table S17)."
• I would like clarification on how the PERMANOVA was conducted and whether repeated measurements were accounted for. For instance, an HMP2 study (PMID: 31142855) employed a PERMANOVA method that accommodates repeated measures, which may be more suitable for this dataset.
• I could not locate the details of the statistical model used to analyze species-level changes over time, which limits my ability to evaluate the robustness of these findings.
Given that the authors use Aitchison distance as the primary beta-diversity metric, it would be helpful to include details on how zero values were handled. Were pseudo-counts applied for the log-ratio transformation? Additionally, how do the main findings compare if Bray-Curtis dissimilarity is used instead?

2. Lines 186-189:
"Neither late time point communities (at M6, M9, and M12) nor maternal communities before birth could accurately predict W2 clustering (area under the curve (AUC) Ppermutations > 0.05), demonstrating that the developing infant gut microbiome follows a non-deterministic maturation path."
• I was unable to find the data or visual presentations supporting this claim.

3. Regarding Figures 2a and 2b, it is unclear how repeated measurements were accounted for in the analysis. Additionally, were the results multivariable-adjusted? Given the observational nature of the study, univariate analyses would be highly susceptible to confounding. Multivariable models and adjustments for multiple comparisons should be applied for robust inference.

4. The MEFISTO model was trained on 10 predefined factors. How was the number 10 determined? More detail on this selection process would enhance the transparency of the methodology.

5. Figures 2 and 3 Analyses:
• Many of the tests appear hypothesis-free; it is not clear to me how multivariable adjustment and correction for multiple

comparisons were made, which raises concerns about the validity of the reported associations.

6. Lines 296-300:
"When investigating the relationships of individual bacterial species with infant-specific predictors, we found 424 significant associations, including 186 with dietary-related factors (113 related to feeding mode), 73 with delivery-related factors (such as delivery mode and birth complications), 46 with stool frequency and structure, 20 with parity and 5 with infant body weight."
• Again, for such a large number of associations, multivariable adjustment and corrections for multiple comparisons are essential to avoid false positives. This is especially critical given the hypothesis-free nature of these analyses.

7. Several analyses, such as those described in Lines 354-366 and the cluster-based analysis in Lines 179-189, appear to be underpowered. Results from these tests should be interpreted with caution and may not be suitable for robust inference. Overinterpretation of these findings detracts from the overall rigor of the study.

8. While the authors generated functional profiling of the microbiome, the functional results are sparse throughout the manuscript. This omission limits the study's translational relevance, particularly in linking host factors such as dietary intake to microbiome function. The paper will be enhanced by including more functional data.

9. Lines 397-399:
"Alpha diversity was lower in mothers who gave birth in hospitals compared to those who delivered at home (FDR = 0.006), with this difference already evident at P28."
• As acknowledged by the authors, this finding is likely confounded. Reporting such results without addressing confounding through multivariable models may not be appropriate.

10. Lines 480-484:
• Previous strain-sharing analyses (e.g., PMID: 36653448) excluded phylogenetically close MAGs of food origin (e.g., Streptococcus thermophilus, S. salivarius, S. vestibularis) upfront. The rationale for including these in the current study is unclear and should be explained.

11. Much of the strain-sharing analysis appears confirmatory and replicates findings from prior studies, such as PMID: 36653448. While replication is valuable, the novelty of these results should be better highlighted.

12. Lines 569-570:
"We then investigated the relationship between infant and maternal factors and species phylogenies, adjusted for sampling time."
• Adjusting for sampling time may not be the most appropriate approach given the repeated sampling design. Including time as a random effect would better account for within-subject correlations.

(Remarks on code availability)

Referee #2

(Remarks to the Author)
The study by Sinha, Brushett et al. investigates the dynamics of gut microbiomes of mothers and infants during pregnancy and early life in relation to their effects on health. The authors analysed a very comprehensive sample set comprised of 4,577 fecal samples from 714 mother-infant pairs in the Dutch LLNEXT cohort, during pregnancy to one year postpartum. Key findings include:

1. The maternal gut microbiome changes subtly during pregnancy and postpartum, influenced by diet, infections, and smoking history.
2. Significant mother-to-infant microbial strain transmission occurs, with higher maternal abundances increasing transmission likelihood.
3. Maternal gut diversity before birth is a strong predictor of infant eczema.
4. Infant gut microbiome is shaped by delivery mode and feeding, with other factors like maternal smoking and parity affecting specific species.
5. Early (2 weeks) infant microbiome composition does not predict later health outcomes.

The study by Sinha, Brushett et al. represents the most extensive study of the pregnancy and early life microbiome to date. The impact of microbiome impacts during critical stages of human life remains an important research topic. Hence, the present study is timely as it provides insights for potential future microbiome-targeted therapies. Overall, the analyses performed of the very extensive dataset result in new insights and involve state-of-the-art methods. The manuscript is well written and all figures are clear.

Major comments

1. The authors should provide a clear rationale for not having obtained infant samples from timepoints prior to two weeks

postpartum (this is the earliest timepoint considered). Earlier work has in particular highlighted differences between infants born vaginally and those delivered by caesarian section. If possible, the authors should seek to analyse data from earlier timepoints or at least contextualise their findings, e.g. with respect to Bacteroides spp., in relation to these earlier studies.
2. The authors' analyses are very focused on taxonomically resolved micrbiome data including diversity metrics. As such, the analyses are lacking with respect to detailed functional and pathway-level analyses. Earlier work has for example highlighted functional microbiome differences related to delivery mode in relation to important immunomodulatory molecules such as LPS (e.g. PMIDs: 27133167 & 30504906) or vitamins (PMID: 35295713). The authors should perform more precise function-directed analyses and relate this data to the extensive set of clinical, dietary and exposure variables.
3. With respect to the strain-tracking analyses relating the mothers' and infants' microbiomes, it would be insightful to understand which gut or other reservoirs the shared strains (Bacteroides spp., Bifidobacterium spp., Streptococcus spp. etc.) come from. This is in particular important to understand the routes of transfer from mothers to infants in view of the development of future interventions. Additional sample sets might exist which the authors could leverage to ascertain the origins of specific strains.

Minor comments

L.161: "B. breve"
L.170-172: The intra- versus inter-individual alongside intra- versus inter-familial variations observed for the infant microbiome could be related to the patterns observed more generally for humans.
L.214-216: The MEFISTO analysis should be explained in more detail and a rationale for selecting 10 factors should be provided.
L.225: Figure 2c should be relabelled as Figure 2b to fit the flow of the text.
L.292: "mode of delivery".
L.381: "sweets and fats and oils".
L. 517: "B. thetaiotaomicron"

(Remarks on code availability)
The paths to the code used could be clearer. I assume that the following path was the one to follow (?):
NEXT_pilot_microbial_transmission_mother_infant/

Referee #3

(Remarks to the Author)
Sinha et al. present a comprehensive longitudinal analysis of the maternal and infant gut microbiomes using data from 714 mother-infant pairs in the Dutch Lifelines NEXT cohort. This is a well-designed and ambitious study that aims to address the impact of microbiome changes occurring during pregnancy and the infant's first year of life on 474 clinical/dietary variables.

Beyond confirming several well-documented associations, the study offers additional insights into how the maternal microbiome is especially affected by diet, infections and smoking history. One particularly interesting observation was that the level of diversity in the maternal microbiome was found to be a key predictor of eczema in infants. Furthermore, they also show that early-life colonizers of the infant gut do not necessarily predict how the microbial community will establish later in life. I found this to be a very high-quality and in-depth study that further advances our understanding of microbial transmission and assembly in early life, a key area of microbiome research. My main criticism is that there are virtually no biological insights/mechanisms discussed underpinning their associations. This seems like a wasted opportunity with such high-quality data. In addition, as mentioned above, several of their associations (feeding mode, mother-infant strain sharing, etc. have already been covered in prior works such as in Shao et al. Nature 2019).

1) The strength of the study is undoubtedly the detailed associations established between microbial markers of pregnancy and birth with health outcomes. However, there is limited effort to extract biological meaning from such interactions. Given the rich metagenomic data collected, it is a shame there is such a superficial analysis performed at a functional pathway level. The authors state there are >1,000 associations with various predictors, but there is no comparison on the strength of associations between taxonomy vs pathways and pinpointing specific functions linked to health outcomes.

2) The authors show that maternal alpha diversity has high predictive power of infant eczema. Beyond overall diversity, were there specific species showing a stronger association? They should compare model accuracy in using the microbiome composition (e.g., species/functions) vs alpha diversity alone.

3) How was the non-independent nature of strains across timepoints within the same subject taken into account in their phylogenetic analysis (e.g., Lines 553 onwards)? They find that late colonizers are different compared to early colonizers. But how many instances did the authors find of the same strain being maintained from one timepoint to the next in the same individual vs introduction of new strains later on?

4) Why were read depth, DNA concentration and batch number the only variables corrected for in the association tests? Was there a statistical basis for this? I would imagine other aspects such as diet and medication would need to be considered as well.

5) Different prevalence and abundance thresholds were used for mothers (0.001% abundance, 30% prevalence) compared to infants (0.1% abundance and 10% prevalence). This does not make much sense to me. The authors should decide on the

thresholds that maximize accuracy and apply them consistently to all samples.

6) How was a CLR transformation applied to the MetaPhlAn results? CLR transformation is typically applied to read count data (e.g., see the ALDEx2 approach), but MetaPhlAn outputs relative abundance data. This should be carefully checked to ensure their approach complies with best practices in the field.

7) The definition of “parity” for the purpose of this study should be defined early on (i.e., infants with siblings vs without siblings).

8) Line 292: Typo “pf” -> “of”

(Remarks on code availability)
The code appears to have been made available. However, the authors should provide in their README more detailed instructions on the structure of the folders/scripts and how to replicate the analysis in their manuscript. At the moment, the repo's structure is quite confusing and makes it hard to reproduce their work. In addition, the current README mentions both virome and multi-omics analysis which do not appear to be relevant for this study (although would be definitely worth including if available).

Version 1:

Reviewer comments:

Referee #1

(Remarks to the Author)
Thank you to the authors for their efforts in improving this manuscript and for responding to my earlier comments. While the paper has been strengthened in certain areas, I continue to have serious concerns about several key methodological issues.
1. Unaddressed concern over the robustness of the findings in my comments 3, 5 and 6.
My main concern regarding the robustness of the findings, raised previously in comments 3, 5, and 6, has not been addressed. The authors claim that their “sequential association analysis” mimics stepwise regression or fine-mapping. While this analogy may make sense in genetics, where SNPs are largely independent and the linkage structure is well characterized, it is not appropriate for phenotype-based observational data. In such data, stepwise conditioning can inflate type I error rates due to repeated testing and model selection, overfit the data, particularly when the sample size is small relative to the number of phenotypes (which is the case in this study), and distort effect estimates, as variable inclusion depends on prior P-values rather than substantive reasoning. This practice leads to unstable and data-dependent inference, which is problematic for a paper intended for a high-impact journal.
The analogy to GWAS fine-mapping is also conceptually flawed. In GWAS, conditioning on top SNPs helps disentangle causal variants within linkage disequilibrium blocks, where the correlation structure is known. In contrast, the “phenotypes” in this study (e.g., birth mode, feeding type, antibiotic exposure) are interrelated, and causal directionality is unclear. Sequential conditioning risks treating mediators or colliders as confounders, resulting in biased estimates. Furthermore, the statement that Bonferroni correction was applied “in both the initial and corrected rounds” lacks clarity: the correction should be applied across all models and rounds. Otherwise, the family-wise error rate will be inflated. Bonferroni correction also assumes test independence, an assumption unlikely to hold given the correlated nature of both the phenotypes and microbial factors. Therefore, the claim that results were “adjusted for multiple comparisons” does not convincingly demonstrate proper control of false positives. I remain concerned that many reported associations are likely false positives and that this revision did not adequately address this fundamental issue.
2. The authors’ response to comment 4
The justification for selecting ten factors based on a subjective “balance between interpretability and fit” is insufficient without additional evidence from model selection steps such as marginal likelihood, cross-validation, or held-out likelihood. The explanation that an “elbow” appears at factor six, while only the first five are biologically meaningful, yet ten are retained for “flexibility,” reflects internal inconsistency and suggests potential overfitting. A clearer presentation of cumulative variance explained and validation of additional factors using independent data or metadata would be useful. Moreover, the manuscript lacks evidence of model diagnostics or robustness checks; there is no indication that alternative factor numbers were tested, factor stability was evaluated, or redundancy among factors was examined. The reference to a “stepwise regression strategy” is even more confusing by conflating the unsupervised MEFISTO factor inference with the subsequent association testing, implying potential post hoc justification of factor number based on downstream associations. Such reasoning introduces bias and undermines confidence in the analytical rigor.
3. The authors’ response to comment 7,
While I appreciate the authors’ acknowledgment that many tests are underpowered, this admission reinforces my concern about the overall robustness and interpretability of the findings. When combined with an analysis framework that inflates significance and increases the likelihood of false positives, the scientific validity of the reported results becomes even more questionable.
4. The response to comment 10
This response also remains unconvincing. The authors have not clearly explained how dietary data were controlled in their analyses to achieve the purpose they claim. Also, it would be helpful to know whether sensitivity analyses excluding mothers who consume yogurt were performed. The argument that S. thermophilus had “some of the lowest levels of strain-sharing” does not constitute justification for inclusion; it merely shows the species behaves differently, without demonstrating that this behavior supports the study’s conclusions.
5. The authors’ response to comment 12

This response does not directly address my comment at all. The authors instead addressed confounding by time but not for dependence due to repeated measures. Their choice to include only one (earliest) strain per species per individual is a data simplification rather than a proper statistical solution, as it discards within-subject information, reduces power, and introduces potential bias if early versus late colonizers differ systematically. The justification that more complex models failed to converge reflects computational limitations, not methodological rationale, and is not an appropriate and justifiable response. Alternatives, such as simplifying random effects structures, using penalized likelihoods, or employing frequentist mixed models, should have been considered before discarding repeated measures entirely.
Moreover, the manuscript conflates analyses of timepoint-phylogeny and phenotype-phylogeny associations without a clear description of how repeated measures and individuals were treated in each case. This creates confusion and raises concerns about data independence. The assertion that "within-subject correlation is not an issue" is incorrect, as microbial species within an individual remain correlated even when only one timepoint per species is used. The hierarchical nature of the data, species nested within individuals, nested within families, violates independence assumptions and should be explicitly modeled.
The authors also claim to have conducted "extensive testing" but provide no diagnostics or summary statistics to substantiate this, such as the number of models that failed to converge or comparisons of one-timepoint versus multi-timepoint analyses. Without such evidence, their explanation reads as post hoc justification rather than a transparent modeling decision. Conceptually, their reframing of the analysis from longitudinal within-individual dynamics to cross-sectional comparisons between early and late colonizers may have biological interest, but it represents a fundamentally different research question and should be presented as such.

(Remarks on code availability)

Referee #2

(Remarks to the Author)
The authors have performed a thorough revision of their original manuscript. I have the following remaining comment:

Functional and pathway-level analyses: In line with my previous comments and given their role in potentially influencing health in later life, I would invite the authors to specifically check their data for the biosynthesis of immunomodulatory molecules such as LPS (e.g. PMID: 30504906) or vitamins (PMID: 35295713), and discuss their results in the context of these earlier studies.

(Remarks on code availability)

Referee #3

(Remarks to the Author)
I thank the authors for addressing all my comments. I particularly appreciate the added functional analysis focusing on carbohydrate utilisation and the additional results showing limited strain sharing between mother breast milk and infant gut samples. I believe this will be a useful addition to our understanding of microbiome dynamics in pregnancy and early life.

(Remarks on code availability)
The authors have improved the documentation of the code, providing separate description of the scripts used in their GitHub repo for the different analyses sections of their project.

Version 2:

Reviewer comments:

Referee #1

(Remarks to the Author)
The authors have addressed all of my comments satisfactorily. I appreciate their efforts to improve the manuscript.

(Remarks on code availability)

Referee #2

(Remarks to the Author)
No substantive concerns remain from my last set of comments. The only minor caveat is that the overall manuscript is now very complex, and some methods may be difficult for readers to follow without more schematic illustrations. However, this is more of a stylistic and editorial comment, and not so much a concern regarding the scientific elements of this important study.

(Remarks on code availability)

Open Access This Peer Review File is licensed under a Creative Commons Attribution 4.0 International License, which permits use, sharing, adaptation, distribution and reproduction in any medium or format, as long as you give appropriate credit to the original author(s) and the source, provide a link to the Creative Commons license, and indicate if changes were made.
In cases where reviewers are anonymous, credit should be given to 'Anonymous Referee' and the source.
The images or other third party material in this Peer Review File are included in the article's Creative Commons license, unless indicated otherwise in a credit line to the material. If material is not included in the article's Creative Commons license and your intended use is not permitted by statutory regulation or exceeds the permitted use, you will need to obtain permission directly from the copyright holder.
To view a copy of this license, visit https://creativecommons.org/licenses/by/4.0/

Rebuttal manuscript “Mom Matters: Maternal influences on infant gut microbiome and health in 714 mother-infant pairs in the Lifelines NEXT cohort”.

Referee expertise:

Referee #1: human microbiome, epidemiology, statistical genetics

Referee #2: human microbiome, multi-omics

Referee #3: human microbiome, metagenomics, computational and bioinformatics analysis

Referees' comments:

Referee #1 (Remarks to the Author):
Referee #1: human microbiome, epidemiology, statistical genetics

This study by Sinha et al. is a commendable investigation into maternal and early-life microbiomes and their influencing factors. The authors leverage a unique dataset of 4,577 longitudinally collected metagenomes from a cohort of mother-infant pairs, which is a significant contribution to the field. This effort undoubtedly advances our understanding of early-life microbiome development. The authors also confirm several well-established findings, including maternal-to-infant microbial strain transmission and the roles of infant feeding and delivery mode in shaping the microbiome.
Despite these strengths, I have concerns about the statistical methods employed, insufficient functional investigations (which limit the translational potential of this study), and overinterpretations based on underpowered analyses. Furthermore, the limited exploration of connections between the developing microbiome and host health outcomes, despite intriguing results related to infant eczema, represents another missed opportunity to strengthen the study’s impact. My detailed comments are as follows:

Reply: We thank the reviewer for their comments. We have substantially revised the manuscript according to their suggestions. Firstly, we have reviewed and refined our statistical methods throughout the manuscript to ensure greater robustness, as further outlined in our answers to the specific questions below. Secondly, we have also revised the language to avoid overinterpretation of findings, particularly where power may be limited. Where applicable, we now explicitly acknowledge limitations related to statistical power. Thirdly, we have carried out additional functional investigations to improve the translational potential of this study, as described in detail in our response to Question 8.

Please find the answers to specific questions below.

Question 1. Lines 140-142:

"We observed subtle yet significant changes in the overall maternal gut microbiome composition over time (Figure 1c, Table S16), with 111 species changing significantly with time (Figure S1a, Table S17)."

1.1 • I would like clarification on how the PERMANOVA was conducted and whether repeated measurements were accounted for. For instance, an HMP2 study (PMID: 31142855) employed a PERMANOVA method that accommodates repeated measures, which may be more suitable for this dataset.

Reply: We thank the reviewer for this comment and are happy to clarify.

For this analysis, we first constructed a distance matrix using Aitchison distance, for both mothers and infants separately. We then performed classical multidimensional scaling and extracted the first 20 principal components for mothers and infants separately. Each of these components in mothers and infants was subsequently associated with a time point using a linear mixed-effects model, with sample ID included as a random effect. The results of these associations can be found in supplementary tables S16 and S17, and the procedure is described in the Methods: "The effect of time on the first 20 principal components was calculated using time point as a fixed effect in a mixed model using again the lmerTest package."

Following the reviewer's suggestion, we have now also validated this analysis with a PERMANOVA restricted to within-patient sample permutations and obtained significant association of overall microbiome composition with time, with R^2 of 7.78% for infants and of 0.77% for mothers (both $P < 1e-4$, number of permutations=10,000). We have now updated the main text with these values and updated the Methods accordingly:

"The association of microbial beta diversity with time was also validated using PERMANOVA (adonis2 analysis of 'vegan' package) with constraint to within-subject sample permutations to derive a P-value and R2."

1.2 • I could not locate the details of the statistical model used to analyze species-level changes over time, which limits my ability to evaluate the robustness of these findings.

Reply: We apologize for this oversight. To analyze species-level changes over time, we used linear mixed-effects models using time as the explanatory variable and the CLR-transformed SGB relative abundance as an outcome. Each time point was tested using P12 as the reference for mothers and W2 as the reference for infants. This information has now been added to the Methods:

"To calculate the beta diversity within and between samples, we used Aitchison distances performed on filtered CLR-transformed data. The effect of time on the first 20 Principal Components and the CLR-transformed SGB relative abundances was calculated using time point as a fixed effect in a mixed model using again the lmerTest package with the same covariates. P12 was used as the reference for mothers and W2 as the reference for infants."

1.3 Given that the authors use Aitchison distance as the primary beta-diversity metric, it would be helpful to include details on how zero values were handled. Were pseudo-counts applied for the log-ratio transformation? Additionally, how do the main findings compare if Bray-Curtis dissimilarity is used instead?

Reply: The zero values were handled as follows. First, zeros were adjusted by adding half of the lowest non-zero value to all zero-value species. Then, all taxa were CLR-transformed using the geometric mean of the relative abundance of microbial species as the CLR denominator. The corresponding text in the Methods reads: "CLR-transformation was then applied at SGB level and at higher taxonomic levels. All microbial taxa, regardless of taxonomic level, were CLR-transformed using the geometric mean of the relative abundance of microbial species as the CLR denominator. Since CLR-transformation cannot be applied to zero values, zeros were adjusted by adding half of the lowest non-zero value."

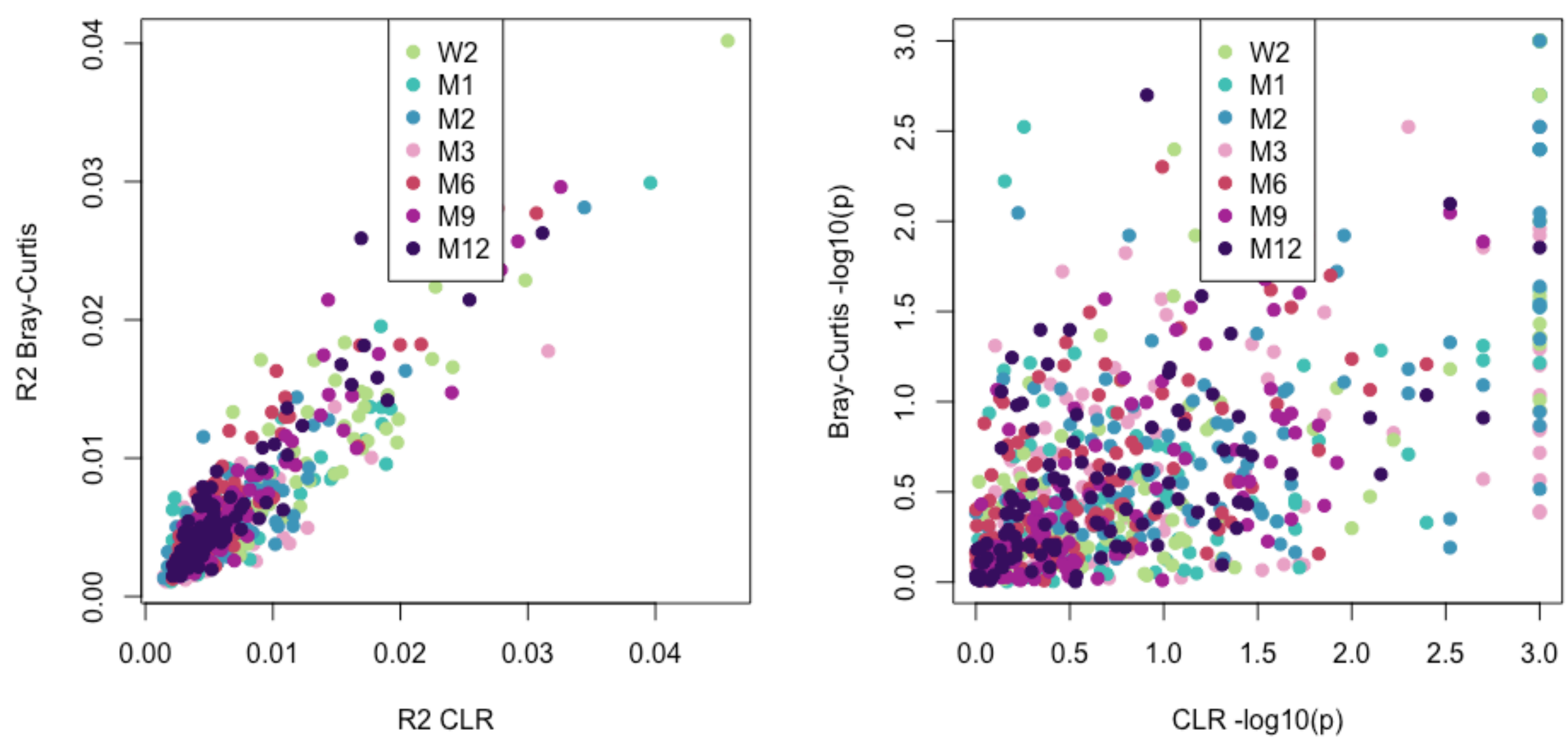

We have also replicated the analysis of the association of various factors with the infant gut microbiome using Bray-Curtis dissimilarity instead of Aitchison distance. As shown in the plot above, the R^2s derived from the distance matrix variance explained by each of the factors tested, e.g., delivery mode where each point represents Adonis run for one factor at one timepoint) of Adonis show a high degree of concordance between Aitchison and Bray-Curtis dissimilarity (left panel). However, the corresponding permutation *P*-values tend to be smaller for Aitchison distance (right panel). This can only be explained by higher spread for the null distribution of F statistics reconstructed by permutations observed for Bray-Curtis dissimilarity. Due to the generally sparser microbiome composition of infants compared to adults, we believe that this is likely to be an artefact of compositionality, with Bray-Curtis dissimilarity affected more severely than Aitchison distance (with the latter was originally proposed to reduce the compositionality

artefacts, see Gloor et al, *Front Microbiol* 2017, PMC5695134). We therefore decided to use Aitchison distance as our main distance measure for all downstream analyses.

2. Lines 186-189:
"Neither late time point communities (at M6, M9, and M12) nor maternal communities before birth could accurately predict W2 clustering (area under the curve (AUC) Permutations > 0.05), demonstrating that the developing infant gut microbiome follows a non-deterministic maturation path."
• I was unable to find the data or visual presentations supporting this claim.

Reply: We thank the reviewer for pointing this out. Indeed, we did not add a visual presentation of this data in our previous submission. We have now included a supplementary figure (Figure S3, see below) displaying the AUC resulting from leave-one-out cross-validation (LOO-cv). We have now updated the analysis, switching from a 10-fold cross-validation with AUCs as score to a more reliable LOO-cv scheme and from elastic net to XGBoost predictions. In concordance with the initial findings, we observe low predicted class probabilities and relatively low AUCs.

We now describe this update analysis in the Methods:
"Next, we assessed the community composition of the clustered samples at late time points (M6, M9 and M12) and attempted to predict the early W2 clustering based on the microbial composition at these later time points. We built a multi-class XGBoost prediction model using R's XGBoost v1.7.9.1 (objective: multi:softprob, default parameters)89. We applied this prediction using a compositional data analysis approach90 by generating all possible log-ratios between bacterial species abundances with >20% prevalence in a leave-one-out cross-validation (LOO-cv) setting. We performed the same analysis using the microbial community from maternal samples at P12, at either P28 or birth and at M3."

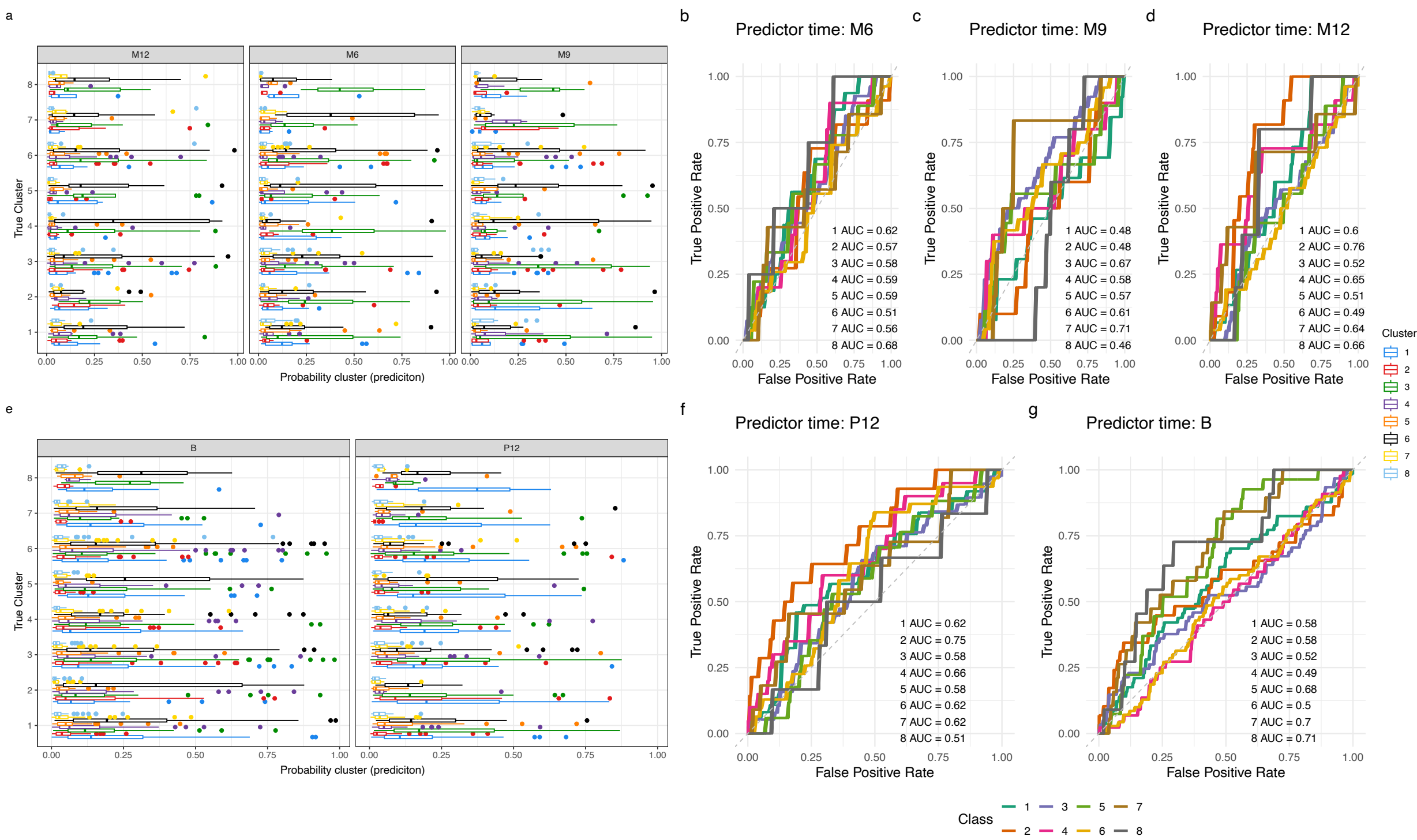

Figure S3. Prediction of early-life clusters using late-time points and maternal community data. **a,e**. Prediction of early time point clusters using a, late infant time points and e, maternal time points. Plots display the prediction probability per sample and per predicted class (color) per true cluster correspondence. High predictive probabilities do not correspond to cluster-belonging. **b,c,d**. AUC-ROC curves of the prediction of each cluster, using early infant time points and **f,g.** maternal time points.

3.1 Regarding Figures 2a and 2b, it is unclear how repeated measurements were accounted for in the analysis.

Reply: The purpose of this analysis was to understand which of the most significant phenotypes are influencing infant microbiome colonization and how they change over time. For this, we employed a temporal factor analysis, MEFISTO (Velten et al., *Nature Methods*, 2022; PMID: 35027765). In MEFISTO, time is added as a covariate in the estimation of factors, i.e., time is integrated directly into the modeling of factors. Technically, this is done by modeling factors as a Gaussian process, where the difference in factor values between samples depends on their distance in time.

3.2 Additionally, were the results multivariable-adjusted? Given the observational nature of the study, univariate analyses would be highly susceptible to confounding. Multivariable models and adjustments for multiple comparisons should be applied for robust inference.

Reply: Yes, these results were multivariable-adjusted, both for technical and biological covariates and also adjusted for multiple comparisons. Our aim was to identify which infant phenotype most significantly explained each factor while correcting for other

confounding technical and biological covariates. To achieve this, a set of phenotypes known to promote taxonomic variation of the infant microbiome were associated with each factor (see Methods). The modeling strategy employed can best be described as a sequential association analysis with a stepwise correction. This strategy is analogous to fine-mapping in genome-wide association studies where, after an initial round of testing each SNP individually, the top-associated SNP is added as a covariate in the next round of models. This stepwise conditioning helps unmask additional signals that may be hidden in the first round and reveals which SNP has the strongest effect on the phenotype.

In brief, all 13 phenotypes were initially associated to each of the factors, adjusting for time and technical covariates related to each sample's microbiome sequencing (DNA concentration, read depth and batch number). Each phenotype-factor pair was thus tested separately, and significance was assessed with the likelihood ratio test. The phenotype with the lowest *P*-value was considered the strongest association to the factor. To correct for potential confounding effects and reveal a more accurate relationship between this 'most significantly associated' phenotype and the other phenotypes, we performed an additional sequence of associations. In this next round, models were refitted for the remaining 12 phenotypes, with the addition of the most significant phenotype from the first round of associations as a corrective covariate. If the *P*-value of any of the now-corrected phenotypes was lower than that of the initially most significant phenotype, the corrected phenotype with the lowest *P*-value would be considered the strongest association. In both the initial and corrected round of analyses, models were corrected for multiple testing via Bonferroni correction. This modeling strategy was carried out per factor.

All in all, the strategy described above allowed us to evaluate whether the effect of a phenotype was previously masked by a more dominant, potentially confounding phenotype. This procedure is described in the **Methods** section of the manuscript:

"A set of 13 predictors known to exert strong ecological selective pressures on the colonization of the infant microbiome12,23 were modeled against each factor. These predictors were birth mode, feeding mode, duration of delivery, place of delivery, parity, pet ownership, infant eczema diagnosis, infant birth weight, infant food allergy, sex, maternal age at delivery, duration of broken water during delivery and maternal smoking history. The modeling strategy employed a sequential association analysis with a stepwise correction. In brief, all 13 predictors were initially fit to each of the factors, adjusting for time and covariates related to each sample's microbiome sequencing, including the DNA concentration, read depth and sequencing batch number. Each predictor-factor pair was thus tested separately, and significance was assessed with the likelihood ratio test. The predictor with the lowest P-value was considered the strongest association to the factor. This first round of associations is referred to as the 'initial_model' in Table S21. To both correct for potential confounding effects and reveal a more accurate relationship between the most significantly associated predictors and the other predictors, we performed an additional sequence of associations. These are referred to as the 'corrected_model' in Table S21. In this next round, models were refitted for the remaining 12 predictors, adding the most significant predictors from the first round of associations as a covariate. If the P-value of any of the now-corrected

predictors was lower than that of the predictor that was initially most significant, the corrected predictor with the lowest P-value was considered the strongest association. In both the initial and corrected round of analyses, models were corrected for multiple testing via Bonferroni correction. This modeling strategy was carried out per factor. This correction procedure did not change the most strongly associated predictors from the first round of fitting, with the exception of Factor 10, where parity was considered the strongest association after correction for feeding mode."

4. The MEFISTO model was trained on 10 predefined factors. How was the number 10 determined? More detail on this selection process would enhance the transparency of the methodology.

Reply: We determined that using 10 factors strikes a balance between interpretability and optimal fitting of the data. In general, fitting fewer factors tends to yield more biologically interpretable results, as the variance explained by each factor—and the underlying biological process(es) it represents—is less likely to be split across factors. On the other hand, while increasing the number of factors can help capture more subtle variance structures, it also increases the risk that a single biological signal is fragmented across multiple factors, which can obscure interpretation.

In our analysis, we observed an "elbow" in the variance explained by each factor at Factor 6 (see the figure below). This elbow marks the point where the drop in variance explained per factor sharply levels off. This suggests that the first five factors account for most of the structured variation in the data and are thus the key biological variables (i.e., phenotypes) driving variation of the infant microbiome. Subsequent Factors 6–10 explain incrementally smaller amounts of variance. As such, adding more factors would likely not contribute to the overall interpretability of the data. In addition, while we could have run the analysis with only five factors, we chose to retain 10 to allow the model to detect finer-grained patterns of variation. This stepwise regression strategy was insightful for Factor 10, which showed that parity was the most significant phenotype after correcting for feeding mode.

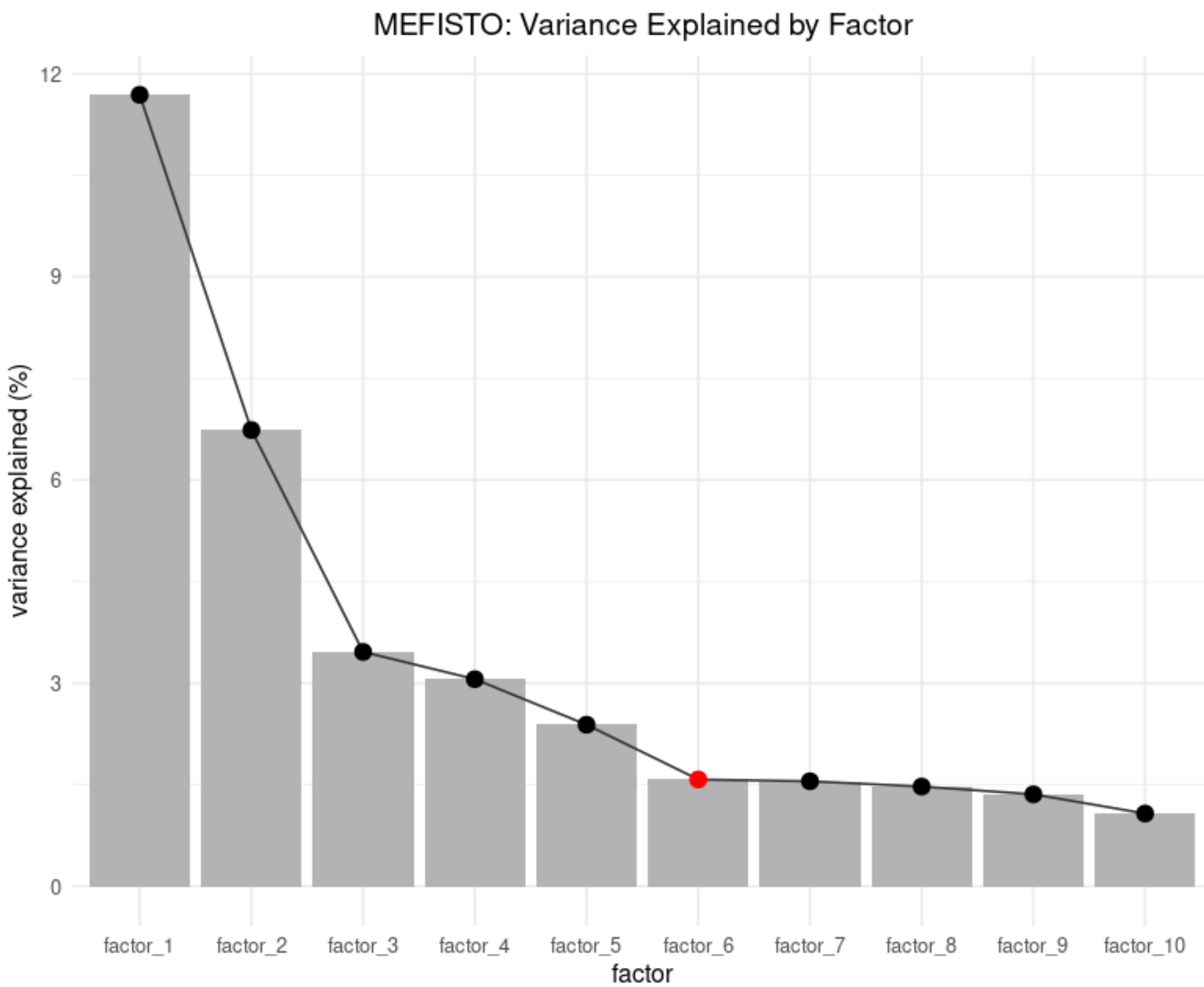

Variance explained by each factor in the MEFISTO analysis. *Each bar represents one of the ten factors estimated by MEFISTO and the proportion of variation explained in the microbial dataset. The red dot represents the elbow–the factor where the rate of decrease in variation explanation begins to level off.*

5. Figures 2 and 3 Analyses:
• Many of the tests appear hypothesis-free; it is not clear to me how multivariable adjustment and correction for multiple comparisons were made, which raises concerns about the validity of the reported associations.

Reply: We thank the reviewer for their comments. Below, we provide more explanation regarding the details of the analysis shown in Figure 2 and Figure 3.

Figure 2 a–d
The analyses underlying this figure were multivariable-adjusted, both for technical and biological covariates and also consistently apply multiple testing correction.
Indeed, the true underlying process or processes that contribute to the variation driving a factor estimated by MEFISTO is likely due to a mix of biological variables. Our aim was to identify the biological variable (i.e., phenotype) that most significantly explained each factor, while correcting for other confounding technical and biological covariates.

To achieve this, a set of phenotypes known to exert strong ecological selective pressures on the colonization of the infant microbiome were modeled against each factor. The modeling strategy employed can best be described as a sequential association analysis with a stepwise correction. In brief, all 13 phenotypes were initially fit to each of the factors, adjusting for time and technical covariates related to each sample's microbiome sequencing (DNA concentration, read depth and batch number). Each phenotype-factor pair was thus tested separately, and significance was assessed with the likelihood ratio test. The phenotype with the lowest *P*-value was considered to have the strongest association to the factor.

To both correct for potential confounding effects and reveal a more accurate relationship between the most significantly associated phenotype and the other phenotypes, we performed an additional sequence of associations. In this next round, models were refitted for the remaining 12 phenotypes, with the addition of the most significant phenotype from the first round of associations as a covariate. Should the *P*-value of any of the now-corrected phenotypes be lower than that of the initially most significant phenotype, the corrected phenotype with the lowest *P*-value would be considered the strongest association. In both the initial and corrected round of analyses, models were corrected for multiple testing using Bonferroni correction. This modeling strategy was carried out per factor. Given the stepwise approach described above, the analysis of phenotype-factor associations was therefore multivariable-adjusted, accounting for both technical and biological covariates.

All in all, the strategy described above allowed us to evaluate whether the effect of a phenotype was previously masked by a more dominant, potentially confounding phenotype. This procedure is described in the **Methods** section of the manuscript (see section of MEFISTO above).

For all further analyses in this study, we corrected for basic technical variables (DNA concentration, read depth and batch number). Further, based on the results of the MEFISTO analysis described above—where mode of delivery and feeding mode were identified as major drivers of infant gut microbiome composition—all analyses were also adjusted for these variables unless otherwise stated. In all supplementary tables reporting associations between factors and microbial species, pathways, gut–brain modules or CAZymes, the corrected estimates and *P*-values are provided. We also consistently apply multiple testing correction and report FDR-significant results, unless specified otherwise.

Figure 2e
Only FDR-significant factors associated with infant alpha diversity, corrected for mode of delivery and feeding mode (apart from these factors themselves), are shown in this figure.

Figure 2f
Only significant factors (permutation-based $P < 0.001$ with 10,000 permutations, corrected for delivery mode and feeding mode, apart from these factors themselves), are shown in this figure.

Figure 3 a, b
This analysis was performed exclusively in vaginally delivered infants using only birth-related factors to address a very specific hypothesis: 'What birth factor determines the abundances of *Bacteroides* in vaginally delivered infants?' Hence only correction for technical variables (DNA concentration, read depth and batch number) was performed. Here we report only nominally significant results, depicted with a P instead of FDR in results and Figure.

Figure 3c
This figure shows only associations with feeding mode, and correction was only performed for technical variables (DNA concentration, read depth and batch number) and mode of delivery.

Figure 3d
This association of acetate synthesis I and feeding mode is corrected for technical variables and mode of delivery and is FDR significant (see **Tables S31** and **S32**).

Figure 3e
This new figure does not relate to association with variables.

Figure 3f
Performed only in early versus late timepoint in infants. Only FDR-significant factors results shown (see **Table S48**)

6. Lines 296-300:
"When investigating the relationships of individual bacterial species with infant-specific predictors, we found 424 significant associations, including 186 with dietary-related factors (113 related to feeding mode), 73 with delivery-related factors (such as delivery mode and birth complications), 46 with stool frequency and structure, 20 with parity and 5 with infant body weight."
• Again, for such a large number of associations, multivariable adjustment and corrections for multiple comparisons are essential to avoid false positives. This is especially critical given the hypothesis-free nature of these analyses.

Reply: For all analyses in this study, we corrected for basic technical variables (DNA concentration, read depth and batch number). Furthermore, based on the results of the MEFISTO analysis, which identified delivery mode and feeding mode as major drivers of infant gut microbiome composition, all analyses were also adjusted for these variables unless otherwise stated. In all supplementary tables reporting associations between factors and microbial species, pathways, gut–brain modules or CAZymes, the corrected estimates and *P*-values are provided. We also consistently apply multiple testing correction and report corrected FDR-significant results (indicated with FDR throughout the manuscript), unless noted otherwise. Lastly, when looking at infant dietary patterns in relation to the infant microbiome we correct for feeding mode and total kcal intake by infant, as also specified in the text.

7. Several analyses, such as those described in Lines 354-366 and the cluster-based analysis in Lines 179-189, appear to be underpowered. Results from these tests should be interpreted with caution and may not be suitable for robust inference. Overinterpretation of these findings detracts from the overall rigor of the study.

Reply: We thank the reviewer for this comment and have revised the text to stress this.

Lines 179-189

"Subsequently we assessed whether the infant gut microbiome at W2, which was largely dominated by the singular bacterial species mentioned above, was deterministic of the later infant gut microbiome composition. Cluster analysis identified eight distinct community compositions at W2, comprising a range of 65 (Cluster 1) to 12 infants (Cluster 8). Clusters 1, 2, 3, 4 and 7 were dominated by a single highly abundant taxon: *E. coli* (SGB10068), *B. bifidum* (SGB17256), *B. longum* (SGB17248), *B. breve* (SGB17247) or *Bacteroides fragilis* (SBG1855 group), respectively (**Figure S2**). Neither late time point communities (at M6, M9 and M12) nor maternal communities before birth could accurately predict W2 clustering (area under the curve (AUC) $P_{permutations}$>0.05, Figure S3), demonstrating that the developing infant gut microbiome follows a non-deterministic maturation path. However, our study might still be underpowered to capture this effect."

Lines 354-366

"We then sought to investigate whether HMOs in breast milk were associated with the gut microbiome of exclusively breastfed infants in the first three months of life. We observed that maternal secretor negative status (Le-Se-) resulted in a lower Shannon diversity index in infants compared to other milk groups (*P*=0.006); however, the power of this analysis was low given the small number of mothers with this status (n=9 samples from three individuals; **Figure S5a**, **Table S34**). In the same group, the abundance of *Clostridium perfringens* (SGB6191) was increased, with nominal significance (*P*=0.0001, **Figure S5a**). No FDR-significant associations with any of the 24 measured HMOs and microbial species were found (**Table S35**). Similarly, we also did not find any associations of HMOs with bacterial pathways (**Table S36**). These results contrast with several smaller previous studies that reported associations between HMOs and the abundance and dynamics of *Bifidobacterium* species[28–30] but are in line with other studies reporting no or weak associations[31–33]. It is possible that the lack of observed associations in our study reflects limited statistical power, particularly given that individual HMOs may have small effect sizes and exhibit high variability, even within a single feeding session. Furthermore, 83.8% of infants in our cohort were breastfed immediately after birth (**Table S4**), which may have reduced variation in early HMO exposure, as the vast majority of infants were exposed to HMOs."

8. While the authors generated functional profiling of the microbiome, the functional results are sparse throughout the manuscript. This omission limits the study's translational relevance, particularly in linking host factors such as dietary intake to microbiome function. The paper will be enhanced by including more functional data.

Reply:
We agree with the reviewer. To enhance functional insights, we have undertaken the following steps:

Given the relatively limited capacity of the infant digestive enzyme repertoire and their high reliance on microbial carbohydrate metabolism, we performed comprehensive functional annotation of the **Carbohydrate-Active Enzyme (CAZyme) profile** of the infant gut microbiome. This revealed dynamic changes throughout the first year of life. In contrast to adults, the infant CAZyme landscape was predominantly driven by the presence of *Bacteroides* species, with delivery mode playing a major role by influencing *Bacteroides* abundance. Further substrate-specific analysis showed that infants delivered vaginally were enriched for CAZymes involved in non-starch polysaccharide metabolism and those receiving breast feeding with CAZymes involved in resistant oligosaccharides, mucin and human milk glycans. Prior to solid food introduction, infants showed a significant enrichment of CAZymes involved in the metabolism of resistant oligosaccharides and starch, peptidoglycan, amylose, pullulan and amylopectin. In contrast, later timepoints were significantly enriched in CAZymes associated with glycoprotein, pectin and arabinogalactan metabolism. Finally, we observed significant associations between specific CAZyme subfamilies and infant growth trajectories.

We further conducted deeper investigations into **bacterial functional pathways using MetaCyc**, which revealed associations not only with delivery mode and infant feeding but also with parity, infant stool frequency and, notably, maternal dietary patterns. These findings reinforce the impact of maternal nutrition on the infant microbiome.

Lastly, a new analysis of **gut–brain modules** revealed compelling associations between infant feeding and pathways related to acetate synthesis, with formula feeding associated with lower acetate synthesis, potentially linking infant nutrition with microbial production of neuroactive metabolites.

These new results have been incorporated into the text (see below) and are shown in amended main **Figure 3** and new supplementary figures **S4, S5 and S6**.

With regards to the analysis of CAZymes we added the following text:

"Infant CAZyme profile is highly dynamic during the first year of life, shaped by the presence of *Bacteroides*, *Parabacteroides* and *Phocaeicola*"

Given the relatively limited capacity of the infant digestive enzyme repertoire and their high reliance on microbial carbohydrate metabolism, we annotated the carbohydrate-active enzyme (CAZyme) profiles of the infant and maternal gut microbiomes to investigate their dynamics and association with exposures. Similar to microbiome composition, the maternal CAZyme profile remained stable during pregnancy, while the infant CAZyme profile was highly dynamic throughout the first year of life (**Figure S5b** and **c; Table S41**). In the infant gut, *Bacteroides*, *Parabacteroides* and *Phocaeicola* were the main contributors to CAZyme profile variation (**Figure 3e, Table S42**), whereas *Bacteroides* played a less prominent role in the maternal gut (**Figure S5d, Table S43**). Most CAZyme associations were linked to mode of delivery, driven primarily by differences in the presence or absence of genus *Bacteroides*, *Parabacteroides* and

Phocaeicola between the groups (**Figure S6**). Interestingly, after investigate associations with infant-specific traits (**Table S44** and **Table S45**) we also found significant associations of infant weight with CAZyme subfamilies even after correction for delivery and feeding mode, with the Glycoside Hydrolase Family 89 (GH89) being associated with infant weight, length and head circumference (FDR=7.12×10^{-6} , 4.95×10^{-4} and 4.74×10^{-4}, respectively; **Table S45**). Further annotation of CAZyme substrates revealed that infants delivered vaginally were enriched for CAZymes involved in non-starch polysaccharide metabolism (NSP), whereas those delivered by CS showed enrichment for starch-metabolizing CAZymes (overrepresentation analysis, FDR=0.002 and 0.008, respectively; **Table S46**). These findings highlight the substantial role of delivery mode in shaping the functional potential of the infant gut microbiome.

In accordance with expectations, breastfeeding was associated with an enrichment of CAZymes related to the metabolization of resistant oligosaccharides, mucin and human milk glycans compared to formula feeding (FDR=0.013, 0.001, 0.000 respectively; **Table S47**). Compared to later time points, earlier time points showed a significant enrichment of CAZymes involved in the metabolism of resistant oligosaccharides and starch, peptidoglycan, amylose, pullulan and amylopectin (**Figure 3f, Table S48**). In contrast, later time points were significantly enriched in CAZymes associated with glycoprotein, pectin and arabinogalactan metabolism (**Figure 3f, Table S48**). This pattern reflects the rapid and dynamic development of the gut microbiome's functional capacity to metabolize diverse substrates during early infancy.

With regards to bacterial pathways, we added the following text:

"When investigating the relationships between individual bacterial species of the infant gut microbiome and infant-specific predictors, we identified 425 significant associations. However, the majority of these were related to delivery and feeding mode, and 193 associations remained after correction for these variables (**Tables S27** and **S28**). Associations were mostly linked to parity, infant stool consistency, frequency and diet (**Tables S27** and **S28**). Interestingly, we observed few associations with some of the unique predictors captured through our detailed questionnaires. For example, only single associations were found for sleeping and no FDR-significant associations were found with crying time (**Tables S27** and **S28**).

We observed 280 bacterial metabolic pathways of the infant gut microbiome significantly associated with 51 variables, with the highest numbers linked to feeding mode (238), parity and gravidity (169), infant stool frequency (162) and delivery-related factors (84), together mirroring the associations observed with bacterial species (**Tables S29** and **S30**). Some pathway associations were mainly driven by a single bacterial species, e.g. the negative association of PWY-5845 (menaquinol-9 biosynthesis) to infant fish and meat consumption was mainly driven by a decrease in abundance of *E. coli* genes belonging to this pathway. For other pathways, the association of pathway to predictors was driven by multiple bacteria. For example, the positive association of PWY-7013 (S-propane-1,2-diol degradation) to formula feeding was driven by the combined pathway-contributing genes of several *Klebsiella* species, *Salmonella* enterica, *Enterobacter cloacae*, *E. coli* and other species. Similarly, the positive association of LACTOSECAT-

PWY (lactose and galactose degradation I) with formula feeding is driven by the pathway genes of multiple *Enterococcus* and *Streptococcus* species (**Figure S4**). After adjusting for delivery and feeding mode, we identified parity, gravidity and stool frequency to be associated with the majority of infant pathways. Additionally, infant complementary feeding was associated with numerous pathways, including 22 linked to bread intake (primarily metabolic pathways) and 12 related to meat/fish intake. Intriguingly, maternal dietary patterns were also associated with 36 infant microbial pathways, reinforcing the potential impact of maternal nutrition on the developing infant microbiome (**Table S29**)."

With regards to infant microbial neuroactive potential, we added the following text:
We next examined associations between the estimated abundance of gut–brain modules (GBMs)[27] and infant-specific predictors. We identified 125 FDR-significant associations, the majority of which were related to infant feeding mode (**Tables S31** and **S32**). Among the most significant findings, we observed a decrease in the quinolinic acid degradation pathway in formula-fed infants, while the acetate synthesis I pathway was significantly enriched in breastfed infants (**Figure 3d**). These results highlight potential differences in microbial neuroactive metabolism linked to early-life nutrition.

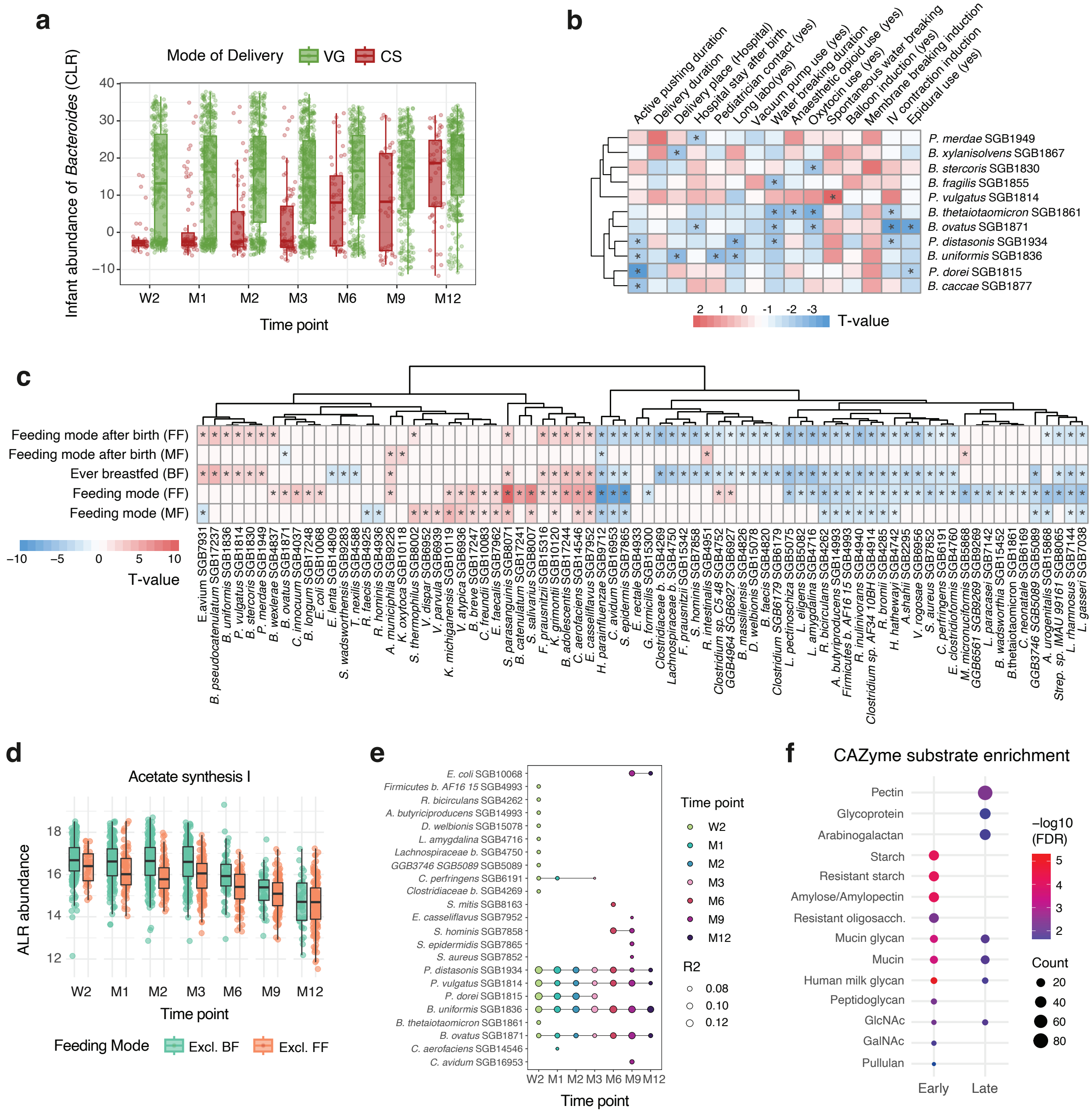

***Figure 3. Associations of infant gut microbiome and CAZyme profile with predictors. a**, Boxplots of the association between the centered log ratio (CLR)-transformed relative abundance of Bacteroides and delivery mode across different time points. **b**, Heatmap of T-values for the association between birth-related factors and various Bacteroides species. * P-value < 0.05. **c**, Heatmap of T-values for associations between feeding mode at birth, feeding mode from 2 weeks to 12 months of age and ever being breastfed or not with bacterial species at species-level genomic bin (SGB) level. * FDR < 0.05. **d**, Boxplots of the additive log ratio (ALR)-transformed abundance of acetate synthesis with either exclusive breastfeeding or formula feeding over time. **e**, PERMANOVA of CLR-transformed SGB abundance on the Aitchison's distance matrix of the entire CAZyme profile of infants at seven time points. Only significant bacteria (permutation P-value <0.0001 and R^2 >0.06) are shown. For full results see **Table S42**. **f**, Overrepresentation analysis of CAZyme substrates at early (3 months or younger) versus late (6 months or older) time point of infants. Only FDR-significant (<0.05) results are shown. For **a** and **d**, each boxplot visualizes the median, hinges (25th and 75th percentiles) and whiskers extending up to 1.5 times the interquartile range from the hinges.*

New supplementary figures pertaining to this part are as follows:

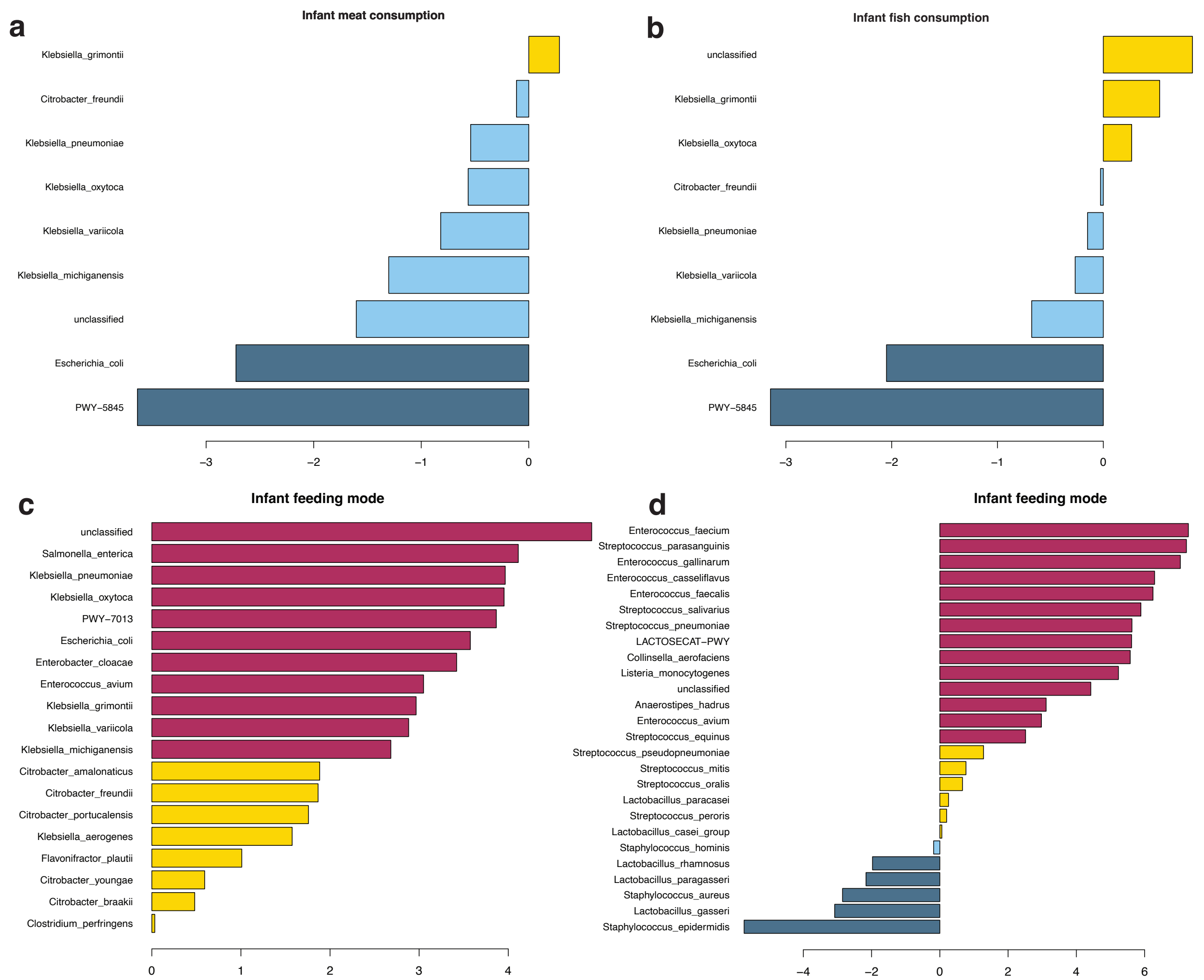

***Figure S4. Per-species pathway abundance association to phenotypes**. X-axis denotes T-statistics of association of total pathway abundance (encoded with METACYC ID). Species names represent the association T-values of total abundance of genes belonging to a pathway from individual species. The "Unclassified" category represents association of pathway genes with no taxonomic binning.*

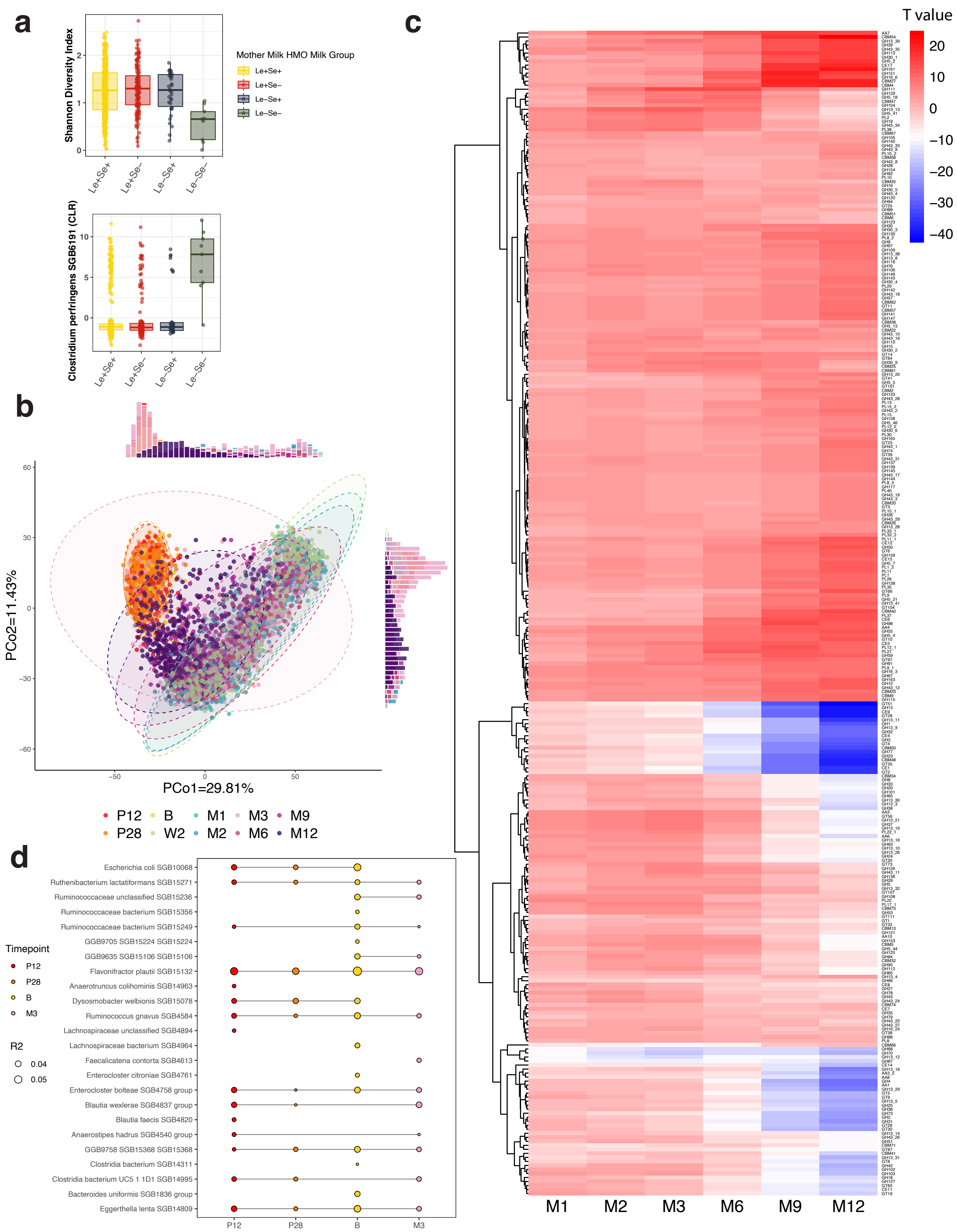

Figure S5. Maternal milk group and HMO associations and maternal and infant CAZyme profile. a, *Maternal secreter status and infant Shannon diversity index (above) and infant relative abundance of*

Clostridium perfringens (CLR-transformed) (below). ***b,*** *PCoA analysis based on Aitchison distance calculated at the subfamily level of maternal and infant CAZyme profile.* ***c,*** *T-values of the association of infant time point with CAZyme subfamilies using W2 as the reference.* ***d,*** *PERMANOVA of CLR-transformed SGB abundance on the Aitchison's distance matrix of the entire CAZyme profile of mothers at various time points.*

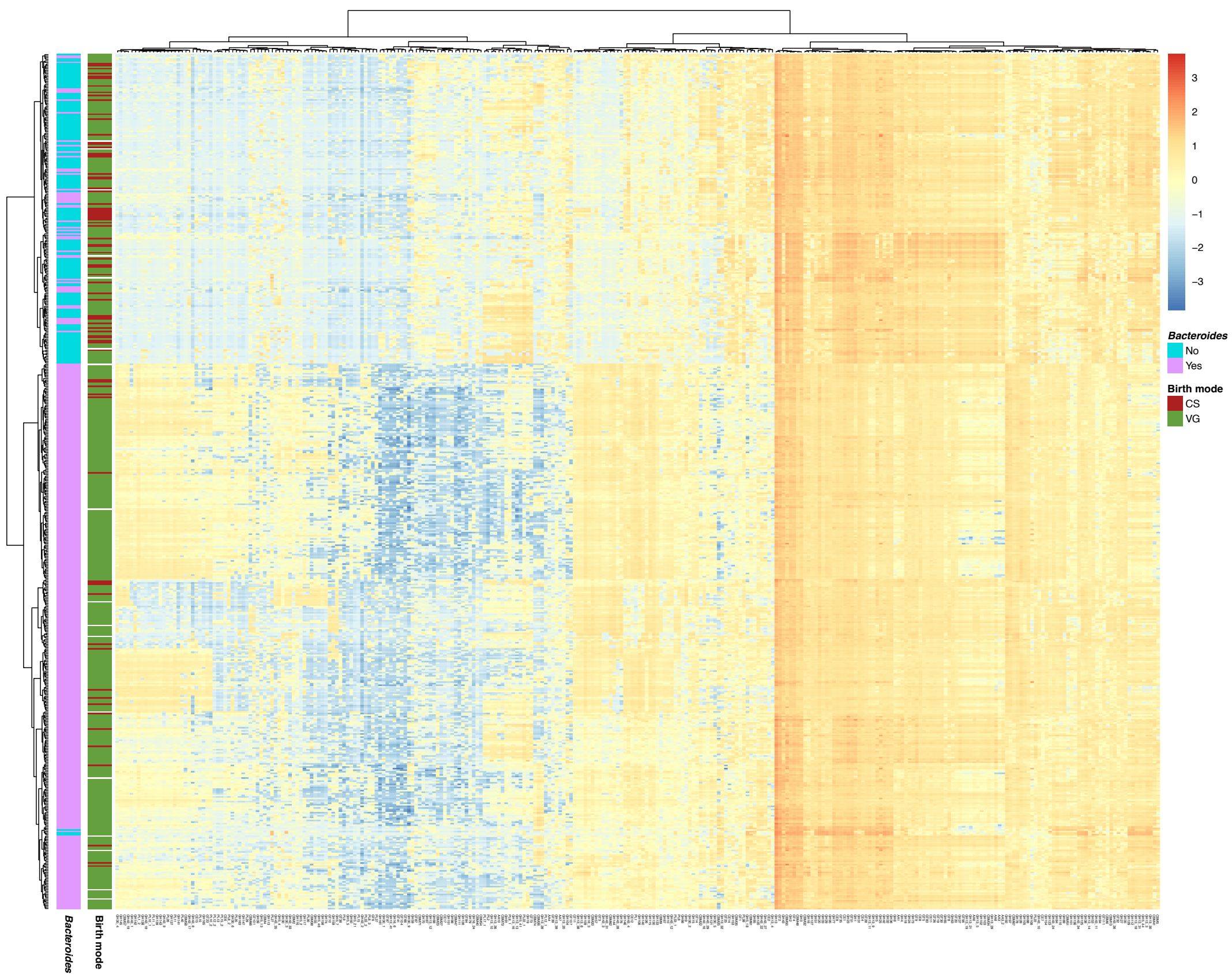

Figure S6. Bacteroides is the major driver of infant CAZyme profile*. Heatmap of the infant CAZyme profile at month 3 (M3), with the X-axis representing CAZyme subfamilies. The Y-axis includes bar plots indicating the presence or absence of Bacteroides and the mode of delivery (vaginal (VG) vs. cesarean section (CS)).*

9. Lines 397-399:
"Alpha diversity was lower in mothers who gave birth in hospitals compared to those who delivered at home (FDR = 0.006), with this difference already evident at P28."
• As acknowledged by the authors, this finding is likely confounded. Reporting such results without addressing confounding through multivariable models may not be appropriate.

Reply: We agree that the observed association between lower alpha diversity and hospital birth is likely confounded by maternal health and risk profiles. In the Dutch healthcare system, home birth is generally reserved for women with a low medical risk profile and absence of previous obstetric and birth complications (Amelink-Verburg et

al., 2008, PMID: 18162116). Women delivering at home are thus typically in better overall health and may also engage in healthier lifestyle behaviors, both of which have previously been linked to increased microbiome diversity (Zhernakova et al., 2016, PMID: 27126040; Manor et al., 2020, PMID: 33060586).

In **Figure 4a** and **Table S49,** we examined associations between alpha diversity and a range of obstetric and maternal lifestyle-related phenotypes. Specifically, as shown in **Table S49**, several pregnancy complications were related to Shannon diversity. Of these, hypertension showed a significant association with reduced alpha diversity (P=0.02), although this did not remain significant after FDR-correction and was thus not included in Figure 4. Similarly, gestational diabetes mellitus (GDM) was also associated with lower diversity, but this result did not reach statistical significance, likely due to limited power (only 40 women in the GDM group). In addition, we observed significant associations between lower Shannon diversity and other health-related variables, including higher pre-pregnancy BMI, positive smoking history, current smoking and exposure to second-hand smoke (**Table S49**).

Taken together, these findings suggest that alpha diversity in pregnant women reflects their broader health and lifestyle context. We have now revised this section in the text to better illustrate this:

"We next associated 292 maternal-specific factors (**Tables S7-S10**) with maternal gut microbiome features. Maternal alpha diversity was significantly associated with several food preferences, delivery mode and place of delivery, gestational age, maternal education, pre-pregnancy BMI and smoking, urinary tract infections, gastroenteritis and stool characteristics (**Figure 4a, Tables S49** and **S50**). Notably, alpha diversity was lower in mothers who gave birth in hospitals compared to those who delivered at home (FDR = 0.006), with this difference already evident at P28 (**Figure 4b, Table S49**). In the Netherlands, home birth is relatively common, and 22.5% of vaginal deliveries in our cohort occurred at home. However, as this option is only recommended for women with a low medical risk profile and no prior obstetric complications[36], women who deliver at home tend to be in better overall health that those who give birth in non–midwife-led hospital settings. Better overall health has previously also consistently been linked to higher gut microbiome diversity[37,38]. In our study, lower alpha diversity in women was also significantly associated with several indicators of poorer health, including higher pre-pregnancy BMI, smoking exposure and pregnancy complications such as hypertension (**Figure 4a, Table S49**). These findings suggest that the observed difference in diversity with delivery setting likely reflects underlying differences in maternal health and lifestyle."

10. Lines 480-484:
• Previous strain-sharing analyses (e.g., PMID: 36653448) excluded phylogenetically close MAGs of food origin (e.g., Streptococcus thermophilus, S. salivarius, S. vestibularis) upfront. The rationale for including these in the current study is unclear and should be explained.

Reply: We agree that the inclusion of food-derived bacterial strains in strain-sharing analyses requires careful consideration. In meta-analyses with limited metadata, such

as that by Valles-Colomer et al. (2023, PMID: 36653448), excluding these strains upfront can be the safer approach to avoid potential confounding from dietary sources. However, our study differs in that we collected detailed dietary information, including maternal and infant food frequency questionnaires, which allows us to more effectively contextualize and control for the influence of diet.

This enabled us to include food-associated species such as *Streptococcus thermophilus* to explore their transmission dynamics in greater detail. Notably, *S. thermophilus* exhibited some of the lowest levels of strain-sharing between mothers and infants (Figure 5b), suggesting limited seeding from a common source in early life. Additionally, our phylogenetic generalized linear mixed model (PGLMM) analysis revealed intriguing patterns within *S. thermophilus* strains, distinguishing those likely derived from yogurt from others potentially originating from earlier non-dietary sources.

That said, we acknowledge that the association of *S. thermophilus* strains in relation to diet outside the context of an infant gut is not novel and have now revised the manuscript accordingly. Figure 6 has now been replaced with a different figure regarding transmission from vagina and breastmilk, and the original Figure 6 has been moved to Supplementary figure S9.

11. Much of the strain-sharing analysis appears confirmatory and replicates findings from prior studies, such as PMID: 36653448. While replication is valuable, the novelty of these results should be better highlighted.

Reply: We thank the reviewer for their comment. To increase the novelty of our study we have now included further insights about vertical transmission of strains from vaginal and breast milk in addition to maternal gut. For this purpose, in addition to metagenomically sequencing infant stool, we now include deep metagenomic sequencing of maternal breast milk and vaginal microbiomes. To our knowledge, this is one of the only existing metagenomic studies to carry out strain-level analysis on these maternal body sites in the context of infant seeding and colonization. Our findings indicate that, while there is species-level overlap between breast milk and the infant gut, strain-level transmission is infrequent, suggesting that breast milk is not a major reservoir of dominant gut-colonizing strains in infants.

For the vaginal microbiome, species overlap with the infant gut is even more limited. However, in specific cases, e.g. *Bifidobacterium breve*, we observe 100% strain transmission when the species was present in the maternal vaginal microbiome at delivery (see newly added **Figure 5**). Hence, using this unique dataset, we could conclude that the maternal gut microbiome is a major reservoir of infant gut strains, with occasional transmission from vaginal and breast milk microbiomes.

See new text pertaining to this part below:

"The maternal gut microbiome is the main reservoir for infant gut strains, with occasional transmission from vaginal and breast milk microbiomes

Mother-to-infant sharing of bacterial strains is a hallmark of early-life bacterial transmission[6,41–44] . However, the source and transmission routes from mother are not yet clearly defined. As some of the maternal body-niches have low bacterial biomass (e.g., milk) or very high amounts of human DNA (e.g., vaginal), we needed to sequence especially deep to reach high confidence in our strain annotations. Thus, for this analysis, in addition to our sequenced fecal samples, we generated ultra-deep (~40 GB/sample) metagenomic data for 90 maternal breast milk samples collected during the first month of life and 82 vaginal samples collected close to birth, enabling us to quantify strain-sharing from these additional maternal sources. For each individual species, we defined strain-sharing among two metagenomes using a previously developed approach[45]. To differentiate between the same and different strains, we established genetic distance cut-offs for 1,205 species using longitudinal samples from individuals (see **Table S57** for specific cut-offs per SGB).

When analyzing maternal breast milk samples, we found that *Staphylococcus epidermidis* (SGB7865), *Streptococcus mitis* (SGB8163) and the *Streptococcus salivarius* group (SGB8007) were abundant and prevalent members of the breast milk microbiome (**Table S58**). These species co-occurred in the infant gut (relative abundance >0.01%) in 50.0%, 42.2% and 38.9% of the 90 mother-infant pairs, respectively (**Table S59, Figure S7a**). Among those co-occurring cases, strain-level sharing between breast milk and the infant gut was observed only sporadically (**Table S60**), with strain-sharing of maximally co-occurrent taxa *Staphylococcus epidermidis* (SGB7865) detected in just two families (**Table S60**). Furthermore, while phylogenetic distances between dominant strains of related mother-infant pairs were lower than those between unrelated mother-infant pairs in the majority of the cases (**Figure S7b**), this was only significant for *Bifidobacterium breve* (SGB17247) and *Enterococcus faecalis* (SGB7962) (**Figure S7b**) with these two species showing strain transmission from breast milk to infant gut in two families each (**Table S60**). Hence, although there is some species-level overlap, strain transmission from maternal breast milk to the infant gut appears infrequent, suggesting that breast milk is not a major reservoir for gut-colonizing strains that become dominant in infants.

We observed even fewer species shared between the vaginal microbiome and the infant gut. The vaginal microbiome was dominated by *Lactobacillus crispatus* (SGB7045), *Lactobacillus iners* (SGB7025) and *Gardnerella vaginalis* (SGBs 17302 and 17307), all of which were rarely detected in infant gut samples, corresponding to what was previously observed[7]. (**Table S59** and **Table S61**). The species *Lactobacillus gasseri* (SGB7038 group) co-occurred in 13.4% of mother-infant pairs, with strain transmission observed in a single family (**Figure S7a, Table S59, Table 62**). Intriguingly, in all five mothers in which we could construct dominant strains of *Bifidobacterium breve* (SGB17247) in vagina, the exact same strain was constructed in their infant's gut (**Figure 5 a, b**). Notably, we could construct the same strain in the maternal gut from only one of these five families. Four of these five infants were born via vaginal delivery, and the fifth was born by post-labor CS. Although strains of these species were also detected in infants at later time points (**Figure**

5), these strains differed from those found earlier, strongly suggesting vertical transmission of the vaginal *B. breve* strain during birth."

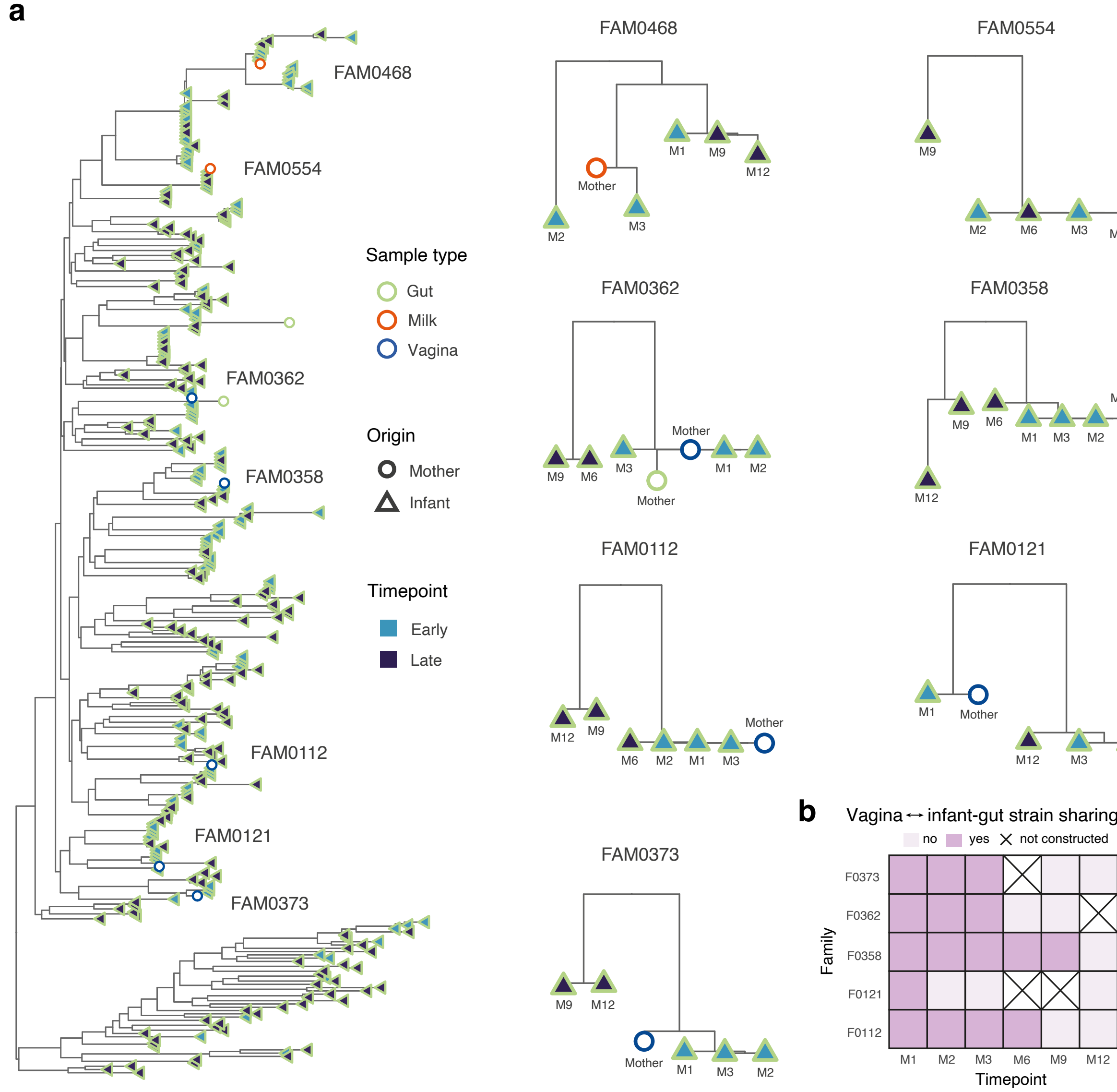

Figure 5. Strain-sharing between maternal breast milk, vaginal and infant gut samples. a, *Maximum likelihood phylogenetic tree of Bifidobacterium breve dominant strains reconstructed from families from which a vaginal sample or breast milk sample was sequenced. Maternal samples are depicted by circles, infant samples by triangles. Colors indicate sample type: green for gut, blue for vagina and orange for breast milk. Expanded insets for all five families are also shown.* ***b****, Heatmap depicting families in which Bifidobacterium breve strains were reconstructed from vaginal samples at infant birth and infant gut over time (M1 to M12). Light color indicates no strain-sharing. Cross indicates that the strain could not be reconstructed from the infant gut at the corresponding time point.*

Going beyond the findings reported in PMID: 36653448, we now not only characterize time-dependent strain-sharing between mothers and infants, we also investigate the functional characteristics of vertically transmitted fecal strains. We observe that mother-infant gut transmitted strains are enriched in genes related to carbohydrate and coenzyme transport and metabolism, suggesting possible functional advantages for successful colonization. The added text now reads:

"Finally, we investigated if bacterial strains transmitted from mothers to infants might be enriched for specific metabolic capacities compared to the strains that were not transmitted. For this, we used a genome-resolved strain-sharing analysis focused on 365 prevalent and/or abundant SGBs detected in mothers and infants. In total, we identified 645 high-quality metagenomic assembled genomes (MAGs), dereplicated at the subspecies level (98% average nucleotide identity (ANI), that could be assigned to 112 of these SGBs (MASH distance < 0.05). We identified 61 MAGs belonging to 29 different SGBs with high nucleotide identity between maternal and infant metagenomes (population ANI ≥ 99.999), a cut-off commonly indicative of transmission in comparisons between whole MAGs (**Figure S8a**). These SGBs were also shown to be transmitted in families in our previous approach using dominant strain marker genes (99.52% concordance).

Functional enrichment analysis revealed 121 Clusters of Orthologous Genes (COGs) that were significantly overrepresented among the transmitted MAGs, based on their presence across a higher number of unique mother-infant pairs than expected by chance (FDR<0.05, one-sided permutation-based test; **Table S70**). On average, enriched COGs were transmitted across 25.4% of MAGs and 44.6% of species in which they were detected (IQR: 15.3–32.1% for MAGs and 32.8–52.3% for species per COG), suggesting that these functions may be broadly relevant to vertical transmission across diverse bacterial taxa (**Figure S8b**). Grouping the enriched COGs into broader functional categories revealed several functions potentially relevant to the early-life gut environment, including *carbohydrate transport and metabolism* (top enriched COGs include COG5498, Endoglucanase Acf2; COG3775, Phosphotransferase system, galactitol-specific IIC component and COG4975, Glucose uptake protein GlcU) and *coenzyme transport and metabolism* (including COG1441, O-succinylbenzoate synthase and COG0854, Pyridoxine 5′-phosphate synthase PdxJ) (**Figure S8c**). To identify the microbial contributors to the transmission of these functions, we estimated the proportion of mother-infant pairs in which each species, or species combination, contributed to the transmission of COGs within these two key functional categories. We found that *Phocaeicola vulgatus* was the predominant contributor, showing functional transmission in the largest proportion of mother-infant pairs, followed by *Bifidobacterium bifidum* and several *Bacteroides* species (**Figure S8d**)."

Lastly, we have now revised our text to highlight the novelty of our maternal-to-infant gut strain transmission analysis. On top of commonly seen trends, such as time-dependent strain-sharing rates between mother and infants and vertical transmission rates per taxa, we have highlighted the novel findings linking bacterial abundance in mothers during pregnancy, and the odds of vertical transmission of the species. The corresponding paragraph now reads:

“Overall, this analysis highlights that a large proportion of strains are transmitted from maternal gut to infant gut, and the frequency of transmission events is positively associated with the abundance of those bacteria in the maternal gut. Strain-sharing was highest at early time points and in vaginally born and breastfed infants, with the transmitted strains enriched in functions potentially relevant to infant development and health”.

Supplementary figures pertaining to this part are shown as follows:

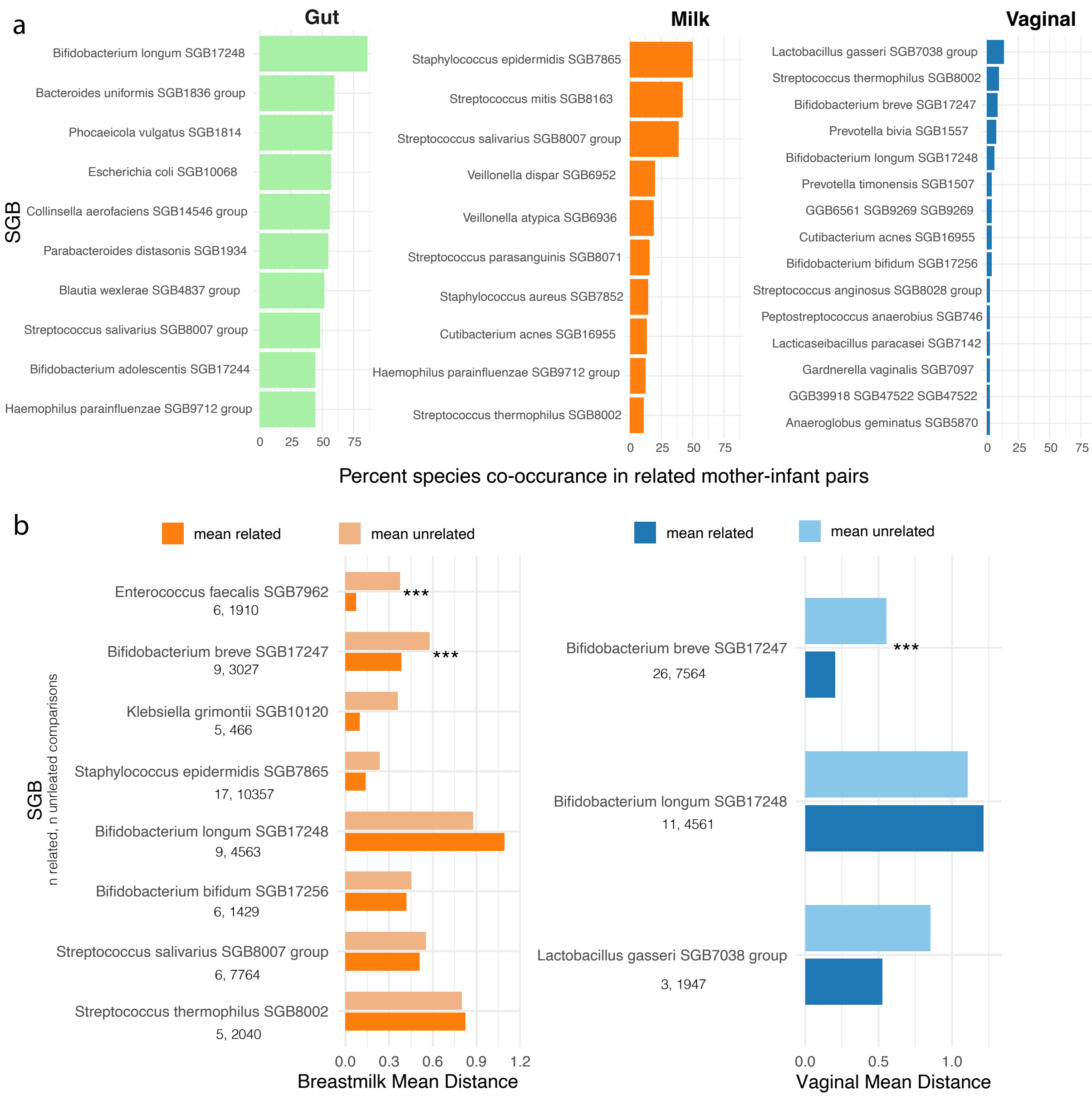

Figure S7. Species co-occurrence between mothers and infants and phylogenetic distances. a, Percentage of species co-occurrence between maternal samples (gut, breast milk or vaginal) and the infant gut in paired mother-infants. Presence was defined as a relative abundance of an SGB >0.01% in both mother and infant. Sample sizes for gut, milk and vaginal pairs were 666, 90 and 82, respectively. The top 10 SGBs for each group are shown. ***b,*** *Mean phylogenetic distances between related and unrelated mother-*

infant pairs between breast milk and infant gut (left in light and dark orange) and vagina and infant gut (right in light and dark blue). The number of related versus unrelated comparisons are shown in the numbers below the SGB name. P-values were calculated using a permutation-based test. *** P-value < 0.05.

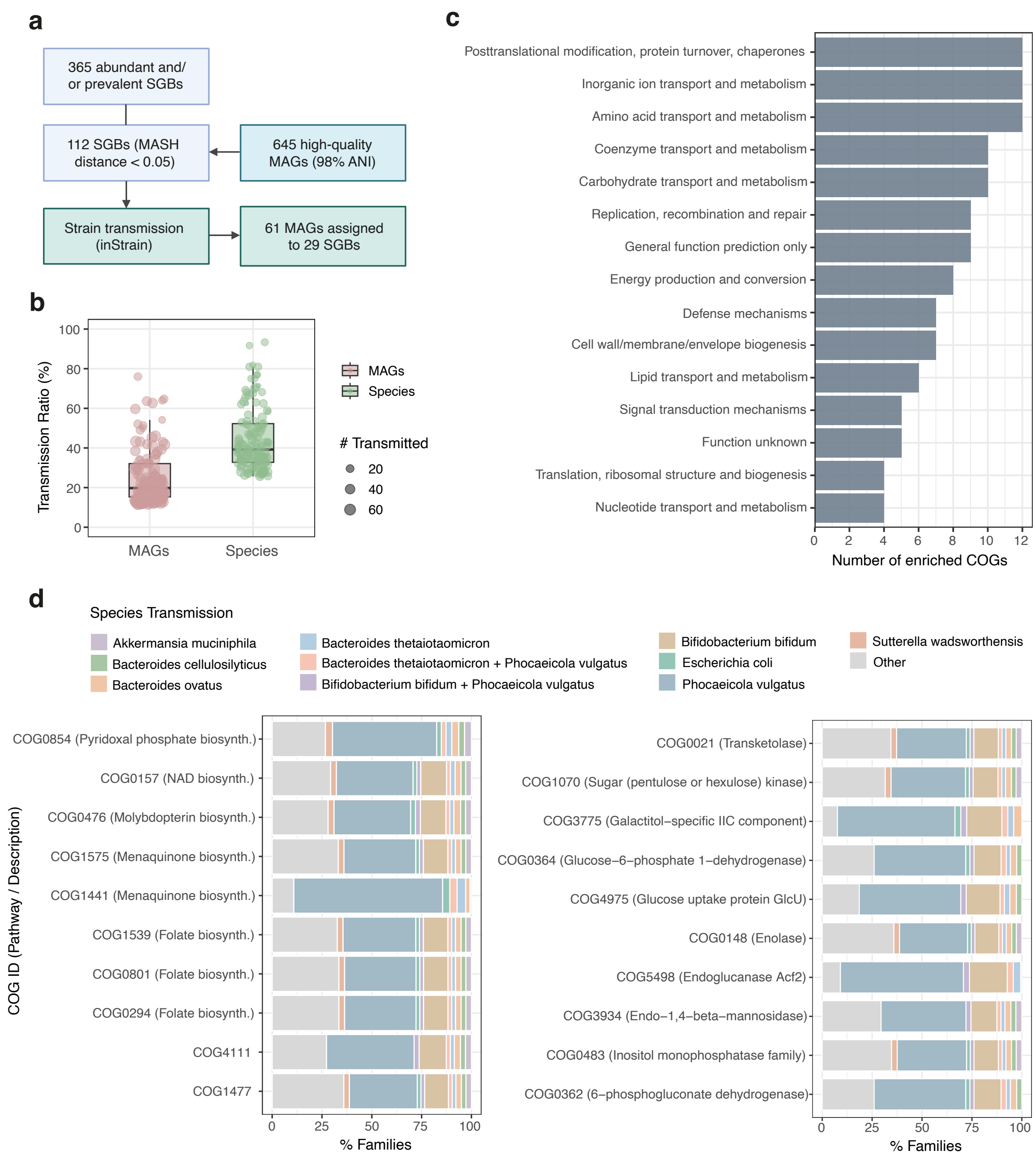

***Figure S8. Functional enrichment of transmitted bacterial strains. a**, Schematic overview of genome-resolved strain-sharing analysis. Starting from 365 prevalent and/or abundant SGBs detected in mothers and infants, 645 high-quality MAGs were identified (dereplicated at 98% ANI), corresponding to 112 SGBs (MASH distance < 0.05). Strain transmission was detected for 61 MAGs assigned to 29 SGBs based on high nucleotide identity (population ANI >99.999) between maternal and infant metagenomes. **b**, Boxplots comparing transmission ratios of individual COGs across MAGs (red) and species (green). Each dot represents a single COG. The Y-axis shows the transmission ratio, calculated as the number of MAGs (or*

species) that encode the COG and were transmitted, divided by the total number of MAGs (or species) that encode the COG. The size of each dot reflects the number of transmitted MAGs or species in which the COG was detected. ***c****, Bar plot of the number of significantly enriched COGs grouped by COG functional categories (top 15 categories are shown).* ***d****, Species-level contributions to the transmission of enriched COGs within the Coenzyme Transport and Metabolism (left) and Carbohydrate Transport and Metabolism (right) categories. Stacked bars show the percentage of mother-infant pairs in which each species or species combination contributed to COG transmission. Top contributors are highlighted; others are grouped as "Other."*

12. Lines 569-570:
"We then investigated the relationship between infant and maternal factors and species phylogenies, adjusted for sampling time."
• Adjusting for sampling time may not be the most appropriate approach given the repeated sampling design. Including time as a random effect would better account for within-subject correlations.

Reply: We apologize for the unclear description of the PGLMM approach. This question was also raised by another reviewer. We would like to clarify that in this analysis only one (earliest) strain per species per individual is included because extensive testing showed that addition of multiple timepoints per individual causes poor PGLMM performance.

The details of our extensive testing are as follows: In the initial phase of testing the PGLMM approaches, we observed that inclusion of several strains (i.e. several timepoints) per individual, and therefore inclusion of two random factors (variance-covariance matrix of strain similarity and infant ID), caused very poor PGLMM performance. Both 'mcmcGLMM', with its Metropolis-Hastings algorithm, and the Hamiltonian MC algorithm implemented in the 'brms' package do not show good chain convergence for most species because there are too many model parameters to optimize

In the timepoint-phylogeny association analysis, given the fact that each individual was included only once with its earliest occurrence of the strain, the actual separation of "early strains" and "late strains" is a separation of individuals who acquired a bacterial species at earlier timepoints from individuals who acquired it later in time. The within-subject correlation problem is thus absent in this analysis because each individual is included only once. However, this approach doesn't allow us to study strain dynamics within infants. We are aware of this limitation and discuss the results only in the context of what the model actually tests for, e.g., the comparison of "early colonization" and "late colonization".

In the phenotype-phylogeny association analysis, we were required to avoid time-dependent spurious associations where both phylogeny and phenotype are time-dependent. Inclusion of time as a fixed factor efficiently solves this problem. Similar to the time-phylogeny associations, we were operating single, the earliest, strain per individual due to the same problem: poor MCMC convergence in case of too many parameters to estimate, if multiple timepoints of the same individual included, and random factor of individual IDs is necessary. This analysis was coupled with PGLMM analysis on each timepoint individually, as illustrated in **Figures S9b** and **S9g**. In both

the single-timepoint and multiple-timepoint PGLMM analysis, within-subject correlation is not an issue due to the absence of multiple strains per individual.

We have now updated the text accordingly to clarify these points:

Methods

"For each species, only one strain per individual—the earliest detected—was included in the analysis, since including multiple strains per individual required treating Individual IDs as an additional random factor, which, in combination with strain variance-covariance matrix as another random factor, leads to too many parameters to optimize during the MCMC process."

Referee #2 (Remarks to the Author):
Referee #2: human microbiome, multi-omics

The study by Sinha, Brushett et al. investigates the dynamics of gut microbiomes of mothers and infants during pregnancy and early life in relation to their effects on health. The authors analysed a very comprehensive sample set comprised of 4,577 fecal samples from 714 mother-infant pairs in the Dutch LLNEXT cohort, during pregnancy to one year postpartum. Key findings include:

1. The maternal gut microbiome changes subtly during pregnancy and postpartum, influenced by diet, infections, and smoking history.
2. Significant mother-to-infant microbial strain transmission occurs, with higher maternal abundances increasing transmission likelihood.
3. Maternal gut diversity before birth is a strong predictor of infant eczema.
4. Infant gut microbiome is shaped by delivery mode and feeding, with other factors like maternal smoking and parity affecting specific species.
5. Early (2 weeks) infant microbiome composition does not predict later health outcomes.

The study by Sinha, Brushett et al. represents the most extensive study of the pregnancy and early life microbiome to date. The impact of microbiome impacts during critical stages of human life remains an important research topic. Hence, the present study is timely as it provides insights for potential future microbiome-targeted therapies. Overall, the analyses performed of the very extensive dataset result in new insights and involve state-of-the-art methods. The manuscript is well written and all figures are clear.

Reply: we thank the reviewer for their positive comments on our paper.

Major comments

1. The authors should provide a clear rationale for not having obtained infant samples from timepoints prior to two weeks postpartum (this is the earliest timepoint considered). Earlier work has in particular highlighted differences between infants born vaginally and those delivered by caesarian section. If possible, the authors should seek to analyse data from earlier timepoints or at least contextualise their findings, e.g. with respect to Bacteroides spp., in relation to these earlier studies.

Reply: We thank the reviewer for this comment. We would like to confirm that we did in fact collect meconium samples from most of the infants included in this study. However, despite our efforts, all test meconium samples failed to yield sufficient quality for genomic library preparation and subsequent successful sequencing in our pilot study (Garmaeve and Sinha et al. PMID: 38431663) This outcome has now been noted in our Methods:

"Meconium was also collected from infants, but the very low microbial biomass in these samples meant our attempts to isolate viable microbial reads were unsuccessful, as previously reported[43]. Therefore, these samples were not included in the current study."

Furthermore, regarding sample collection at 2 weeks of age, we would like to clarify the timeline of sample collection across the first months of life. Samples from parents were indeed requested around 2 weeks postpartum. In reality, these spanned from day 3 to day 22 (after first meconium had passed). For clarity in our graphical representations, categorical age labels (e.g., "2 weeks") are used. However, all statistical analyses were performed using continuous age. This approach allows us to maintain analytical precision while ensuring that visual representations are easily interpretable.

We agree with the reviewer that delivery mode (vaginal vs cesarean section) has the strongest and long-lasting effect on the infant gut microbiome. We took into consideration the adjustment for delivery mode (as well as feeding mode as the second strongest phenotype) in all analyses in this paper. Moreover, the specific analysis of *Bacteroides* abundances was performed only in vaginally born babies. According to reviewer's request, this part has been further elaborated in the text and an important reference to previous work has been added:

"Characteristics of a complicated labor are associated with a decreased abundance of *Bacteroides* in vaginal deliveries

Having one of the largest cohorts of vaginally delivered infants (n=585, 84.8%, **Table S4**), including a significant number of home births (n=155, 22.5%, **Table S4**), we aimed to further investigate the relationship between delivery-related predictors (**Table S1**) and the gut microbiome of vaginally delivered infants. Consistent with previous findings[6–9], infants born via CS showed a depletion of the *Bacteroides* genus until M3, whereas vaginally delivered infants exhibited a bimodal distribution of this genus, with this difference (between CS and vaginal delivery) persisting until M12 (**Figure 3a**). We first investigated what determines *Bacteroides* colonization in vaginally born infants. We observed a lower *Bacteroides* abundance in infants born vaginally at the hospital versus at home (specifically *B. uniformis* (SGB1836 group), *P*=0.002 and *B. xylanisolvens* (SGB1867), *P*=0.006; **Figure 3b**, **Table S33**). In addition, prolonged labor, maternal use of painkillers and anesthetics and a longer duration of ruptured membranes were associated with a decreased abundance of *B. uniformis* (SGB1836), *Parabacteroides distasonis* (SGB1934), *Phocaeicola dorei* (SGB181), *Bacteroides ovatus* (SGB1871), *Bacteroides stercoris* (SGB1830), *Bacteroides thetaiotaomicron* (SGB1861) and *Bacteroides fragilis* (SGB1855) (**Figure 3b, Table S33**). These findings suggest that variations in *Bacteroides* abundance in vaginally delivered infants are likely associated with a combination of multiple factors, rather than a single phenotypic determinant, although all these factors are characteristic of a long-complicated labor."

2. The authors' analyses are very focused on taxonomically resolved microbiome data including diversity metrics. As such, the analyses are lacking with respect to detailed functional and pathway-level analyses. Earlier work has for example highlighted functional microbiome differences related to delivery mode in relation to important immunomodulatory molecules such as LPS (e.g. PMIDs: 27133167 & 30504906) or vitamins (PMID: 35295713). The authors should perform more precise function-directed analyses and relate this data to the extensive set of clinical, dietary and exposure variables.

Reply: We agree with the reviewer. This comment was also raised by another reviewer. To enhance functional insights, we have undertaken the following steps:

Given the relatively limited capacity of the infant digestive enzyme repertoire and their high reliance on microbial carbohydrate metabolism, we performed comprehensive functional annotation of the **Carbohydrate-Active Enzyme (CAZyme) profile** of the infant gut microbiome. This revealed dynamic changes throughout the first year of life. In contrast to adults, the infant CAZyme landscape was predominantly driven by the presence of *Bacteroides* species, with delivery mode playing a major role by influencing *Bacteroides* abundance. Further substrate-specific analysis showed that infants delivered vaginally were enriched for CAZymes involved in non-starch polysaccharide metabolism and those receiving breast feeding with CAZymes involved in resistant oligosaccharides, mucin and human milk glycans. Prior to solid food introduction, infants showed a significant enrichment of CAZymes involved in the metabolism of resistant oligosaccharides and starch, peptidoglycan, amylose, pullulan and amylopectin. In contrast, later timepoints were significantly enriched in CAZymes associated with glycoprotein, pectin and arabinogalactan metabolism. Finally, we observed significant associations between specific CAZyme subfamilies and infant growth trajectories.

We further conducted deeper investigations into **bacterial functional pathways using MetaCyc**, which revealed associations not only with delivery mode and infant feeding but also with parity, infant stool frequency and, notably, maternal dietary patterns. These findings reinforce the impact of maternal nutrition on the infant microbiome.

Lastly, a new analysis of **gut–brain modules** revealed compelling associations between infant feeding and pathways related to acetate synthesis, with formula feeding associated with lower acetate synthesis, potentially linking infant nutrition with microbial production of neuroactive metabolites.

These new results have been incorporated into the text (see below) and are shown in amended main **Figure 3** and new supplementary figures **S4, S5 and S6**.

With regards to the analysis of CAZymes we added the following text:

“Infant CAZyme profile is highly dynamic during the first year of life, shaped by the presence of *Bacteroides*, *Parabacteroides* and *Phocaeicola*

Given the relatively limited capacity of the infant digestive enzyme repertoire and their high reliance on microbial carbohydrate metabolism, we annotated the carbohydrate-active enzyme (CAZyme) profiles of the infant and maternal gut microbiomes to investigate their dynamics and association with exposures. Similar to microbiome composition, the maternal CAZyme profile remained stable during pregnancy, while the infant CAZyme profile was highly dynamic throughout the first year of life (**Figure S5b** and **c; Table S41**). In the infant gut, *Bacteroides*, *Parabacteroides* and *Phocaeicola* were the main contributors to CAZyme profile variation (**Figure 3e, Table S42**), whereas *Bacteroides* played a less prominent role in the maternal gut (**Figure S5d, Table S43**). Most CAZyme associations were linked to mode of delivery, driven primarily by differences in the presence or absence of genus *Bacteroides*, *Parabacteroides* and

Phocaeicola between the groups (**Figure S6**). Interestingly, after investigate associations with infant-specific traits (**Table S44** and **Table S45**) we also found significant associations of infant weight with CAZyme subfamilies even after correction for delivery and feeding mode, with the Glycoside Hydrolase Family 89 (GH89) being associated with infant weight, length and head circumference (FDR=7.12×10^{-6} , 4.95×10^{-4} and 4.74×10^{-4}, respectively; **Table S45**). Further annotation of CAZyme substrates revealed that infants delivered vaginally were enriched for CAZymes involved in non-starch polysaccharide metabolism (NSP), whereas those delivered by CS showed enrichment for starch-metabolizing CAZymes (overrepresentation analysis, FDR=0.002 and 0.008, respectively; **Table S46**). These findings highlight the substantial role of delivery mode in shaping the functional potential of the infant gut microbiome.

In accordance with expectations, breastfeeding was associated with an enrichment of CAZymes related to the metabolization of resistant oligosaccharides, mucin and human milk glycans compared to formula feeding (FDR=0.013, 0.001, 0.000 respectively; **Table S47**). Compared to later time points, earlier time points showed a significant enrichment of CAZymes involved in the metabolism of resistant oligosaccharides and starch, peptidoglycan, amylose, pullulan and amylopectin (**Figure 3f, Table S48**). In contrast, later time points were significantly enriched in CAZymes associated with glycoprotein, pectin and arabinogalactan metabolism (**Figure 3f, Table S48**). This pattern reflects the rapid and dynamic development of the gut microbiome's functional capacity to metabolize diverse substrates during early infancy."

With regards to bacterial pathways, we added the following text:

"When investigating the relationships between individual bacterial species of the infant gut microbiome and infant-specific predictors, we identified 425 significant associations. However, the majority of these were related to delivery and feeding mode, and 193 associations remained after correction for these variables (**Tables S27** and **S28**). Associations were mostly linked to parity, infant stool consistency, frequency and diet (**Tables S27** and **S28**). Interestingly, we observed few associations with some of the unique predictors captured through our detailed questionnaires. For example, only single associations were found for sleeping and no FDR-significant associations were found with crying time (**Tables S27** and **S28**).

We observed 280 bacterial metabolic pathways of the infant gut microbiome significantly associated with 51 variables, with the highest numbers linked to feeding mode (238), parity and gravidity (169), infant stool frequency (162) and delivery-related factors (84), together mirroring the associations observed with bacterial species (**Tables S29** and **S30**). Some pathway associations were mainly driven by a single bacterial species, e.g. the negative association of PWY-5845 (menaquinol-9 biosynthesis) to infant fish and meat consumption was mainly driven by a decrease in abundance of *E. coli* genes belonging to this pathway. For other pathways, the association of pathway to predictors was driven by multiple bacteria. For example, the positive association of PWY-7013 (S-propane-1,2-diol degradation) to formula feeding was driven by the combined pathway-contributing genes of several *Klebsiella* species, *Salmonella* enterica, *Enterobacter cloacae*, *E. coli* and other species. Similarly, the positive association of LACTOSECAT-

PWY (lactose and galactose degradation I) with formula feeding is driven by the pathway genes of multiple *Enterococcus* and *Streptococcus* species (**Figure S4**). After adjusting for delivery and feeding mode, we identified parity, gravidity and stool frequency to be associated with the majority of infant pathways. Additionally, infant complementary feeding was associated with numerous pathways, including 22 linked to bread intake (primarily metabolic pathways) and 12 related to meat/fish intake. Intriguingly, maternal dietary patterns were also associated with 36 infant microbial pathways, reinforcing the potential impact of maternal nutrition on the developing infant microbiome (**Table S29**)."

With regards to infant microbial neuroactive potential, we added the following text:
"We next examined associations between the estimated abundance of gut–brain modules (GBMs)[27] and infant-specific predictors. We identified 125 FDR-significant associations, the majority of which were related to infant feeding mode (**Tables S31** and **S32**). Among the most significant findings, we observed a decrease in the quinolinic acid degradation pathway in formula-fed infants, while the acetate synthesis I pathway was significantly enriched in breastfed infants (**Figure 3d**). These results highlight potential differences in microbial neuroactive metabolism linked to early-life nutrition."

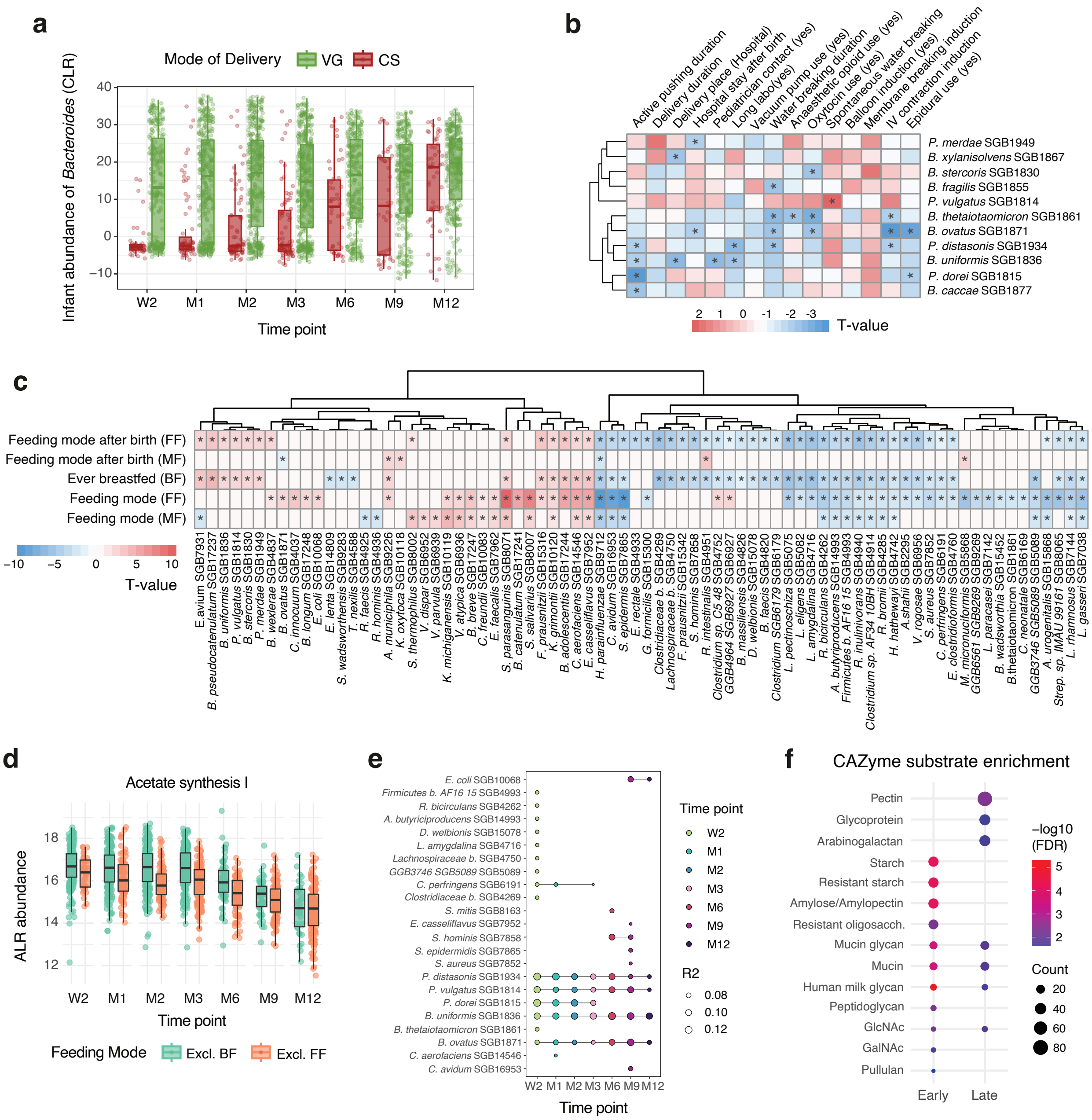

Figure 3. Associations of infant gut microbiome and CAZyme profile with predictors. a, Boxplots of the association between the centered log ratio (CLR)-transformed relative abundance of Bacteroides and delivery mode across different time points. ***b***, Heatmap of T-values for the association between birth-related factors and various Bacteroides species. * P-value < 0.05. ***c***, Heatmap of T-values for associations between feeding mode at birth, feeding mode from 2 weeks to 12 months of age and ever being breastfed or not with bacterial species at species-level genomic bin (SGB) level. * FDR < 0.05. ***d***, *Boxplots of the additive log ratio (ALR)-transformed abundance of acetate synthesis with either exclusive breastfeeding or formula feeding over time.* ***e***, *PERMANOVA of CLR-transformed SGB abundance on the Aitchison's distance matrix of the entire CAZyme profile of infants at seven time points. Only significant bacteria (permutation P-value <0.0001 and R^2 >0.06) are shown. For full results see* ***Table S42***. ***f***, *Overrepresentation analysis of CAZyme substrates at early (3 months or younger) versus late (6 months or older) time point of infants. Only FDR-significant (<0.05) results are shown. For* ***a*** *and* ***d***, *each boxplot visualizes the median, hinges (25th and 75th percentiles) and whiskers extending up to 1.5 times the interquartile range from the hinges.*

New supplementary figures pertaining to this part are as follows:

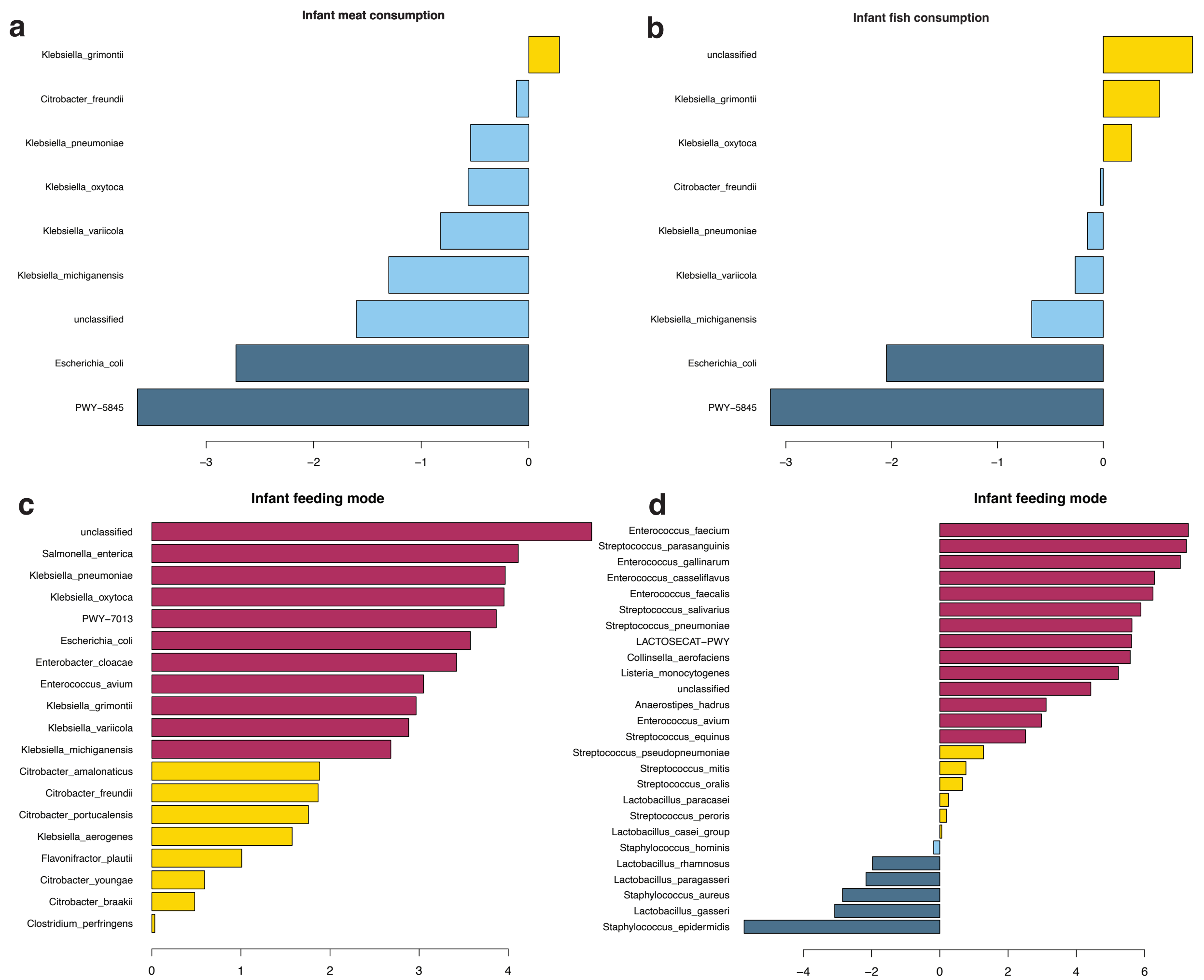

Figure S4. Per-species pathway abundance association to phenotypes*. X-axis denotes T-statistics of association of total pathway abundance (encoded with METACYC ID). Species names represent the association T-values of total abundance of genes belonging to a pathway from individual species. The "Unclassified" category represents association of pathway genes with no taxonomic binning.*

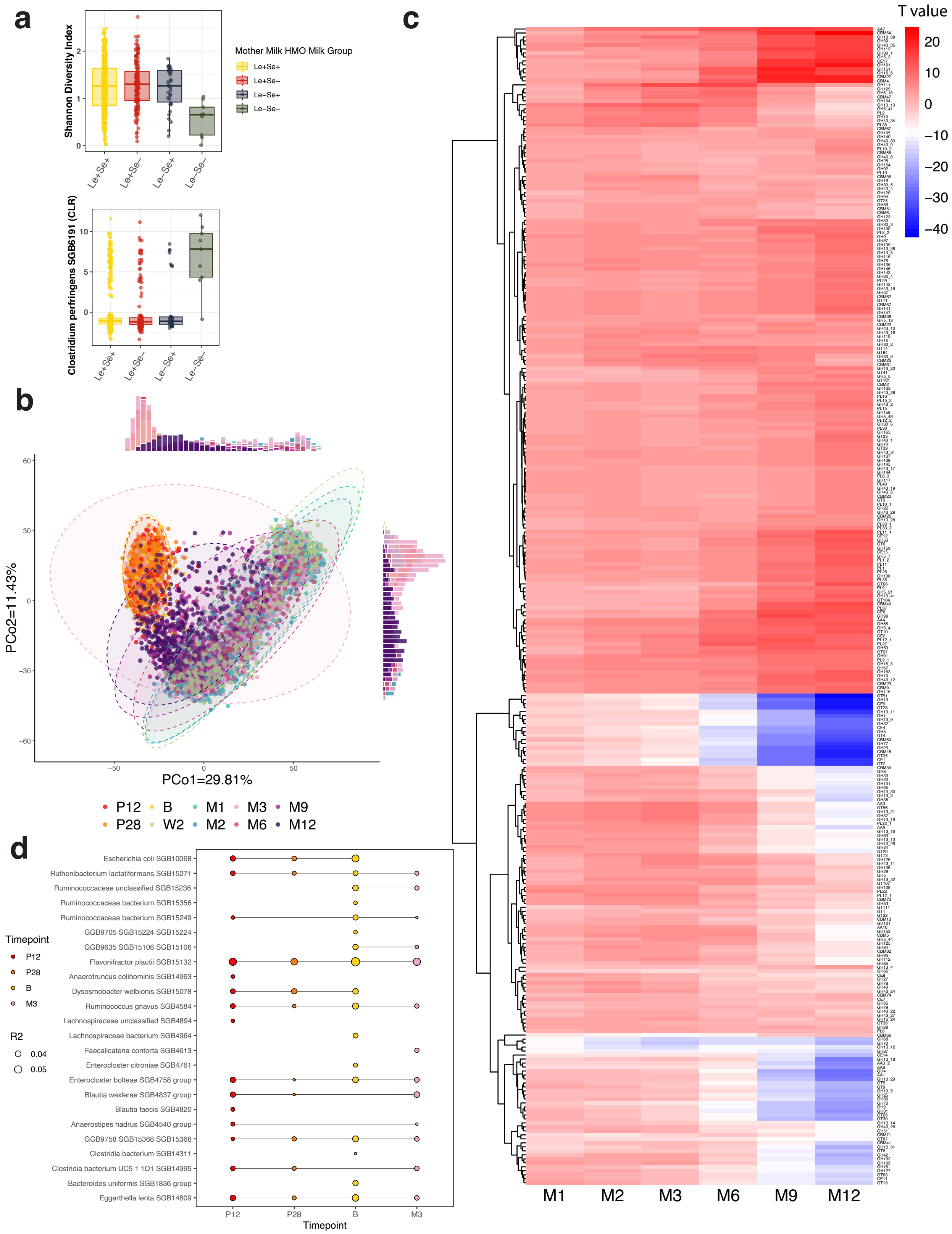

Figure S5. Maternal milk group and HMO associations and maternal and infant CAZyme profile. a, *Maternal secreter status and infant Shannon diversity index (above) and infant relative abundance of*

Clostridium perfringens (CLR-transformed) (below). ***b,*** *PCoA analysis based on Aitchison distance calculated at the subfamily level of maternal and infant CAZyme profile.* ***c,*** *T-values of the association of infant time point with CAZyme subfamilies using W2 as the reference.* ***d,*** *PERMANOVA of CLR-transformed SGB abundance on the Aitchison's distance matrix of the entire CAZyme profile of mothers at various time points.*

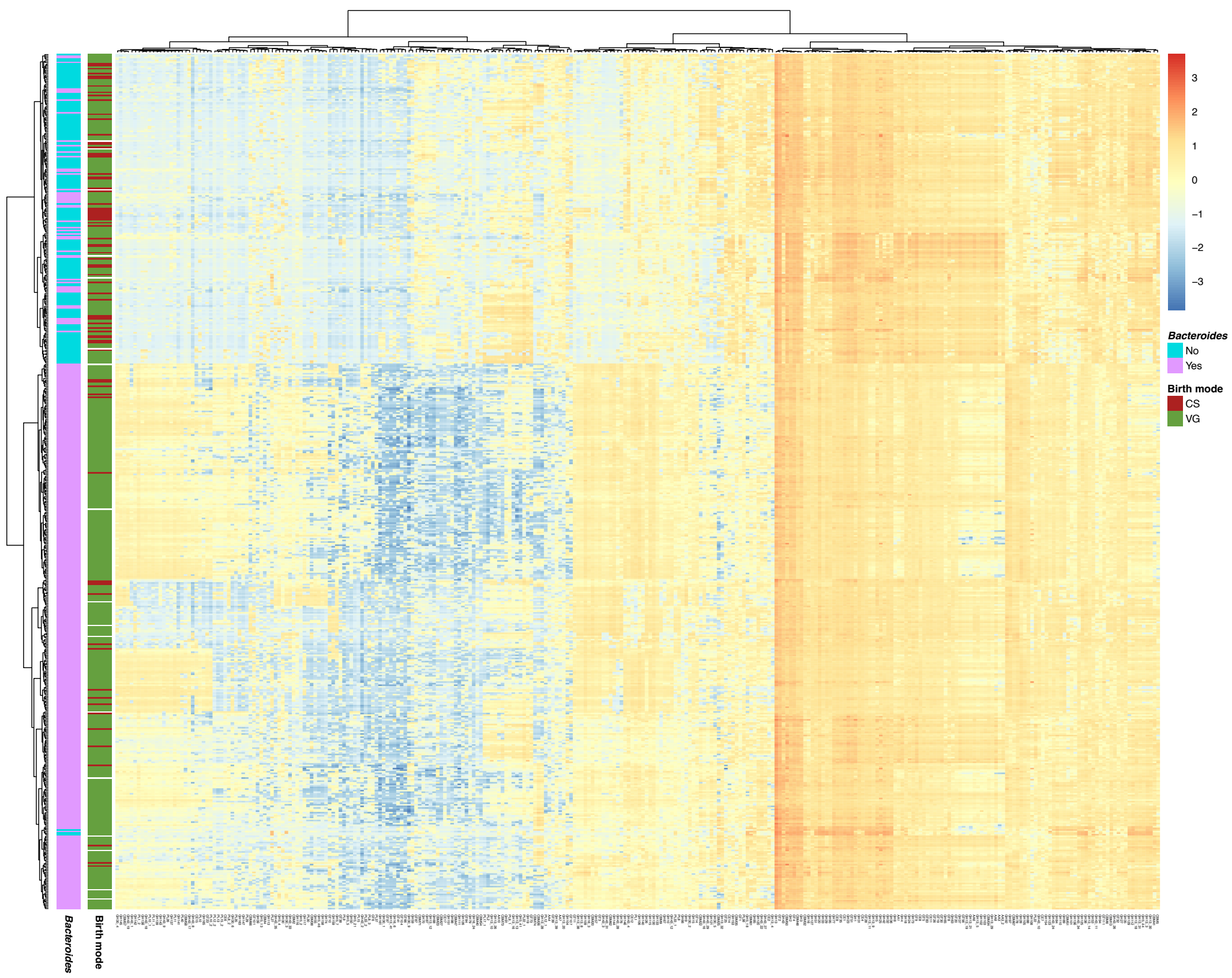

Figure S6. Bacteroides is the major driver of infant CAZyme profile*. Heatmap of the infant CAZyme profile at month 3 (M3), with the X-axis representing CAZyme subfamilies. The Y-axis includes bar plots indicating the presence or absence of Bacteroides and the mode of delivery (vaginal (VG) vs. cesarean section (CS)).*

3. With respect to the strain-tracking analyses relating the mothers' and infants' microbiomes, it would be insightful to understand which gut or other reservoirs the shared strains (Bacteroides spp., Bifidobacterium spp., Streptococcus spp. etc.) come from. This is in particular important to understand the routes of transfer from mothers to infants in view of the development of future interventions. Additional sample sets might exist which the authors could leverage to ascertain the origins of specific strains.

Reply: We thank the reviewer for their comment. Following this suggestion, we have now generated additional data to better understand the routes of transfer from mothers to infants in view of the development of future intervention. For this purpose, in addition

to metagenomically sequencing infant stool, we have now included deep metagenomic sequencing (40GB/sample) of the maternal breast milk and vaginal microbiomes. To our knowledge, ours is one of the only metagenomic studies to carry out strain-level analysis on these maternal body sites in the context of infant seeding and colonization. Our findings indicate that, while there is species-level overlap between breast milk and the infant gut (see newly added **supplementary figure S7**), strain-level transmission is infrequent, suggesting that breast milk is not a major reservoir of dominant gut-colonizing strains in infants.

For the vaginal microbiome, species overlap with the infant gut is even more limited. However, in specific cases, e.g. *Bifidobacterium breve*, we observed 100% strain transmission when the species was present in the maternal vaginal microbiome at delivery (see newly added **Figure 5**). Hence, using this unique dataset, we can conclude that the maternal gut microbiome is a major reservoir of infant gut strains, with occasional transmission from vaginal and breast milk microbiomes.

See new text pertaining to this part below:

"The maternal gut microbiome is the main reservoir for infant gut strains, with occasional transmission from vaginal and breast milk microbiomes

Mother-to-infant sharing of bacterial strains is a hallmark of early-life bacterial transmission[6,41–44] . However, the source and transmission routes from mother are not yet clearly defined. As some of the maternal body-niches have low bacterial biomass (e.g., milk) or very high amounts of human DNA (e.g., vaginal), we needed to sequence especially deep to reach high confidence in our strain annotations. Thus, for this analysis, in addition to our sequenced fecal samples, we generated ultra-deep (~40 GB/sample) metagenomic data for 90 maternal breast milk samples collected during the first month of life and 82 vaginal samples collected close to birth, enabling us to quantify strain-sharing from these additional maternal sources. For each individual species, we defined strain-sharing among two metagenomes using a previously developed approach[45]. To differentiate between the same and different strains, we established genetic distance cut-offs for 1,205 species using longitudinal samples from individuals (see **Table S57** for specific cut-offs per SGB).

When analyzing maternal breast milk samples, we found that *Staphylococcus epidermidis* (SGB7865), *Streptococcus mitis* (SGB8163) and the *Streptococcus salivarius* group (SGB8007) were abundant and prevalent members of the breast milk microbiome (**Table S58**). These species co-occurred in the infant gut (relative abundance >0.01%) in 50.0%, 42.2% and 38.9% of the 90 mother-infant pairs, respectively (**Table S59, Figure S7a**). Among those co-occurring cases, strain-level sharing between breast milk and the infant gut was observed only sporadically (**Table S60**), with strain-sharing of maximally co-occurrent taxa *Staphylococcus epidermidis* (SGB7865) detected in just two families (**Table S60**). Furthermore, while phylogenetic distances between dominant strains of related mother-infant pairs were lower than those between unrelated mother-infant pairs in the majority of the cases (**Figure S7b**), this was only significant for *Bifidobacterium breve* (SGB17247) and *Enterococcus faecalis* (SGB7962) (**Figure S7b**) with these two species showing strain transmission from breast milk to infant gut in two families each

(**Table S60**). Hence, although there is some species-level overlap, strain transmission from maternal breast milk to the infant gut appears infrequent, suggesting that breast milk is not a major reservoir for gut-colonizing strains that become dominant in infants.

We observed even fewer species shared between the vaginal microbiome and the infant gut. The vaginal microbiome was dominated by *Lactobacillus crispatus* (SGB7045), *Lactobacillus iners* (SGB7025) and *Gardnerella vaginalis* (SGBs 17302 and 17307), all of which were rarely detected in infant gut samples, corresponding to what was previously observed[7]. (**Table S59** and **Table S61**). The species *Lactobacillus gasseri* (SGB7038 group) co-occurred in 13.4% of mother-infant pairs, with strain transmission observed in a single family (**Figure S7a, Table S59, Table 62**). Intriguingly, in all five mothers in which we could construct dominant strains of *Bifidobacterium breve* (SGB17247) in vagina, the exact same strain was constructed in their infant's gut (**Figure 5 a, b**). Notably, we could construct the same strain in the maternal gut from only one of these five families. Four of these five infants were born via vaginal delivery, and the fifth was born by post-labor CS. Although strains of these species were also detected in infants at later time points (**Figure 5**), these strains differed from those found earlier, strongly suggesting vertical transmission of the vaginal *B. breve* strain during birth."

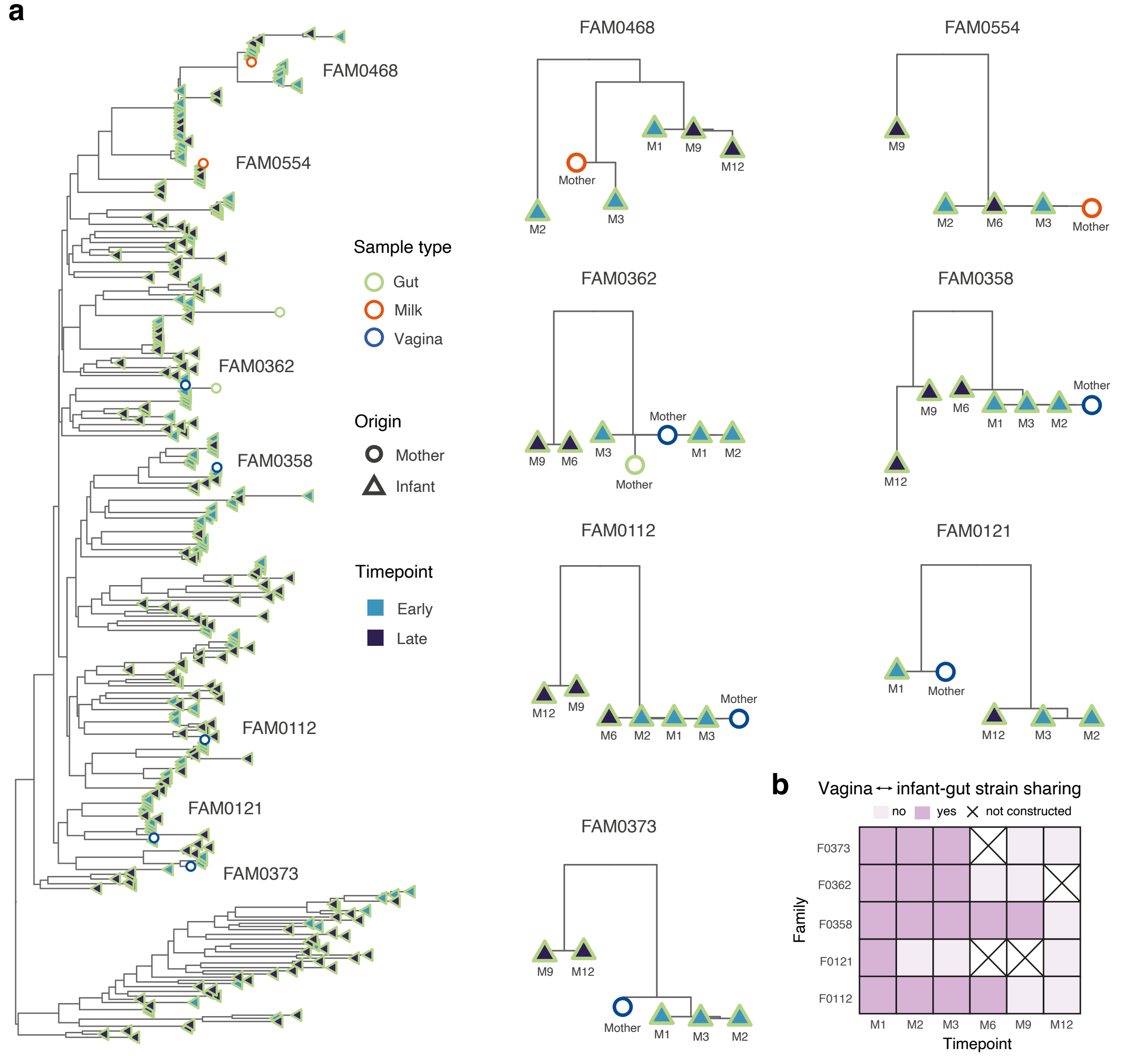

Figure 5. Strain-sharing between maternal breast milk, vaginal and infant gut samples. a, *Maximum likelihood phylogenetic tree of Bifidobacterium breve dominant strains reconstructed from families from which a vaginal sample or breast milk sample was sequenced. Maternal samples are depicted by circles, infant samples by triangles. Colors indicate sample type: green for gut, blue for vagina and orange for breast milk. Expanded insets for all five families are also shown.* ***b****, Heatmap depicting families in which Bifidobacterium breve strains were reconstructed from vaginal samples at infant birth and infant gut over time (M1 to M12). Light color indicates no strain-sharing. Cross indicates that the strain could not be reconstructed from the infant gut at the corresponding time point.*

Going beyond the findings reported in PMID: 36653448, we now not only characterize time-dependent strain-sharing between mothers and infants, we also investigate the functional characteristics of vertically transmitted fecal strains. We observe that mother-infant gut transmitted strains are enriched in genes related to carbohydrate and coenzyme transport and metabolism, suggesting possible functional advantages for successful colonization. The added text now reads:

"Finally, we investigated if bacterial strains transmitted from mothers to infants might be enriched for specific metabolic capacities compared to the strains that were not transmitted. For this, we used a genome-resolved strain-sharing analysis focused on 365 prevalent and/or abundant SGBs detected in mothers and infants. In total, we identified 645 high-quality metagenomic assembled genomes (MAGs), dereplicated at the subspecies level (98% average nucleotide identity (ANI), that could be assigned to 112 of these SGBs (MASH distance < 0.05). We identified 61 MAGs belonging to 29 different SGBs with high nucleotide identity between maternal and infant metagenomes (population ANI ≥ 99.999), a cut-off commonly indicative of transmission in comparisons between whole MAGs (**Figure S8a**). These SGBs were also shown to be transmitted in families in our previous approach using dominant strain marker genes (99.52% concordance).

Functional enrichment analysis revealed 121 Clusters of Orthologous Genes (COGs) that were significantly overrepresented among the transmitted MAGs, based on their presence across a higher number of unique mother-infant pairs than expected by chance (FDR<0.05, one-sided permutation-based test; **Table S70**). On average, enriched COGs were transmitted across 25.4% of MAGs and 44.6% of species in which they were detected (IQR: 15.3–32.1% for MAGs and 32.8–52.3% for species per COG), suggesting that these functions may be broadly relevant to vertical transmission across diverse bacterial taxa (**Figure S8b**). Grouping the enriched COGs into broader functional categories revealed several functions potentially relevant to the early-life gut environment, including *carbohydrate transport and metabolism* (top enriched COGs include COG5498, Endoglucanase Acf2; COG3775, Phosphotransferase system, galactitol-specific IIC component and COG4975, Glucose uptake protein GlcU) and *coenzyme transport and metabolism* (including COG1441, O-succinylbenzoate synthase and COG0854, Pyridoxine 5′-phosphate synthase PdxJ) (**Figure S8c**). To identify the microbial contributors to the transmission of these functions, we estimated the proportion of mother-infant pairs in which each species, or species combination, contributed to the transmission of COGs within these two key functional categories. We found that *Phocaeicola vulgatus* was the predominant contributor, showing functional transmission in the largest proportion of mother-infant pairs, followed by *Bifidobacterium bifidum* and several *Bacteroides* species (**Figure S8d**)."

Lastly, we have now revised our text to highlight the novelty of our maternal-to-infant gut strain transmission analysis. On top of commonly seen trends, such as time-dependent strain-sharing rates between mother and infants and vertical transmission rates per taxa, we have highlighted the novel findings linking bacterial abundance in mothers

during pregnancy, and the odds of vertical transmission of the species. The corresponding paragraph now reads:

“Overall, this analysis highlights that a large proportion of strains are transmitted from maternal gut to infant gut, and the frequency of transmission events is positively associated with the abundance of those bacteria in the maternal gut. Strain-sharing was highest at early time points and in vaginally born and breastfed infants, with the transmitted strains enriched in functions potentially relevant to infant development and health”.

Supplementary figures pertaining to this part are shown as follows:

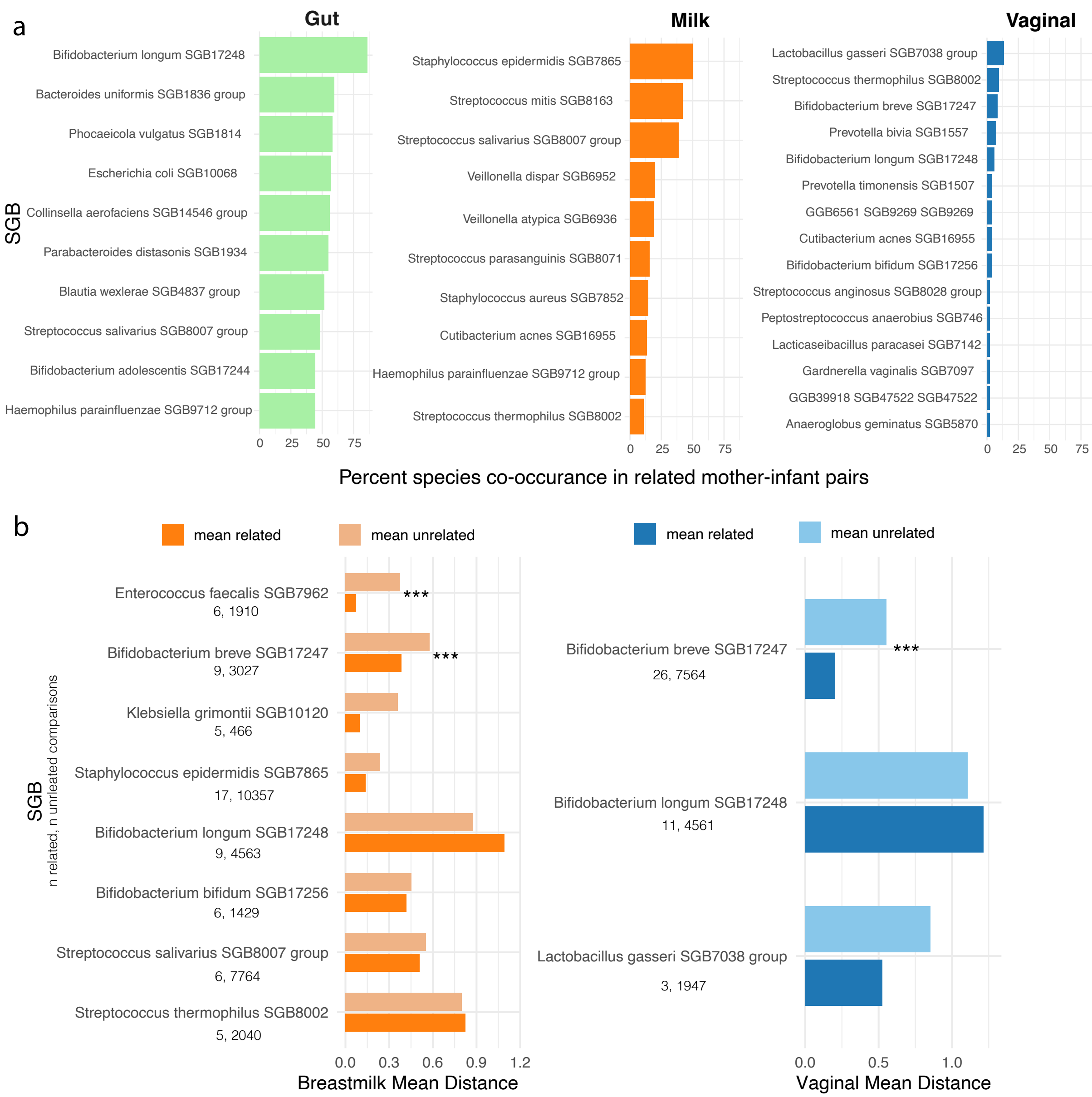

Figure S7. Species co-occurrence between mothers and infants and phylogenetic distances. a, *Percentage of species co-occurrence between maternal samples (gut, breast milk or vaginal) and the infant*

gut in paired mother-infants. Presence was defined as a relative abundance of an SGB >0.01% in both mother and infant. Sample sizes for gut, milk and vaginal pairs were 666, 90 and 82, respectively. The top 10 SGBs for each group are shown. **b,** Mean phylogenetic distances between related and unrelated mother-infant pairs between breast milk and infant gut (left in light and dark orange) and vagina and infant gut (right in light and dark blue). The number of related versus unrelated comparisons are shown in the numbers below the SGB name. P-values were calculated using a permutation-based test. *** P-value < 0.05.

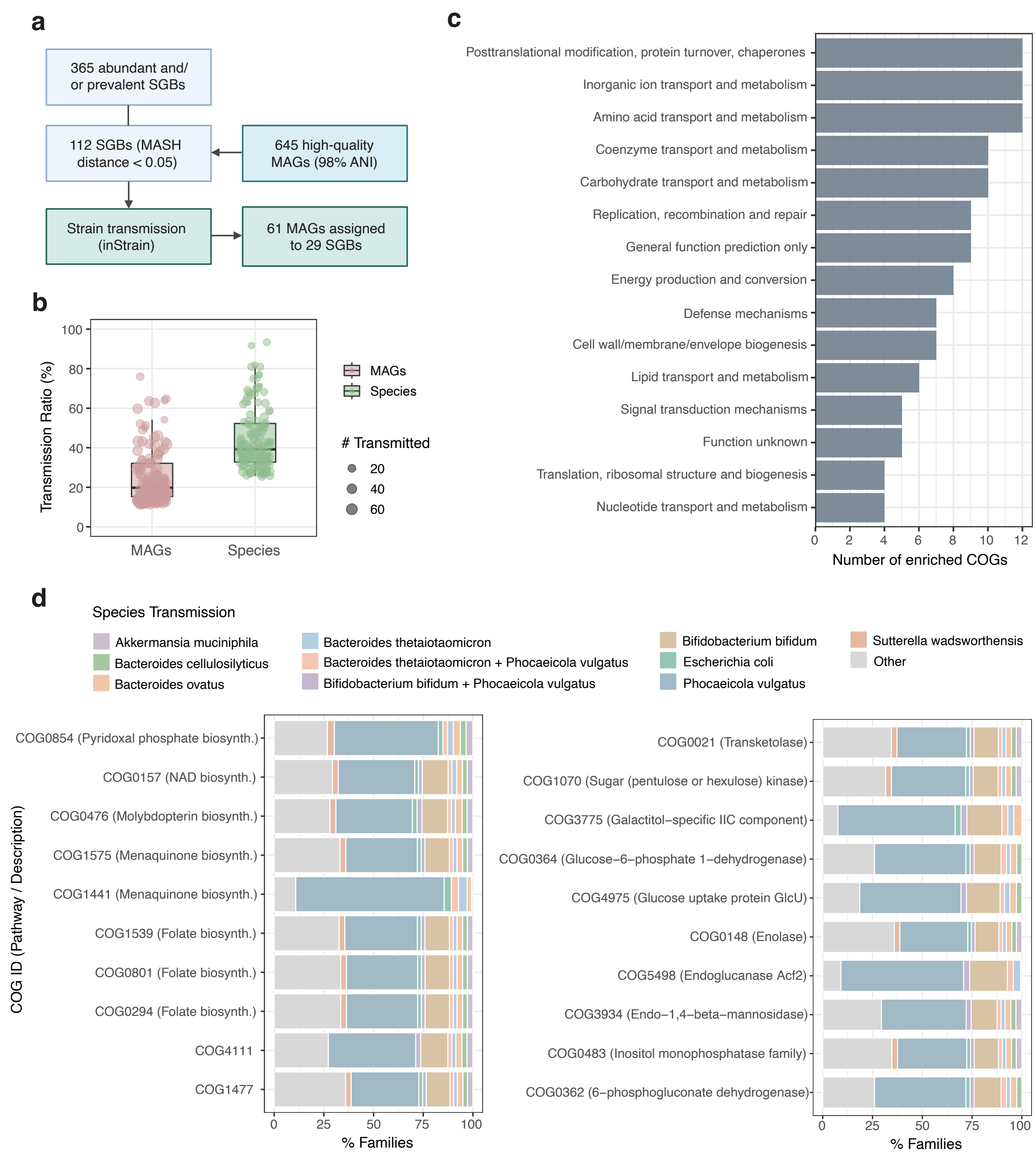

Figure S8. Functional enrichment of transmitted bacterial strains. a, *Schematic overview of genome-resolved strain-sharing analysis. Starting from 365 prevalent and/or abundant SGBs detected in mothers and infants, 645 high-quality MAGs were identified (dereplicated at 98% ANI), corresponding to 112 SGBs (MASH distance < 0.05). Strain transmission was detected for 61 MAGs assigned to 29 SGBs based on*

high nucleotide identity (population ANI >99.999) between maternal and infant metagenomes. ***b****, Boxplots comparing transmission ratios of individual COGs across MAGs (red) and species (green). Each dot represents a single COG. The Y-axis shows the transmission ratio, calculated as the number of MAGs (or species) that encode the COG and were transmitted, divided by the total number of MAGs (or species) that encode the COG. The size of each dot reflects the number of transmitted MAGs or species in which the COG was detected.* ***c****, Bar plot of the number of significantly enriched COGs grouped by COG functional categories (top 15 categories are shown).* ***d****, Species-level contributions to the transmission of enriched COGs within the Coenzyme Transport and Metabolism (left) and Carbohydrate Transport and Metabolism (right) categories. Stacked bars show the percentage of mother-infant pairs in which each species or species combination contributed to COG transmission. Top contributors are highlighted; others are grouped as "Other."*

Minor comments

L.161: "B. breve"

Reply: This has now been corrected: "Bifidobacterium species (*B. bifidum, B. longum and B. breve*) increased in the first months with the highest abundances at M6 (**Table S19**)."

L.170-172: The intra- versus inter-individual alongside intra- versus inter-familial variations observed for the infant microbiome could be related to the patterns observed more generally for humans.

Reply: We agree and have added the corresponding sentence to the end of this paragraph to highlight 3 important previous studies (PMID: 35418674, PMID: 34969979 & PMID: 33838112): "These patterns align with previous reports demonstrating higher microbiome similarity over time within individuals and among family members[20–22]. "

L.214-216: The MEFISTO analysis should be explained in more detail and a rationale for selecting 10 factors should be provided.

Reply: We thank the reviewer for this question, which was also posed by Reviewer 1. The purpose of the MEFISTO analysis was to understand which phenotypes drive patterns of microbiome variation throughout the first year of an infant's life. MEFISTO is an ideal tool for this objective because it reduces the dimensions of the data into factors, which are latent representations of biological processes driving variation. Furthermore, MEFISTO is unique in that it estimates the strength of these factors over time. Indeed, the true underlying process (or processes) that contribute to the variation driving a factor estimated by MEFISTO is likely due to a mix of biological variables. Therefore, after factors were estimated by the MEFISTO model, our aim was to identify the biological variable (phenotype) that most significantly explained each factor while correcting for other confounding technical and biological covariates.

To achieve this, a set of phenotypes known to exert strong ecological selective pressures on the colonization of the infant microbiome were modeled against each factor. The modeling strategy employed what can best be described as a sequential association analysis with a stepwise correction. This strategy is analogous to fine-mapping in genome-wide association studies where, after an initial round of testing each SNP individually, the top-associated SNP is added as a covariate in the next round of models. This stepwise conditioning helps unmask additional signals that may be hidden in the first round and reveals which SNP has the strongest effect on the phenotype. In brief, all 13 phenotypes included in the MEFISTO analysis (see Methods) were initially fit to each of the factors, adjusting for time and technical covariates related to each sample's microbiome sequencing (DNA concentration, read depth, and batch number). Each phenotype-factor pair was thus tested separately, and significance was assessed with the likelihood ratio test. The phenotype with the lowest *P*-value was considered the strongest association to the factor.

To both correct for potential confounding effects and reveal a more accurate relationship between this most significantly associated phenotype and the other phenotypes, we performed an additional sequence of associations. In this next round, models were refitted for the remaining 12 phenotypes, with the addition of the most significant phenotype from the first round of associations as a covariate. Should the *P*-value of any of the now-corrected phenotypes be lower than that of the initially most significant phenotype, the corrected phenotype with the lowest *P*-value would be considered the strongest association. In both the initial and corrected round of analyses, models were corrected for multiple testing using Bonferroni correction. This modeling strategy was carried out per factor. Given the stepwise approach described above, the analysis of phenotype-factor associations was multivariable-adjusted, accounting for both technical and biological covariates.

All in all, this strategy allowed us to evaluate whether the effect of a phenotype was previously masked by a more dominant, potentially confounding phenotype. This procedure is described in the Methods section of the manuscript, and the revised corresponding section now reads:

"A set of 13 predictors known to exert strong ecological selective pressures on the colonization of the infant microbiome[12,23] were modeled against each factor. These predictors were birth mode, feeding mode, duration of delivery, place of delivery, parity, pet ownership, infant eczema diagnosis, infant birth weight, infant food allergy, sex, maternal age at delivery, duration of broken water during delivery and maternal smoking history. The modeling strategy employed a sequential association analysis with a stepwise correction. In brief, all 13 predictors were initially fit to each of the factors, adjusting for time and covariates related to each sample's microbiome sequencing, including the DNA concentration, read depth and sequencing batch number. Each predictor-factor pair was thus tested separately, and significance was assessed with the likelihood ratio test. The predictor with the lowest *P*-value was considered the strongest association to the factor. This first round of associations is referred to as the 'initial_model' in **Table S21**. To both correct for potential confounding effects and reveal a more accurate relationship between the most significantly associated predictors and the other predictors, we performed an additional sequence of associations. These are referred to as the 'corrected_model' in **Table S21**. In this next round, models were refitted for the remaining 12 predictors, adding the most significant predictors from the first round of associations as a covariate. If the *P*-value of any of the now-corrected predictors was lower than that of the predictor that was initially most significant, the corrected predictor with the lowest *P*-value was considered the strongest association. In both the initial and corrected round of analyses, models were corrected for multiple testing via Bonferroni correction. This modeling strategy was carried out per factor. This correction procedure did not change the most strongly associated predictors from the first round of fitting, with the exception of Factor 10, where parity was considered the strongest association after correction for feeding mode."

Regarding the pre-determined selection of 10 factors: we determined that using 10 factors strikes a balance between interpretability and optimal fitting of the data. In general, fitting fewer factors tends to yield more biologically interpretable results, as the variance explained by each factor—and the underlying biological process(es) it represents—is less likely to be split across factors. On the other hand, while increasing the number of factors can help capture more subtle variance structures, it also raises the risk that a single biological signal is fragmented across multiple factors, which can obscure interpretation.

In our analysis, we observed an "elbow" in the variance explained by each factor at Factor 6 (see figure below). This elbow marks the point where the drop in variance explained per factor levels off sharply. This suggests that the first five factors account for most of the structured variation in the data and are thus the key biological variables (i.e., phenotypes) driving variation of the infant microbiome. Subsequent Factors 6–10 explain incrementally smaller amounts of variance. As such, adding more factors would likely not contribute to the overall interpretability of the data. And, while we could have run the analysis with only 5 factors, we chose to retain 10 to allow the model to detect finer-grained patterns of variation. This strategy was insightful for Factor 10, which allowed us to determine that parity was the most significant phenotype after correcting for feeding mode.

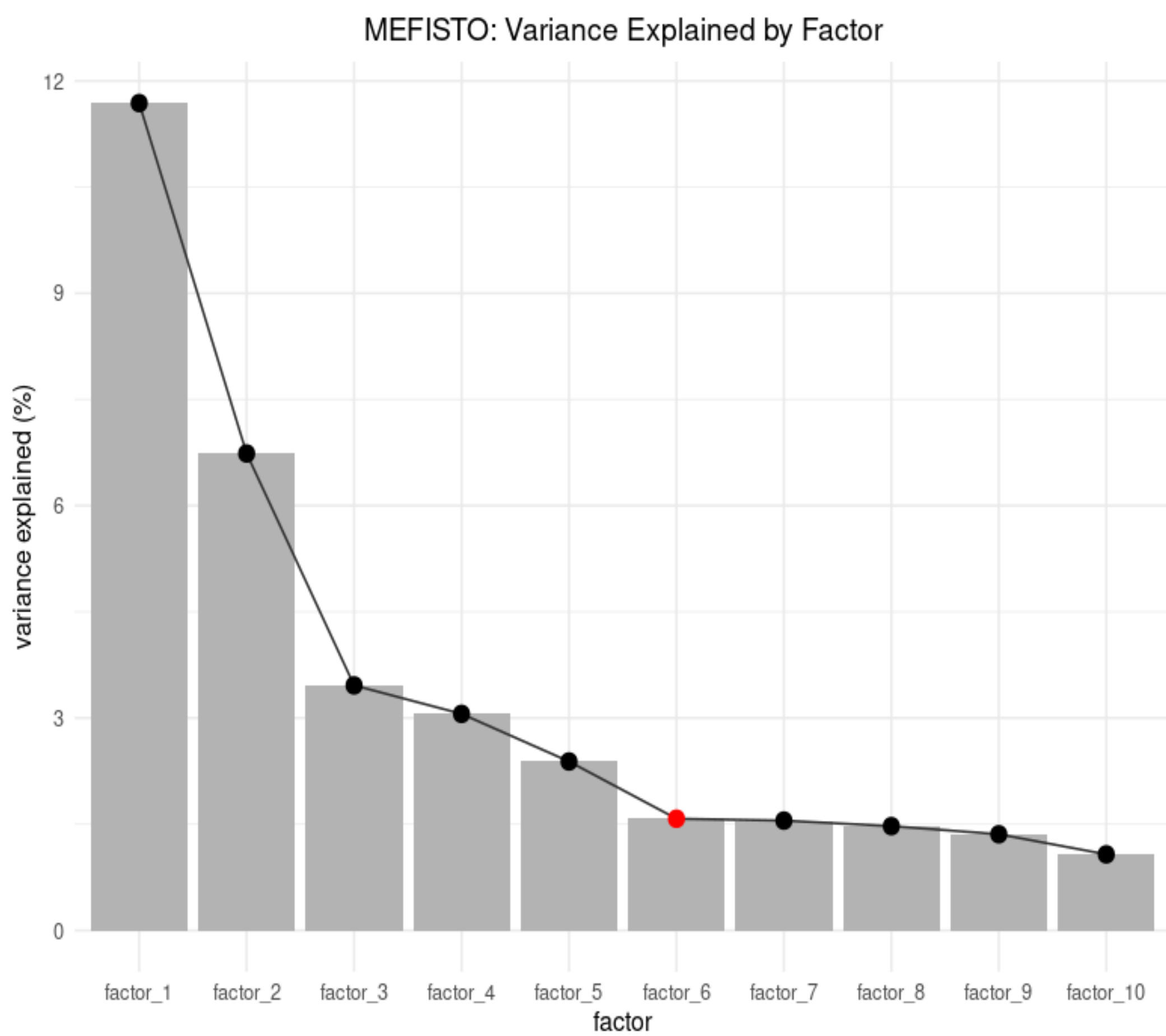

Variance explained by each factor in the MEFISTO analysis. *Each bar represents one of the ten factors estimated by MEFISTO and the proportion of variation explained in the microbial dataset. The red dot represents the elbow–the factor where the rate of decrease in variation explanation begins to level off.*

L.225: Figure 2c should be relabelled as Figure 2b to fit the flow of the text.

Reply: We agree and have relabeled Figure 2c as Figure 2b.

L.292: “mode of delivery”.

Reply: We thank the reviewer for noticing this and have corrected this.

L.381: “sweets and fats and oils”.

Reply: We thank the reviewer for spotting this and have corrected this.

L. 517: “B. thetaiotaomicron”

Reply: We thank the reviewer for spotting this and have corrected this.

Referee #2 (Remarks on code availability):

The paths to the code used could be clearer. I assume that the following path was the one to follow (?): NEXT_pilot_microbial_transmission_mother_infant/

Reply: We apologize for the earlier lack of clarity regarding the location of the codebase. We have now restructured our GitHub repository to make this more transparent and accessible. [redacted]

[redacted]

The repository now includes a comprehensive README file that outlines each component of the analysis, structured according to the order of the results presented in the manuscript.

[redacted]

Each section includes all relevant code, along with direct links to the corresponding Figures, Supplementary Figures, and Supplementary Tables generated.

Referee #3 (Remarks to the Author):
Referee #3: human microbiome, metagenomics, computational and bioinformatics analysis

Sinha et al. present a comprehensive longitudinal analysis of the maternal and infant gut microbiomes using data from 714 mother-infant pairs in the Dutch Lifelines NEXT cohort. This is a well-designed and ambitious study that aims to address the impact of microbiome changes occurring during pregnancy and the infant's first year of life on 474 clinical/dietary variables.

Beyond confirming several well-documented associations, the study offers additional insights into how the maternal microbiome is especially affected by diet, infections and smoking history. One particularly interesting observation was that the level of diversity in the maternal microbiome was found to be a key predictor of eczema in infants. Furthermore, they also show that early-life colonizers of the infant gut do not necessarily predict how the microbial community will establish later in life. I found this to be a very high-quality and in-depth study that further advances our understanding of microbial transmission and assembly in early life, a key area of microbiome research. My main criticism is that there are virtually no biological insights/mechanisms discussed underpinning their associations. This seems like a wasted opportunity with such high-quality data. In addition, as mentioned above, several of their associations (feeding mode, mother-infant strain sharing, etc. have already been covered in prior works such as in Shao et al. Nature 2019).

Reply: We thank the reviewer for their compliments, comments and suggestions. We have now substantially revised and extended the part on functional analysis and added extra data layers to study transmission of microbes from breast milk and vaginal microbiomes. Please see our replies to specific comments below.

1) The strength of the study is undoubtedly the detailed associations established between microbial markers of pregnancy and birth with health outcomes. However, there is limited effort to extract biological meaning from such interactions. Given the rich metagenomic data collected, it is a shame there is such a superficial analysis performed at a functional pathway level. The authors state there are >1,000 associations with various predictors, but there is no comparison on the strength of associations between taxonomy vs pathways and pinpointing specific functions linked to health outcomes.

Reply:
We agree with the reviewer. This comment was in line with both other reviewers and in response to this we have significantly expanded the functional analysis, extended the prediction of infant eczema using the maternal microbiome profile, and performed additional deep sequencing of vaginal and breast milk samples to address mother-to-infant transmission of microbes from all body sights. Please find the extended description of these changed below:

Given the relatively limited capacity of the infant digestive enzyme repertoire and their high reliance on microbial carbohydrate metabolism, we performed comprehensive functional annotation of the **Carbohydrate-Active Enzyme (CAZyme) profile** of the infant gut microbiome. This revealed dynamic changes throughout the first year of life. In contrast to adults, the infant CAZyme landscape was predominantly driven by the presence of *Bacteroides* species, with delivery mode playing a major role by influencing *Bacteroides* abundance. Further substrate-specific analysis showed that infants delivered vaginally were enriched for CAZymes involved in non-starch polysaccharide metabolism and those receiving breast feeding with CAZymes involved in resistant oligosaccharides, mucin and human milk glycans. Prior to solid food introduction, infants showed a significant enrichment of CAZymes involved in the metabolism of resistant oligosaccharides and starch, peptidoglycan, amylose, pullulan and amylopectin. In contrast, later timepoints were significantly enriched in CAZymes associated with glycoprotein, pectin and arabinogalactan metabolism. Finally, we observed significant associations between specific CAZyme subfamilies and infant growth trajectories.

We further conducted deeper investigations into **bacterial functional pathways using MetaCyc**, which revealed associations not only with delivery mode and infant feeding but also with parity, infant stool frequency and, notably, maternal dietary patterns. These findings reinforce the impact of maternal nutrition on the infant microbiome.

Lastly, a new analysis of **gut–brain modules** revealed compelling associations between infant feeding and pathways related to acetate synthesis, with formula feeding associated with lower acetate synthesis, potentially linking infant nutrition with microbial production of neuroactive metabolites.

These new results have been incorporated into the text and are shown in amended main Figure 3 and new supplementary figures S4, S5 and S6.

With regards to the analysis of CAZymes we added the following text:

"Infant CAZyme profile is highly dynamic during the first year of life, shaped by the presence of *Bacteroides*, *Parabacteroides* and *Phocaeicola*

Given the relatively limited capacity of the infant digestive enzyme repertoire and their high reliance on microbial carbohydrate metabolism, we annotated the carbohydrate-active enzyme (CAZyme) profiles of the infant and maternal gut microbiomes to investigate their dynamics and association with exposures. Similar to microbiome composition, the maternal CAZyme profile remained stable during pregnancy, while the infant CAZyme profile was highly dynamic throughout the first year of life (**Figure S5b** and **c; Table S41**). In the infant gut, *Bacteroides*, *Parabacteroides* and *Phocaeicola* were the main contributors to CAZyme profile variation (**Figure 3e, Table S42**), whereas *Bacteroides* played a less prominent role in the maternal gut (**Figure S5d, Table S43**). Most CAZyme associations were linked to mode of delivery, driven primarily by differences in the presence or absence of genus *Bacteroides*, *Parabacteroides* and *Phocaeicola* between the groups (**Figure S6**). Interestingly, after investigate associations with infant-specific traits (**Table S44** and **Table S45**) we also found significant associations of infant weight with CAZyme subfamilies even after correction for delivery and feeding mode, with the Glycoside Hydrolase Family 89 (GH89) being associated with

infant weight, length and head circumference (FDR=7.12×10^{-6} , 4.95×10^{-4} and 4.74×10^{-4}, respectively; **Table S45**). Further annotation of CAZyme substrates revealed that infants delivered vaginally were enriched for CAZymes involved in non-starch polysaccharide metabolism (NSP), whereas those delivered by CS showed enrichment for starch-metabolizing CAZymes (overrepresentation analysis, FDR=0.002 and 0.008, respectively; **Table S46**). These findings highlight the substantial role of delivery mode in shaping the functional potential of the infant gut microbiome.

In accordance with expectations, breastfeeding was associated with an enrichment of CAZymes related to the metabolization of resistant oligosaccharides, mucin and human milk glycans compared to formula feeding (FDR=0.013, 0.001, 0.000 respectively; **Table S47**). Compared to later time points, earlier time points showed a significant enrichment of CAZymes involved in the metabolism of resistant oligosaccharides and starch, peptidoglycan, amylose, pullulan and amylopectin (**Figure 3f, Table S48**). In contrast, later time points were significantly enriched in CAZymes associated with glycoprotein, pectin and arabinogalactan metabolism (**Figure 3f, Table S48**). This pattern reflects the rapid and dynamic development of the gut microbiome's functional capacity to metabolize diverse substrates during early infancy."

With regards to bacterial pathways, we added the following text:

"When investigating the relationships between individual bacterial species of the infant gut microbiome and infant-specific predictors, we identified 425 significant associations. However, the majority of these were related to delivery and feeding mode, and 193 associations remained after correction for these variables (**Tables S27** and **S28**). Associations were mostly linked to parity, infant stool consistency, frequency and diet (**Tables S27** and **S28**). Interestingly, we observed few associations with some of the unique predictors captured through our detailed questionnaires. For example, only single associations were found for sleeping and no FDR-significant associations were found with crying time (**Tables S27** and **S28**).

We observed 280 bacterial metabolic pathways of the infant gut microbiome significantly associated with 51 variables, with the highest numbers linked to feeding mode (238), parity and gravidity (169), infant stool frequency (162) and delivery-related factors (84), together mirroring the associations observed with bacterial species (**Tables S29** and **S30**). Some pathway associations were mainly driven by a single bacterial species, e.g. the negative association of PWY-5845 (menaquinol-9 biosynthesis) to infant fish and meat consumption was mainly driven by a decrease in abundance of *E. coli* genes belonging to this pathway. For other pathways, the association of pathway to predictors was driven by multiple bacteria. For example, the positive association of PWY-7013 (S-propane-1,2-diol degradation) to formula feeding was driven by the combined pathway-contributing genes of several *Klebsiella* species, *Salmonella* enterica, *Enterobacter cloacae*, *E. coli* and other species. Similarly, the positive association of LACTOSECAT-PWY (lactose and galactose degradation I) with formula feeding is driven by the pathway genes of multiple *Enterococcus* and *Streptococcus* species (**Figure S4**). After adjusting for delivery and feeding mode, we identified parity, gravidity and stool frequency to be associated with the majority of infant pathways. Additionally, infant complementary

feeding was associated with numerous pathways, including 22 linked to bread intake (primarily metabolic pathways) and 12 related to meat/fish intake. Intriguingly, maternal dietary patterns were also associated with 36 infant microbial pathways, reinforcing the potential impact of maternal nutrition on the developing infant microbiome (**Table S29**)."

With regards to infant microbial neuroactive potential, we added the following text:
"We next examined associations between the estimated abundance of gut–brain modules (GBMs)[27] and infant-specific predictors. We identified 125 FDR-significant associations, the majority of which were related to infant feeding mode (**Tables S31** and **S32**). Among the most significant findings, we observed a decrease in the quinolinic acid degradation pathway in formula-fed infants, while the acetate synthesis I pathway was significantly enriched in breastfed infants (**Figure 3d**). These results highlight potential differences in microbial neuroactive metabolism linked to early-life nutrition."

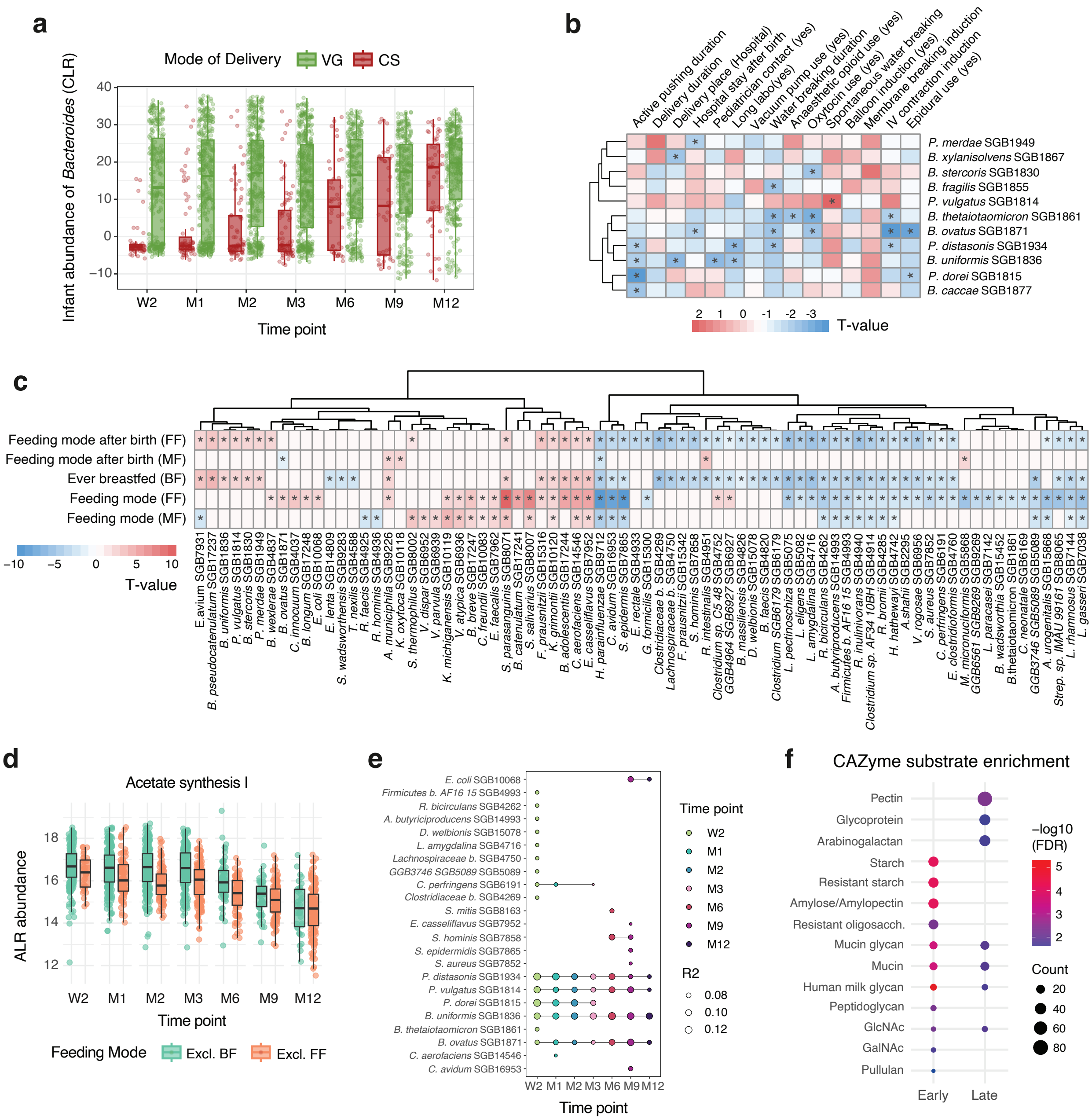

Figure 3. Associations of infant gut microbiome and CAZyme profile with predictors. a, Boxplots of the association between the centered log ratio (CLR)-transformed relative abundance of Bacteroides and delivery mode across different time points. ***b***, Heatmap of T-values for the association between birth-related factors and various Bacteroides species. * P-value < 0.05. ***c***, Heatmap of T-values for associations between feeding mode at birth, feeding mode from 2 weeks to 12 months of age and ever being breastfed or not with bacterial species at species-level genomic bin (SGB) level. * FDR < 0.05. ***d***, *Boxplots of the additive log ratio (ALR)-transformed abundance of acetate synthesis with either exclusive breastfeeding or formula feeding over time.* ***e***, *PERMANOVA of CLR-transformed SGB abundance on the Aitchison's distance matrix of the entire CAZyme profile of infants at seven time points. Only significant bacteria (permutation P-value <0.0001 and* R^2 *>0.06) are shown. For full results see* ***Table S42***. ***f***, *Overrepresentation analysis of CAZyme substrates at early (3 months or younger) versus late (6 months or older) time point of infants. Only FDR-significant (<0.05) results are shown. For* ***a*** *and* ***d***, *each boxplot visualizes the median, hinges (25th and*

75th percentiles) and whiskers extending up to 1.5 times the interquartile range from the hinges.

New supplementary figures pertaining to this part are as follows:

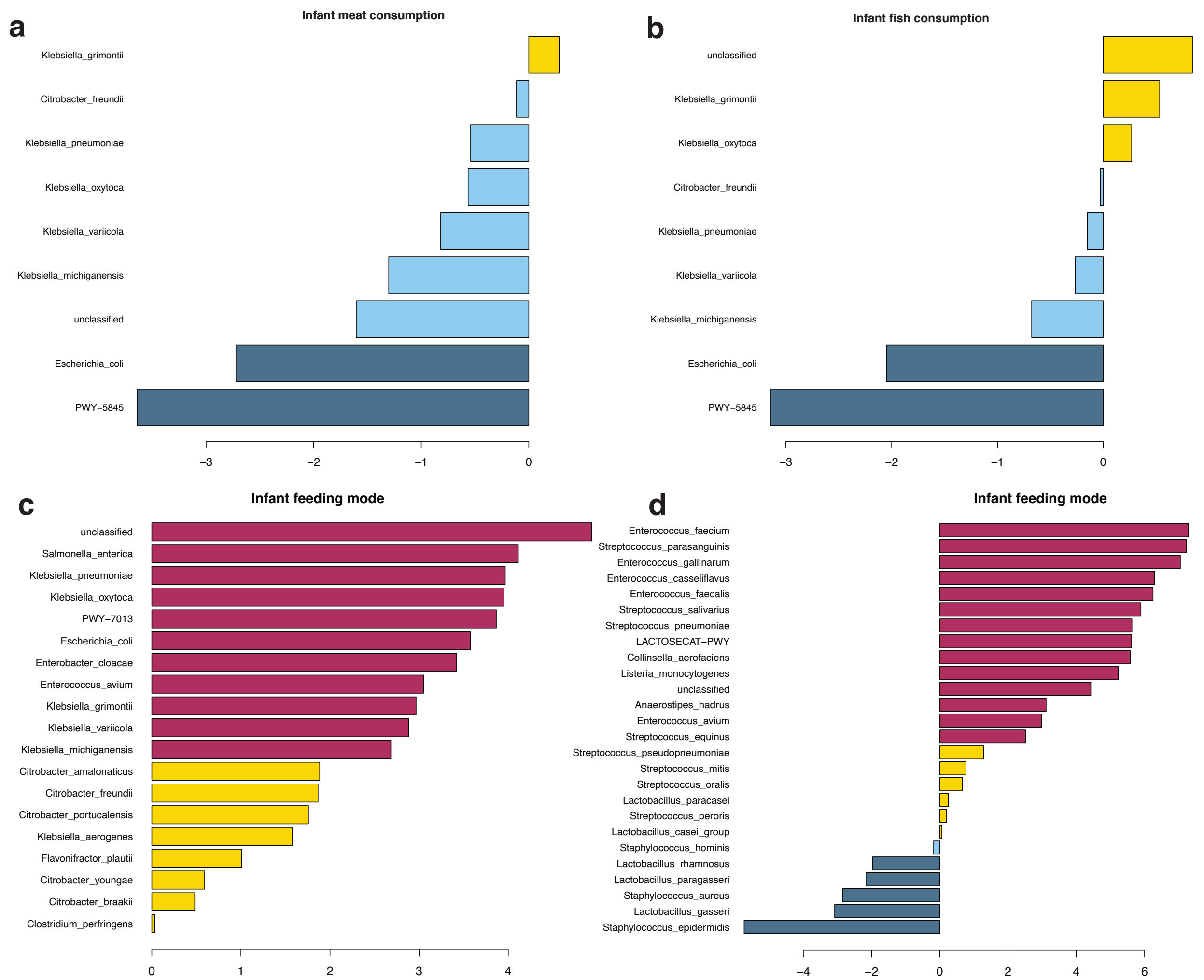

Figure S4. Per-species pathway abundance association to phenotypes*. X-axis denotes T-statistics of association of total pathway abundance (encoded with METACYC ID). Species names represent the association T-values of total abundance of genes belonging to a pathway from individual species. The “Unclassified” category represents association of pathway genes with no taxonomic binning.*

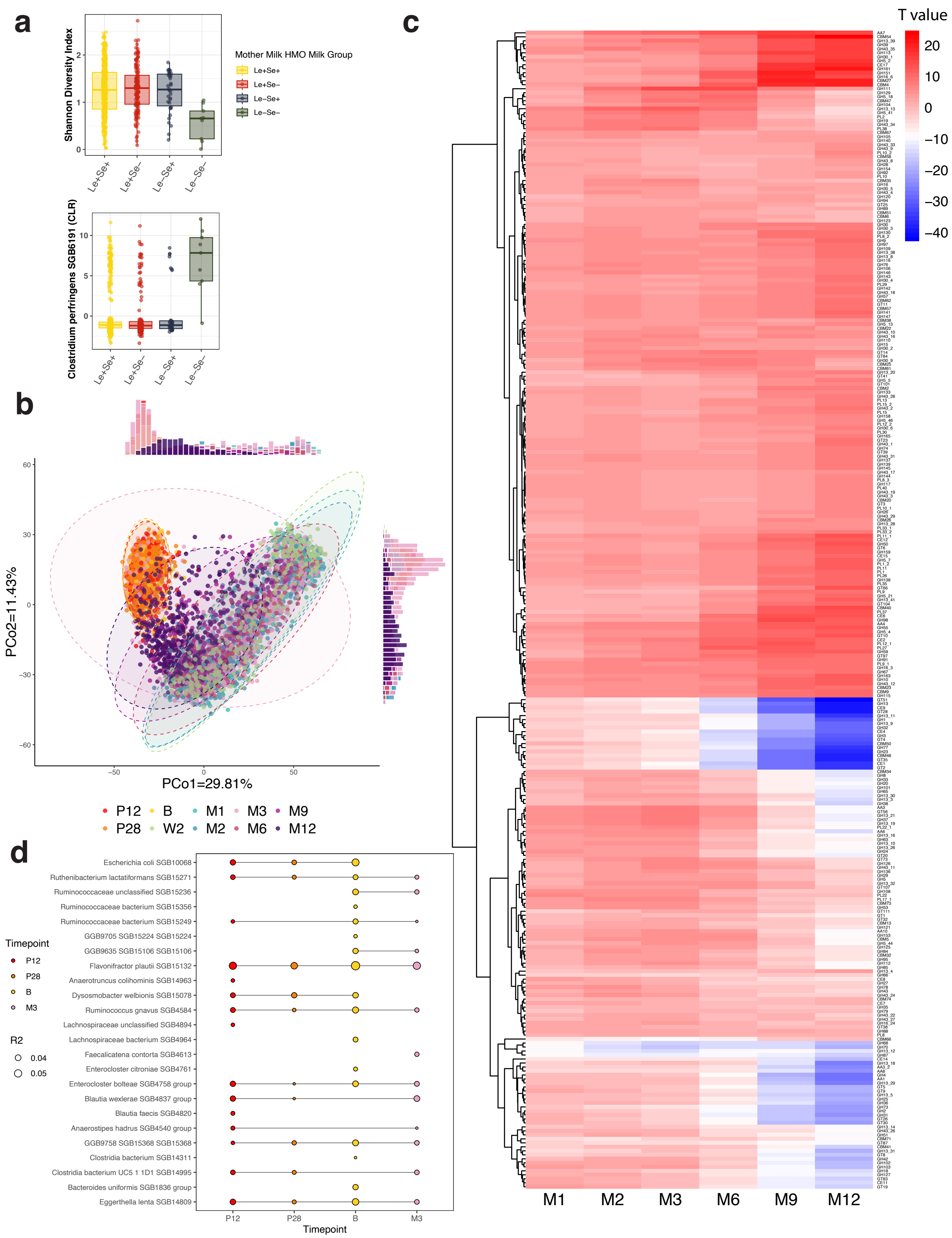

Figure S5. Maternal milk group and HMO associations and maternal and infant CAZyme profile. a, *Maternal secreter status and infant Shannon diversity index (above) and infant relative abundance of*

*Clostridium perfringens (CLR-transformed) (below). **b,** PCoA analysis based on Aitchison distance calculated at the subfamily level of maternal and infant CAZyme profile. **c,** T-values of the association of infant time point with CAZyme subfamilies using W2 as the reference. **d,** PERMANOVA of CLR-transformed SGB abundance on the Aitchison's distance matrix of the entire CAZyme profile of mothers at various time points.*

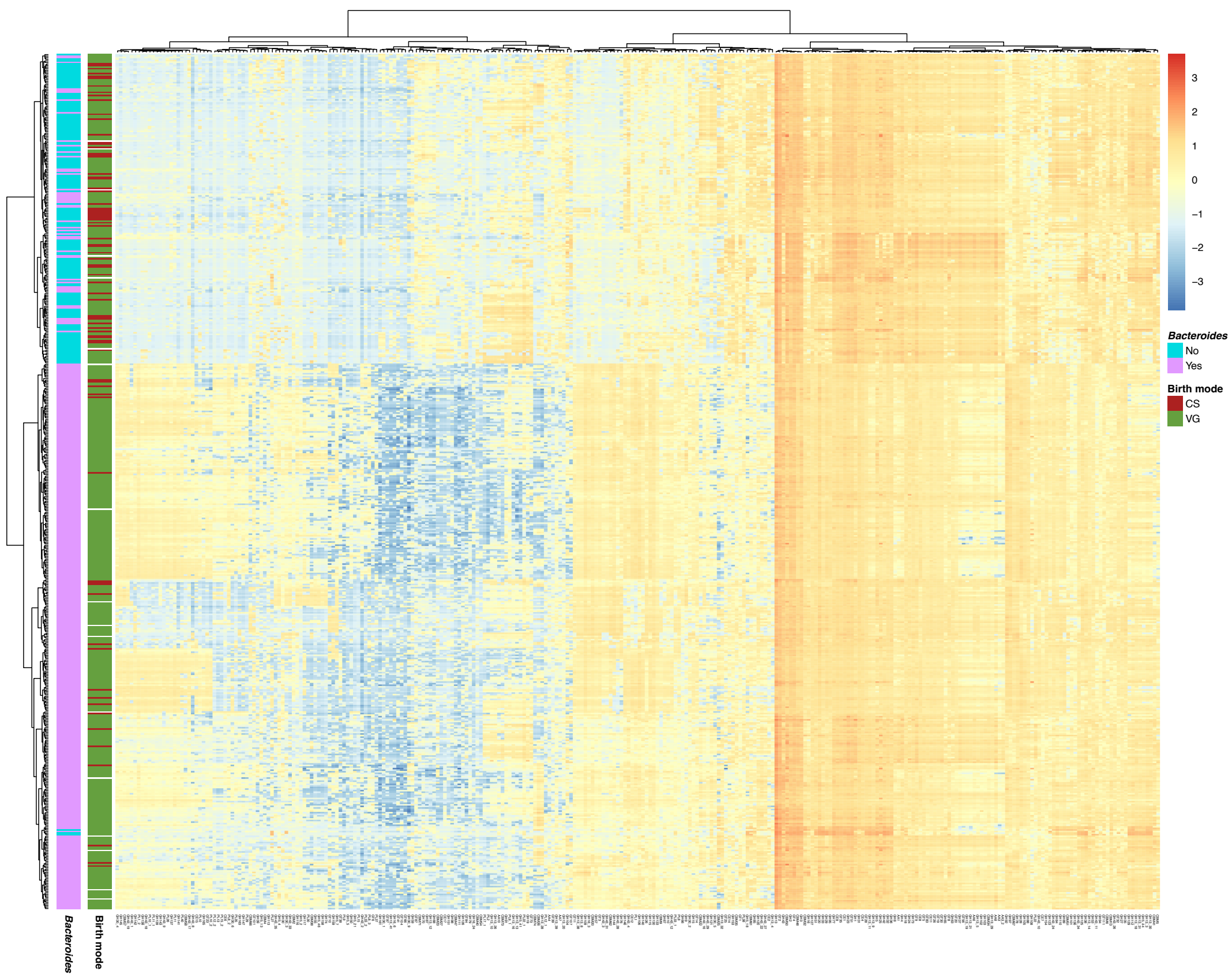

***Figure S6. Bacteroides is the major driver of infant CAZyme profile**. Heatmap of the infant CAZyme profile at month 3 (M3), with the X-axis representing CAZyme subfamilies. The Y-axis includes bar plots indicating the presence or absence of Bacteroides and the mode of delivery (vaginal (VG) vs. cesarean section (CS)).*

Our strain transmission analysis from mothers to infants gut now not only identifies transmitted strains and quantifies the extent of transmission, it also functionally characterizes transmitted strains. For this purpose, we generated >18,000 high-quality MAGs corresponding to 1,448 bacterial subspecies. Focusing on those assigned to prevalent and/or abundant SGBs, we predicted and functionally annotated their predicted proteins (n = 1.68 million), identifying >3,900 unique COG annotations. By integrating these annotations with our results regarding mother-to-infant strain transmission, we identified a set of protein functions that are significantly enriched among transmitted bacterial strains. Notably, **mother-to-infant gut transmitted strains are shown to be enriched in genes related to carbohydrate and coenzyme transport and metabolism**, suggesting possible functional advantages for successful

colonization. These new results have been incorporated into the text and are shown in new supplementary figure S8.

"Finally, we investigated if bacterial strains transmitted from mothers to infants might be enriched for specific metabolic capacities compared to the strains that were not transmitted. For this, we used a genome-resolved strain-sharing analysis focused on 365 prevalent and/or abundant SGBs detected in mothers and infants. In total, we identified 645 high-quality metagenomic assembled genomes (MAGs), dereplicated at the subspecies level (98% average nucleotide identity (ANI), that could be assigned to 112 of these SGBs (MASH distance < 0.05). We identified 61 MAGs belonging to 29 different SGBs with high nucleotide identity between maternal and infant metagenomes (population ANI ≥ 99.999), a cut-off commonly indicative of transmission in comparisons between whole MAGs (**Figure S8a**). These SGBs were also shown to be transmitted in families in our previous approach using dominant strain marker genes (99.52% concordance).

Functional enrichment analysis revealed 121 Clusters of Orthologous Genes (COGs) that were significantly overrepresented among the transmitted MAGs, based on their presence across a higher number of unique mother-infant pairs than expected by chance (FDR<0.05, one-sided permutation-based test; **Table S70**). On average, enriched COGs were transmitted across 25.4% of MAGs and 44.6% of species in which they were detected (IQR: 15.3–32.1% for MAGs and 32.8–52.3% for species per COG), suggesting that these functions may be broadly relevant to vertical transmission across diverse bacterial taxa (**Figure S8b**). Grouping the enriched COGs into broader functional categories revealed several functions potentially relevant to the early-life gut environment, including *carbohydrate transport and metabolism* (top enriched COGs include COG5498, Endoglucanase Acf2; COG3775, Phosphotransferase system, galactitol-specific IIC component and COG4975, Glucose uptake protein GlcU) and *coenzyme transport and metabolism* (including COG1441, O-succinylbenzoate synthase and COG0854, Pyridoxine 5′-phosphate synthase PdxJ) (**Figure S8c**). To identify the microbial contributors to the transmission of these functions, we estimated the proportion of mother-infant pairs in which each species, or species combination, contributed to the transmission of COGs within these two key functional categories. We found that *Phocaeicola vulgatus* was the predominant contributor, showing functional transmission in the largest proportion of mother-infant pairs, followed by *Bifidobacterium bifidum* and several *Bacteroides* species (**Figure S8d**)."

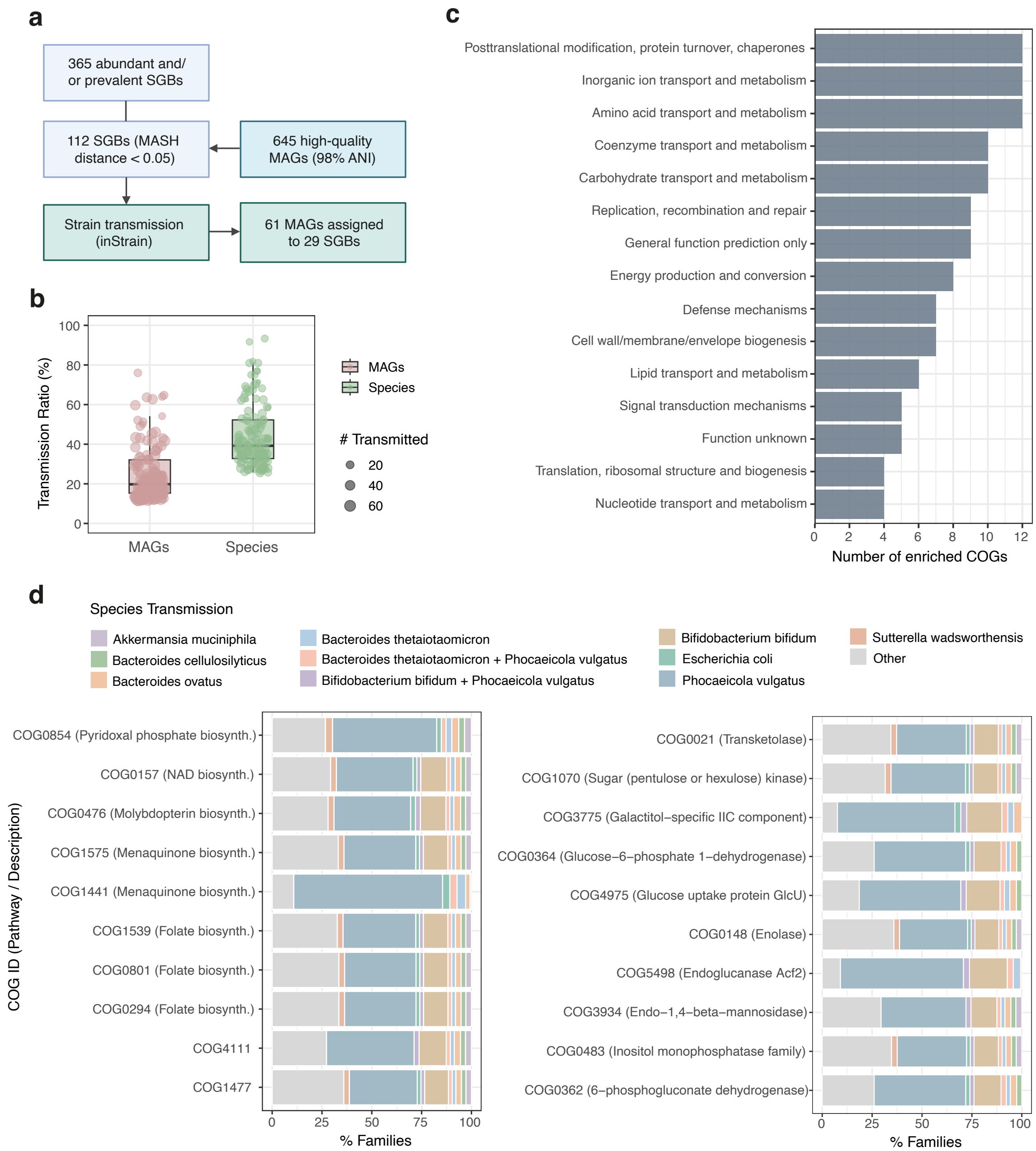

***Figure S8. Functional enrichment of transmitted bacterial strains. a**, Schematic overview of genome-resolved strain-sharing analysis. Starting from 365 prevalent and/or abundant SGBs detected in mothers and infants, 645 high-quality MAGs were identified (dereplicated at 98% ANI), corresponding to 112 SGBs (MASH distance < 0.05). Strain transmission was detected for 61 MAGs assigned to 29 SGBs based on high nucleotide identity (population ANI >99.999) between maternal and infant metagenomes. **b**, Boxplots comparing transmission ratios of individual COGs across MAGs (red) and species (green). Each dot represents a single COG. The Y-axis shows the transmission ratio, calculated as the number of MAGs (or species) that encode the COG and were transmitted, divided by the total number of MAGs (or species) that encode the COG. The size of each dot reflects the number of transmitted MAGs or species in which the COG was detected. **c**, Bar plot of the number of significantly enriched COGs grouped by COG functional categories (top 15 categories are shown). **d**, Species-level contributions to the transmission of enriched*

COGs within the Coenzyme Transport and Metabolism (left) and Carbohydrate Transport and Metabolism (right) categories. Stacked bars show the percentage of mother-infant pairs in which each species or species combination contributed to COG transmission. Top contributors are highlighted; others are grouped as "Other."

Lastly, in addition to metagenomically sequencing infant stool, we have now included **deep metagenomic sequencing of maternal breast milk and vaginal microbiomes.** To our knowledge, ours is one of the only metagenomic studies to carry out strain-level analysis on these maternal body sites in the context of infant seeding and colonization. Our findings indicate that, while there is species-level overlap between breast milk and infant gut, strain-level transmission is infrequent, suggesting that breast milk is not a major reservoir of dominant gut-colonizing strains in infants. For the vaginal microbiome, the species overlap with the infant gut is even more limited. However, in specific cases—e.g., *Bifidobacterium breve*—we observed 100% strain transmission when the species was present in the maternal vaginal microbiome at delivery. Hence, using this unique dataset, we can conclude that the maternal gut microbiome is a major reservoir of infant gut strains, with occasional transmission from vaginal and breast milk microbiomes. These new results have been incorporated into the text and are shown in new main Figure 5 and new supplementary figure S7.

"The maternal gut microbiome is the main reservoir for infant gut strains, with occasional transmission from vaginal and breast milk microbiomes

Mother-to-infant sharing of bacterial strains is a hallmark of early-life bacterial transmission[6,41–44] . However, the source and transmission routes from mother are not yet clearly defined. As some of the maternal body-niches have low bacterial biomass (e.g., milk) or very high amounts of human DNA (e.g., vaginal), we needed to sequence especially deep to reach high confidence in our strain annotations. Thus, for this analysis, in addition to our sequenced fecal samples, we generated ultra-deep (~40 GB/sample) metagenomic data for 90 maternal breast milk samples collected during the first month of life and 82 vaginal samples collected close to birth, enabling us to quantify strain-sharing from these additional maternal sources. For each individual species, we defined strain-sharing among two metagenomes using a previously developed approach[45]. To differentiate between the same and different strains, we established genetic distance cut-offs for 1,205 species using longitudinal samples from individuals (see **Table S57** for specific cut-offs per SGB).

When analyzing maternal breast milk samples, we found that *Staphylococcus epidermidis* (SGB7865), *Streptococcus mitis* (SGB8163) and the *Streptococcus salivarius* group (SGB8007) were abundant and prevalent members of the breast milk microbiome (**Table S58**). These species co-occurred in the infant gut (relative abundance >0.01%) in 50.0%, 42.2% and 38.9% of the 90 mother-infant pairs, respectively (**Table S59, Figure S7a**). Among those co-occurring cases, strain-level sharing between breast milk and the infant gut was observed only sporadically (**Table S60**), with strain-sharing of maximally co-occurrent taxa *Staphylococcus epidermidis* (SGB7865) detected in just two families (**Table S60**). Furthermore, while phylogenetic distances between dominant strains of related mother-infant pairs were lower than those between unrelated mother-infant pairs

in the majority of the cases (**Figure S7b**), this was only significant for *Bifidobacterium breve* (SGB17247) and *Enterococcus faecalis* (SGB7962) (**Figure S7b**) with these two species showing strain transmission from breast milk to infant gut in two families each (**Table S60**). Hence, although there is some species-level overlap, strain transmission from maternal breast milk to the infant gut appears infrequent, suggesting that breast milk is not a major reservoir for gut-colonizing strains that become dominant in infants.

We observed even fewer species shared between the vaginal microbiome and the infant gut. The vaginal microbiome was dominated by *Lactobacillus crispatus* (SGB7045), *Lactobacillus iners* (SGB7025) and *Gardnerella vaginalis* (SGBs 17302 and 17307), all of which were rarely detected in infant gut samples, corresponding to what was previously observed[7]. (**Table S59** and **Table S61**). The species *Lactobacillus gasseri* (SGB7038 group) co-occurred in 13.4% of mother-infant pairs, with strain transmission observed in a single family (**Figure S7a, Table S59, Table 62**). Intriguingly, in all five mothers in which we could construct dominant strains of *Bifidobacterium breve* (SGB17247) in vagina, the exact same strain was constructed in their infant's gut (**Figure 5 a, b**). Notably, we could construct the same strain in the maternal gut from only one of these five families. Four of these five infants were born via vaginal delivery, and the fifth was born by post-labor CS. Although strains of these species were also detected in infants at later time points (**Figure 5**), these strains differed from those found earlier, strongly suggesting vertical transmission of the vaginal *B. breve* strain during birth."

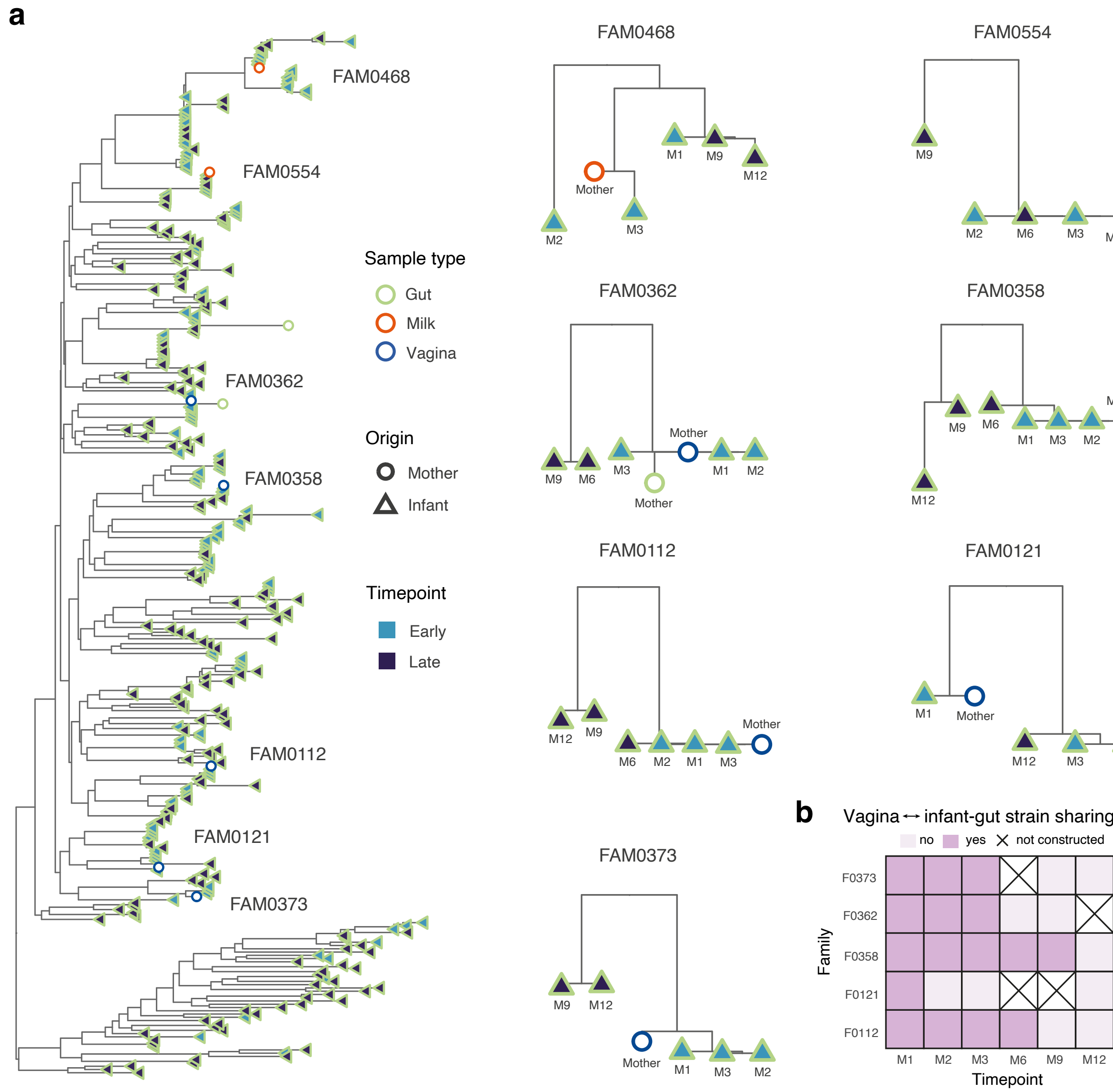

Figure 5. Strain-sharing between maternal breast milk, vaginal and infant gut samples. a, *Maximum likelihood phylogenetic tree of Bifidobacterium breve dominant strains reconstructed from families from which a vaginal sample or breast milk sample was sequenced. Maternal samples are depicted by circles, infant samples by triangles. Colors indicate sample type: green for gut, blue for vagina and orange for breast milk. Expanded insets for all five families are also shown.* ***b****, Heatmap depicting families in which Bifidobacterium breve strains were reconstructed from vaginal samples at infant birth and infant gut over time (M1 to M12). Light color indicates no strain-sharing. Cross indicates that the strain could not be reconstructed from the infant gut at the corresponding time point.*

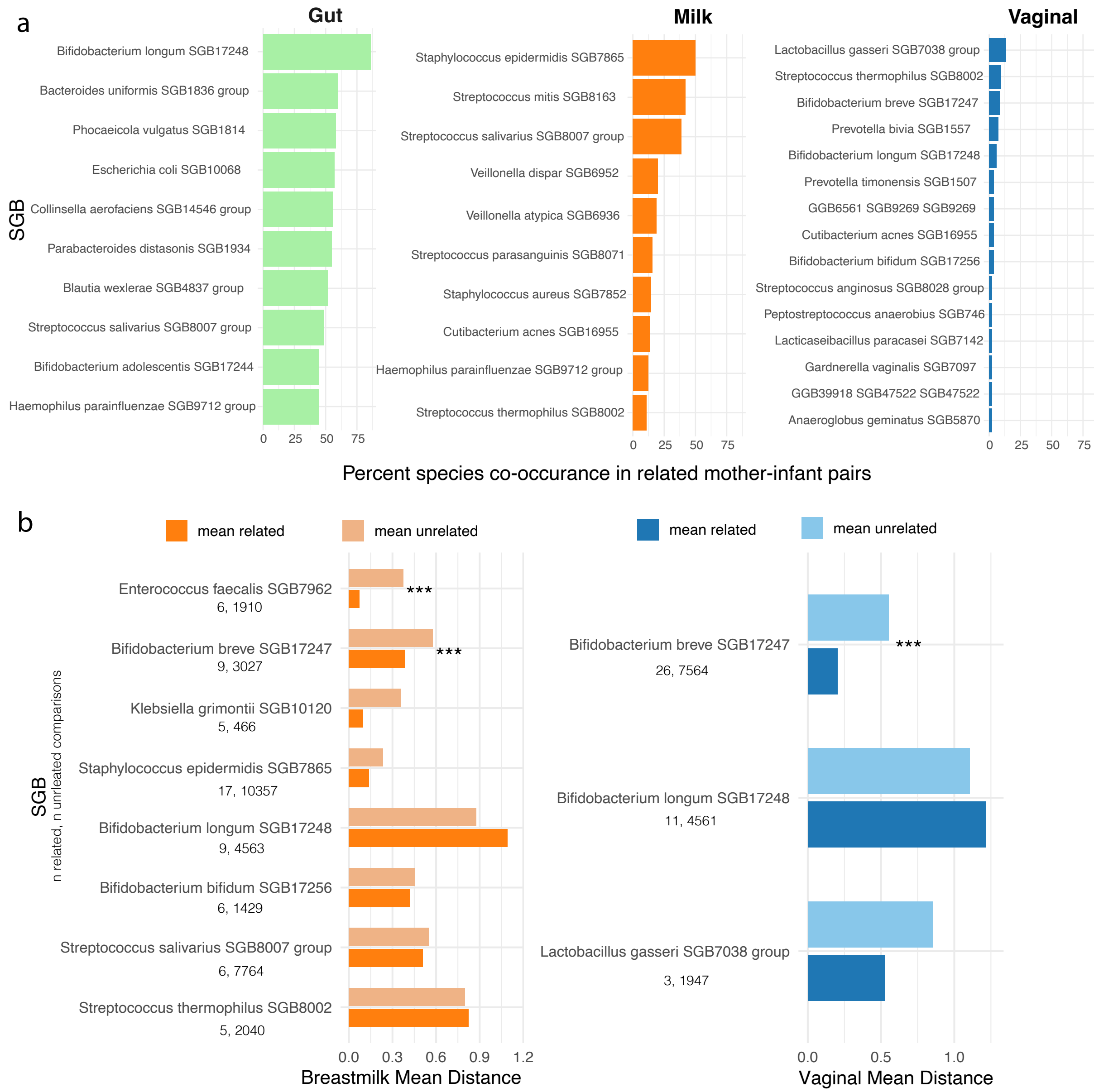

Figure S7. Species co-occurrence between mothers and infants and phylogenetic distances. a, *Percentage of species co-occurrence between maternal samples (gut, breast milk or vaginal) and the infant gut in paired mother-infants. Presence was defined as a relative abundance of an SGB >0.01% in both mother and infant. Sample sizes for gut, milk and vaginal pairs were 666, 90 and 82, respectively. The top 10 SGBs for each group are shown.* ***b,*** *Mean phylogenetic distances between related and unrelated mother-infant pairs between breast milk and infant gut (left in light and dark orange) and vagina and infant gut (right in light and dark blue). The number of related versus unrelated comparisons are shown in the numbers below the SGB name. P-values were calculated using a permutation-based test. *** P-value < 0.05.*

2) The authors show that maternal alpha diversity has high predictive power of infant eczema. Beyond overall diversity, were there specific species showing a stronger

association? They should compare model accuracy in using the microbiome composition (e.g., species/functions) vs alpha diversity alone.

Reply: We thank the reviewer for this important comment. In response, we have substantially revised the analysis to compare the predictive performance of different microbiome-derived features.

Firstly, to compare the independent association of alpha diversity with infant eczema, we conducted a multivariable logistic regression including maternal Shannon diversity, smoking history (previously associated with decreased diversity) and family history of allergic disease. Only alpha diversity remained significantly associated with infant eczema (*P* = 0.0038), while the other covariates were not significant.

We then went on to compare the predictive power of Shannon diversity with other microbiome-derived features like species and pathways. To do so, we updated our predictive analysis framework to use leave-one-out cross-validation (LOO-cv), which offers more robust estimates compared to our previous 5-fold cross-validation approach. Using LOO-cv and XGBoost classifiers, we evaluated three sets of predictors derived from the maternal microbiome at birth:

- Taxonomic features (CLR transformed SGB level): AUC = 0.74
- Functional pathways (ALR transformed MetCyc): AUC = 0.73
- Alpha diversity (Shannon diversity index): AUC = 0.68

These updated results are now presented in revised **Figure 4e**. The results show that taxonomic composition outperforms alpha diversity and functional profiles in predicting infant eczema.

To further explore which microbial features were most influential in the predictive model, we computed SHAP (SHapley Additive exPlanations) values for the species-level taxonomic model. The top contributor was *Dialister invisus*, a species previously implicated in mediating the effects of a prudent maternal diet on systemic inflammation. We have now updated the text and added these new panels to **Figure 4**.

"Lastly, we investigated whether the maternal microbiome could predict infant health outcomes. Maternal alpha diversity was significantly lower in women whose infants developed eczema during the first year of life (FDR = 0.02; **Figure 4d, Table S49**). To assess whether this association was independent of other risk factors, we performed a multivariable logistic regression including maternal Shannon diversity, smoking history (as it was previously linked to reduced diversity; **Figure 4a, Table S49**) and family history of allergic disease. Notably, only maternal alpha diversity remained significantly associated with infant eczema (*P*=0.004), while the other variables were not.

Building on this finding, we developed predictive models to evaluate three sets of maternal microbiome features at birth: SGB taxonomic composition (AUC = 0.74), alpha diversity (AUC = 0.68) and functional pathway profiles (AUC = 0.73) (**Figure 4e**). To identify the main microbial contributors within the taxonomic model, we computed SHAP (SHapley Additive exPlanations) values. The top-ranked species was *Dialister invisus*, which was associated with a lower risk of eczema in infants (**Figure 4f**). This species has

previously been implicated in modulating systemic inflammation in response to a prudent maternal diet[40].”

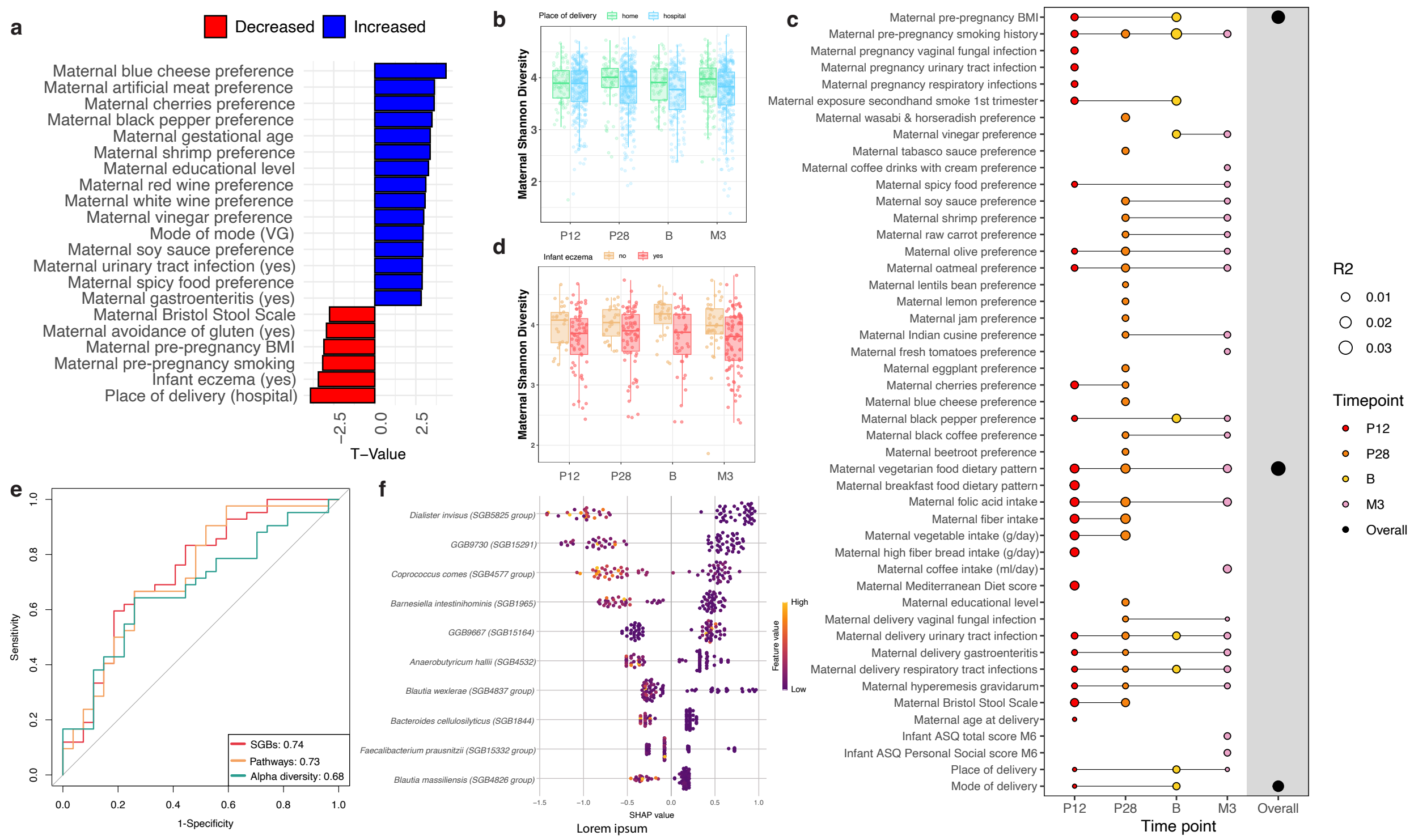

Figure 4. Maternal gut microbiome dynamics and associated factors. a, *T-values of the FDR-significant (<0.05) associations between maternal alpha diversity (Shannon diversity index) and maternal-specific predictors.* ***b,d,*** *Boxplots of the distribution of maternal alpha diversity (Shannon diversity index) between* ***b****) place of delivery and* ***d****) infant eczema presence at 12 weeks of pregnancy (P12), birth (B) and 3 months postpartum (M3). Each boxplot visualizes the median, hinges (25th and 75th percentiles) and whiskers extending up to 1.5 times the interquartile range from the hinges.* ***c****, Maternal microbiome variation explained (R^2) by predictors (PERMANOVA) at four specific time points and an overall time point of combined samples (n=101) collected at P12, B and M3. Sample sizes for each time point are depicted in* ***Figure 1a*** *above. The ‘Overall’ group was obtained by PERMANOVA on factors derived from TCAM. All PERMANOVA was performed with 10,000 permutations, and only results with significance of P<0.005 are shown.* ***e,*** *Receiver operating characteristic (ROC) curve derived from a logistic regression prediction model of infant eczema using maternal alpha diversity. Curves represent statistics from each model obtained from a leave-one out cross-validation. The overall area under the curve (AUC) value, derived from predictions from all models, is displayed.* ***f,*** *SHAP (SHapley Additive exPlanations) values of the top 10 species contributing most to model predictions*

3) How was the non-independent nature of strains across timepoints within the same subject taken into account in their phylogenetic analysis (e.g., Lines 553 onwards)? They find that late colonizers are different compared to early colonizers. But how many instances did the authors find of the same strain being maintained from one timepoint to the next in the same individual vs introduction of new strains later on?

Reply: We apologize for the fact that the description of PGLMM approach was insufficient and that this raised the concerns regarding non-independence of strains

within individuals. We thoroughly address a similar concern raised by Reviewer 1 in our response to their Question 12. In brief, the inclusion of multiple strains per individual makes PGLMMs perform very poorly. Therefore, each individual was included only once in our PGLMM analysis, even if data from multiple time points was available for that individual.

4) Why were read depth, DNA concentration and batch number the only variables corrected for in the association tests? Was there a statistical basis for this? I would imagine other aspects such as diet and medication would need to be considered as well.

Reply: We agree that other aspects need to be considered and indeed corrected for them in specific analyses.

The three variables mentioned (read depth, DNA concentration and batch number) were true technical covariates that were adjusted for 'as is', while the selection for biological covariates differed across the approaches we used.

Furthermore, based on the results of the MEFISTO analysis described above, which identified delivery mode and feeding mode as major drivers of infant gut microbiome composition, all analyses were adjusted for these variables, unless otherwise stated. In all supplementary tables reporting associations between factors and microbial species, pathways, gut–brain modules, or CAZymes, the corrected estimates and *P*-values are provided. We also consistently apply multiple testing correction and report FDR-significant results, unless specified otherwise.

Commonly used medications in infants, such as analgesics, did not show a major impact on the gut microbiome in any of the analyses we performed. Other medications, like probiotics and antibiotics, were excluded from the analysis due to insufficient statistical power. For instance, at each timepoint, fewer than 2% of infants consumed probiotics and fewer than 10% consumed antibiotics (**Table S4, S6**). As a result, we chose to exclude these variables from further consideration.

5) Different prevalence and abundance thresholds were used for mothers (0.001% abundance, 30% prevalence) compared to infants (0.1% abundance and 10% prevalence). This does not make much sense to me. The authors should decide on the thresholds that maximize accuracy and apply them consistently to all samples.

Reply: We would like to clarify why we chose separate cut-offs for both mothers and infants. For the maternal samples, we applied a minimal relative abundance threshold of 0.001% and a prevalence threshold of 30% per individual (as opposed to per sample). These cut-offs were selected to ensure that we retained species with sufficient prevalence across individuals to support robust phenotype association testing.

However, infant gut microbiome composition is markedly different from that of adults, with a characteristic structure often dominated by a few highly abundant taxa. We therefore used a higher relative abundance threshold (0.1%), as there is a higher likelihood of false detection of species via contamination. We also decided to use a lower prevalence threshold (10%) based on prevalence per infant (again, not per

sample). We do observe that some species were detected in a small fraction of infants, but in a relatively high abundance. Those species might thus have a substantial biological relevance, and we strongly believe they should be kept in the analysis.

6) How was a CLR transformation applied to the MetaPhlAn results? CLR transformation is typically applied to read count data (e.g., see the ALDEx2 approach), but MetaPhlAn outputs relative abundance data. This should be carefully checked to ensure their approach complies with best practices in the field.

Reply: CLR-transformation carries the characteristic of being scale-invariant. Thus, CLR will give exactly the same values for both count data and derived relative abundance data:

Counts:

$$clr_i = log\left(\frac{x_i}{g(x)}\right), \text{ where } g(x) = \prod_1^n \quad x_i$$

Abundance data derived from counts:

$$clr_i = log\left(\frac{x_i/\sum_1^n \quad x_i}{g(x)}\right), \text{ where } g(x) = \prod_1^n \quad (x_i/\sum_1^n \quad x_i)$$

$$clr_i = log\left(\frac{x_i/\sum_1^n \quad x_i}{\sqrt[n]{\prod_1^n \quad \frac{x_i}{\sum_1^n \quad x_i}}}\right) = log\left(\frac{x_i/\sum_1^n \quad x_i}{(\sqrt[n]{\prod_1^n \quad x})/\sum_1^n \quad x_i}\right) = log\left(\frac{x_i}{g(x)}\right)$$

Thus, applying the CLR-transformation—and using Aitchison distance—yields equivalent results whether it is done on raw counts or relative abundances. The original framework of compositional data analysis proposed by Aitchison starts with the idea of the positive simplex, i.e., data made up of proportions that add up to a whole. Applying this approach to count data is possible because CLR-transformation is scale-invariant, meaning it gives the same result regardless of the total size of the sample.

7) The definition of "parity" for the purpose of this study should be defined early on (i.e., infants with siblings vs without siblings).

Reply: We agree and have changed the text to read: "45.1% of mothers delivering their first infant (i.e. parity is 0)"

We have additionally also clarified this in the Results: "This is likely a proxy for sibling exposure, as previous studies have shown that having older siblings influences the development of the infant gut microbiome[24,25]"

8) Line 292: Typo "pf" -> "of"

Reply: We thank the reviewer for this remark and have corrected this error.

Referee #3 (Remarks on code availability):

The code appears to have been made available. However, the authors should provide in their README more detailed instructions on the structure of the folders/scripts and how to replicate the analysis in their manuscript. At the moment, the repo's structure is quite confusing and makes it hard to reproduce their work. In addition, the current README mentions both virome and multi-omics analysis which do not appear to be relevant for this study (although would be definitely worth including if available).

Reply: We apologize for the earlier lack of clarity regarding the location of the codebase. We have now restructured our GitHub repository to make this more transparent and accessible. [redacted]

[redacted]

The repository now includes a comprehensive README file that outlines each component of the analysis, structured according to the order of the results presented in the manuscript.

[redacted]

Each section includes all relevant code, along with direct links to the corresponding Figures, Supplementary Figures, and Supplementary Tables generated.

Referees' comments:

Referee #1 (Remarks to the Author):

Thank you to the authors for their efforts in improving this manuscript and for responding to my earlier comments. While the paper has been strengthened in certain areas, I continue to have serious concerns about several key methodological issues.

Reply: We thank the reviewer for the thorough evaluation of our methodology. Please find below our responses to each specific question, broken down into several smaller parts, followed by a comprehensive description of our revised analyses and updated methodology.

Q1.1 Unaddressed concern over the robustness of the findings in my comments 3, 5 and 6. My main concern regarding the robustness of the findings, raised previously in comments 3, 5, and 6, has not been addressed. The authors claim that their “sequential association analysis” mimics stepwise regression or fine-mapping. While this analogy may make sense in genetics, where SNPs are largely independent and the linkage structure is well characterized, it is not appropriate for phenotype-based observational data. In such data, stepwise conditioning can inflate type I error rates due to repeated testing and model selection, overfit the data, particularly when the sample size is small relative to the number of phenotypes (which is the case in this study), and distort effect estimates, as variable inclusion depends on prior P-values rather than substantive reasoning. This practice leads to unstable and data-dependent inference, which is problematic for a paper intended for a high-impact journal.
The analogy to GWAS fine-mapping is also conceptually flawed. In GWAS, conditioning on top SNPs helps disentangle causal variants within linkage disequilibrium blocks, where the correlation structure is known. In contrast, the “phenotypes” in this study (e.g., birth mode, feeding type, antibiotic exposure) are interrelated, and causal directionality is unclear. Sequential conditioning risks treating mediators or colliders as confounders, resulting in biased estimates.

Reply 1.1: We thank the reviewer for their insightful comments regarding the sequential association analysis. We agree that the analogy to GWAS fine-mapping was not appropriate. In this observational setting, the significance of any one variable depends strongly on which others are included in the model, and stepwise selection based on P-values allows random fluctuations in these interdependencies to shape model structure, which can inflate false positives, yield unstable effect estimates, and increase the risk of overfitting.

Hence, to follow-up on the reviewer’s suggestions, we have now removed the sequential association framework and instead implemented a unified penalized mixed-

effects model in which all predictors are estimated jointly under regularization, rather than selected iteratively. Specifically, the penalized regression framework introduces a regularization term that shrinks regression coefficients toward zero and constrains their magnitude. This coefficient shrinkage reduces the instability in effect size estimates that arises when correlated predictors compete to explain overlapping variation and limits model complexity, thereby mitigating overfitting. Moreover, by avoiding P-value–driven variable selection, the penalized framework mitigates bias introduced through data-dependent model-building and enhances the robustness and interpretability of the analysis, which was the reviewer's major concern. Details of this method can be found below in section *1.3 New penalized regression framework replaces earlier sequential association analysis.*

The results of this new analysis confirm, as before, that in addition to time and technical parameters, mode of delivery is the most important factor shaping the gut microbiome, followed by feeding mode.

Q1.2 Furthermore, the statement that Bonferroni correction was applied "in both the initial and corrected rounds" lacks clarity: the correction should be applied across all models and rounds. Otherwise, the family-wise error rate will be inflated. Bonferroni correction also assumes test independence, an assumption unlikely to hold given the correlated nature of both the phenotypes and microbial factors. Therefore, the claim that results were "adjusted for multiple comparisons" does not convincingly demonstrate proper control of false positives. I remain concerned that many reported associations are likely false positives and that this revision did not adequately address this fundamental issue.

Reply 1.2: We agree. As discussed above, we have now replaced the sequential association analysis with a penalized regression framework that more appropriately accounts for collinear predictors and does not rely on Bonferroni correction. The details of our method are further elaborated below in section *1.3 New penalized regression framework replaces earlier sequential association analysis.*

Q2.1 The authors' response to comment 4. The justification for selecting ten factors based on a subjective "balance between interpretability and fit" is insufficient without additional evidence from model selection steps such as marginal likelihood, cross-validation, or held-out likelihood. The explanation that an "elbow" appears at factor six, while only the first five are biologically meaningful, yet ten are retained for "flexibility," reflects internal inconsistency and suggests potential overfitting.

Reply 2.1: We agree with the reviewer that the previous selection criteria did not stem from a quantitative assessment and thus may have led to overfitting in which later factors explain fractions of variation with little biological significance. To avoid this, we have now revised the methodology, and included a stability analysis to determine factor

consistency on different data subsets for different factor numbers. This analysis allowed us to assess the stability and reproducibility of the inferred factors across these subsampled datasets and to use the consistency of the latent structure as an objective criterion for factor retention. Based on this analysis, we retained only those factors that were reproducibly recovered, thereby providing a more quantitatively supported determination of model dimensionality while also reducing the risk of overfitting.

Based on this analysis, the final number of factors was determined to be three (in contrast to the 10 used in the previous version), as described in detail below in section *1.1 Factor stability analysis and choice of appropriate number of factors.*

Q2.2 A clearer presentation of cumulative variance explained and validation of additional factors using independent data or metadata would be useful.

Reply 2.2: We agree. Following our revised selection of factor numbers based on stability (discussed above), we additionally computed the cumulative variance explained by the final factor selection. This is described below in section *1.2 Cumulative variance explained of factors.* In addition, the cumulative variance explained has been added to the Results section of the manuscript (see below). While we did not conduct this analysis on held-out data, we argue that the proposed stability analysis above-described should address any concern regarding generalizability of the findings.

Q 2.3 Moreover, the manuscript lacks evidence of model diagnostics or robustness checks; there is no indication that alternative factor numbers were tested, factor stability was evaluated, or redundancy among factors was examined.

Reply 2.3: We thank the reviewer for this comment. In response to these comments regarding model diagnostics (and as previously described), we have now included a stability evaluation framework using factor analysis on different data subsets and factor numbers. Indeed, we have now checked the model with alternative factor dimensions and assessed factor stability, and we now report the correlations among factors in the main text. This analysis is described below in detail in section *1.1 Factor stability analysis and choice of appropriate number of factors.*

Q 2.4 The reference to a “stepwise regression strategy” is even more confusing by conflating the unsupervised MEFISTO factor inference with the subsequent association testing, implying potential post hoc justification of factor number based on downstream associations. Such reasoning introduces bias and undermines confidence in the analytical rigor.

Reply 2.4: As described above, we have now removed the “stepwise regression strategy” framework in response to the reviewer’s concerns and replaced it with a unified penalized mixed-effects modeling framework. All the association analyses in

which predictor variables are used to infer MEFISTO factor dynamics and variation are now conducted within this framework. Predictors are evaluated jointly in a regularized regression model, rather than being conditionally selected based on P-values. This reduces data-dependent model-building and mitigates the inflation of type I errors associated with repeated stepwise testing. Details can be found below in section *1.3 New penalized regression framework replaces earlier sequential association analysis.*

We have also refined the methodology used for factor selection, please see section *1.1 Factor stability analysis and choice of appropriate number of factors.*

Importantly, the final number of factors (K = 3) in the revised analysis was determined based exclusively on the results of a quantitative stability assessment, i.e., no downstream biological interpretation, phenotype association testing, or external information influenced the selection of the factor number. Thus, the dimensionality of the MEFISTO model was defined independently of subsequent analyses, directly addressing the reviewer's concern about potential post hoc justification of the number of factors selected based on downstream associations.

The results of this new analysis confirm our previous associations, that beyond time and technical parameters, the most important factor shaping the gut microbiome is mode of delivery, followed by feeding mode. Any mention of other factors previously associated with pre-pregnancy smoking and parity (Factors 4 and 10) has now been removed from this section. The previously reported association of Factor 5 with delivery place is now identified in Factor 1, based on the results of the penalized regression framework.

Details of the new MEFISTO analysis (pertaining to Q1 and Q2)

1.1 Factor stability analysis and choice of appropriate number of factors

To define the number of factors, MEFISTO factor models were fitted with varying numbers of latent factors (2–12). We then explored models spanning the 2–12 factors, extending well beyond the previously observed elbow at six factors to ensure that higher-dimensional solutions could be rigorously evaluated. For each dimensionality, 15 independent subsampling splits were generated by selecting 80% of individuals, without replacement (i.e., each sample was included a maximum of one time in each subsample). Each subset was used to refit the model. Factor reproducibility was then quantified using pairwise Pearson correlations across all combinations of subsampling splits for:

(i) Factor weights (W), following factor matching (e.g., Factor 3 in one iteration discovered as Factor 4 in another) and sign alignment to account for rotational and sign indeterminacy.

(ii) Factor scores (Z), evaluated across timepoints after matching and sign alignment.

This framework directly measures the stability of the inferred latent structure as a function of model dimensionality (i.e., the number of factors estimated in the model).

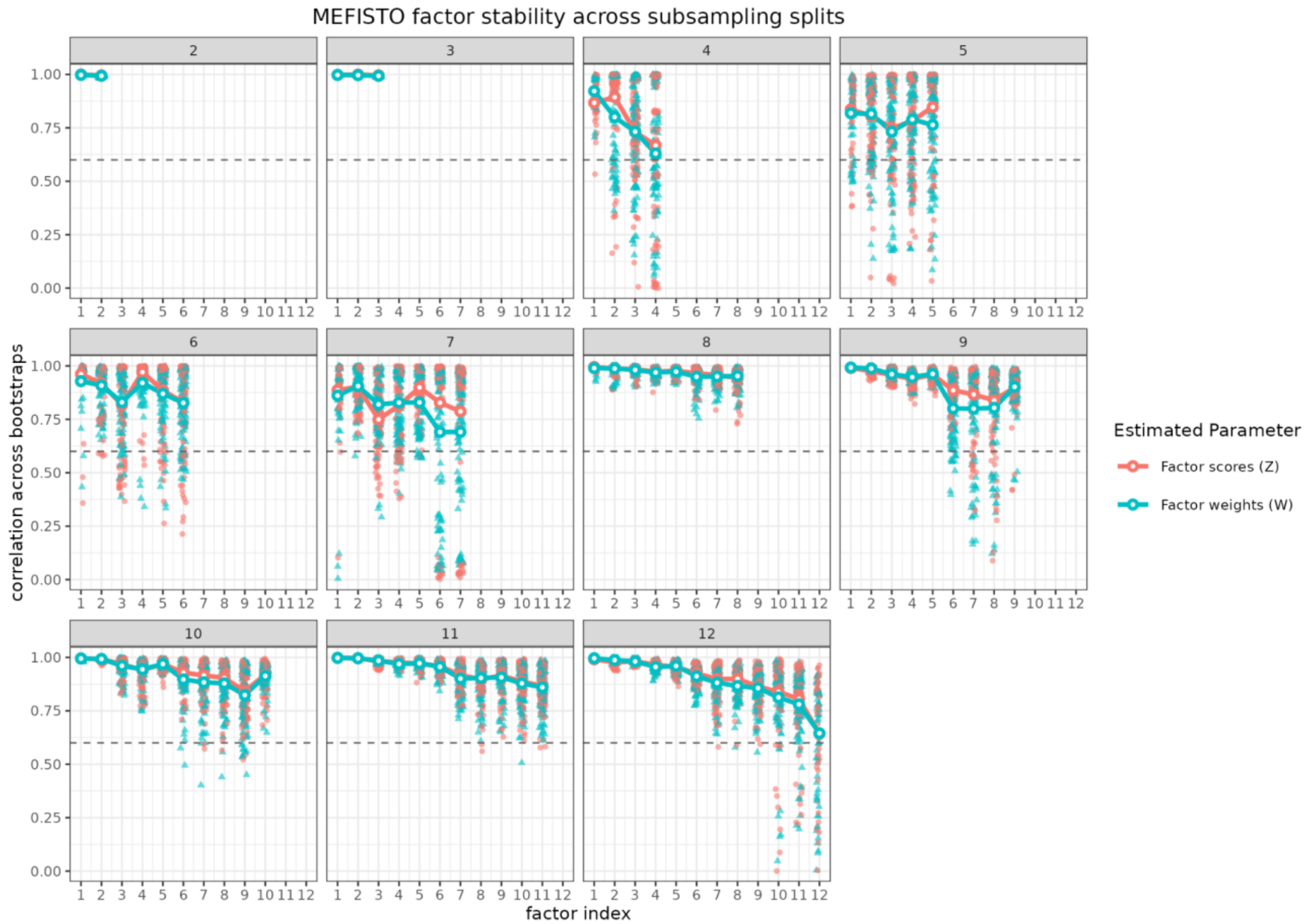

Figure R2a: Factor stability analysis. Each panel shows the results of 15 random subsampling splits in which 80% of individuals were selected, without replacement, from the full infant microbiome dataset. For each factor dimensionality (2 to 12 factors), pairwise correlations were computed across all combinations of splits for the estimated factor scores (Z) across individuals and factor loadings (W) across features. Red and teal points indicate correlations for Z and W, respectively, across all combinations of splits per factor. Higher values indicate greater stability of the inferred Z and W parameters.

The three factor solution exhibited near perfect reproducibility of both factor weights and scores across the subsampling splits (Figure R2a). In contrast, the models with four or more factors demonstrated marked reductions in stability and substantially increased variability in most cases, consistent with overparameterization and diminished model robustness. Interestingly, the eight factor solution also displayed a surprisingly high consistency, but we gave priority to the three factor solution as a simpler and more

reproducible model. Based on the outcomes of these analyses, we selected the three factor model as the most stable and robust.

Importantly, the selection of the number of factors (K = 3) was based exclusively on the results of the quantitative stability assessment, so no downstream biological interpretation, phenotype association testing, or external information influenced factor number selection. Thus, the dimensionality of the MEFISTO model was determined independently of subsequent analyses, directly addressing the reviewer's concern about potential post hoc justification of the factor number selected based on downstream associations.

1.2 Cumulative variance explained by factors

The three selected factors explain 8.27%, 4.73%, and 3.88% of total variance, respectively (19.4% cumulative variance). It is important to note that, as MEFISTO is a factor model, it does not impose orthogonality among factors, and individual factor contributions reflect marginal model-based estimates. Thus, the 19.4% is not additive and represents the joint variance explained by the full latent model.

1.3 New penalized regression framework replaces earlier sequential association analysis

In response to the reviewer's criticism of the sequential association analysis we had used previously, we have replaced it with a penalized regression framework. In this revised analysis, the three inferred factor values are modeled as outcomes, allowing us to quantify the relative contributions of biological predictors (e.g., mode of delivery or feeding), temporal variables (e.g., time), and technical covariates (e.g., sequencing depth) to the variation observed in each latent factor. This regularized modeling strategy reduces the risk of overfitting and avoids data-dependent variable selection, providing a more robust and statistically principled assessment of predictor contributions.

1.4 Robustness of the penalized regression models

To further ensure the robustness of the penalized regression and its ability to identify predictors shaping factor score variation, we tested two elastic-net mixing parameters (α = 1.0 and α = 0.5). The α = 1.0 parameter corresponds to pure LASSO, which promotes sparsity but can yield unstable variable selection when predictors are correlated, as noise in the data may change which variable within a correlated group is selected. In contrast, α = 0.5 incorporates a squared-coefficient penalty that will shrink retained and correlated predictors towards similar values, thereby alleviating instability.

Evaluating both settings provides confirmation that variable selection and the conclusions drawn from the model are robust to the choice of regularization scheme. Moreover, the regularization parameter (λ) was selected via BIC-based penalized likelihood optimization, as implemented in the *glmmPen* (v1.5.4.8) R package framework. As shown

in new Supplemental Figure S4, pure LASSO (α = 1.0) yielded more sparse models than elastic-net penalization (α = 0.5), as expected. Nonetheless, the strongest predictors were consistently retained across α values, indicating that the main biological contributors—delivery and feeding mode—were robust with respect to the penalization scheme chosen.

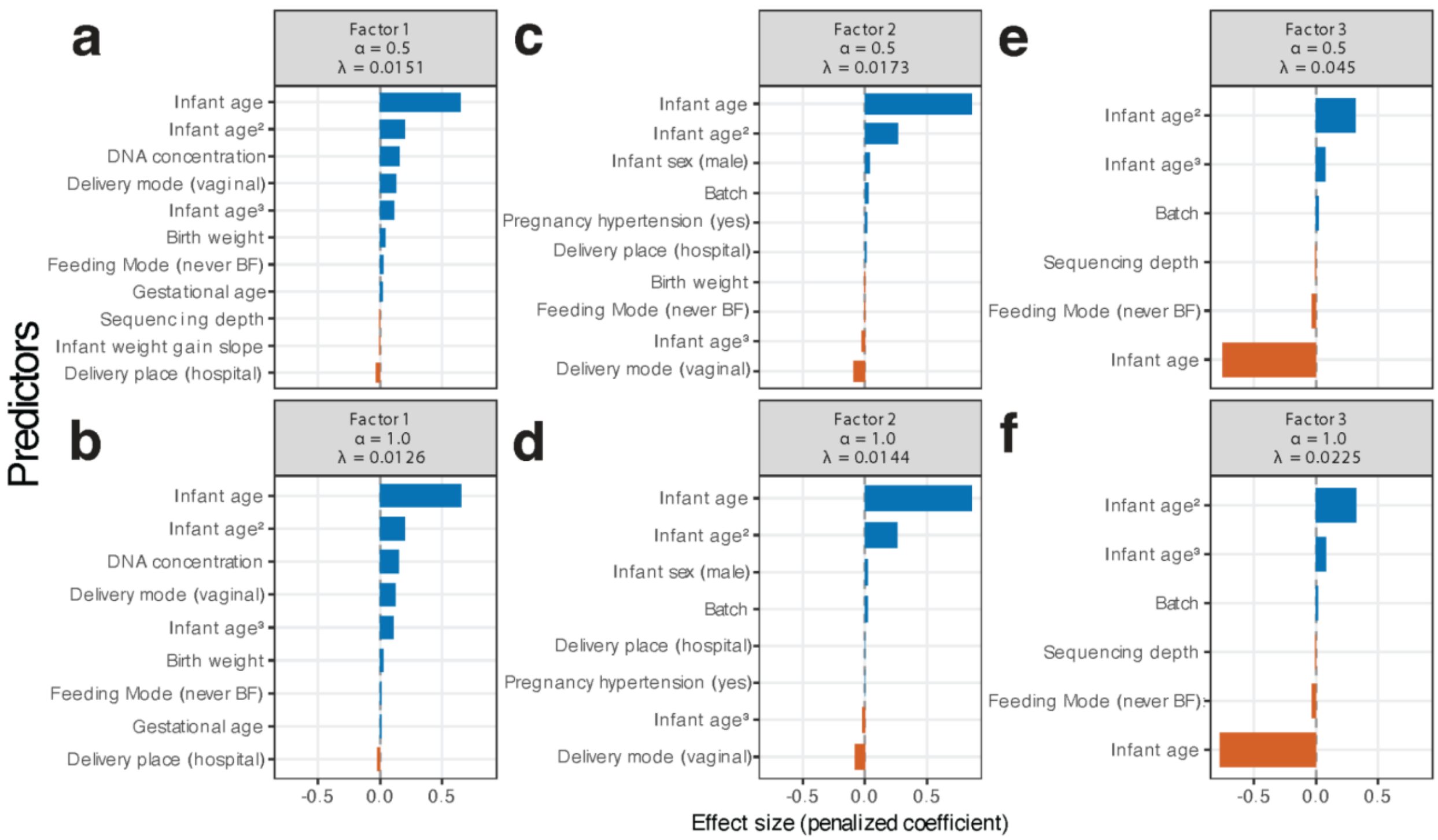

Supplementary figure S4: Penalized regression models of predictors with microbiome MEFISTO factors in the first year of life. Effect sizes of penalized regression model of predictors for Factor 1 with (**a**) α = 0.5 and (**b**) α = 1.0, Factor 2 with (**c**) α = 0.5 and (**d**) α = 1.0, and Factor 3 with (**e**) α = 0.5 and (**f**) α = 1.0.

Conclusion regarding the new MEFISTO analysis (pertaining to Q1 and 2)

We have now implemented a data-driven model-selection strategy for the MEFISTO factor model, removed potentially confounding stepwise regression procedures, explicitly separated unsupervised MEFISTO model inference from downstream modeling, and strengthened the transparency of variance reporting. We believe these revisions directly address the reviewer's concerns regarding subjectivity, overfitting, and analytical rigor.

After completing the MEFISTO analysis using the new approach, the main conclusions remain in line with the findings of the initial analysis, specifically that that mode of delivery and feeding mode are the two most important biological variables that drive microbial composition in early life.

Based on the revised analysis, we have updated the Methods and Results sections accordingly:

Methods

To understand the drivers of temporal variation in the infant microbiome, we performed a factor analysis that assesses the strength of factors (i.e., latent variables) through time[23] (v MOFA2_1.14.0), trained using CLR transformed data at SGB abundance level. It therefore permits a longitudinal analysis that estimates latent factors structuring the data across all time points and allows inclusion of samples with missing observations.

To establish a stable and appropriate number of factors for MEFISTO, we performed a resampling-based factor stability analysis. MEFISTO models were fitted across a range of factor dimensionalities, from 2 to 12 factors. For each dimensionality, 15 random subsampling splits were generated by selecting 80% of individuals from the full infant microbiome dataset, without replacement. Each subsampled dataset was used to refit the MEFISTO model, yielding estimates of factor scores (Z) and factor weights (W). For each factor dimensionality, factor stability was assessed by computing pairwise Pearson correlations across all combinations of subsampling splits. For the factor weights (W), correlations were calculated after matching corresponding factors across splits based on similarity of their weight vectors, as factor ordering is not identifiable. Factor directions were additionally aligned by sign-flipping to ensure consistent orientation across runs. For factor scores (Z), stability was assessed by first computing the mean factor score across individuals within each subsampling split. After matching and sign-aligning factors across splits, pairwise Pearson correlations were calculated for the mean factor scores across all combinations of subsampling splits at each factor number.

Together, these analyses quantify the stability of inferred factors and their associated weights as a function of the number of estimated factors. Higher correlations indicate greater robustness of the inferred latent structure, thereby informing the selection of an appropriate number of factors for downstream analyses. The three factor solution exhibited near perfect reproducibility of both Z and W across splits, indicating a highly stable latent structure. When adding four or more factors, values of Z and W exhibited lower mean correlations and substantially greater variability across subsampling splits, suggesting overparameterization and reduced reproducibility of the inferred latent structure.

Using the three factor MEFISTO model selected through stability analysis, we next modeled the inferred factor values as outcomes in penalized mixed-effects regressions to quantify the relative contributions of biological predictors (e.g., mode of delivery), temporal variables (e.g., time) and technical covariates (e.g., sequencing depth) to variation in each latent factor. In selecting predictors from all cross-sectional variables,

we included those with a maximum of 15% missingness and at least 5% variation. We then retained predictors with a Spearman's correlation not exceeding 0.5. This resulted in 10 predictors, three time variables (the first-, second- and third-degree polynomials of time) and technical variables such as DNA concentration, sequencing depth and batch number, resulting in 2,428 infant samples with no missing data in those variables. To ensure comparability of penalized coefficients, all predictors were standardized to mean zero and unit variance prior to model fitting, including continuous and dummy-coded categorical variables. To include the assessment of non-linear time in addition to linear effects of time, we used the base R function poly() to calculate the first three polynomials of time, standardized their unit sum of squares and orthogonalized their values. This results in variables of time that have a mean of zero. Thereafter, all these components of time were scaled to have a standard deviation of one, thereby matching the standard deviation of all other biological or technical variables.

This set of standardized predictor variables were then used in a penalized regression model that takes into account random effects at the level of the individual. For each factor, a model was fitted using the R package glmmPen (v1.5.4.8), which employs a Monte Carlo Expectation–Maximization (MCEM) algorithm, with adaptively increasing Monte Carlo sample sizes during optimization, until convergence. The regularization parameter lambda (λ) was selected via the Bayesian Information Criterion (BIC) using a penalized likelihood optimization built into glmmPen. Models were fitted using two elastic-net mixing parameters corresponding to pure LASSO ($\alpha = 1.0$) and a partially ridge-regularized model ($\alpha = 0.5$) to confirm that our conclusions based on variable selection were not sensitive to the regularization scheme chosen. Results mentioned in the text are for $\alpha = 0.5$. Figure S4 shows results for both $\alpha = 0.5$ and $\alpha = 1.0$.

Results

To understand the drivers of temporal variation in the infant microbiome, we performed a factor analysis that assesses the strength of factors (i.e., latent variables) through time[23]. A stability analysis across models trained with 2 to 12 factors indicated that the data were best described by three factors, (see **Methods** for more details). These three factors explained 8.27%, 4.73% and 3.88% of total variance, with 19.4% explained by the full latent model. The retained factors are moderately correlated (r = 0.40–0.58), consistent with partially overlapping but non-orthogonal dimensions of microbial variation. We then examined the contribution of biological predictors (e.g., mode of delivery), temporal variables (e.g., time) and technical covariates (e.g., sequencing depth) (**Figure S4, Tables S21 & Table S22**) to the variation in the inferred latent factors. Beyond time and technical effects, birth mode emerged as the strongest biological variable associated with Factors 1 and 2. Vaginal birth was associated with positive values on Factor 1 (penalized

β = 0.13) and negative values on Factor 2 (penalized β = -0.09), whereas CS birth showed the opposite pattern. For Factor 1, the species with the top three positive factor weights were *Bacteroides uniformis* (SGB1836 group), *Phocaeicola vulgatus* (SGB1814) and *Parabacteroides distasonis* (SGB1934), which were considered more representative of vaginal births (**Figure 2b**). Across all samples, we observed that the values of Factor 1 increased over time (linear time term, penalized β = 0.65). Infants delivered by CS displayed lower Factor 1 values than those vaginally delivered, and the differences between the two groups remained stable until M3, then subsequently reduced. Beyond mode of delivery, place of delivery (home versus hospital), and birthweight were also associated with Factor 1, although this effect was much smaller than that of mode of delivery (penalized β = -0.04 and 0.04, respectively). Factor 3 was associated with feeding mode (penalized β = -0.03 for never breastfed, reference = ever). The trajectory of Factor 3 also exhibited an inflection point at M9, which is when we observe a lack of clear separation between feeding-mode groups that suggests the introduction of solid foods may attenuate the feeding-related effects on this factor. Overall, these findings indicate that early-life gut microbiome development is primarily shaped by temporal dynamics and biological predictors such as mode of delivery and feeding practices, followed by minor effects from factors like place of delivery, with their effects varying in magnitude and persistence across infancy.

Q3 The authors' response to comment 7.
Original comment of reviewer: Several analyses, such as those described in Lines 354-366 and the cluster-based analysis in Lines 179-189, appear to be underpowered. Results from these tests should be interpreted with caution and may not be suitable for robust inference. Overinterpretation of these findings detracts from the overall rigor of the study.
While I appreciate the authors' acknowledgment that many tests are underpowered, this admission reinforces my concern about the overall robustness and interpretability of the findings. When combined with an analysis framework that inflates significance and increases the likelihood of false positives, the scientific validity of the reported results becomes even more questionable.

Reply 3: We thank the reviewer for their comment. We acknowledge the risk of overinterpreting underpowered analyses, as discussed during the previous reviewing round. We are also aware of the risk of reporting false discoveries in this observational study, a risk we tried to mitigate by estimating the FDR of the associations through the Benjamini-Hochberg methodology.

However, for the two instances discussed in this comment, we believe the main concern should not be regarding "false positives" because we do not report positive findings in both instances mentioned by the reviewer. On the contrary, in both cases, our concern

is the possibility of *false negatives*, and we have explicitly acknowledged that this may be due to the analyses being underpowered.

We would like to clarify the two claims made here and the methodology behind these findings:

1. *Lines 354-366*: In this analysis we associated the concentrations of human milk oligosaccharides (HMOs) with bacterial species/pathways using generalized additive models correcting for technical variables (DNA concentration, sequencing depth, and sample batch), performing this analysis only on breastfed individuals. The corresponding P-values from all associations were corrected using Benjamini-Hochberg correction.

> *Transformed species/pathway abundance ~ DNA concentration + Read Depth + Batch + HMO concentration or secretor status*

In this analysis we found no FDR-significant associations between any of the 24 measured HMOs and microbial species or pathways and laid out possible reasons for this, including lack of power. This is also clearly mentioned in the heading of the section: "Infant feeding practices, but not breast milk HMO concentrations, are associated with the infant gut microbiome and its neuroactive potential". We further elaborate on the potential reasons for the absence of these associations, referring to literature: "These results contrast with several previous smaller studies that reported associations between HMOs and the abundance and dynamics of *Bifidobacterium* species[28–30] but are in line with other studies reporting no or weak associations[31–33]. It is possible that the lack of observed associations in our study reflects limited statistical power, particularly given that individual HMOs may have small effect sizes and exhibit high variability, even within a single feeding session. Furthermore, 83.8% of infants in our cohort were breastfed immediately after birth (**Table S4**), which may have reduced variation in early HMO exposure, as the vast majority of infants were exposed to HMOs directly after birth."

Our only association that passed FDR-correction was that we observed that maternal secretor negative status (Le-Se-) (characterized by a completely different HMO profile) was associated with a lower Shannon diversity index in infants compared to other milk groups. While mentioning this we specifically mention our low sample size, and we show these results in Figure S6.

"We observed that maternal secretor negative status (Le-Se-) resulted in a lower Shannon diversity index in infants compared to other milk groups (*P*=0.006); however, the power of this analysis was low given the small number of mothers with this status (n=9 samples from three individuals; **Figure S6a**, **Table S34**). In the same group, the abundance of *Clostridium perfringens* (SGB6191) was increased, with nominal

significance (*P*=0.0001, **Figure S6a**). No FDR-significant associations with any of the 24 measured HMOs and microbial species were found (**Table S35**). Similarly, we also did not find any associations of HMOs with bacterial pathways (**Table S36**)."

Because our study constitutes one of the largest investigations of the relationship between HMOs and gut microbial composition, we consider it important to report these null findings. Given our use of mixed models adjusting for technical covariates, restriction to breastfed infants, and stringent multiple testing corrections, the absence of significant associations provides meaningful evidence that, at least within this relatively homogeneous cohort, individual HMO concentrations may not exert strong detectable effects on microbial species or pathways. Reporting these null results is essential to provide a balanced view of the evidence, to draw attention to the contrast with previously reported associations from smaller, and to inform realistic expectations regarding effect sizes and statistical power in future investigations.

2. *Lines 179-189*: In this analysis we assessed whether the infant gut microbiome at W2 was deterministic of the later infant gut microbiome composition. Here, we display the AUC resulting from leave-one-out (LOO) cross-validation combined with XGBoost predictions. In this analysis we observed low predicted class probabilities and relatively low AUC values, which led to our conclusion that the W2 infant microbiome is not predictive of the late microbiome maturation. Following the reviewer's last comment, we also specify that our study might still be underpowered to capture this effect. The results are provided in a Supplementary Figure (Figure S3) and the corresponding Methods and Results in the text read:

Methods

"Next, we assessed the community composition of the clustered samples at late time points (M6, M9 and M12) and attempted to predict the early W2 clustering based on the microbial composition at these later time points. We built a multi-class XGBoost prediction model using R's XGBoost v1.7.9.1 (objective: multi:softprob, default parameters)[89]. We applied this prediction using a compositional data analysis approach[90] by generating all possible log-ratios between bacterial species abundances with >20% prevalence in a leave-one-out cross-validation (LOO-cv) setting. We performed the same analysis using the microbial community from maternal samples at P12, at either P28 or birth and at M3."

Results

"Subsequently we assessed whether the infant gut microbiome at W2, which was largely dominated by the singular bacterial species mentioned above, was deterministic of the later infant gut microbiome composition. Cluster analysis identified eight distinct community compositions at W2, comprising a range of 65 (Cluster 1) to 12 infants (Cluster 8). Clusters 1, 2, 3, 4 and 7 were each dominated by a single highly abundant taxon: *E. coli* (SGB10068), *B. bifidum* (SGB17256), *B. longum* (SGB17248), *B. breve* (SGB17247)

or *Bacteroides fragilis* (SBG1855 group), respectively (**Figure S2**). Neither late time point communities (at M6, M9 and M12) nor maternal communities before birth could accurately predict W2 clustering (**Figure S3**), demonstrating that the developing infant gut microbiome follows a non-deterministic maturation path. However, our study might still be underpowered to capture this effect."

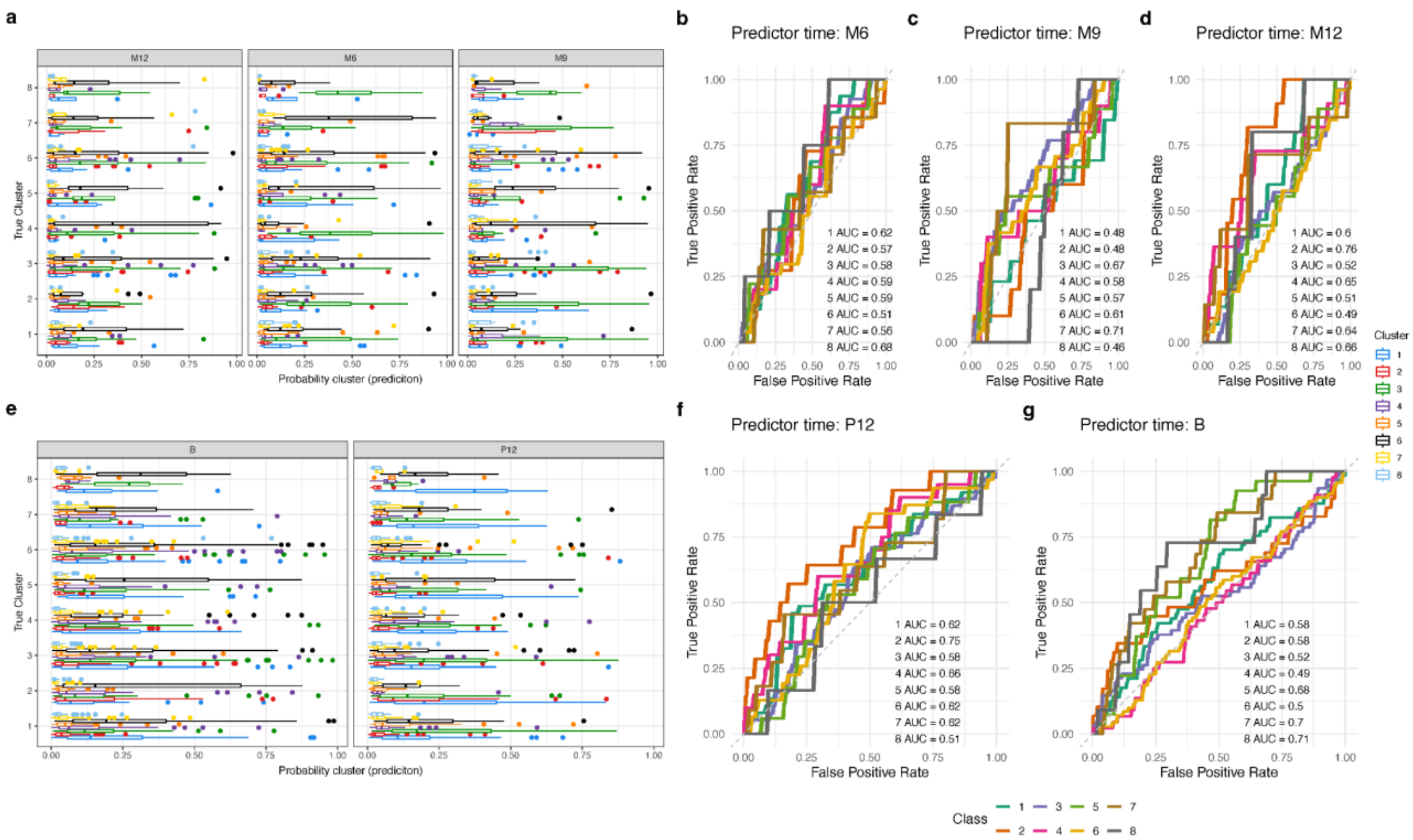

Supplementary Figure S3. Prediction of early-life clusters using late timepoints and maternal community data. a,e. Prediction of early-time point clusters using a) late-infant timepoints and e) maternal timepoints. Plots display the prediction probability per sample and per predicted class (color) per true cluster correspondence. High predictive probabilities do not correspond to cluster-belonging. **b–d,f,g.** AUC-ROC curves of the prediction of each cluster using b–d) early infant timepoints and f,g) maternal timepoints.

Q4 The response to comment 10

Original comment of reviewer:

10. Lines 480-484: Previous strain-sharing analyses (e.g., PMID: 36653448) excluded phylogenetically close MAGs of food origin (e.g., Streptococcus thermophilus, S. salivarius, S. vestibularis) upfront. The rationale for including these in the current study is unclear and should be explained.

This response also remains unconvincing. The authors have not clearly explained how dietary data were controlled in their analyses to achieve the purpose they claim. Also, it

would be helpful to know whether sensitivity analyses excluding mothers who consume yogurt were performed. The argument that S. thermophilus had “some of the lowest levels of strain-sharing” does not constitute justification for inclusion; it merely shows the species behaves differently, without demonstrating that this behavior supports the study’s conclusions.

Reply 4: We thank the reviewer for this comment and agree that our initial explanation did not sufficiently justify the inclusion of phylogenetically close, food-associated species. In previous studies, the potential to investigate transmission of such microbes is limited by cross-sectional designs or by analyses focused on infant fecal samples collected after the introduction of solid foods. In contrast, our study design includes hundreds of samples collected from infants prior to solid food introduction. This longitudinal early-life sampling provides a unique opportunity to address the important question of whether food-borne microbes present in mothers can be transmitted to infants during early life.

We further conducted an in-depth analysis of *Streptococcus thermophilus* to justify its inclusion in the strain transmission analysis. In the earlier study referenced by the reviewer (PMID: 36653448), the species genome bin (SGB) ‘SGB8002’ grouped *S. thermophilus*, *S. salivarius*, and *S. vestibularis* together, meaning that no species-specific marker genes were available for genomes from these species. In the updated MetaPhlAn database we used in our analysis, this SGB group is resolved and each of these taxa are now assigned their own SGB. This allows us to resolve the dynamics of these species with more precision.

Notably, *S. salivarius* has been included in more recent infant microbial transmission analyses conducted by the same group who had originally excluded the SGB- group due to possible food transmission (PMID: 41565819). We wondered if this could be an indication that, now that we can track this taxa independently from *S. thermophilus*, there is no evidence of food transmission. To explore this, we checked a public food-borne metagenomic catalog, curated FoodMetagenomicData (PMID: 39214080), and confirmed that this species is currently not identified among metagenomic-assembled genomes obtained from food sources. Thus, we believe that the strain cut-off computation for this species is not complicated by food transmission.

S. thermophilus is now assigned its own SGB (SGB8002), allowing us to specifically investigate this species in more detail. For this taxa, there is compelling evidence for food origin, which we confirmed by identifying several MAGs of food origin within curated FoodMetagenomicData, which we reannotated using PhyloPhlAn to the MetaPhlAn database of Jan21, confirming their assessment as SGB8002.

In principle, the reason to remove such food-derived taxa is due to the unreliability in the computation of the threshold for same-strain definition. In the original manuscript where this methodology was presented (PMID: 36653448), the conceptual framework is the

assumption that strains tend to be retained over short time-periods within the same individual, while unrelated individuals are unlikely to contain the same strain. Thus, a Youden index can be used to obtain a cut-off that maximizes the separation between the distribution of distances of unrelated samples (assumed to show a distribution peak around phylogenetic distances of unrelated strains), and the distribution of distances between longitudinal samples (assumed to show a peak around phylogenetic distances consistent with strain sharing). The inclusion of a taxa of food origin can, however, cause the assumption of unrelated individuals having different strains to not be true, since high strain-sharing is expected. This would result in poorly optimized phylogenetic distance cut-offs and a biased assessment of strain-sharing.

We evaluated the appropriateness of our strain cut-off using Youden's index. Although unrelated individuals exhibited a small peak of low distances, likely attributed to consumption of food containing clonal strains, the threshold we selected could separate the peaks of largely clonal strains and distinct lineages (Figure R2b). Moreover, even if the clonal peak observed in unrelated individuals did not exist (i.e., if we were to remove all individuals who could possibly share strains due to food source), the Youden cut-off would result in a less stringent cut-off. We believe that, as it is, our cut-off looks convincing enough to be used to discriminate between samples sharing highly similar (or clonal) strains and those that do not, which is sufficient for the aim of our analysis—to understand whether these food strains are transmitted from mother to infant or acquired horizontally from food.

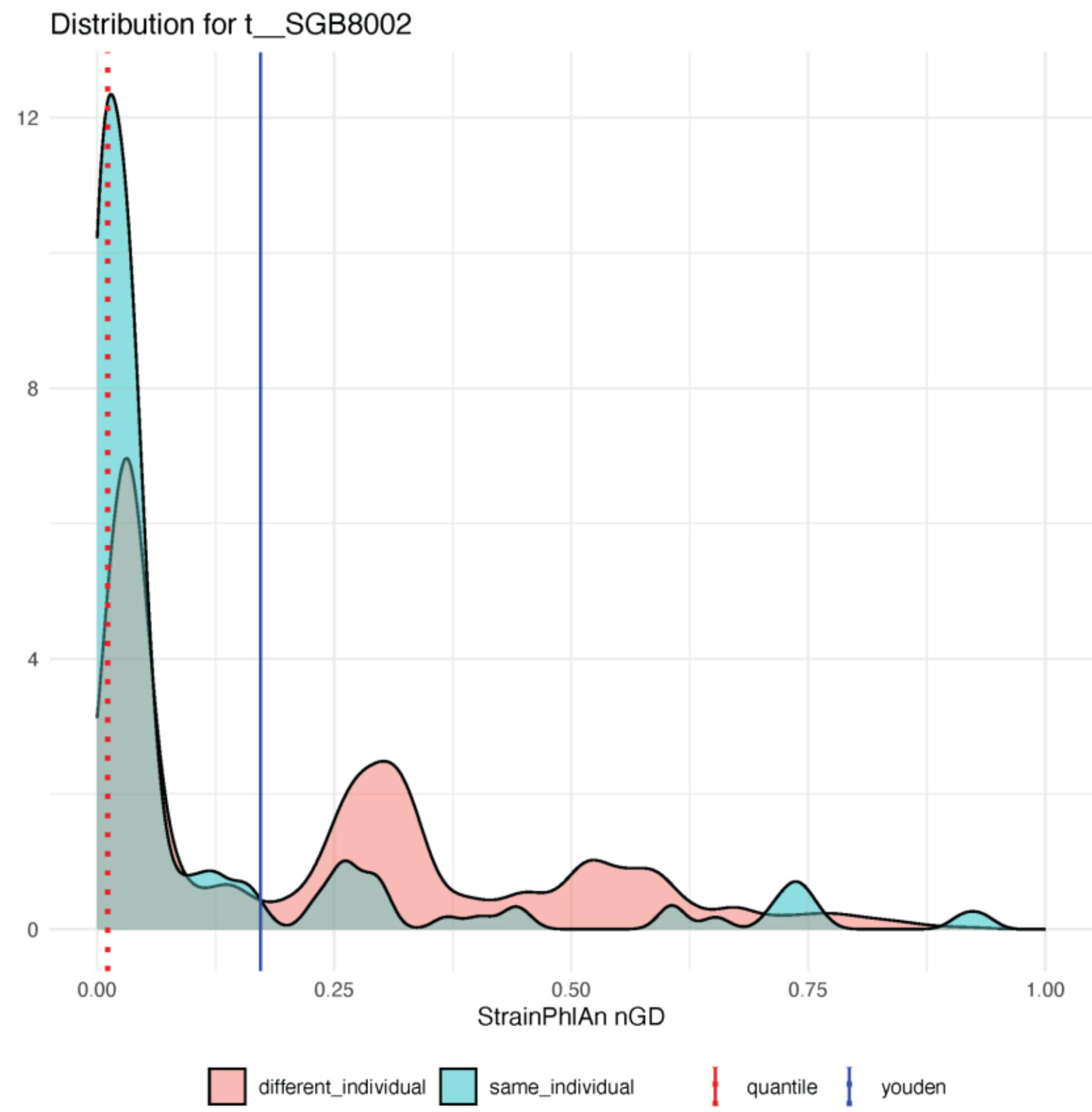

Figure R2b: Normalized distances for strain cut-off. Plot showing the normalized distances of related (blue) versus unrelated individuals (pink).

Importantly, inclusion of *S. thermophilus* did not bias our conclusions regarding early-life strain-sharing. Infants in our cohort do not consume yogurt before 6 months of age. Consistent with this, we observed 0.0% strain-sharing for this SGB between mothers and infants from W2 to M3, despite detection of the species in infants at these early timepoints. This indicates that, while young infants may harbor *S. thermophilus*, these early strains are distinct from those of their mothers and are likely not food-derived. Thus, inclusion of this species strengthens our conclusion that food-borne maternal strains of *S. thermophilus* are not vertically transmitted in the first months of life.

We further explored this by assessing the genetic similarity between infant and maternal *S. thermophilus* strains and food-borne taxa. For this, we reconstructed phylogenetic trees including 18 food-borne *S. thermophilus* assembled genomes from Carlino et al. (PMID: 39214080). In Figure R2c, we visualize the phylogenetic distances from a maximum likelihood tree (StrainPhlAn) as either a phylogeny or a 2D projection to its principal coordinates. Here we observe the presence of two deep and diverse clades, as well as shallower ones with high strain-similarity in their leaves. We defined these into three clades by running a k-means clustering algorithm (k = 3).

We identified one cluster (Cluster 3, containing the series of shallow clades) enriched for food-associated reference genomes (100% of the references fell within this cluster, Fisher's exact test $p = 0.006$). This cluster showed strain-sharing among related mother–infant pairs (mean strain-sharing rate = 0.18), lower but still detectable strain-sharing among unrelated mother–infant pairs (mean strain-sharing rate = 0.08), and enrichment at later infant timepoints compared to early timepoints (logistic regression $p < 2e\text{-}16$). These findings suggest that shared strains may arise from common dietary exposure at later infant ages.

In contrast, Clusters 1 and 2, which depict the deeper, more diverse clades, contained primarily early infant strains, and strain-sharing was rare between unrelated infants and between the few mothers harboring *S. thermophilus* strains in these clusters and their infants (0% unrelated mother–infant sharing in Cluster 1, no mothers in Cluster 2, no related mother–infant pairs in Cluster 1 or 2). This suggests that these *S. thermophilus* populations are likely not food-borne, are found in early-life, and are replaced by new strains obtained from food at later timepoints.

Lastly, regarding dietary control in mothers, while our food frequency questionnaires assess yogurt consumption over the preceding three months, they do not capture consumption at the precise time of stool collection. Therefore, a sensitivity analysis excluding recent yogurt consumers could not be reliably performed.

Taken together, we find:

- No evidence of mother-to-infant transmission of dominant, potentially food-borne, *S. thermophilus* strains during early infancy.
- Emergence of shared strains later in the first year of life (>6 months), likely reflecting shared dietary exposures.
- Inclusion of this species in the analysis allowed us to explicitly demonstrate the absence of early maternal transmission, rather than merely assume it.

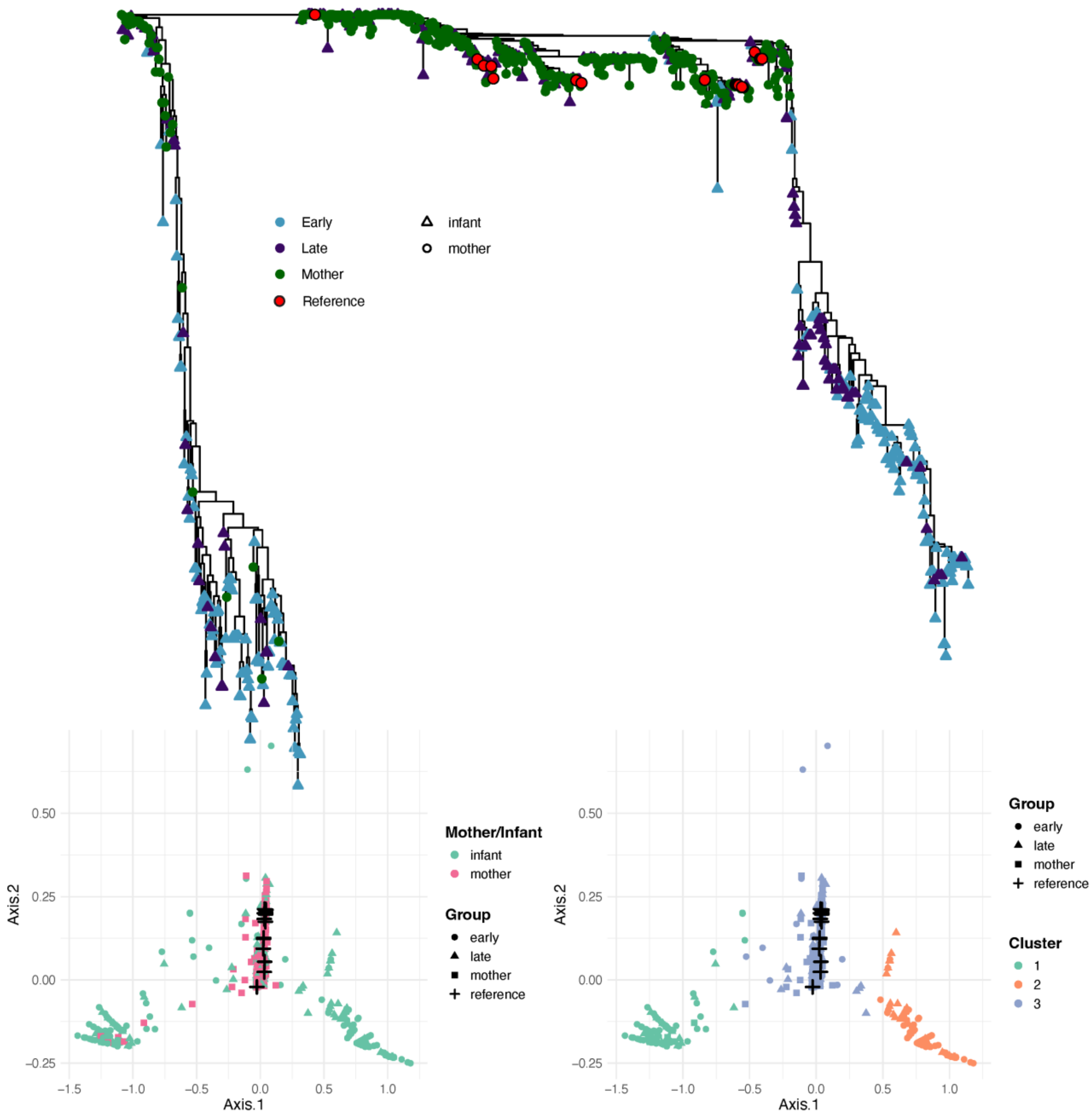

Figure R2c: Phylogenetic tree of *Streptococcus thermophilus* together with publicly available assembled genomes of food origin and PcoA plots. Colors indicate sample origin: mothers (green), infants (early timepoints (blue) and late timepoints (purple)), and references (red). The PcoA plots below depict the major axes of interindividual variability in phylogenetic distance. In the lower left panel, samples are colored by host: infant in green, mother in pink. In the lower right panel, samples are colored by cluster assignment. Shapes indicate sample type (mother, infant, or reference).

Q5. The authors' response to comment 12

Original comment of reviewer:

12. Lines 569-570:
"We then investigated the relationship between infant and maternal factors and species phylogenies, adjusted for sampling time." Adjusting for sampling time may not be the most appropriate approach given the repeated sampling design. Including time as a random effect would better account for within-subject correlations.

Q5.1 This response does not directly address my comment at all. The authors instead addressed confounding by time but not for dependence due to repeated measures. Their choice to include only one (earliest) strain per species per individual is a data simplification rather than a proper statistical solution, as it discards within-subject information, reduces power, and introduces potential bias if early versus late colonizers differ systematically. The justification that more complex models failed to converge reflects computational limitations, not methodological rationale, and is not an appropriate and justifiable response. Alternatives, such as simplifying random effects structures, using penalized likelihoods, or employing frequentist mixed models, should have been considered before discarding repeated measures entirely.

Reply 5.1. We acknowledge that our previous response did not adequately address the issue of within-subject dependence arising from repeated sampling. In our previous approach, we indeed used one (earliest) strain per species per individual, thereby discarding within-subject variance, reducing power, and potentially introducing early-vs-late colonizer bias.

In response to the reviewer's comment above, we have completely revised our statistical framework, which now allows the incorporation of multiple timepoints per individual. The new modeling strategy based on frequentist statistics uses covariates and phenotypes of interest as fixed effects, with time and individual as separate random effects. These variables are then used to infer strain-based phylogenetic variation, as the outcome of the model is now a pairwise distance representing intraspecies phylogenetic distances. Phylogenetic information is therefore no longer treated as a random effect, and the addition of Time and Individual ID as separate random effects simplifies the model. This approach also allows estimation of confidence intervals and of the significance of observed associations.

The StrainPhlAn framework we used reports one (dominant) strain per species per sample (i.e., one strain per each timepoint from one individual). Thus, frequentist mixed models are unable to analytically solve a fully-nested random intercept of the form (1|Individual/Timepoint), or unstructured VCV matrix of a the form (0 + Timepoint | Individual), as in this case the number of observations is smaller or equal than the number of degrees of freedom. To overcome this problem, we followed the reviewer's advice on using simplifying random effects structures, and tested the models to: (1) associate phylogeny with time, treating Timepoint as fixed effect and Individual as a

random effect and (2) associate phylogeny with phenotypes, treating Timepoint and Individual as separate random effects or treating Time as a fixed effect. For phenotypes-phylogeny associations, the results of models with Timepoint as fixed or random effect give overall consistent results (**Figure R2d**).

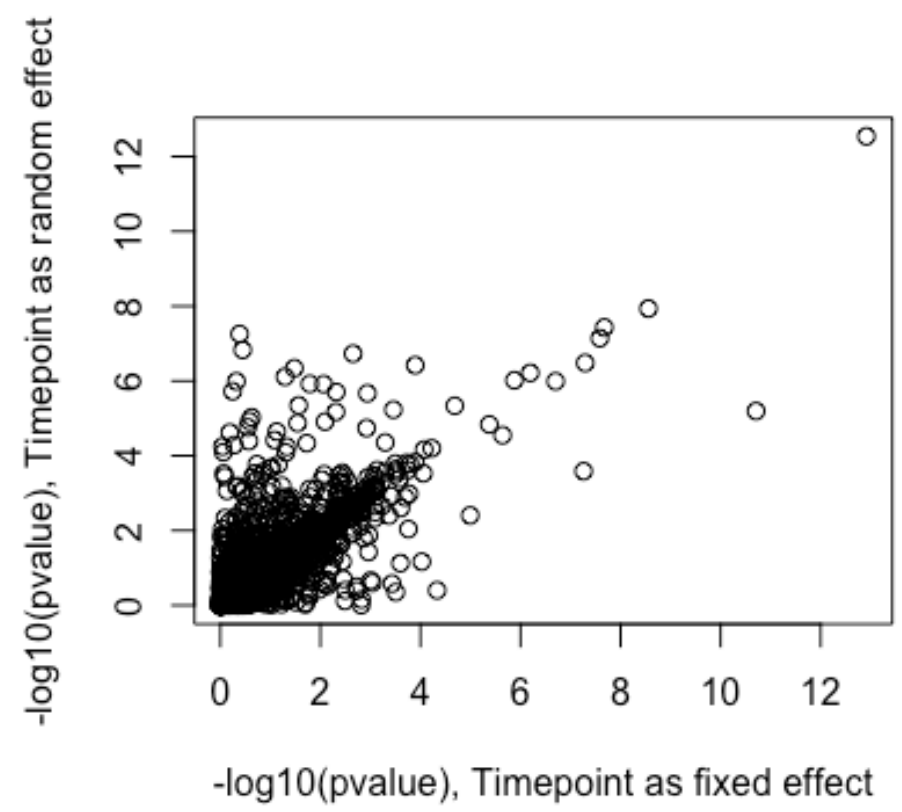

Figure R2d: Correlation between -log10 P-values of associations between phenotypes with phylogeny when treating time as a fixed effect (x-axis) and as a random effect (y-axis).

However, the models with Timepoint as fixed effect tend to be more conservative (see Table below). The 63 associations that were FDR-significant with Timepoint as the random effect and not significant with Timepoint as fixed effect are mostly related to associations with very strong collinearity between phylogeny, phenotype, and time, e.g., associations to infant weight and height. This is an expected difference for random vs fixed effect covariate inclusion, and we therefore considered adjustment for Timepoint as fixed effect more conservative and chose this approach.

		Time.as.random	
		< 0.05	≥ 0.05
**Time.as.fixed**	< 0.05	20	5
	≥ 0.05	63	6743

The analysis was conducted using the MDMR package in R (Multivariate Distance Matrix Regression, DOI: 10.1007/s11336-016-9527-8), which supports mixed-effects multivariate distance matrix regression. To further assess the robustness of the results, we performed permutation testing for all associations that passed FDR-correction, using 20,000 permutations (the number that allows empirical p-values to reach 5% FDR cutoff). Out of 25 associations tested, all were shown to be nominally significant using permutations (empirical P-value below 0.05), yet 7 didn't reach 5%FDR threshold and therefore excluded from the results.

Overall, this analysis addresses reviewers' concerns. It allows for inclusion of multiple samples from the same individual, taking into account the within-individual covariance of the strains from the same species. The estimation of the effect of time on the species' phylogeny is now also performed using the same analytical approach. This approach further allowed us to identify which phenotypes determine time-dependency of the strain, as we show for the example of Time–*S. thermophilus* dependency on yogurt and quark intake.

With this new approach, our results remain overall consistent with our earlier findings. Of the 18 bacterial species shown to be time-dependent using our previous approach, 10 remained significant (under 5% FDR) with the new approach, while 5 were nominally significant ($p < 0.05$) and only 3 lost significance. In relating species phylogeny to phenotypes, the two major drivers remained parity and infant diet, as reported previously.

We have rewritten the corresponding sections in the Methods and Results (including Tables S71 and S72) accordingly and revised Figure S10:

Methods

To associate time and maternal and infant traits with SGB phylogeny, we used multivariate mixed model distance matrix regression. RAxML phylogenetic trees were transformed to distance matrices by their branch lengths using the cophenetic.phylo function from R package ape (v.5.8-1)[103]. Next, using the MDMR package (v.0.5.2)[104] we

performed mixed model distance matrix regression treating SGB distance as outcome. To associate phylogenies to Time, we included Timepoint (factor) and technical covariates (DNA concentration and number of cleaned reads) as fixed effects and Individual ID as random effect. Associations that passed a FDR (Benjamini-Hochberg) cut-off of 5% were considered significant.

Phylogenetic distance ~ covariates + **Timepoint +** (1 | Individual)

To associate species phylogenies to maternal and infant phenotypes, we executed the models with Timepoint adjusted as fixed effect:

Phylogenetic distance ~ covariates + Timepoint + **trait** + (1 | Individual)

Quantitative predictors were normalized using inverse rank transformation. As the sample size varies for each of the trees tested (due to the species prevalence), we only included traits of interest with at least 25% variance and present in more than 100 samples.

The phylogeny-predictor associations with analytical MDMR p-values that passed FDR (Benjamini-Hochberg) control of 5% were additionally validated by performing 20,000 permutations and calculating empirical p-values. The associations were considered significant if both analytical and empirical p-value reach 5% FDR threshold.

Results

Infant microbial intraspecies phylogenies are associated with time, parity and infant complementary food

We next assessed whether infant age influences species phylogeny (see Methods). For 19 species, infant age was a significant determinant of phylogenetic variation ($P_{adj} < 0.05$). These included multiple species from the genera *Bifidobacterium* (*B. longum* SGB17248, *B. catenulatum* SGB17241, *B. breve* SGB17247), *Veillonella* (*V. dispar* SGB6952, *V. rogosae* SGB6956, *V. atypica* SGB6936), *Streptococcus* (*S. thermophilus* SGB8002, *S. mitis* SGB8163, *S. salivarius* SGB8007 group, *S. parasanguinis* SGB8071), and 9 species from other genera (**Table S71**). Many of these time-dependent species exhibited clear separation between early- and late-colonizing strains (**Figures 6a–d**). Notably, the phylogenetic structure of *S. thermophilus* was largely driven by its introduction via yogurt in later timepoints—infants who consumed yogurt acquired a dominant strain from a relatively narrow clade (**Figure 6e**). This phylogenetic specificity aligns with the previously observed associations between *S. thermophilus* abundance and prevalence and the frequency of yogurt and quark intake at M9 and M12 (**Figures 6f–6g**). Furthermore, when considering only infants with FFQ data available (M9 and M12; 93 samples in total), the

observed association between time and *S. thermophilus* phylogeny (P = 0.011) disappears after adjustment for yogurt and quark intake (P = 0.97).

Subspecies genetic variations in bacteria have previously been linked to health outcomes in adults[47–49]. We therefore examined the relationships between infant and maternal traits and species phylogenies, adjusting for sampling time. Among 6,831 associations tested, 18 were statistically significant (FDR < 0.05 for both analytical and empirical p-values, **Table S72**). Multiple species' phylogenies were associated with infant diet, including one species associated to feeding mode: *Prevotella copri* clade A (SGB1626) (Figure 6h). Phylogenetic variation of two highly prevalent species, *V. dispar* (SGB6952) and *R. gnavus* (SGB4584), were associated with parity (Figures 6i–j). These observations highlight that the effects of maternal and infant traits like feeding mode, diet and parity on the gut microbiota enforce a selective pressure, not only across species and higher-level taxa but also at the level of subspecies and strains.

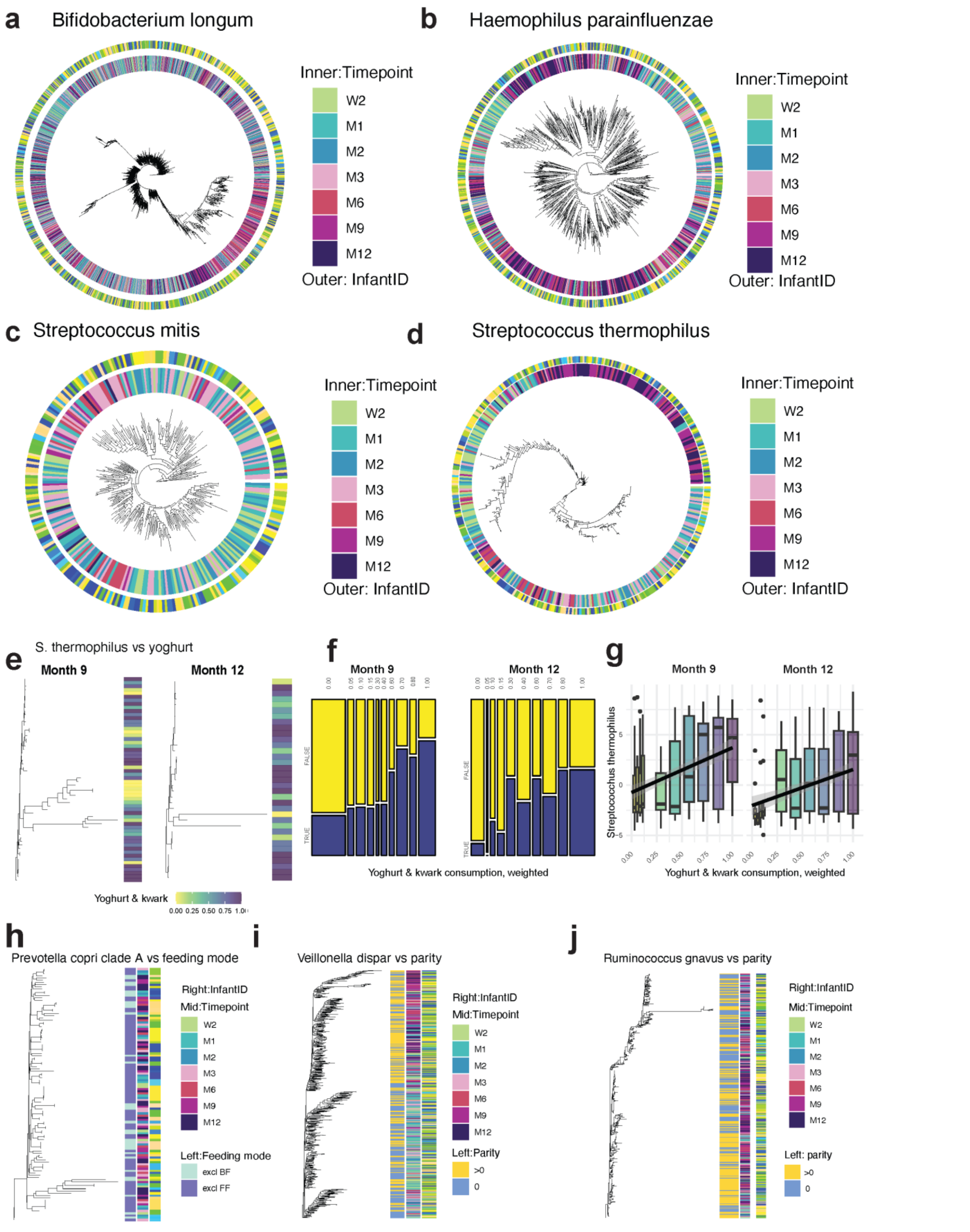

a Bifidobacterium longum
b Haemophilus parainfluenzae
c Streptococcus mitis
d Streptococcus thermophilus
Inner:Timepoint
W2
M1
M2
M3
M6
M9
M12
Outer: InfantID
e S. thermophilus vs yoghurt
Month 9
Month 12
Yoghurt & kwark
f
g
Streptococcus thermophilus
Yoghurt & kwark consumption, weighted
h Prevotella copri clade A vs feeding mode
i Veillonella dispar vs parity
j Ruminococcus gnavus vs parity
Right:InfantID
Mid:Timepoint
Left:Feeding mode
excl BF
excl FF
Left:Parity
>0
0

Supplementary Figure S10. Species phylogenetic trees and their associations with time and traits. a, RAxML tree of *Bifidobacterium longum* across multiple timepoints. **b**, *Haemophilus parainfluenzae* phylogenetic tree. **c**, *Streptococcus mitis* phylogenetic tree. **d**, *Streptococcus thermophilus* phylogenetic tree. In a, b, c, d the inner circle represents sampling timepoint, outer circle represents infant ID. **e**, *Streptococcus thermophilus* phylogenetic trees at M9 and M12. Color bar represents weighted frequency of yogurt and quark intake. **f**, Mosaic plot of the frequency of yogurt and quark intake at M9 and M12 in relation to presence of *S. thermophilus*. **g**, Frequency of consumption of yogurt and quark at M9 and M12 in relation to abundance of *S. thermophilus*. **h**, RAxML tree of *Prevotella copri clade A* in relation to feeding mode. Left bar represents feeding mode, middle bar represents sampling timepoint, right bar represent infant ID. **i,** RAxML tree of *Veillonella dispar* in relation to parity. **j**, RAxML tree of *Ruminococcus gnavus* in relation to parity. In i, j left bar represents parity, middle bar represents sampling timepoint, right bar represent infant ID.

Q 5.2 Moreover, the manuscript conflates analyses of timepoint-phylogeny and phenotype-phylogeny associations without a clear description of how repeated measures and individuals were treated in each case. This creates confusion and raises concerns about data independence. The assertion that "within-subject correlation is not an issue" is incorrect, as microbial species within an individual remain correlated even when only one timepoint per species is used. The hierarchical nature of the data, species nested within individuals, nested within families, violates independence assumptions and should be explicitly modeled.

Reply 5.2. With our new approach, described extensively above, we have also completely revised the Methods and Results section to make our approach clear. The structure of the random effects now represents the time- and individual-dependency of species phylogeny, in line with reviewers' suggestions.

Q 5.3 The authors also claim to have conducted "extensive testing" but provide no diagnostics or summary statistics to substantiate this, such as the number of models that failed to converge or comparisons of one-timepoint versus multi-timepoint analyses. Without such evidence, their explanation reads as post hoc justification rather than a transparent modeling decision.

Reply 5.3. We would like to be transparent regarding our methodology and the rationale underlying the previous analysis. However, as explained in Reply 5.1 above, we have now adopted a different approach in order to fully incorporate the reviewer's comments. Accordingly, this previous line of testing has been superseded by the more robust methodological framework described above. However, we apologize for the lack of an extensive response provided previously and here describe the benchmarks we performed.

Following on the attempt to account for repeated measurements within individuals, we incorporated the species phylogenetic tree as a random effect structured through a variance–covariance (VCV) matrix, thereby explicitly modeling phylogenetic relationships among strains. Phenotypes tested against species phylogeny were modeled as outcomes within a Bayesian framework, with parameter estimation performed using Markov Chain Monte Carlo (MCMC) sampling to obtain posterior estimates and credibility intervals. To account for repeated measurements within individuals, we also included a second random effect with an unstructured covariance specification, formulated as (0 + Timepoint | Individual). This structure allows for time-specific random effects within individuals and explicitly captures both within-individual dependence and temporal variation. However, models including both the phylogenetic VCV random effect and the full unstructured individual-by-time random effect proved computationally unstable for the majority of strain–phenotype associations we tested. We therefore explored model simplifications, including:

(1) Specifying intercept-only random effects for Individual_ID while modeling Time as a fixed effect.

(2) Reducing phylogenetic complexity by collapsing the taxonomic tree using the “same strain–different strain” cut-off used in the strain transmission analysis (**Table S57**).

Despite these simplifications, more than 80% of the tested species–phenotype associations do not fulfill formal criteria for reliable MCMC performance. In particular, of the 9,632 strain–phenotype associations evaluated, only 1,941 satisfied between-chain convergence criteria, the Markov chain convergence diagnostic, an RHat (Gelman-Rubin statistic) below 1.05, and within-chain mixing criteria of Pareto K < 0.7 in more than 70% of iterations. Thus, although a fully specified hierarchical Bayesian model is theoretically appropriate, we concluded that these were unfeasible for large-scale strain–phenotype scanning in this dataset.

Referee #2 (Remarks to the Author):

The authors have performed a thorough revision of their original manuscript. I have the following remaining comment:

Functional and pathway-level analyses: In line with my previous comments and given their role in potentially influencing health in later life, I would invite the authors to specifically check their data for the biosynthesis of immunomodulatory molecules such as LPS (e.g. PMID: 30504906) or vitamins (PMID: 35295713), and discuss their results in the context of these earlier studies.

Reply: We thank the reviewer for their thorough evaluation of this manuscript and for this suggestion. With respect to LPS biosynthesis, our COG enrichment analysis did not show a significant overrepresentation of protein families involved in canonical LPS biosynthetic pathways among the transmitted strains, despite multiple relevant COGs being tested, including COG0859 (ADP-heptose:LPS heptosyltransferase), COG0707 (UDP-N-acetylglucosamine:LPS N-acetylglucosamine transferase), COG1442 (LPS:glycosyltransferase), COG2148 (sugar transferase involved in LPS or colanic acid biosynthesis), COG3083 (PbgA/YejM, regulator of LPS biosynthesis), COG1132 (MsbA, ABC-type LPS transport system), COG3765 (WzzB/FepE family, LPS O-antigen chain-length determinant), and COG1208 (NDP-sugar pyrophosphorylase, LPS-associated nucleotide-sugar biosynthesis enzyme).

The only LPS-related COG showing significant enrichment in transmitted strains was COG1835 (OafA/YrhL family O-acetyltransferase, containing acyltransferase and SGNH-hydrolase domains), which may play a role in modifying LPS rather than in core LPS biosynthesis. Nevertheless, as previously shown (PMID: 7529745), changes in LPS acetylation can potentially affect bacterial immunogenicity and interactions with the host immune system. We have now added this to the Results and incorporate previous studies on LPS immunomodulatory potential as a reference (PMID: 30504906; PMID: 7529745).

Results

"Grouping the enriched COGs into broader functional categories revealed several functions potentially relevant to the early-life gut environment, including *carbohydrate transport and metabolism* (top enriched COGs include COG5498, Endoglucanase Acf2; COG3775, Phosphotransferase system, galactitol-specific IIC component and COG4975, Glucose uptake protein GlcU), *coenzyme transport and metabolism* (including COG1441, O-succinylbenzoate synthase and COG0854, Pyridoxine 5′-

phosphate synthase PdxJ) (**Figure S9c**) and potentially immunomodulatory functions like LPS acetylation[45,46] ”

Regarding vitamin biosynthesis, we describe an enrichment for multiple protein families involved in vitamin and coenzyme biosynthesis, including COG0854 (Pyridoxine 5′-phosphate synthase PdxJ, involved in vitamin B6 biosynthesis) and COG1441 and COG1575 (O-succinylbenzoate synthase and 1,4-dihydroxy-2-naphthoate polyprenyltransferase, respectively, both involved in vitamin K2 (menaquinone) biosynthesis), suggesting that vertically transmitted strains may contribute to the microbial potential for vitamin production in the infant gut. We have now updated Supplementary Table 70 to include COG categories, descriptions, and pathways to facilitate interpretation of the enrichment results.

Referee #3 (Remarks to the Author):

I thank the authors for addressing all my comments. I particularly appreciate the added functional analysis focusing on carbohydrate utilisation and the additional results showing limited strain sharing between mother breast milk and infant gut samples. I believe this will be a useful addition to our understanding of microbiome dynamics in pregnancy and early life.

Referee #3 (Remarks on code availability):

The authors have improved the documentation of the code, providing separate description of the scripts used in their GitHub repo for the different analyses sections of their project.

Reply: We thank the reviewer for their thorough evaluation of this manuscript and are pleased to have contributed to a better understanding of microbiome dynamics in pregnancy and early life.

Referees' comments:

Referee #1 (Remarks to the Author):

The authors have addressed all of my comments satisfactorily. I appreciate their efforts to improve the manuscript.

We thank the reviewer for their positive assessment of our revisions and appreciate their efforts and constructive feedback, which have helped us substantially improve the manuscript.

Referee #2 (Remarks to the Author):

No substantive concerns remain from my last set of comments. The only minor caveat is that the overall manuscript is now very complex, and some methods may be difficult for readers to follow without more schematic illustrations. However, this is more of a stylistic and editorial comment, and not so much a concern regarding the scientific elements of this important study.

We thank the reviewer for their positive comments and constructive feedback throughout the review process. We agree that the manuscript has become increasingly complex, particularly following the substantial revisions to our factor analyses of overall infant gut microbiome composition in relation to the studied traits, which were implemented in response to Reviewer 1's comments. To improve the clarity and interpretability of these methods, we have now added a schematic illustration to guide readers through the analytical workflow.
