# Supplementary material for: Maternal influences on infant gut microbiome and health
Source: Nature. 2026 Aug 12;658(8134):218–29. doi: 10.1038/s41586-026-10922-9 (PMC13626959; doi:10.1038/s41586-026-10922-9)
Supplement: Supplementary file 4 [file 41586_2026_10922_MOESM3_ESM.docx]

**Supplementary Results**

**Association of predictors to infant gut microbiome W2 clusters**

Following the cluster analysis of week 2 (W2) samples, we explored whether delivery mode was associated with early-life clusters of the infant gut microbiome. We found that Cluster 6, characterized by the presence of several (previously characterized) Bacteroides and Bifidobacteria, was most often seen in vaginally born (VG) infants (Fisher test, ORVG=3.71, FDR=0.03), while Cluster 5, which was not dominated by a single species, was most commonly observed in cesarean section (CS)-born infants (Fisher test, ORVG=0.27, FDR=2.8x10-3). While no significant associations were identified with Clusters 7 and 2, they were largely depleted of CS-born infants. We also observed that W2 clusters were not associated with health predictors at 3 months of age (M3), including: average crying time per day, Brussels Infant and Toddler Stool Scale, regurgitation, stool frequency, stool structure, mucus in stool, sudden inconsolable crying fits, infant length, infant weight, delivery mode and delivery place. However, cluster-belonging was associated with alpha diversity (ANOVA, P=1.34x10-11), where infants belonging to Cluster 6 had, on average, higher Shannon diversity than any other clade except 7. However, this distinct diversity was reduced by M3 (P=0.02, only Clusters 7 and 8 showing significant differences) and disappeared by six months of age (M6) (P=0.38).
