# Supplementary material for: Maternal influences on infant gut microbiome and health
Source: Nature. 2026 Aug 12;658(8134):218–29. doi: 10.1038/s41586-026-10922-9 (PMC13626959; doi:10.1038/s41586-026-10922-9)
Supplement: Supplementary file 5 [file 41586_2026_10922_MOESM4_ESM.zip › 2024-10-22869C-s4/Supplementar_table_legends.docx]

**Supplementary Information Guide**

1. Figure describing MEFISTO work process
2. Supplementary Results: Association of predictors to infant gut microbiome W2 clusters
3. Supplementary Tables 1-15 (Excel Sheets)

| Table S1. | Codebook of cross-sectional mother and infant metadata |
| --- | --- |
| Table S2. | Codebook of longitudinal mother and infant metadata |
| Table S3. | Summary statistics of cross-sectional, numeric metadata linked with infant gut microbiome |
| Table S4. | Summary statistics of cross-sectional, categorical metadata linked with infant gut microbiome |
| Table S5. | Summary statistics of longitudinal, numeric metadata linked with infant gut microbiome |
| Table S6. | Summary statistics of longitudinal, categorical metadata linked with infant gut microbiome |
| Table S7. | Summary statistics of cross-sectional, numeric metadata linked with mother gut microbiome |
| Table S8. | Summary statistics of cross-sectional, categorical metadata linked with mother gut microbiome |
| Table S9. | Summary statistics of longitudinal, numeric metadata linked with mother gut microbiome |
| Table S10. | Summary statistics of longitudinal, categorical metadata linked with mother gut microbiome |
| Table S11. | Summary statistics of mother and infant taxa of all fecal samples |
| Table S12. | Summary statistics of mother and infant taxa of fecal samples over time (long format) |
| Table S13. | Summary statistics of mother and infant taxa of fecal samples over time (wide format) |
| Table S14. | Summary statistics of the relative abundance of breast milk HMOs from all breast milk samples over time |
| Table S15. | Summary statistics of mother milk group from all breast milk samples over time |

4. Supplementary Tables 16-76

| Table S16. | Association of time point with alpha diversity in mothers |
| --- | --- |
| Table S17. | Associations of time point with maternal gut microbiome species |
| Table S18. | Associations of time point with alpha diversity in infants |
| Table S19. | Associations of time point with infant gut microbiome species |
| Table S20. | Pairwise distances between same, related and unrelated mother:mother and infant:infant pairs |
| Table S21. | Number of predictors retained in models associated with three factors. |
| Table S22. | Predictors associated with MEFISTO factors of infant gut microbiome trajectories |
| Table S23. | Cross-sectional associations of infant-specific traits with infant alpha diversity |
| Table S24. | Longitudinal associations of infant-specific trait with infant alpha diversity |
| Table S25. | Infant gut microbiome variation explained by infant-specific traits at specific time points |
| Table S26. | Infant gut microbiome variation of the combined and complete stool samples collected at month 1, 3, 6 and 12 explained by infant-specific traits |
| Table S27. | Cross-sectional associations of infant-specific traits with infant gut microbiome bacterial species at SGB level |
| Table S28. | Longitudinal associations of infant-specific traits with infant gut microbiome bacterial species at SGB level |
| Table S29. | Cross-sectional associations of infant-specific traits with infant gut microbiome pathways |
| Table S30. | Longitudinal associations of infant-specific traits with infant gut microbiome pathways |
| Table S31. | Cross-sectional associations of delivery-related traits with the infant gut microbiome |
| Table S32. | Associations of maternal human milk oligosaccharides (HMOs) with the alpha diversity of the infant gut microbiome |
| Table S33. | Associations of maternal human milk oligosaccharides (HMOs) with the infant gut microbiome |
| Table S34. | Associations of maternal human milk oligosaccharides (HMOs) with infant gut microbiome pathways |
| Table S35. | Cross-sectional associations of infant-specific traits with infant GBMs |
| Table S36. | Longitudinal associations of infant-specific traits with infant GBM |
| Table S37. | Complementary foods assessed in Lifelines NEXT infants at ages 6 (18 food groups; 49 items), 9 (21 food groups; 66 items) and 12 (21 food groups; 66 items) months. |
| Table S38. | Associations of infant dietary features with the infant gut microbiome, adjusted for feeding mode and caloric intake |
| Table S39. | Associations of infant dietary patterns, derived by Factor Analysis, with the infant gut microbiome, adjusted for feeding mode and caloric intake |
| Table S40. | Association of time with CAZyme subfamilies in infants |
| Table S41. | Infant CAZyme variation explained by infant SGB's at specific time points |
| Table S42. | Mother CAZyme variation explained by mother SGB's at specific time points |
| Table S43. | Cross-sectional associations of infant-specific traits with infant CAZyme subfamilies |
| Table S44. | Longitudinal associations of infant-specific traits with infant CAZyme subfamilies |
| Table S45. | Substrate enrichment analysis with delivery mode |
| Table S46. | Substrate enrichment analysis with feeding mode |
| Table S47. | Substrate enrichment analysis with early and late time points of infants |
| Table S48. | Cross-sectional associations of maternal-specific traits with maternal alpha diversity |
| Table S49. | Longitudinal associations of maternal-specific traits with maternal alpha diversity |
| Table S50. | Maternal gut microbiome variation explained by maternal-specific traits at specific time points |
| Table S51. | Maternal gut microbiome variation of the combined and complete stool samples collected at 12 weeks gestation, at delivery and 3 months postpartum explained by maternal-specific traits |
| Table S52. | Cross-sectional associations of maternal-specific traits with maternal gut microbiome bacterial species at SGB level |
| Table S53. | Longitudinal associations of maternal-specific traits with maternal gut microbiome bacterial species at SGB level |
| Table S54. | Cross-sectional associations of maternal-specific traits with maternal gut microbiome pathways |
| Table S55. | Longitudinal associations of maternal-specific traits with maternal gut microbiome pathways |
| Table S56. | Genetic distance species-specific threshold |
| Table S57. | Summary statistics of mother taxa of all breast milk samples |
| Table S58. | Summary statistics of mother infant sharing of SGB's |
| Table S59 | Summary statistics of strain sharing of SGB's in breast milk samples and infant gut samples |
| Table S60 | Summary statistics of mother taxa of all vaginal samples |
| Table S61. | Summary statistics of strain sharing of various SGB's in vaginal samples and infant gut samples |
| Table S62. | Overall gut strain-sharing rates |
| Table S63. | Strain-sharing rates of individual gut species |
| Table S64. | Associations of mother-infant strain gut sharing and (clr-transformed) abundance of infant species in early and late time points |
| Table S65. | Associations between maternal gut microbiome species abundance (clr-transformed) and mother-infant gut microbiome strain sharing |
| Table S66. | Odds ratios of early life (W2-M3) transmitted bacterial species persisting in the infant gut microbiome over time |
| Table S67. | Traits associated with mother-infant gut sharing rate |
| Table S68. | Traits associated with strain-sharing in individual gut species |
| Table S69 | Enriched COGs in mother to infant transmitted MAGs compared to non-transmitted MAGs |
| Table S70 | Association of infant age with phylogenetic variation |
| Table S71. | Association of infant and maternal traits with phylogenetic variation |
| Table S72. | Food items and food groups (in Dutch and English) assessed in Lifelines NEXT mothers at 2 months postpartum |
| Table S73. | Spearman correlation of cross-sectional traits linked with maternal gut microbiome |
| Table S74. | Spearman correlation of longitudinal traits linked with maternal gut microbiome |
| Table S75. | Spearman correlation of cross-sectional traits linked with infant gut microbiome |
| Table S76. | Spearman correlation of longitudinal phenotypes linked with infant gut microbiome |
